# Supplementary figures and images for: Repressor element 1-silencing transcription factor deficiency yields profound hearing loss through Kv7.4 channel upsurge in auditory neurons and hair cells (part 1 of 3)
Source: eLife. 2022 Sep 20;11:e76754. doi: 10.7554/eLife.76754 (PMC9525063; doi:10.7554/eLife.76754)

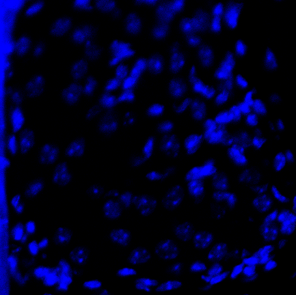

Supplement: Figure 1—source data 1. [file elife-76754-fig1-data1.zip › Figure_1_source_data/Fig.1 A/Rest cKO Apex/Rest cKO Apex SGN DAPI.tif]

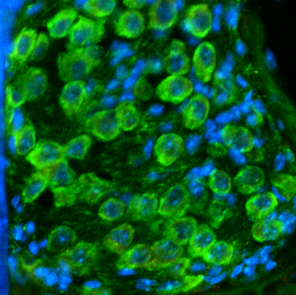

Supplement: Figure 1—source data 1. [file elife-76754-fig1-data1.zip › Figure_1_source_data/Fig.1 A/Rest cKO Apex/Rest cKO Apex SGN Merge.tif]

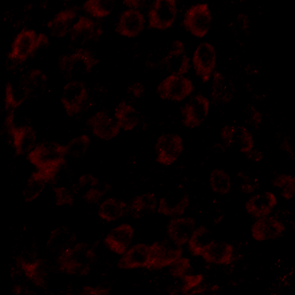

Supplement: Figure 1—source data 1. [file elife-76754-fig1-data1.zip › Figure_1_source_data/Fig.1 A/Rest cKO Apex/Rest cKO Apex SGN Rest.tif]

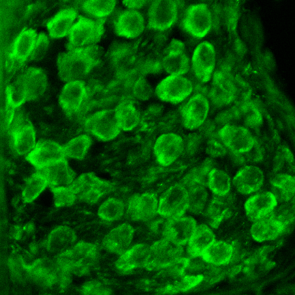

Supplement: Figure 1—source data 1. [file elife-76754-fig1-data1.zip › Figure_1_source_data/Fig.1 A/Rest cKO Apex/Rest cKO Apex SGN Tuj1.tif]

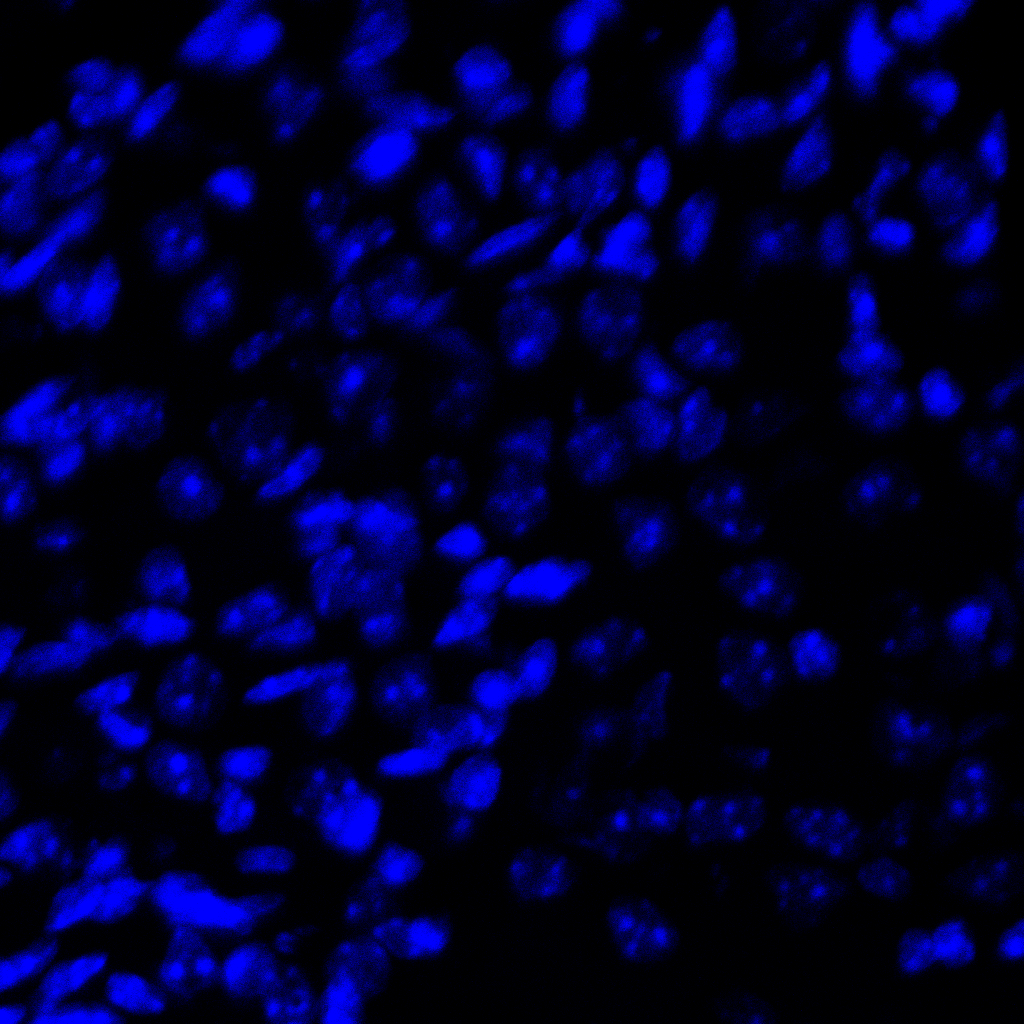

Supplement: Figure 1—source data 1. [file elife-76754-fig1-data1.zip › Figure_1_source_data/Fig.1 A/Rest cKO Base/Rest cKO Base SGN DAPI.tif]

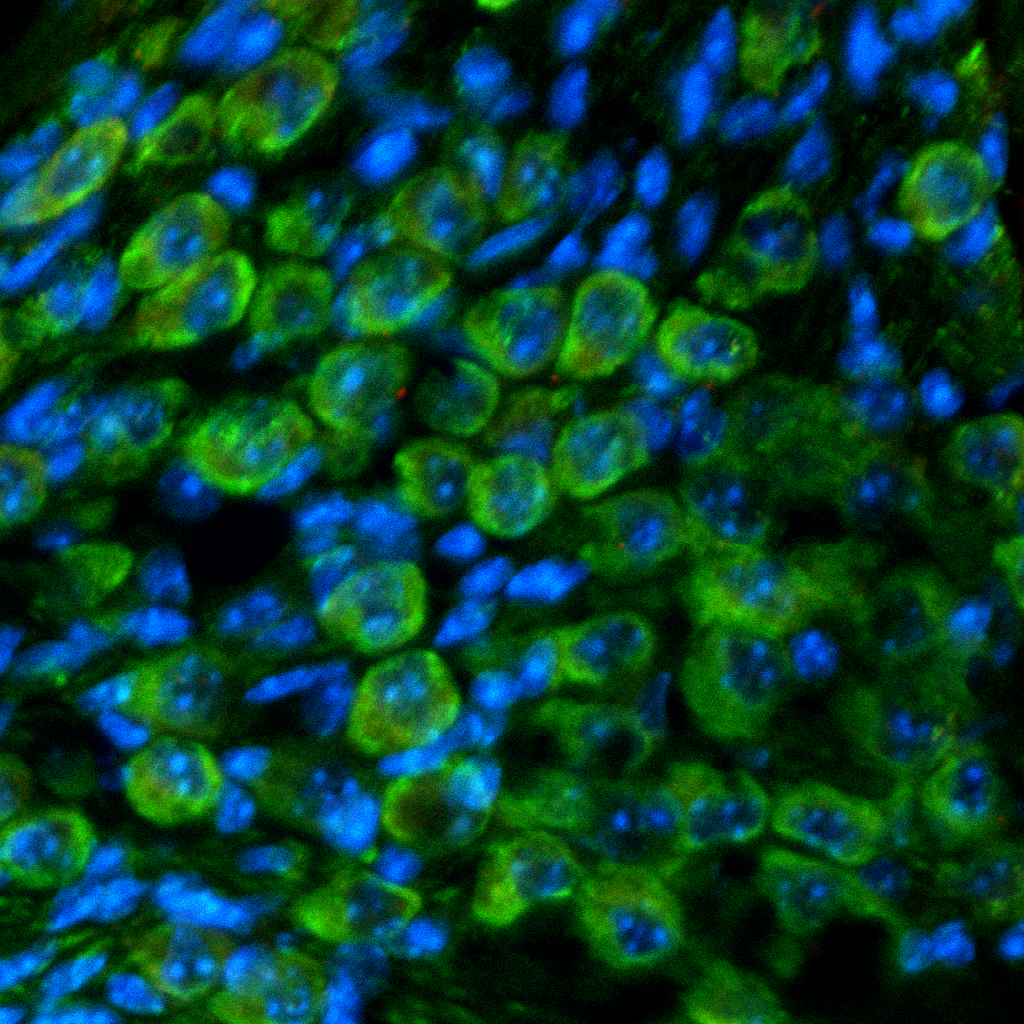

Supplement: Figure 1—source data 1. [file elife-76754-fig1-data1.zip › Figure_1_source_data/Fig.1 A/Rest cKO Base/Rest cKO Base SGN Merge.tif]

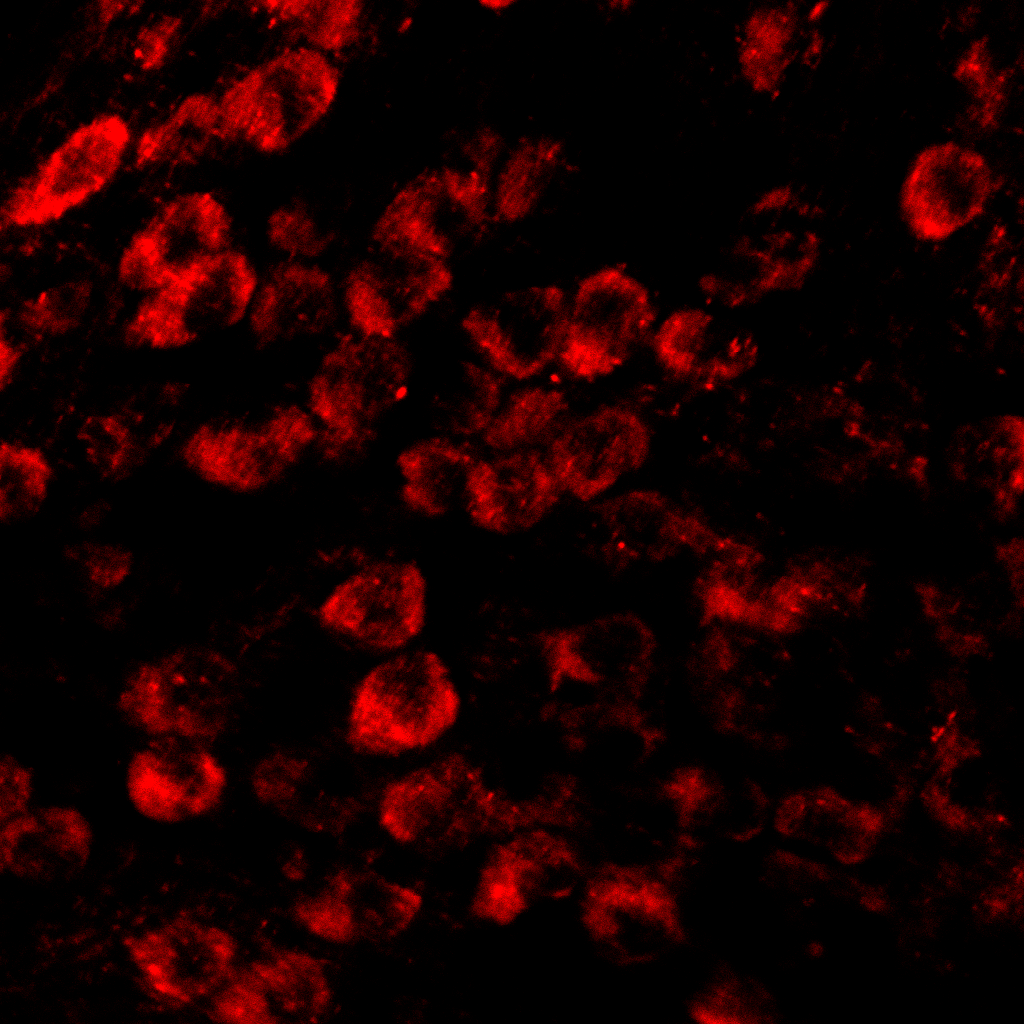

Supplement: Figure 1—source data 1. [file elife-76754-fig1-data1.zip › Figure_1_source_data/Fig.1 A/Rest cKO Base/Rest cKO Base SGN Rest.tif]

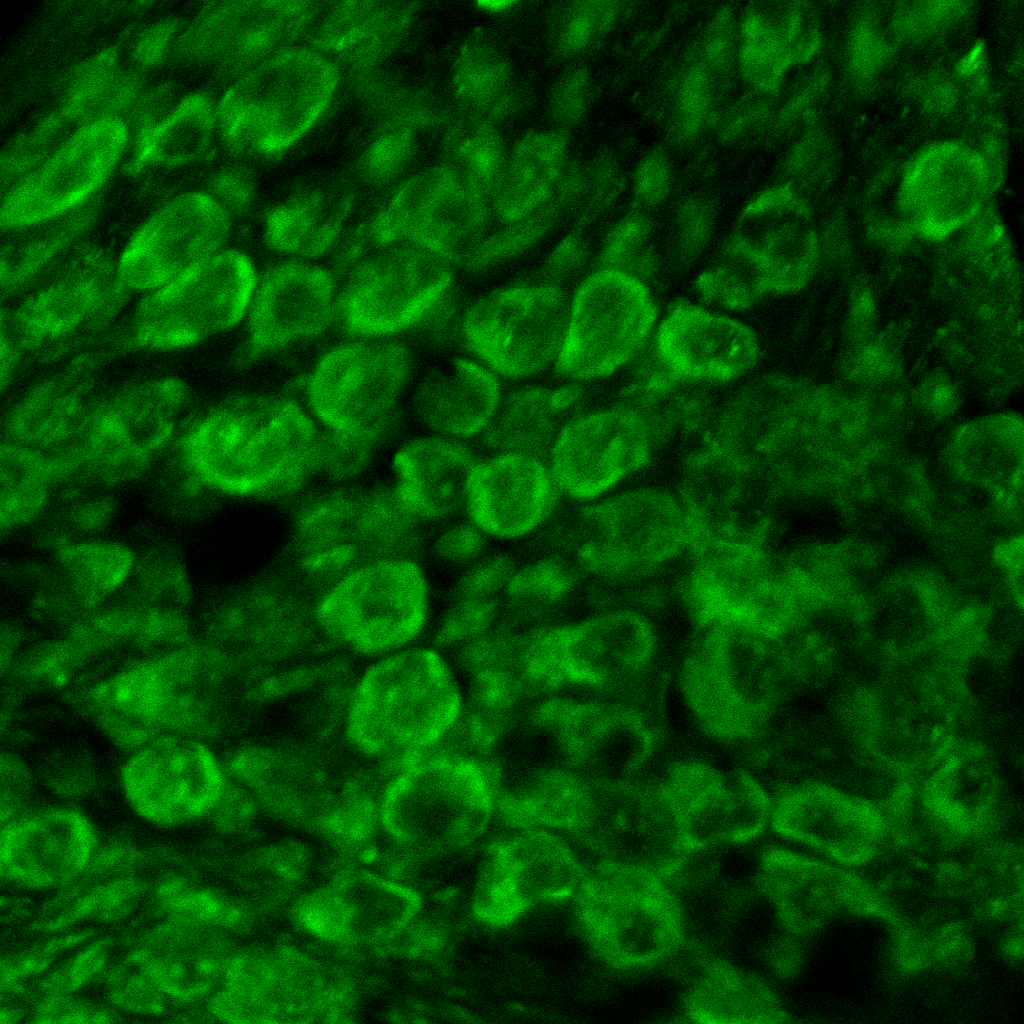

Supplement: Figure 1—source data 1. [file elife-76754-fig1-data1.zip › Figure_1_source_data/Fig.1 A/Rest cKO Base/Rest cKO Base SGN Tuj1.tif]

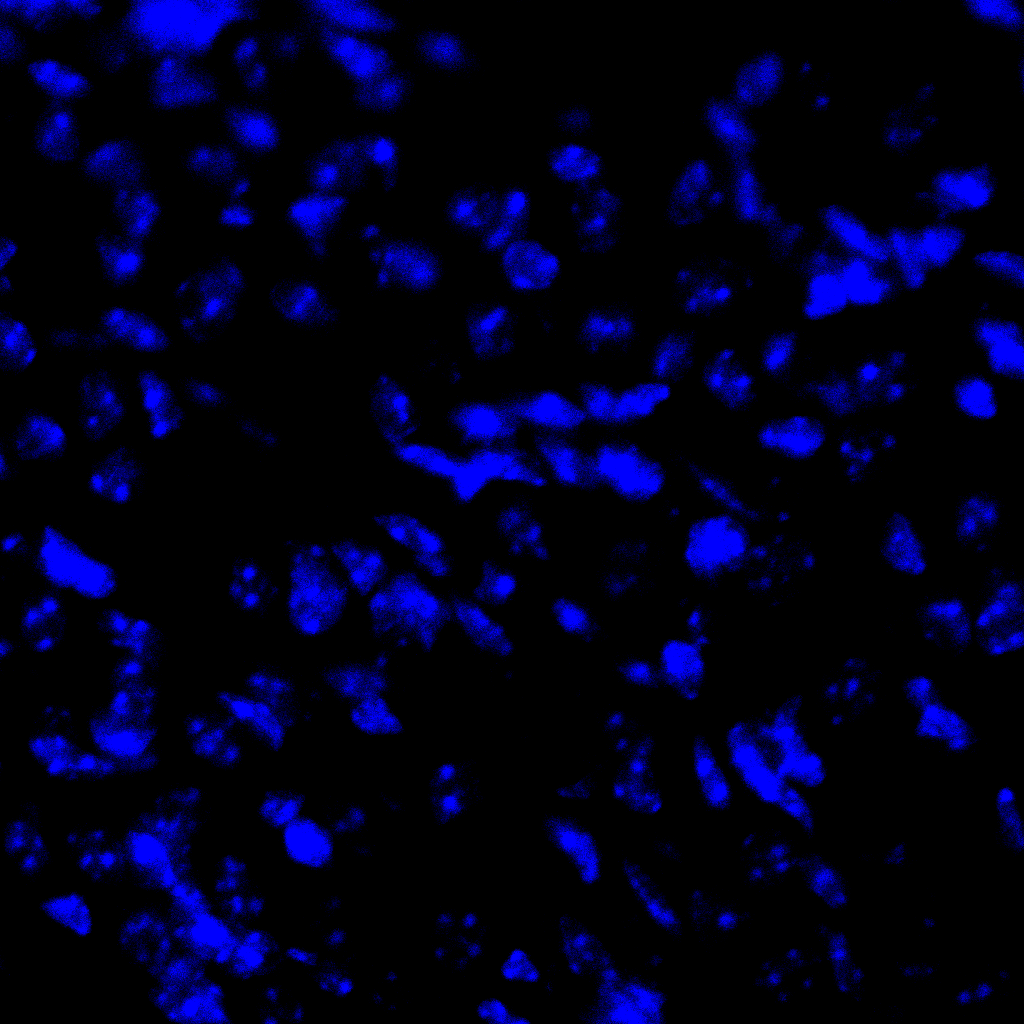

Supplement: Figure 1—source data 1. [file elife-76754-fig1-data1.zip › Figure_1_source_data/Fig.1 A/Rest cKO Middle/Rest cKO Middle SGN DAPI.tif]

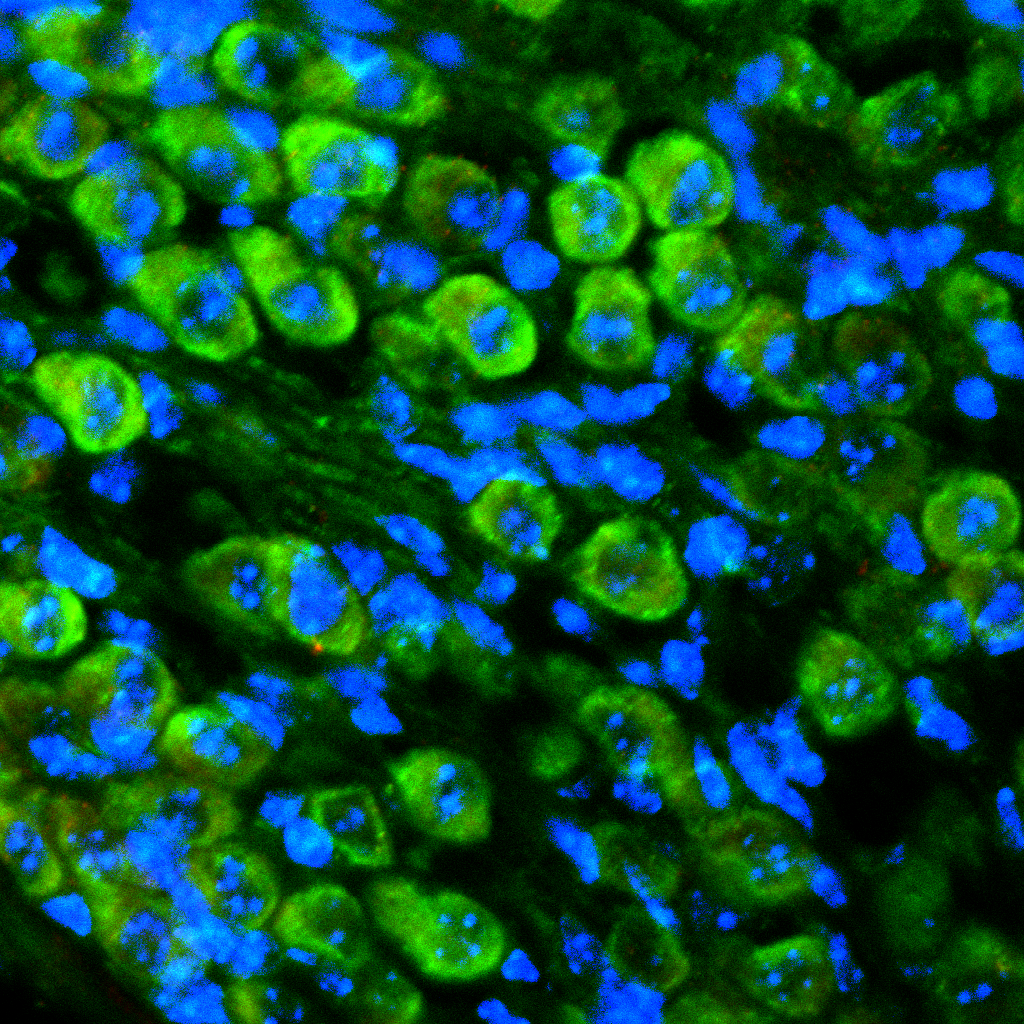

Supplement: Figure 1—source data 1. [file elife-76754-fig1-data1.zip › Figure_1_source_data/Fig.1 A/Rest cKO Middle/Rest cKO Middle SGN Merge.tif]

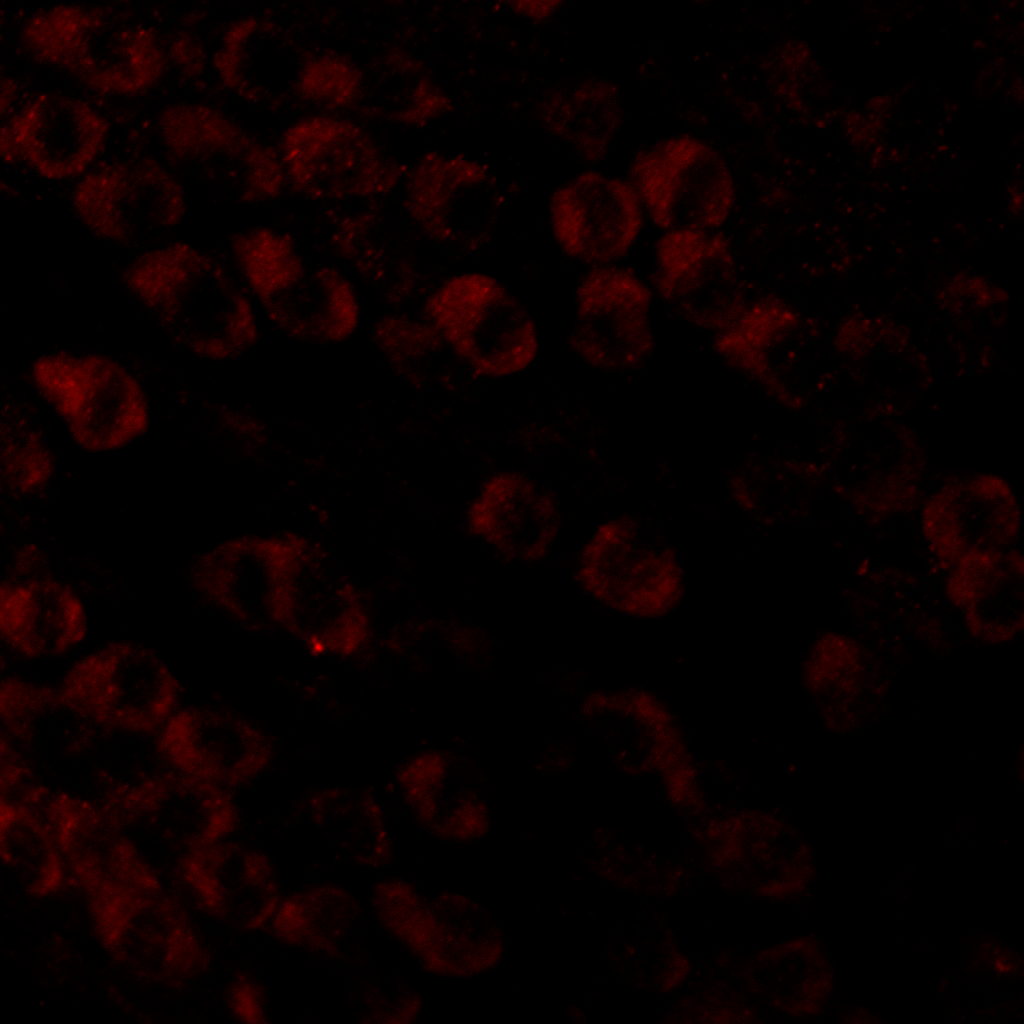

Supplement: Figure 1—source data 1. [file elife-76754-fig1-data1.zip › Figure_1_source_data/Fig.1 A/Rest cKO Middle/Rest cKO Middle SGN Rest.tif]

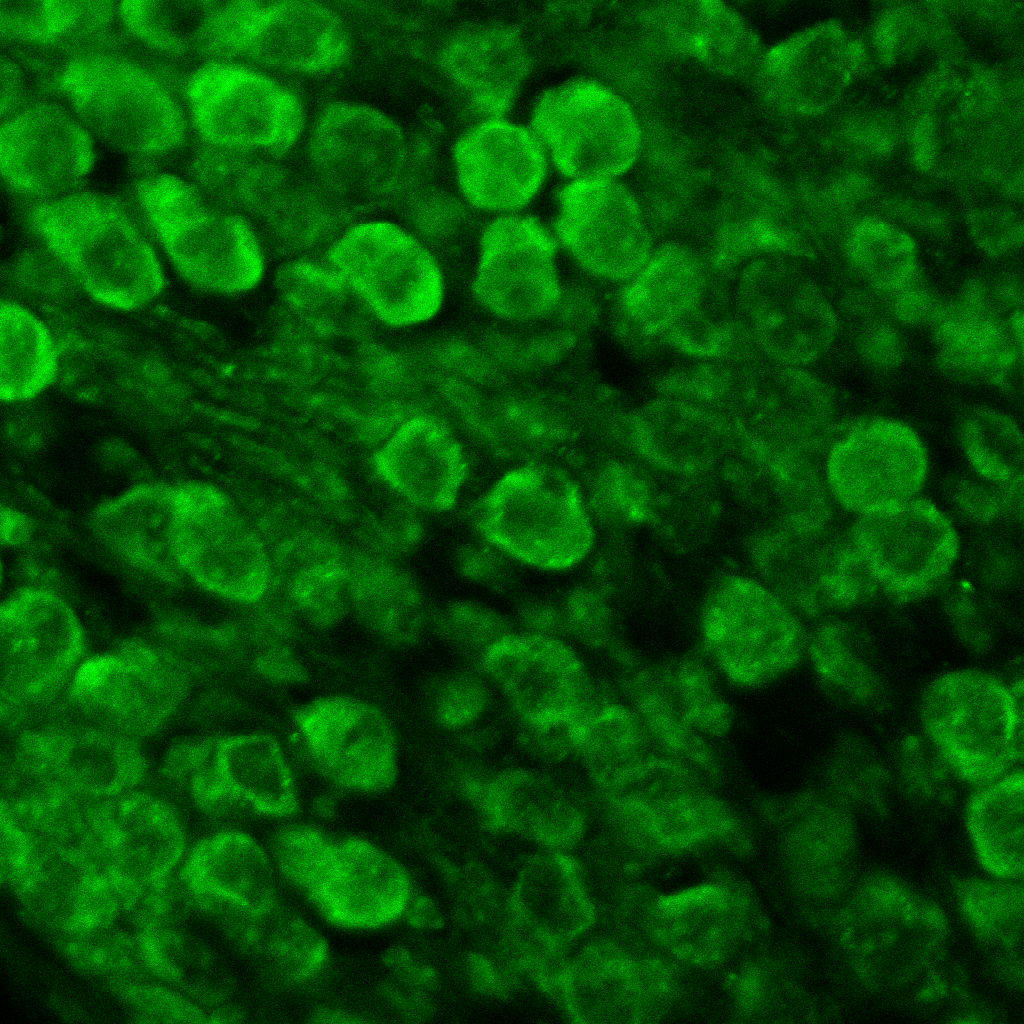

Supplement: Figure 1—source data 1. [file elife-76754-fig1-data1.zip › Figure_1_source_data/Fig.1 A/Rest cKO Middle/Rest cKO Middle SGN Tuj1.tif]

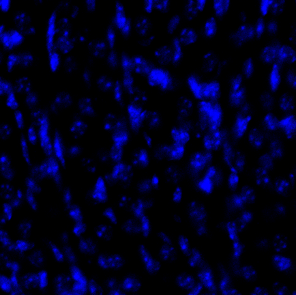

Supplement: Figure 1—source data 1. [file elife-76754-fig1-data1.zip › Figure_1_source_data/Fig.1 A/WT Apex/WT Apex SGN DAPI.tif]

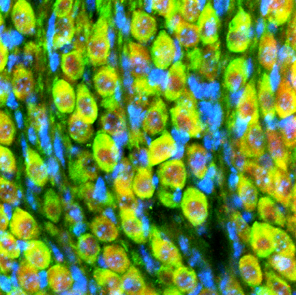

Supplement: Figure 1—source data 1. [file elife-76754-fig1-data1.zip › Figure_1_source_data/Fig.1 A/WT Apex/WT Apex SGN Merge.tif]

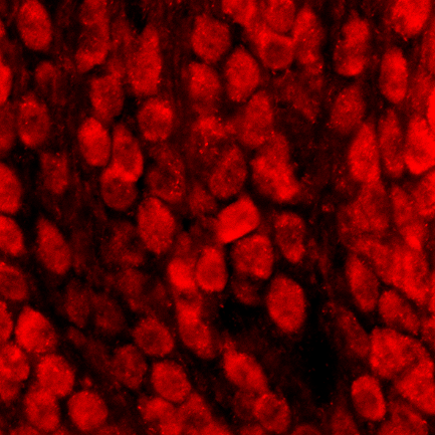

Supplement: Figure 1—source data 1. [file elife-76754-fig1-data1.zip › Figure_1_source_data/Fig.1 A/WT Apex/WT Apex SGN Rest.tif]

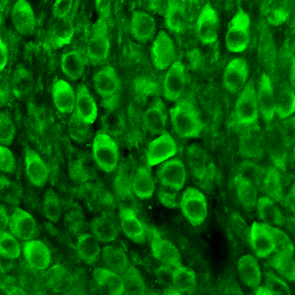

Supplement: Figure 1—source data 1. [file elife-76754-fig1-data1.zip › Figure_1_source_data/Fig.1 A/WT Apex/WT Apex SGN Tuj1.tif]

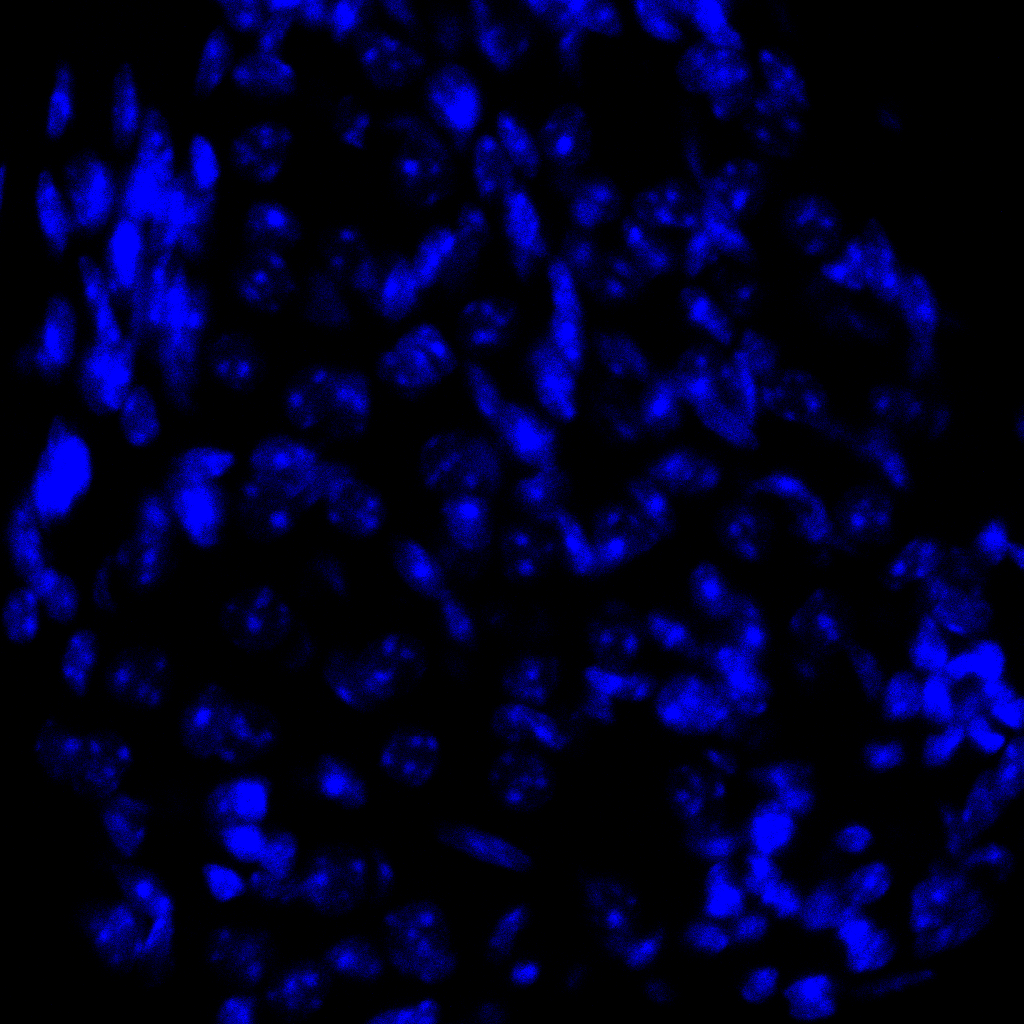

Supplement: Figure 1—source data 1. [file elife-76754-fig1-data1.zip › Figure_1_source_data/Fig.1 A/WT Base/WT Base SGN DAPI.tif]

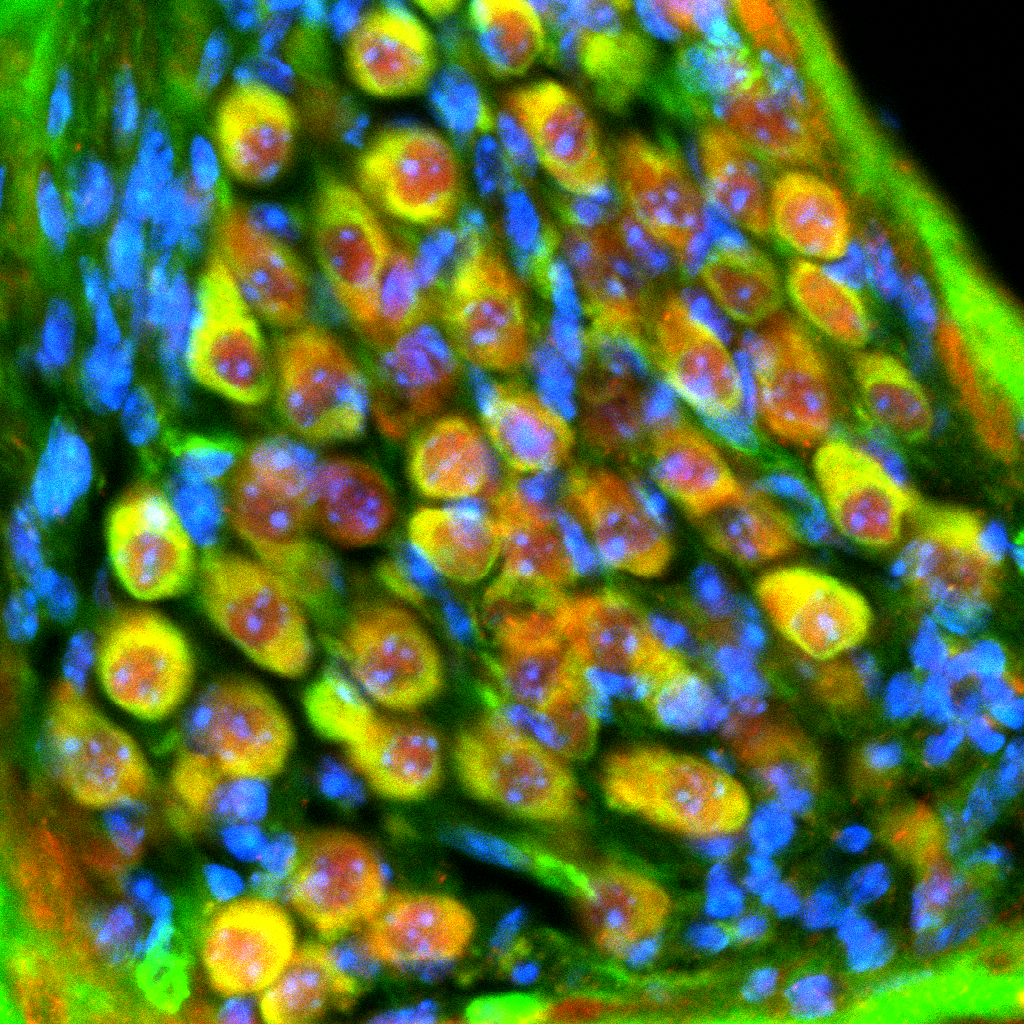

Supplement: Figure 1—source data 1. [file elife-76754-fig1-data1.zip › Figure_1_source_data/Fig.1 A/WT Base/WT Base SGN Merge.tif]

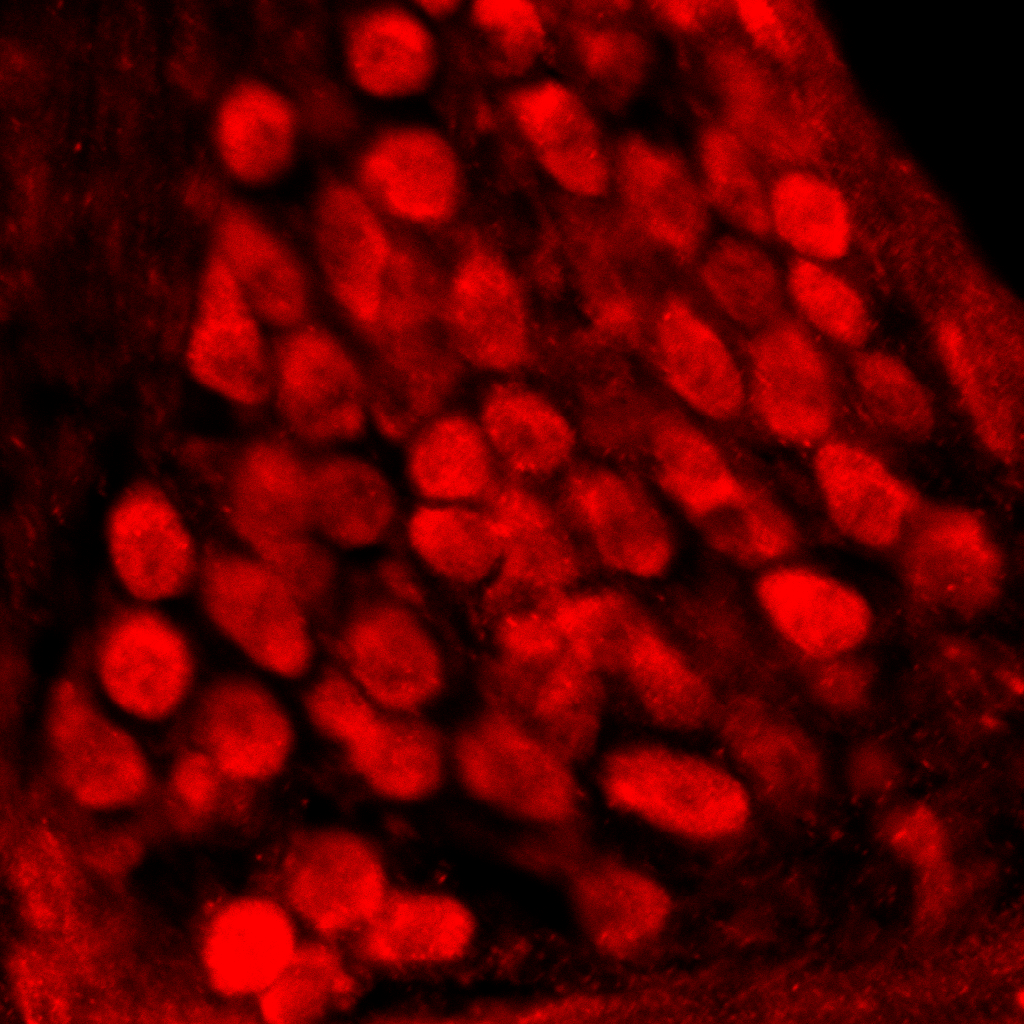

Supplement: Figure 1—source data 1. [file elife-76754-fig1-data1.zip › Figure_1_source_data/Fig.1 A/WT Base/WT Base SGN Rest.tif]

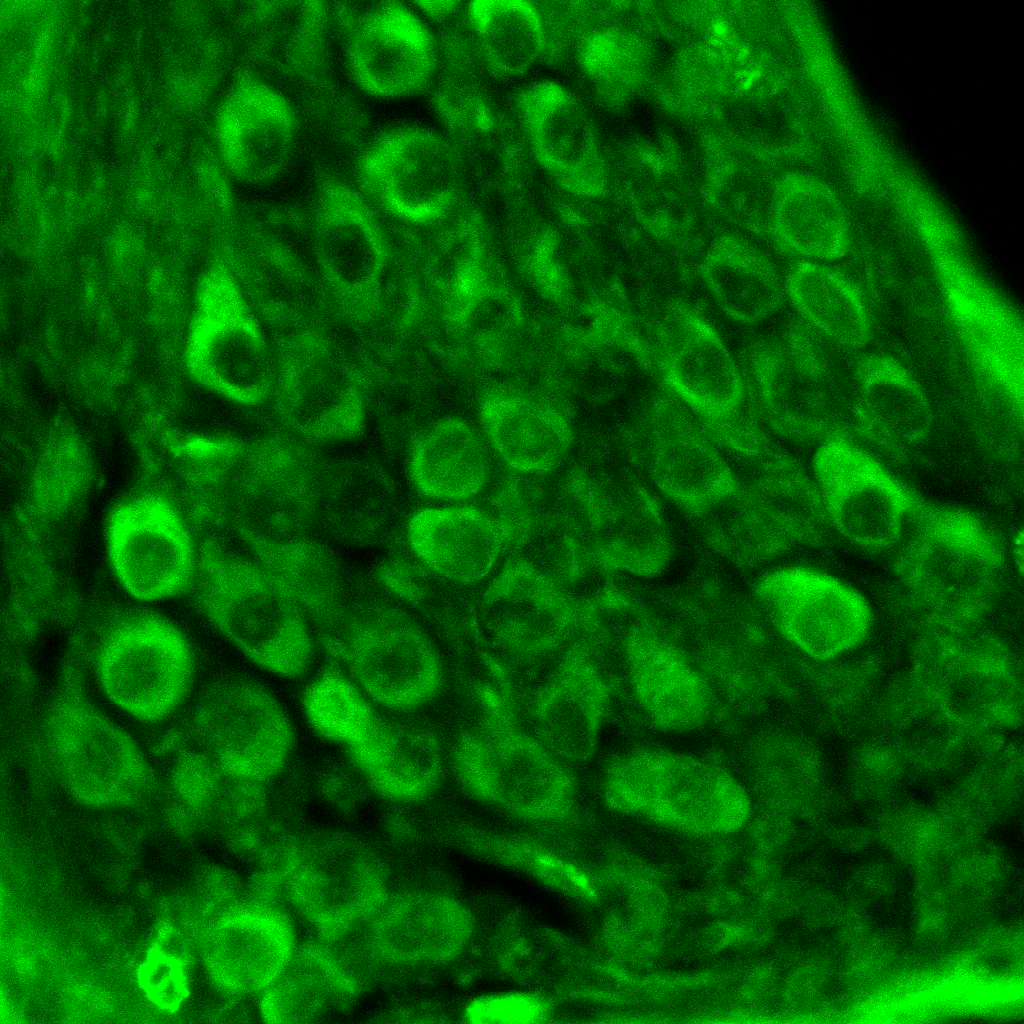

Supplement: Figure 1—source data 1. [file elife-76754-fig1-data1.zip › Figure_1_source_data/Fig.1 A/WT Base/WT Base SGN Tuj1.tif]

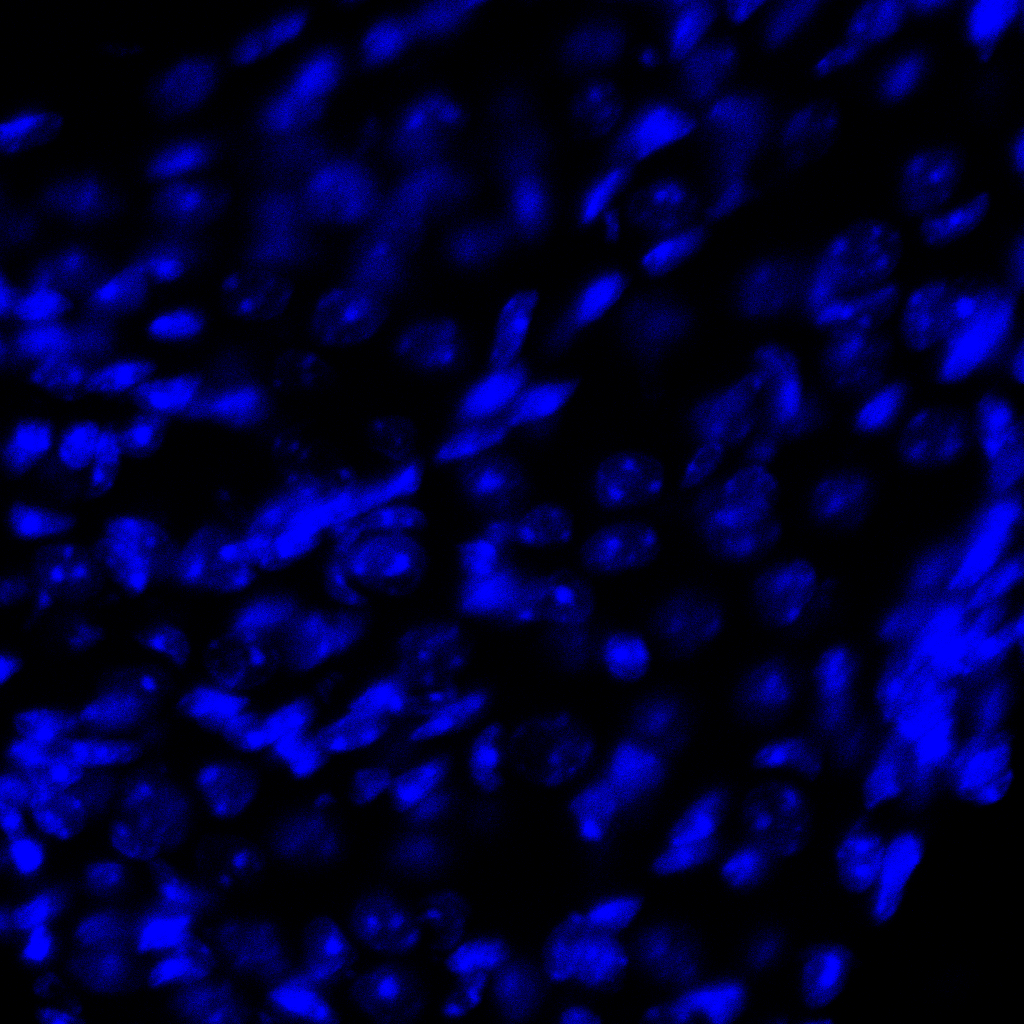

Supplement: Figure 1—source data 1. [file elife-76754-fig1-data1.zip › Figure_1_source_data/Fig.1 A/WT Middle/WT Middle SGN DAPI.tif]

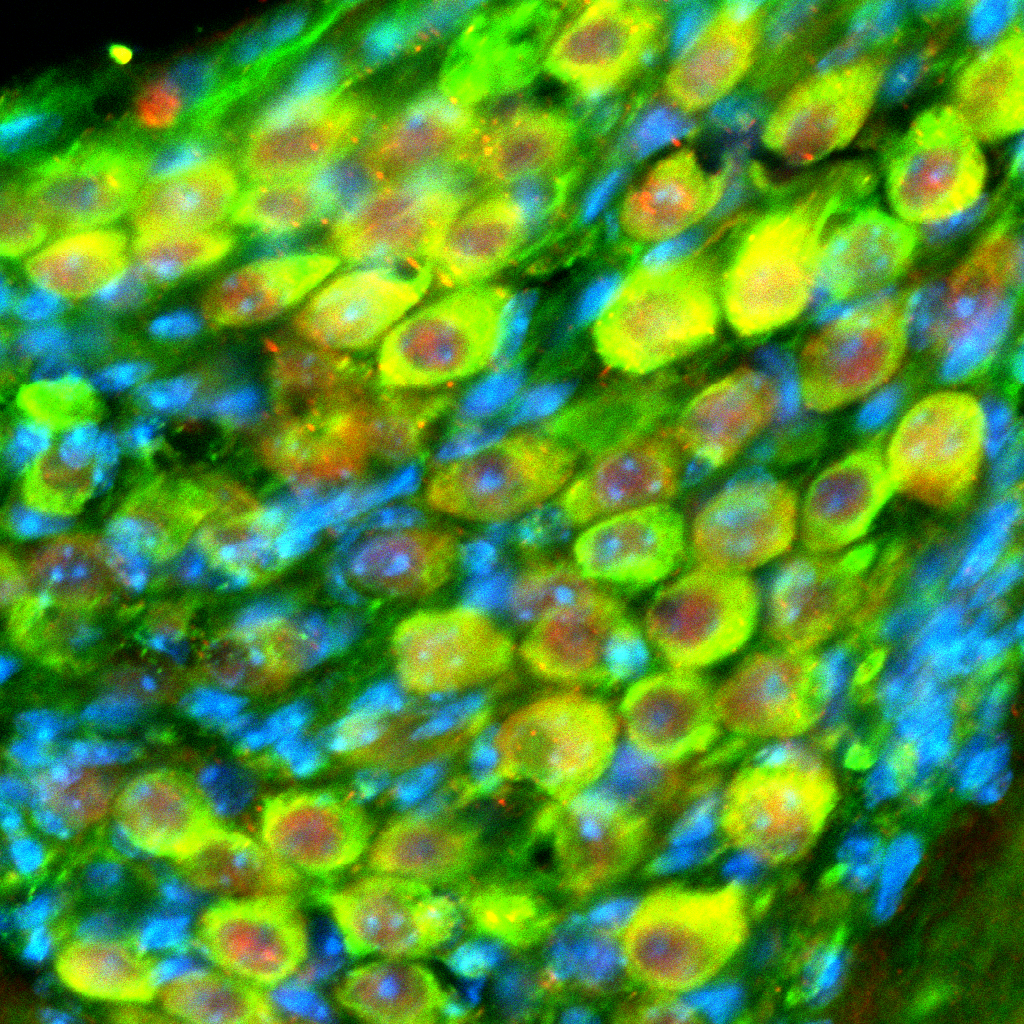

Supplement: Figure 1—source data 1. [file elife-76754-fig1-data1.zip › Figure_1_source_data/Fig.1 A/WT Middle/WT Middle SGN Merge.tif]

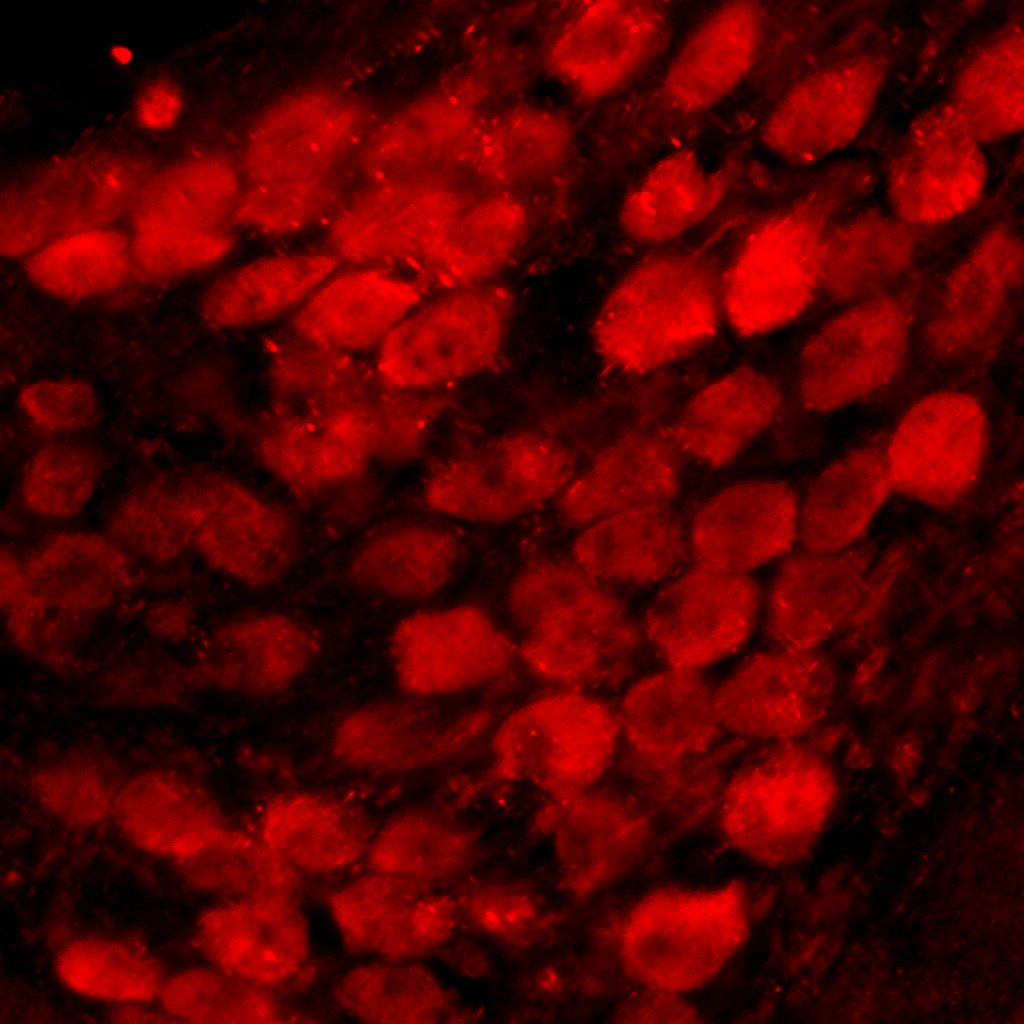

Supplement: Figure 1—source data 1. [file elife-76754-fig1-data1.zip › Figure_1_source_data/Fig.1 A/WT Middle/WT Middle SGN Rest.tif]

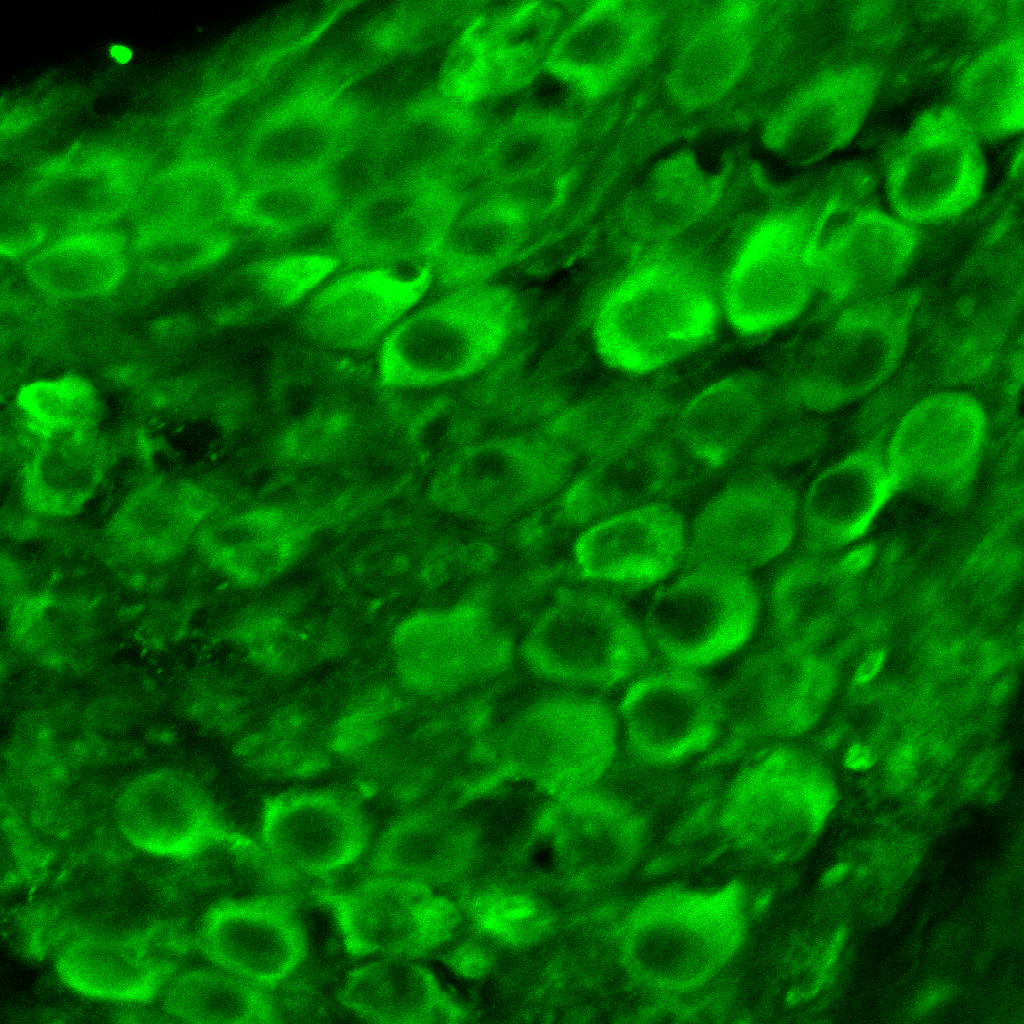

Supplement: Figure 1—source data 1. [file elife-76754-fig1-data1.zip › Figure_1_source_data/Fig.1 A/WT Middle/WT Middle SGN Tuj1.tif]

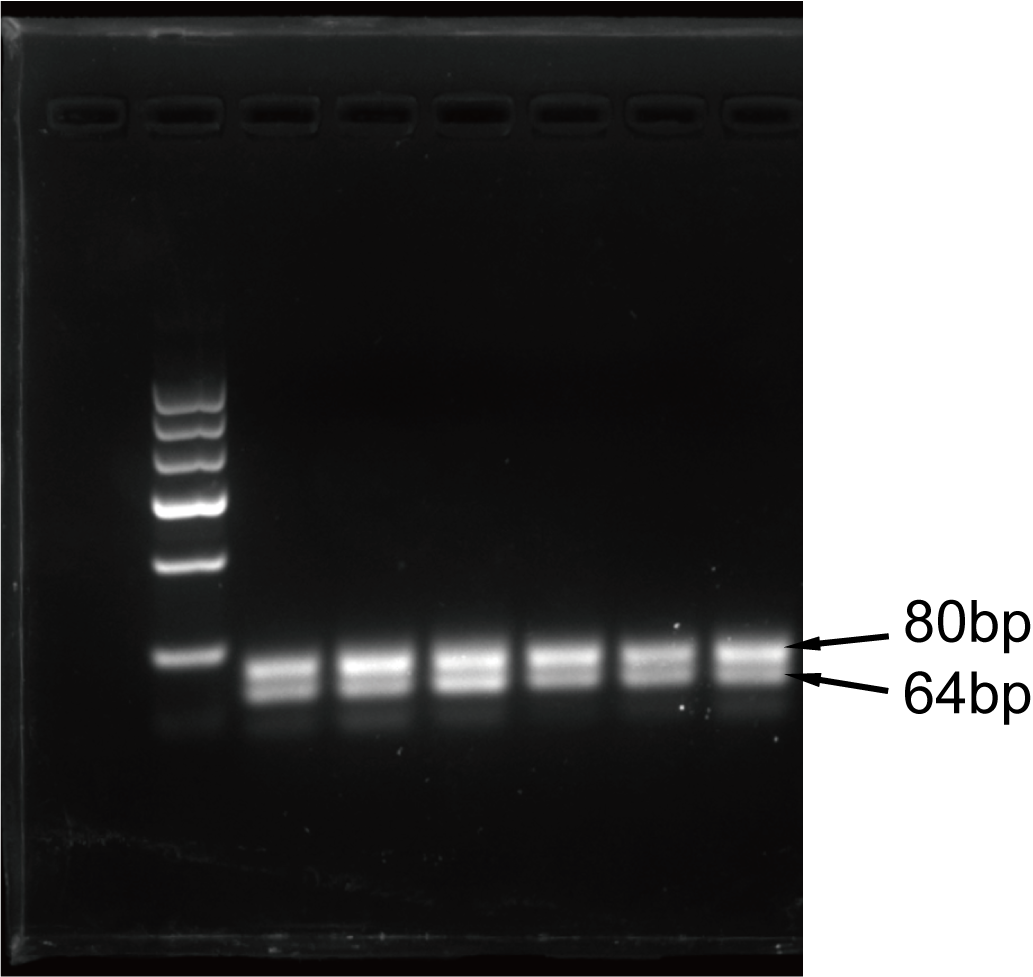

Supplement: Figure 1—source data 1. [file elife-76754-fig1-data1.zip › Figure_1_source_data/Fig.1 B/Figure_1 B.tif]

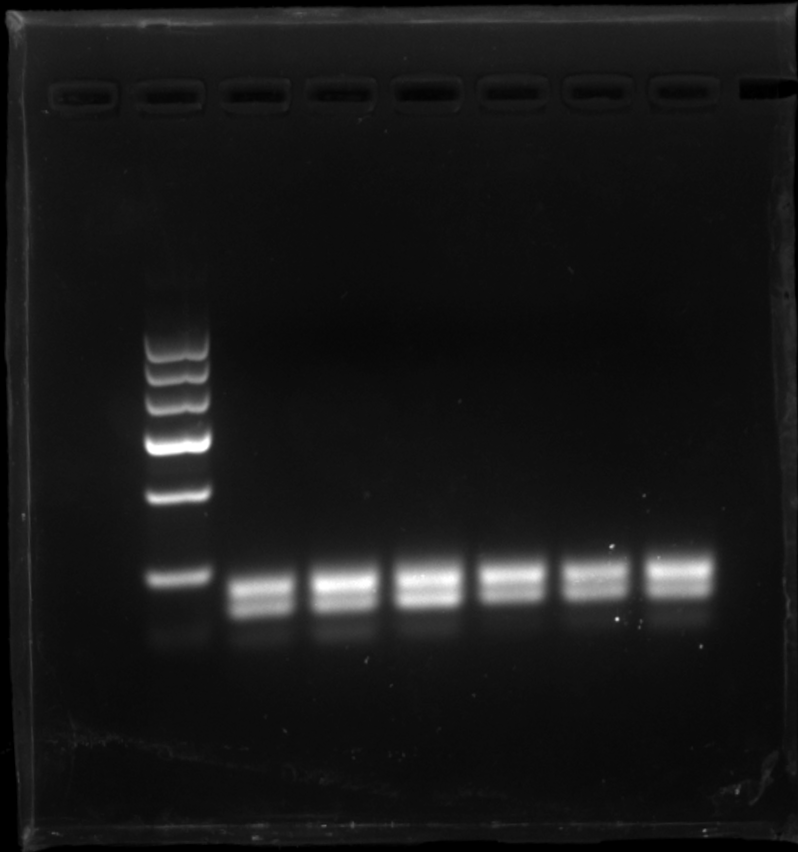

Supplement: Figure 1—source data 1. [file elife-76754-fig1-data1.zip › Figure_1_source_data/Fig.1 B/Figure_1 Bú¿originalú⌐.Tif]

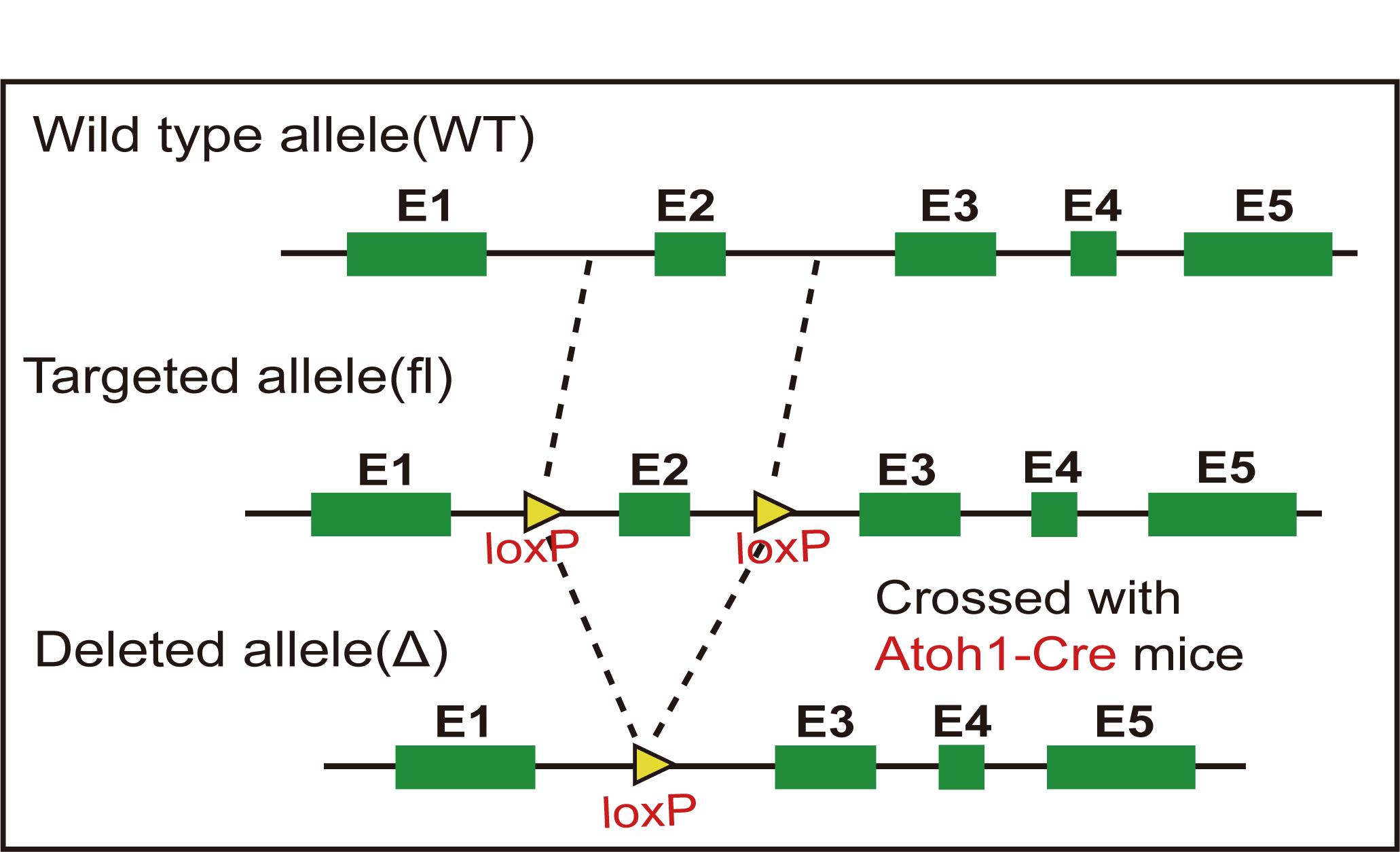

Supplement: Figure 1—source data 1. [file elife-76754-fig1-data1.zip › Figure_1_source_data/Fig.1 C/Fig.1 C.tif]

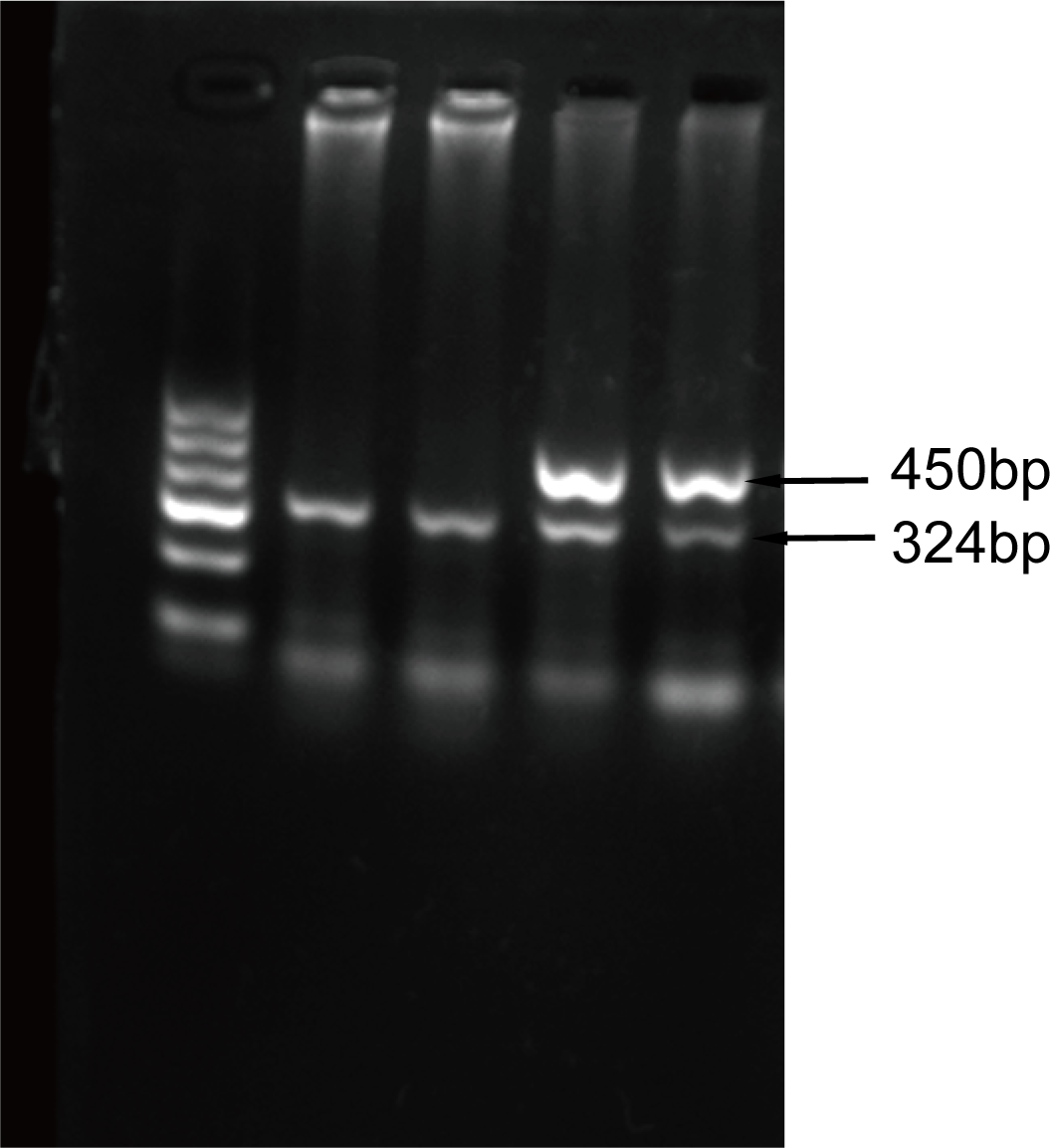

Supplement: Figure 1—source data 1. [file elife-76754-fig1-data1.zip › Figure_1_source_data/Fig.1 D/Figure_1D-Atoh1-cre.tif]

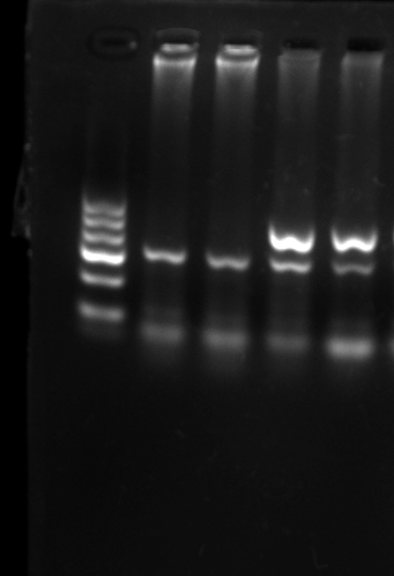

Supplement: Figure 1—source data 1. [file elife-76754-fig1-data1.zip › Figure_1_source_data/Fig.1 D/Figure_1D-Atoh1-creú¿originalú⌐.tif]

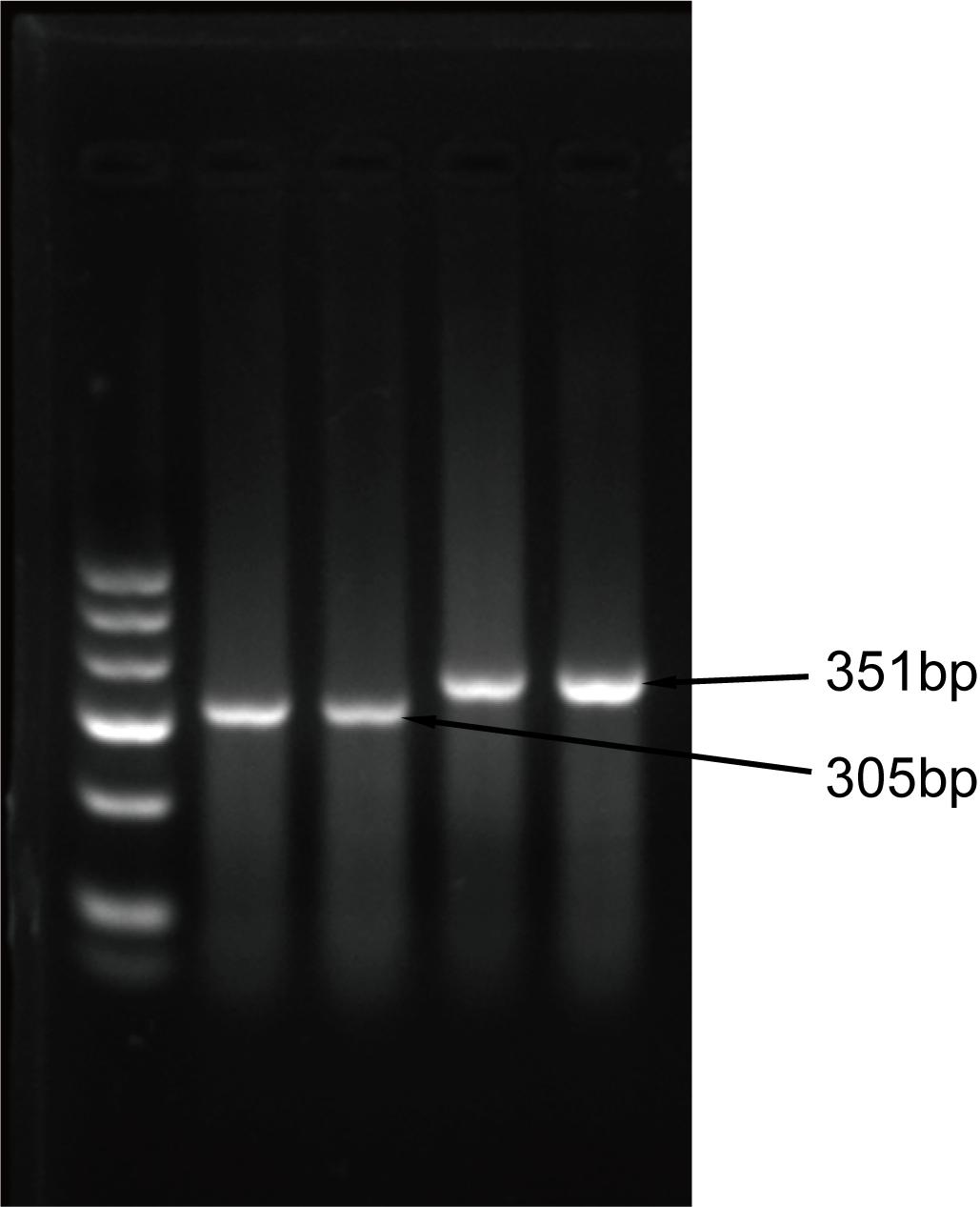

Supplement: Figure 1—source data 1. [file elife-76754-fig1-data1.zip › Figure_1_source_data/Fig.1 D/Figure_1D-Rest-loxp.tif]

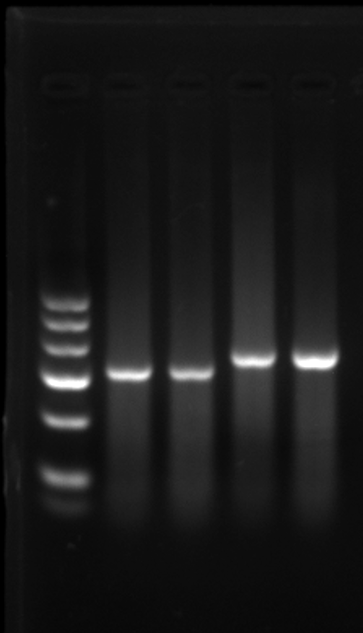

Supplement: Figure 1—source data 1. [file elife-76754-fig1-data1.zip › Figure_1_source_data/Fig.1 D/Figure_1D-Rest-loxpú¿originalú⌐.tif]

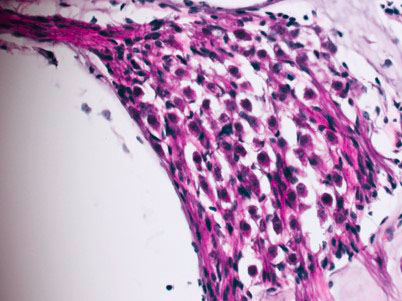

Supplement: Figure 1—source data 1. [file elife-76754-fig1-data1.zip › Figure_1_source_data/Fig.1 G/Rest +cKO Apex.tif]

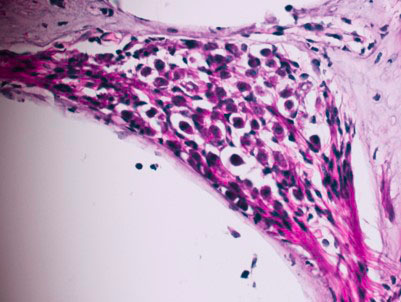

Supplement: Figure 1—source data 1. [file elife-76754-fig1-data1.zip › Figure_1_source_data/Fig.1 G/Rest +cKO Base.tif]

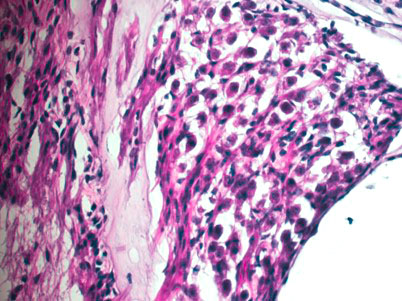

Supplement: Figure 1—source data 1. [file elife-76754-fig1-data1.zip › Figure_1_source_data/Fig.1 G/Rest +cKO Middle.tif]

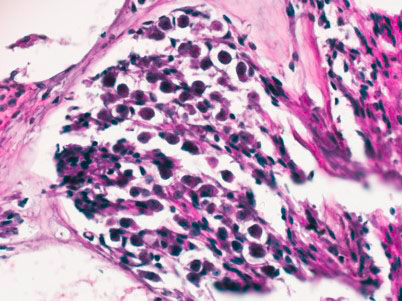

Supplement: Figure 1—source data 1. [file elife-76754-fig1-data1.zip › Figure_1_source_data/Fig.1 G/Rest cKO Apex.tif]

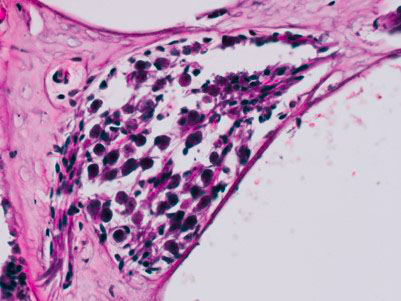

Supplement: Figure 1—source data 1. [file elife-76754-fig1-data1.zip › Figure_1_source_data/Fig.1 G/Rest cKO Base.tif]

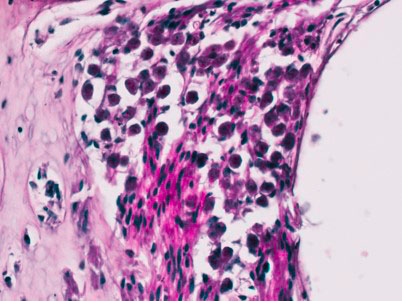

Supplement: Figure 1—source data 1. [file elife-76754-fig1-data1.zip › Figure_1_source_data/Fig.1 G/Rest cKO Middle.tif]

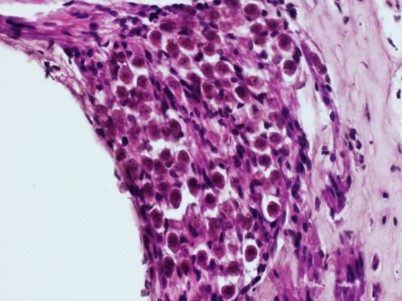

Supplement: Figure 1—source data 1. [file elife-76754-fig1-data1.zip › Figure_1_source_data/Fig.1 G/WT Apex.tif]

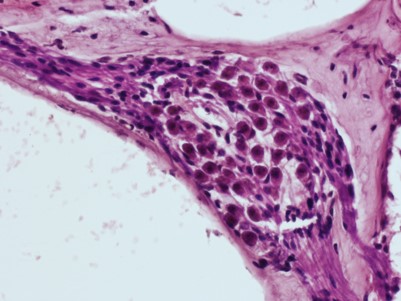

Supplement: Figure 1—source data 1. [file elife-76754-fig1-data1.zip › Figure_1_source_data/Fig.1 G/WT Base.tif]

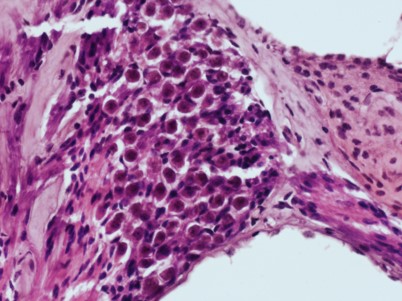

Supplement: Figure 1—source data 1. [file elife-76754-fig1-data1.zip › Figure_1_source_data/Fig.1 G/WT Middle.tif]

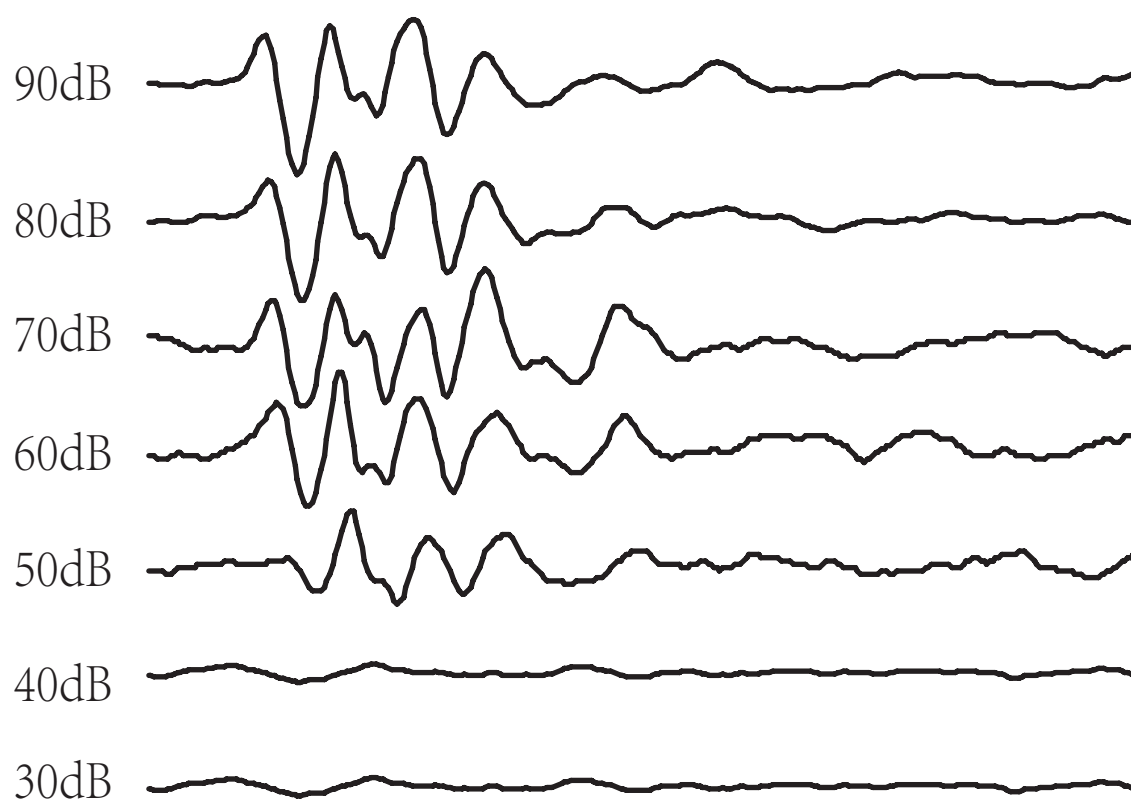

Supplement: Figure 1—source data 1. [file elife-76754-fig1-data1.zip › Figure_1_source_data/Fig.1 H/Rest cKO 1M.pdf]

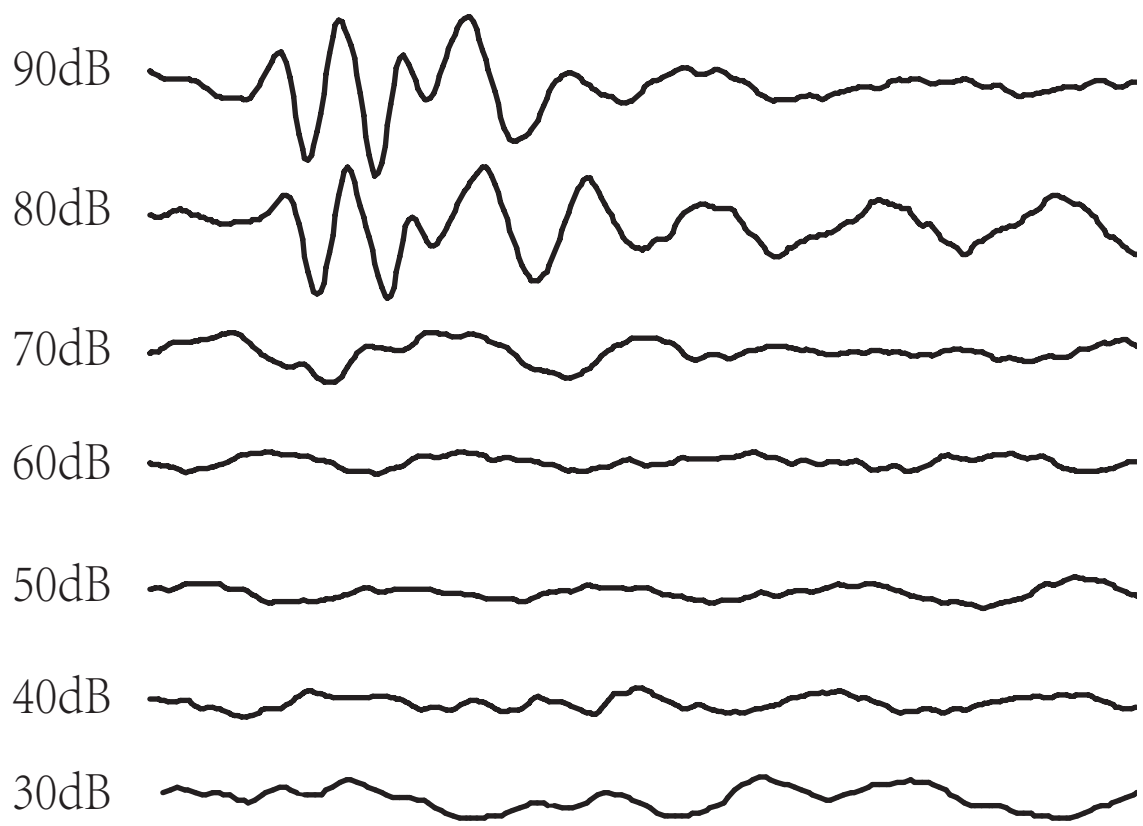

Supplement: Figure 1—source data 1. [file elife-76754-fig1-data1.zip › Figure_1_source_data/Fig.1 H/Rest cKO 3M.pdf]

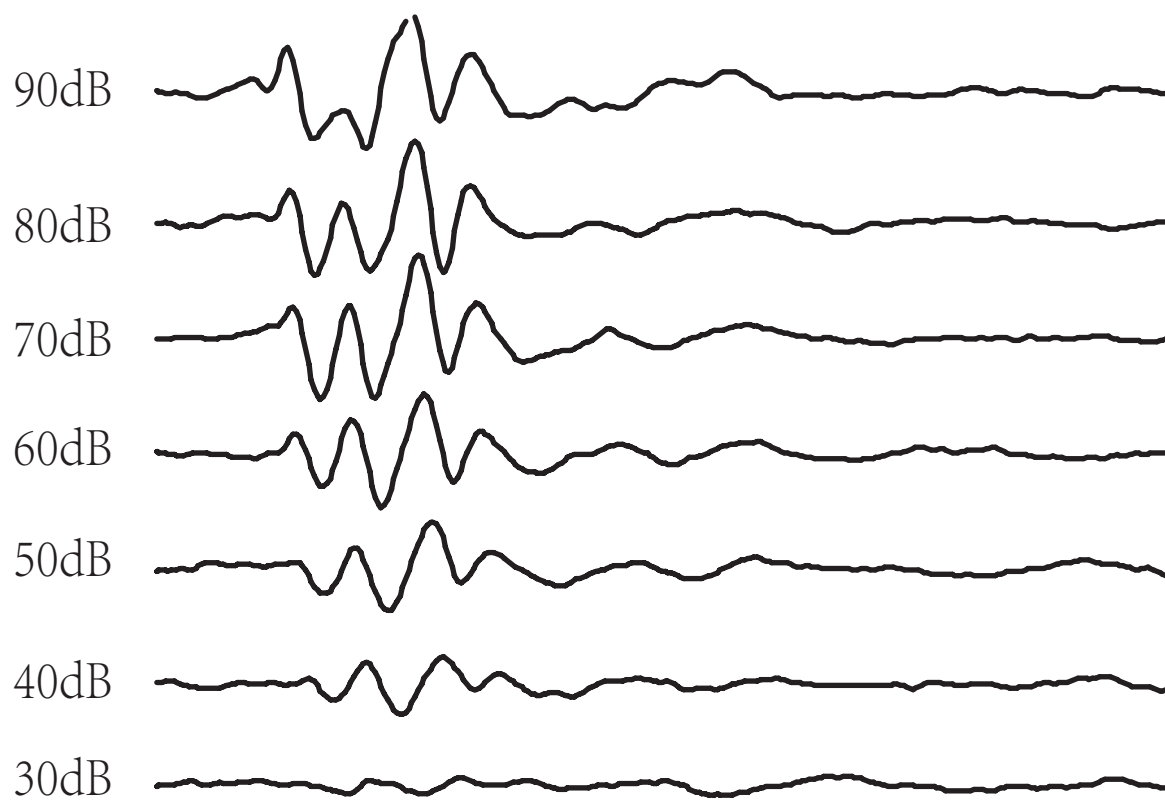

Supplement: Figure 1—source data 1. [file elife-76754-fig1-data1.zip › Figure_1_source_data/Fig.1 H/WT 1M.pdf]

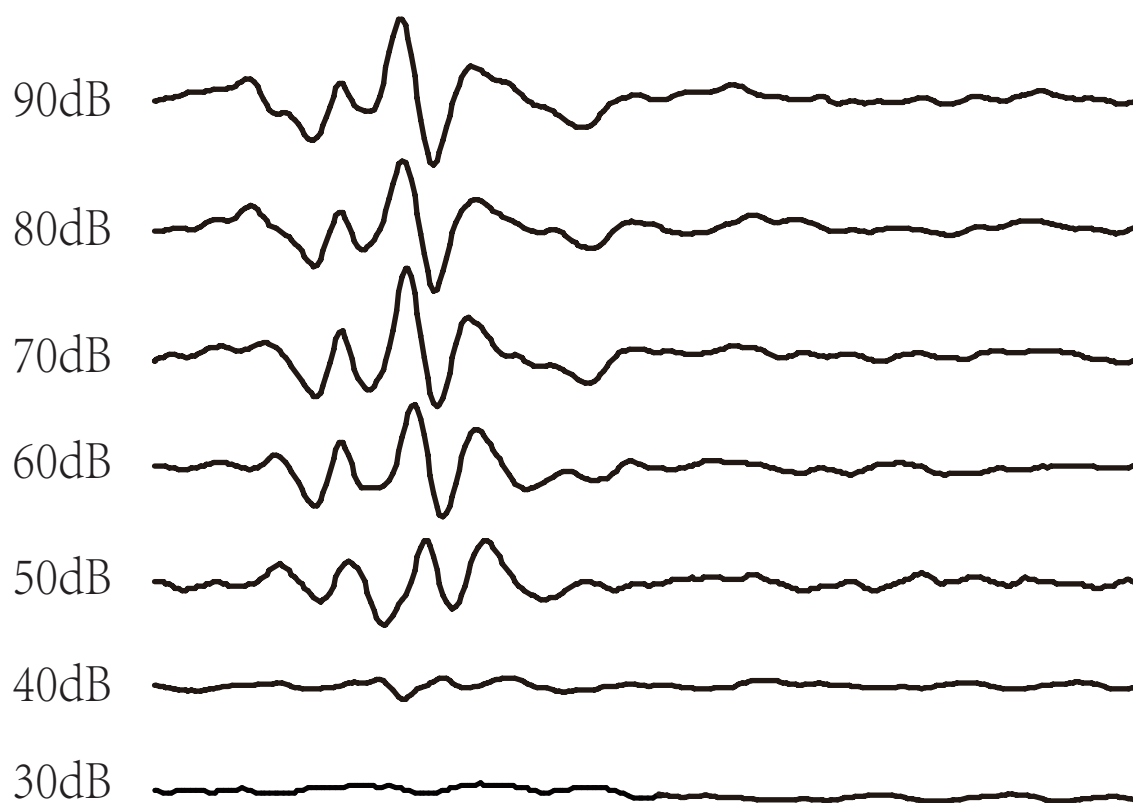

Supplement: Figure 1—source data 1. [file elife-76754-fig1-data1.zip › Figure_1_source_data/Fig.1 H/WT 3M.pdf]

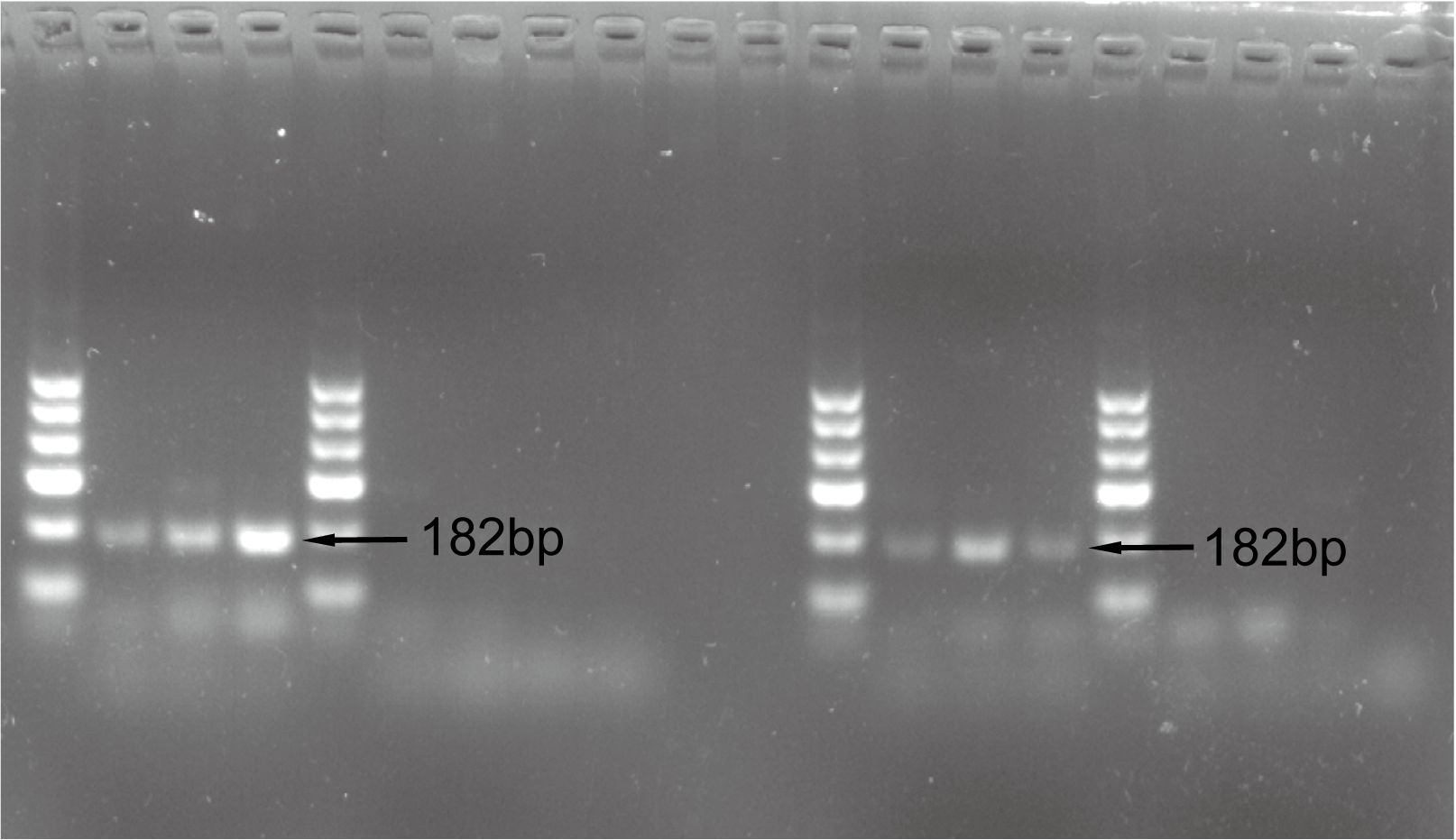

Supplement: Figure 1—figure supplement 1—source data 1. [file elife-76754-fig1-figsupp1-data1.zip › Figure 1-figure supplement 1-source data/Figure 1 - Figure supplement 1.tif]

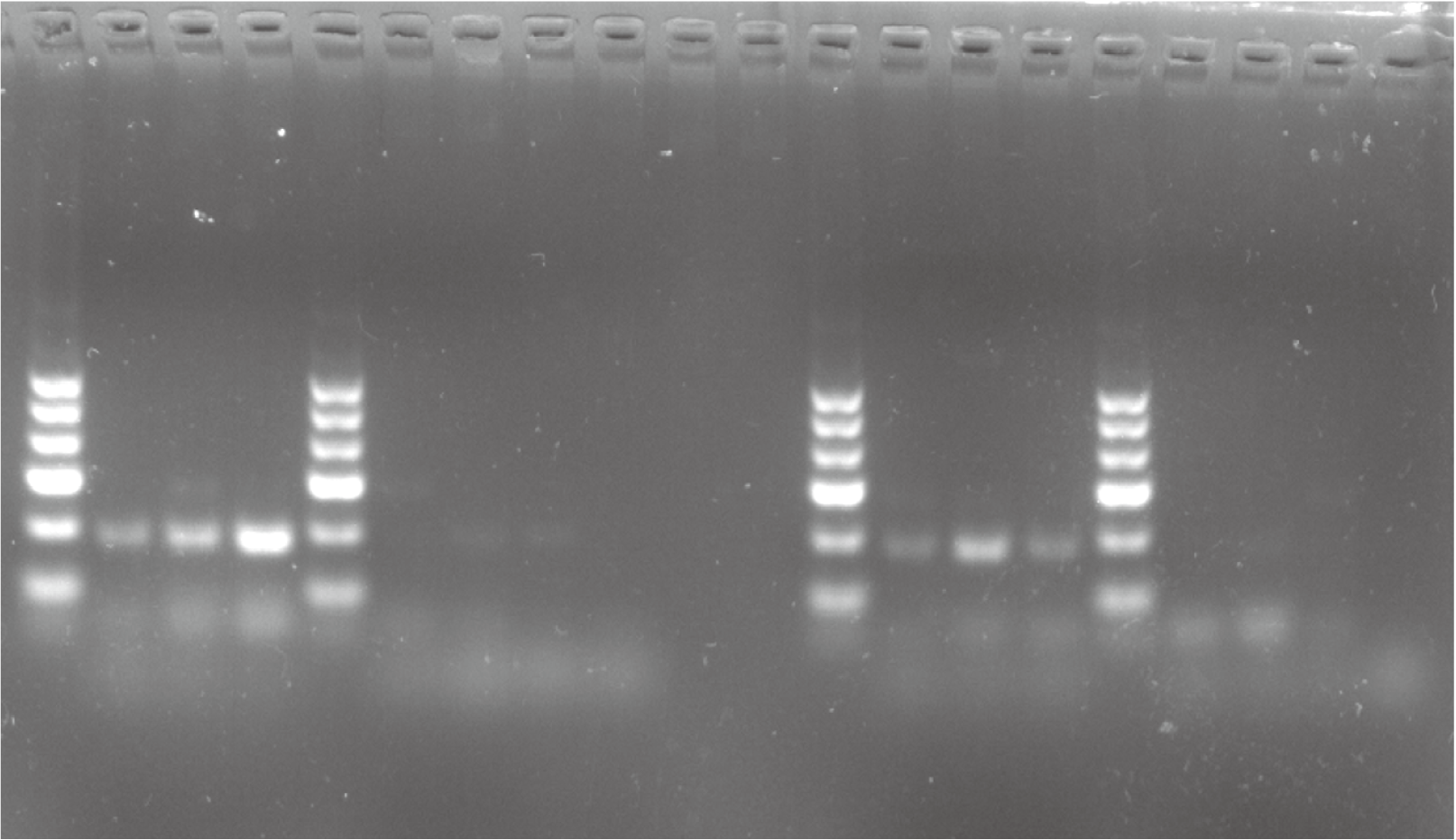

Supplement: Figure 1—figure supplement 1—source data 1. [file elife-76754-fig1-figsupp1-data1.zip › Figure 1-figure supplement 1-source data/Figure 1 - Figure supplement 1ú¿originalú⌐.tif]

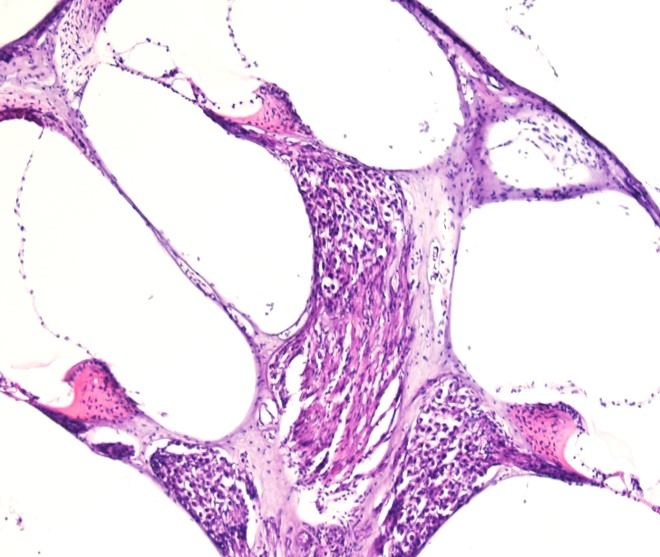

Supplement: Figure 2—source data 1. [file elife-76754-fig2-data1.zip › Figure_2_source_data/Fig.2 A/Fig.2 A.tif]

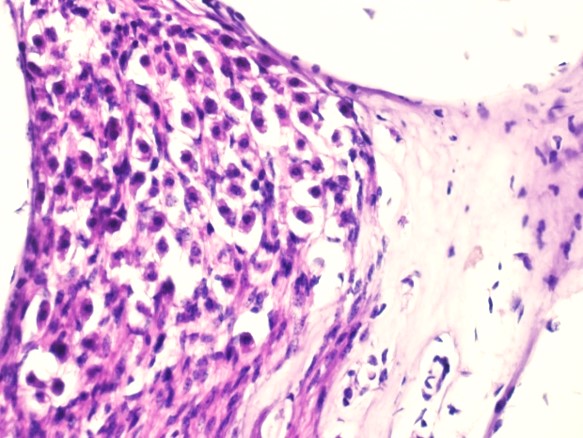

Supplement: Figure 2—source data 1. [file elife-76754-fig2-data1.zip › Figure_2_source_data/Fig.2 B/Rest cKO 1M Apex.tif]

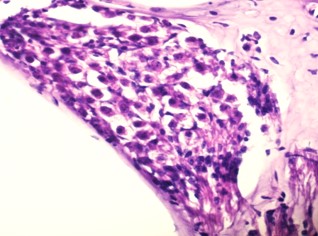

Supplement: Figure 2—source data 1. [file elife-76754-fig2-data1.zip › Figure_2_source_data/Fig.2 B/Rest cKO 1M Base.tif]

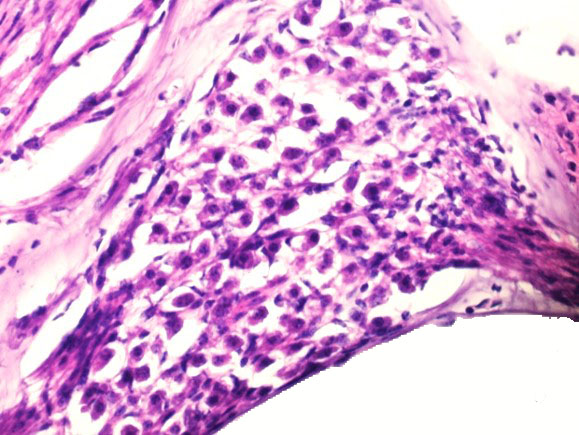

Supplement: Figure 2—source data 1. [file elife-76754-fig2-data1.zip › Figure_2_source_data/Fig.2 B/Rest cKO 1M Middle.tif]

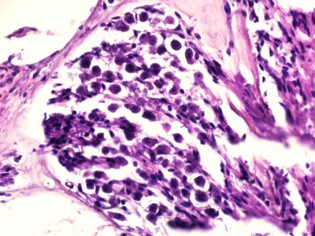

Supplement: Figure 2—source data 1. [file elife-76754-fig2-data1.zip › Figure_2_source_data/Fig.2 B/Rest cKO 3M Apex.tif]

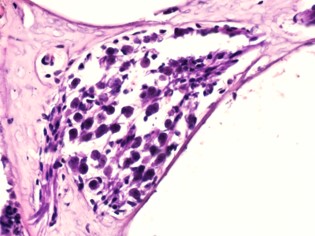

Supplement: Figure 2—source data 1. [file elife-76754-fig2-data1.zip › Figure_2_source_data/Fig.2 B/Rest cKO 3M Base.tif]

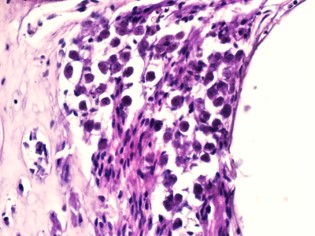

Supplement: Figure 2—source data 1. [file elife-76754-fig2-data1.zip › Figure_2_source_data/Fig.2 B/Rest cKO 3M Middle.tif]

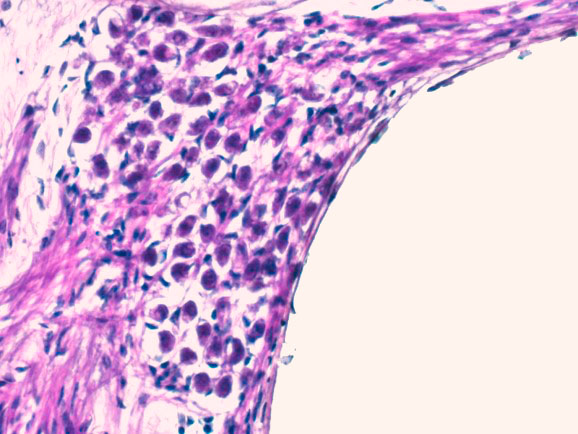

Supplement: Figure 2—source data 1. [file elife-76754-fig2-data1.zip › Figure_2_source_data/Fig.2 B/WT 1M Apex.tif]

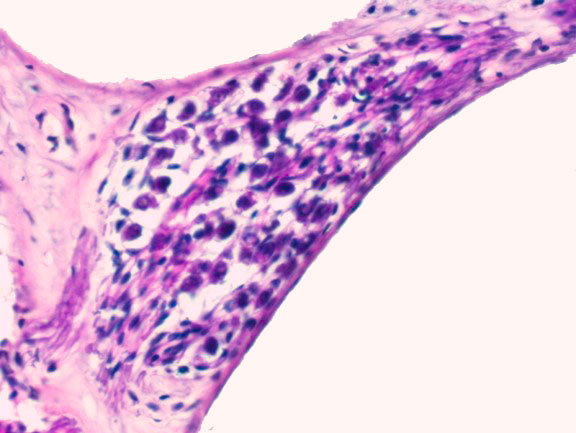

Supplement: Figure 2—source data 1. [file elife-76754-fig2-data1.zip › Figure_2_source_data/Fig.2 B/WT 1M Base.tif]

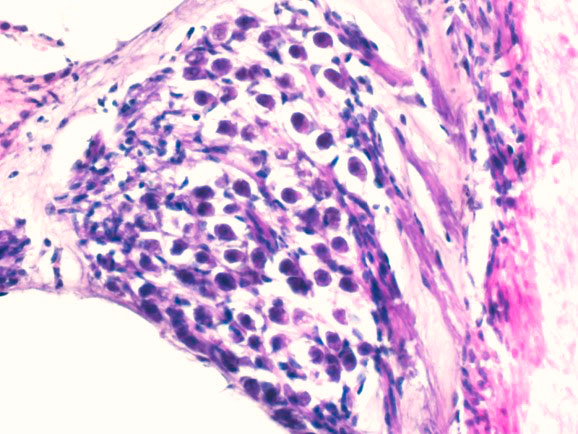

Supplement: Figure 2—source data 1. [file elife-76754-fig2-data1.zip › Figure_2_source_data/Fig.2 B/WT 1M Middle.tif]

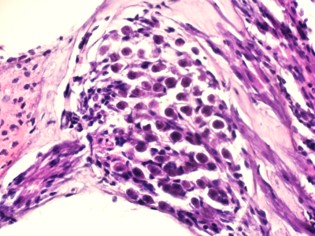

Supplement: Figure 2—source data 1. [file elife-76754-fig2-data1.zip › Figure_2_source_data/Fig.2 B/WT 3M Apex.tif]

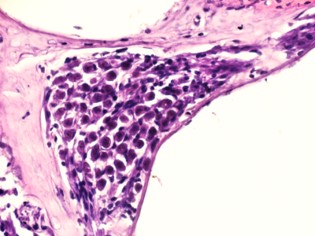

Supplement: Figure 2—source data 1. [file elife-76754-fig2-data1.zip › Figure_2_source_data/Fig.2 B/WT 3M Base.tif]

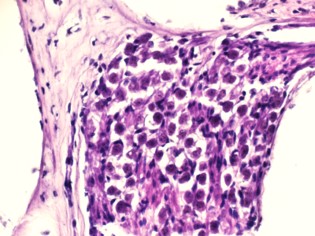

Supplement: Figure 2—source data 1. [file elife-76754-fig2-data1.zip › Figure_2_source_data/Fig.2 B/WT 3M Middle.tif]

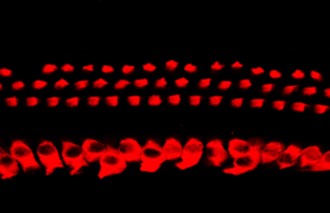

Supplement: Figure 2—source data 1. [file elife-76754-fig2-data1.zip › Figure_2_source_data/Fig.2 F/Rest cKO 1M Apex.tif]

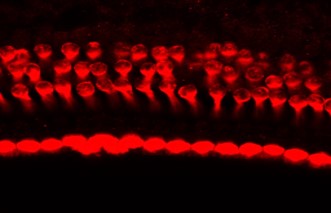

Supplement: Figure 2—source data 1. [file elife-76754-fig2-data1.zip › Figure_2_source_data/Fig.2 F/Rest cKO 1M Base.tif]

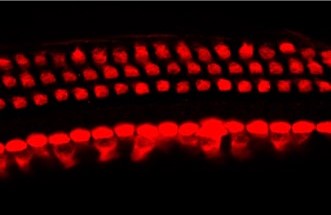

Supplement: Figure 2—source data 1. [file elife-76754-fig2-data1.zip › Figure_2_source_data/Fig.2 F/Rest cKO 1M Middle.tif]

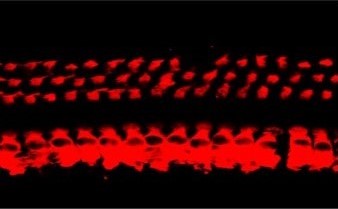

Supplement: Figure 2—source data 1. [file elife-76754-fig2-data1.zip › Figure_2_source_data/Fig.2 F/Rest cKO 3M Apex.tif]

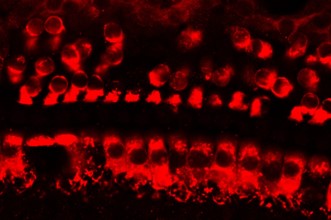

Supplement: Figure 2—source data 1. [file elife-76754-fig2-data1.zip › Figure_2_source_data/Fig.2 F/Rest cKO 3M Base.tif]

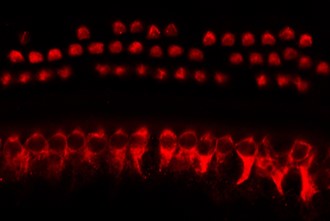

Supplement: Figure 2—source data 1. [file elife-76754-fig2-data1.zip › Figure_2_source_data/Fig.2 F/Rest cKO 3M Middle.tif]

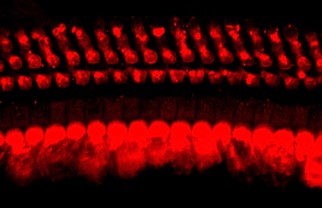

Supplement: Figure 2—source data 1. [file elife-76754-fig2-data1.zip › Figure_2_source_data/Fig.2 F/WT 1M Apex.tif]

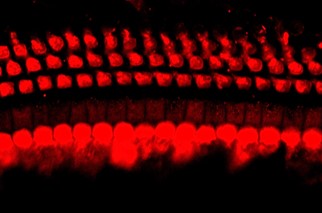

Supplement: Figure 2—source data 1. [file elife-76754-fig2-data1.zip › Figure_2_source_data/Fig.2 F/WT 1M Base.tif]

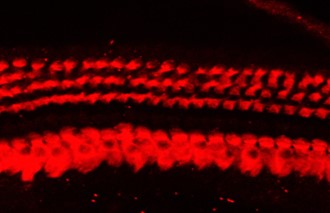

Supplement: Figure 2—source data 1. [file elife-76754-fig2-data1.zip › Figure_2_source_data/Fig.2 F/WT 1M Middle.tif]

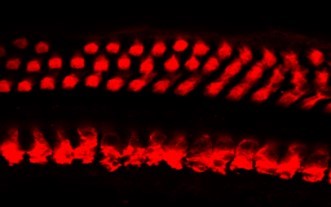

Supplement: Figure 2—source data 1. [file elife-76754-fig2-data1.zip › Figure_2_source_data/Fig.2 F/WT 3M Apex.tif]

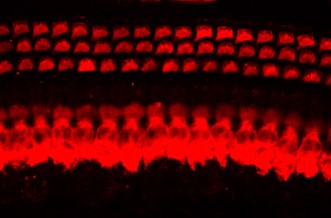

Supplement: Figure 2—source data 1. [file elife-76754-fig2-data1.zip › Figure_2_source_data/Fig.2 F/WT 3M Base.tif]

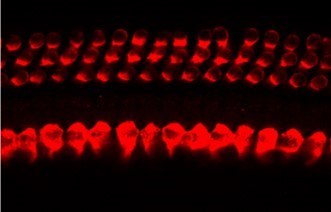

Supplement: Figure 2—source data 1. [file elife-76754-fig2-data1.zip › Figure_2_source_data/Fig.2 F/WT 3M Middle.tif]

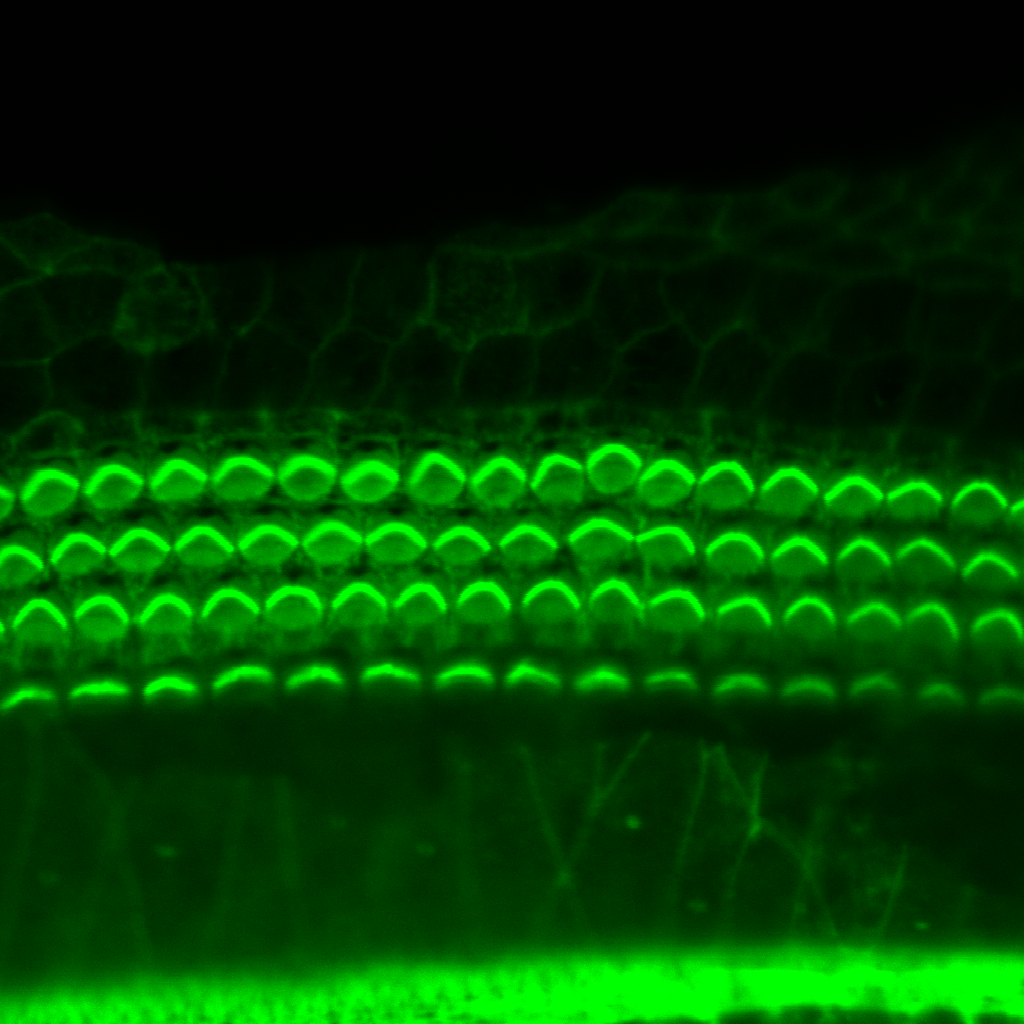

Supplement: Figure 2—figure supplement 1—source data 1. [file elife-76754-fig2-figsupp1-data1.zip › Figure 2 - figure supplement 1 Source data/Figure 2 - figure supplement 1 A/Rest-cko P1 apex.tif]

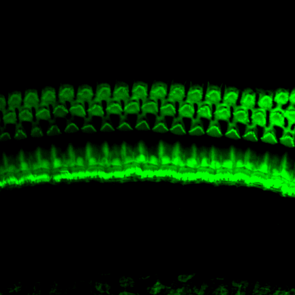

Supplement: Figure 2—figure supplement 1—source data 1. [file elife-76754-fig2-figsupp1-data1.zip › Figure 2 - figure supplement 1 Source data/Figure 2 - figure supplement 1 A/Rest-cko P14 apex.tif]

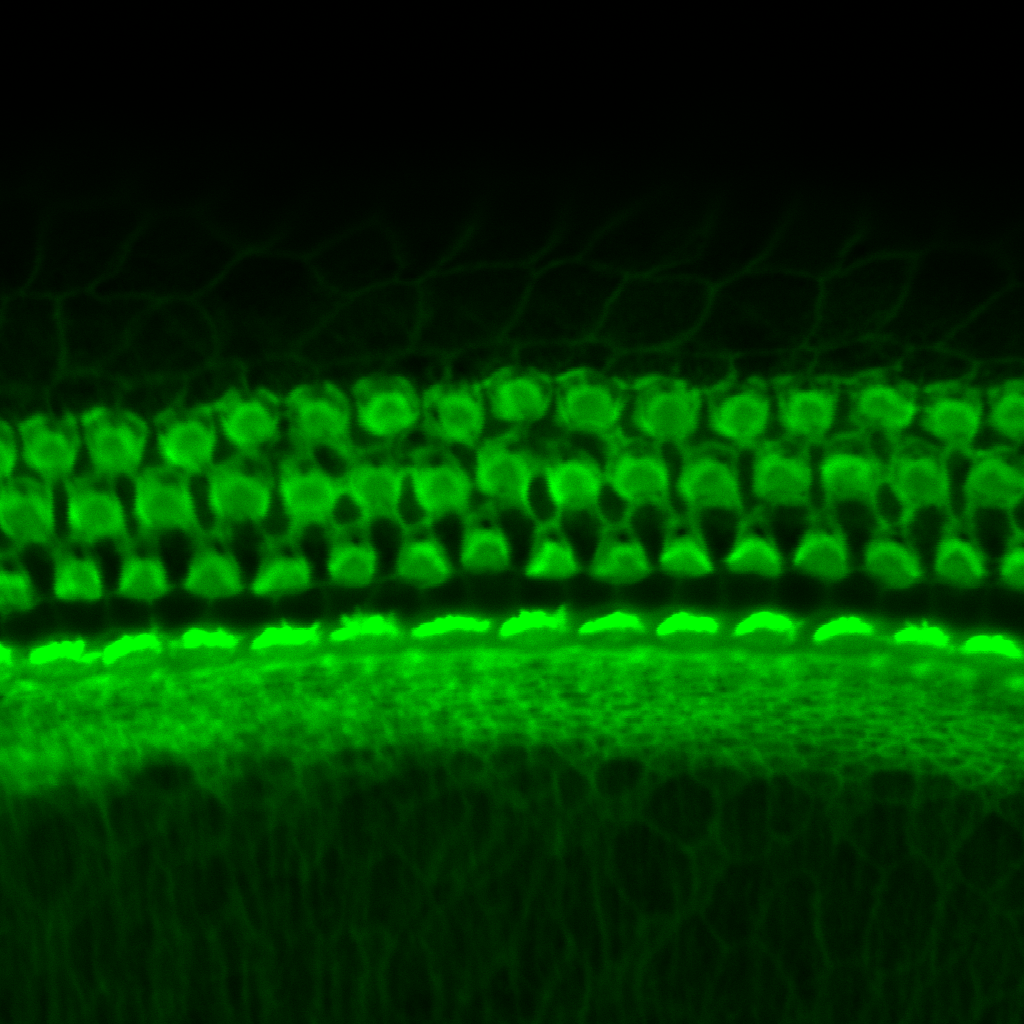

Supplement: Figure 2—figure supplement 1—source data 1. [file elife-76754-fig2-figsupp1-data1.zip › Figure 2 - figure supplement 1 Source data/Figure 2 - figure supplement 1 A/Rest-cko P7 apex.tif]

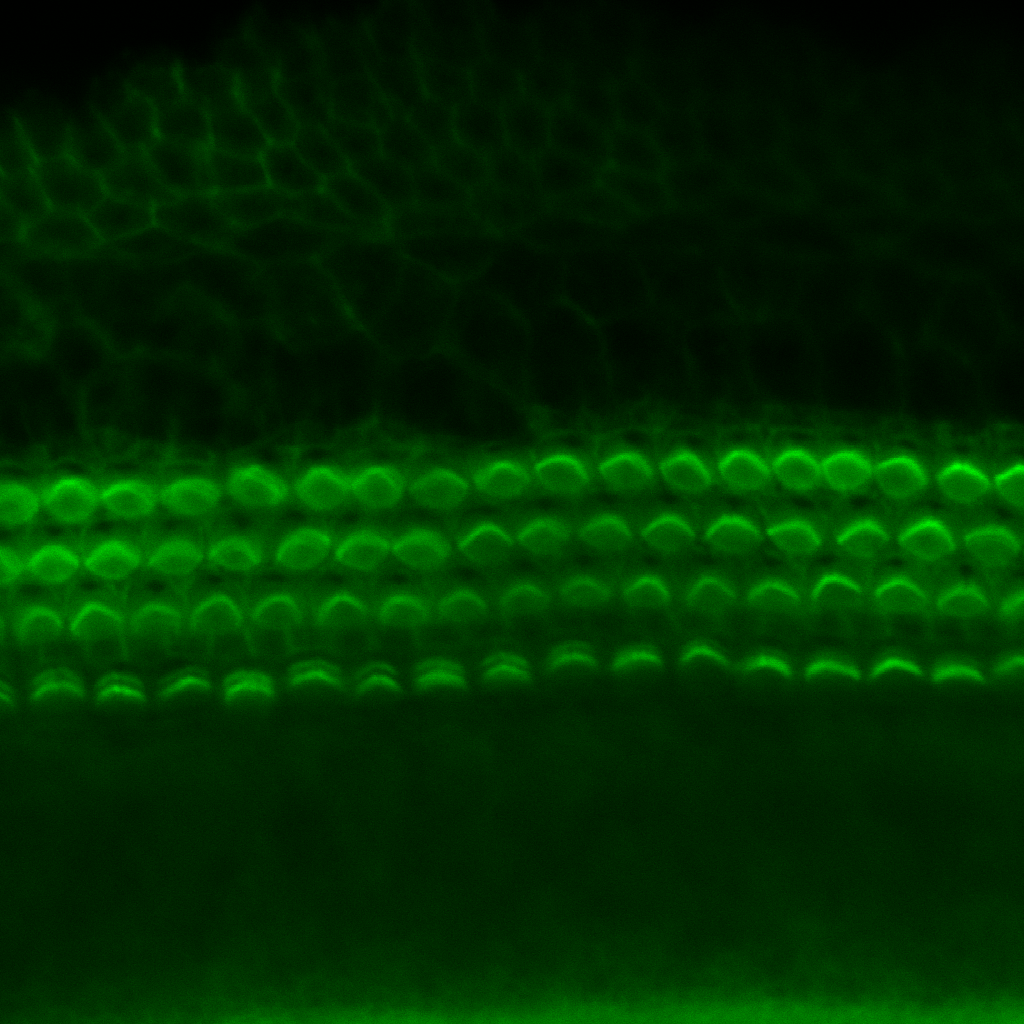

Supplement: Figure 2—figure supplement 1—source data 1. [file elife-76754-fig2-figsupp1-data1.zip › Figure 2 - figure supplement 1 Source data/Figure 2 - figure supplement 1 A/WT P1 apex.tif]

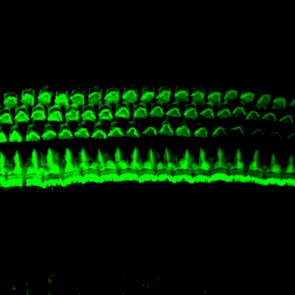

Supplement: Figure 2—figure supplement 1—source data 1. [file elife-76754-fig2-figsupp1-data1.zip › Figure 2 - figure supplement 1 Source data/Figure 2 - figure supplement 1 A/WT P14 apex.tif]

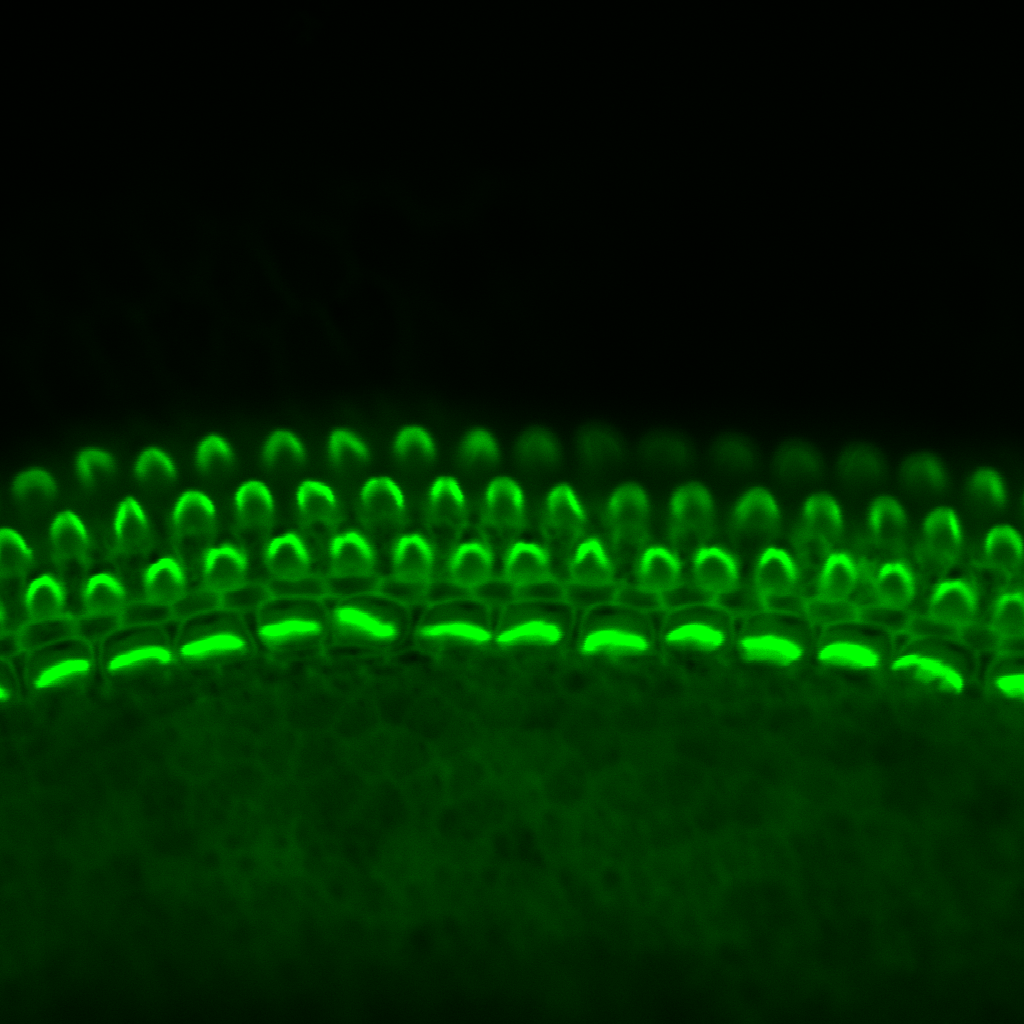

Supplement: Figure 2—figure supplement 1—source data 1. [file elife-76754-fig2-figsupp1-data1.zip › Figure 2 - figure supplement 1 Source data/Figure 2 - figure supplement 1 A/WT P7 apex.tif]

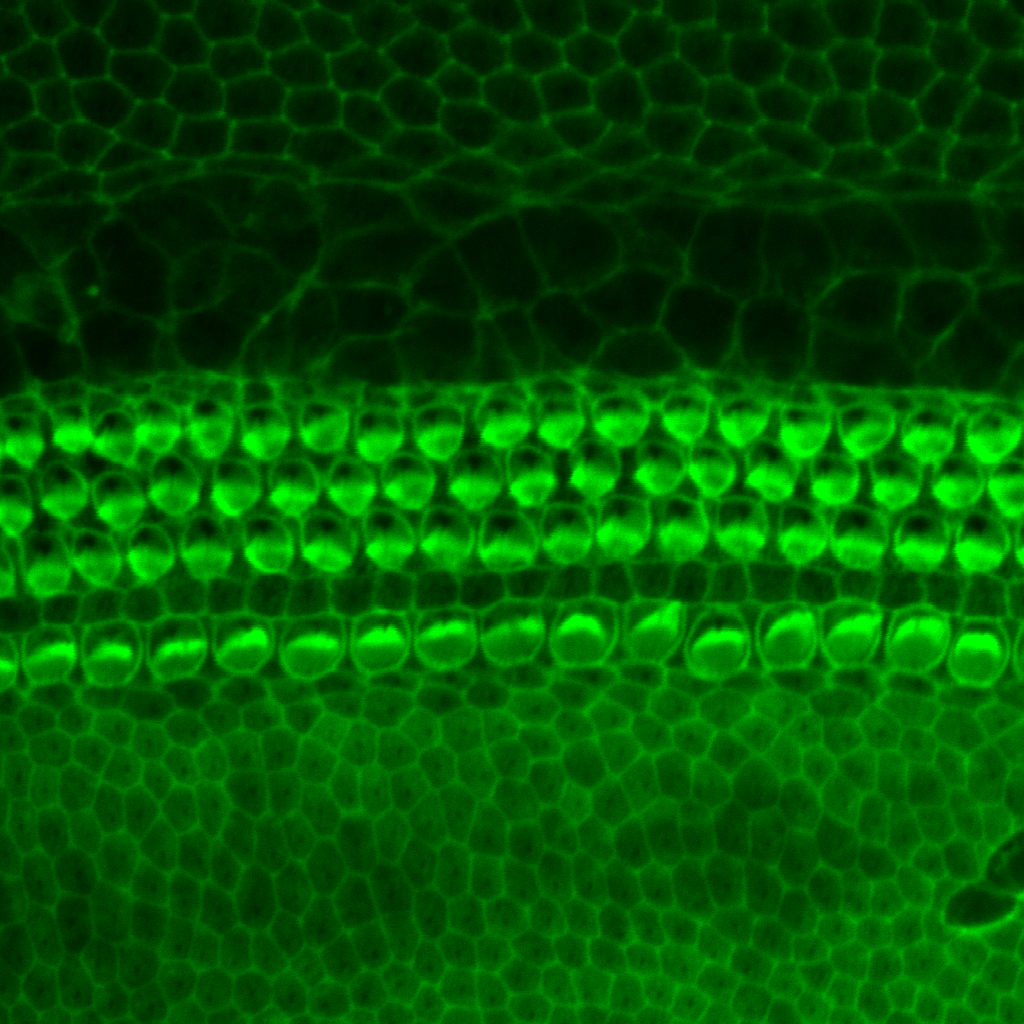

Supplement: Figure 2—figure supplement 1—source data 1. [file elife-76754-fig2-figsupp1-data1.zip › Figure 2 - figure supplement 1 Source data/Figure 2 - figure supplement 1 B/Rest-cko P1 middle.tif]

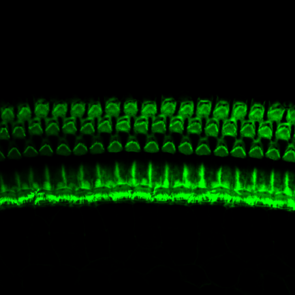

Supplement: Figure 2—figure supplement 1—source data 1. [file elife-76754-fig2-figsupp1-data1.zip › Figure 2 - figure supplement 1 Source data/Figure 2 - figure supplement 1 B/Rest-cko P14 middle.tif]

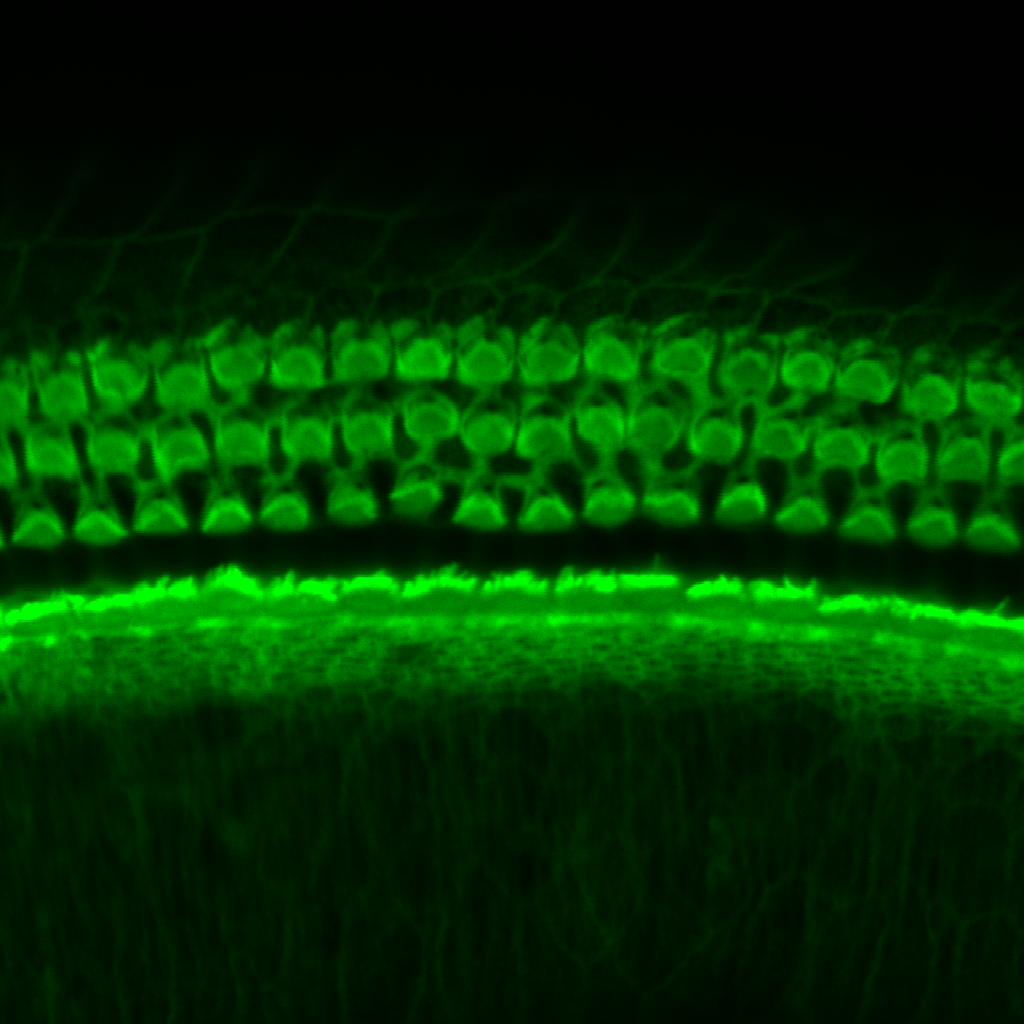

Supplement: Figure 2—figure supplement 1—source data 1. [file elife-76754-fig2-figsupp1-data1.zip › Figure 2 - figure supplement 1 Source data/Figure 2 - figure supplement 1 B/Rest-cko P7 middle.tif]

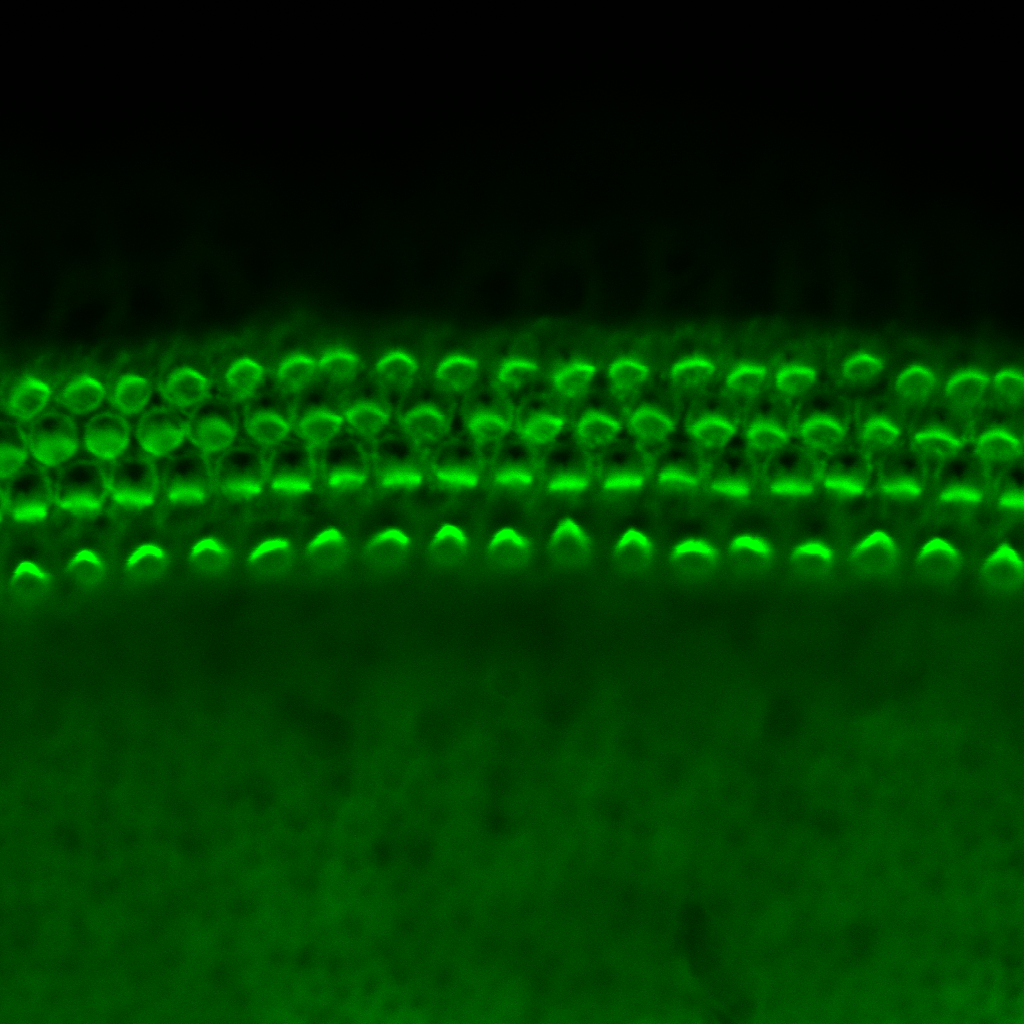

Supplement: Figure 2—figure supplement 1—source data 1. [file elife-76754-fig2-figsupp1-data1.zip › Figure 2 - figure supplement 1 Source data/Figure 2 - figure supplement 1 B/WT P1 middle.tif]

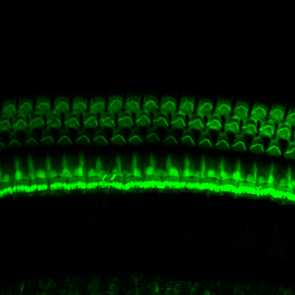

Supplement: Figure 2—figure supplement 1—source data 1. [file elife-76754-fig2-figsupp1-data1.zip › Figure 2 - figure supplement 1 Source data/Figure 2 - figure supplement 1 B/WT P14 middle.tif]

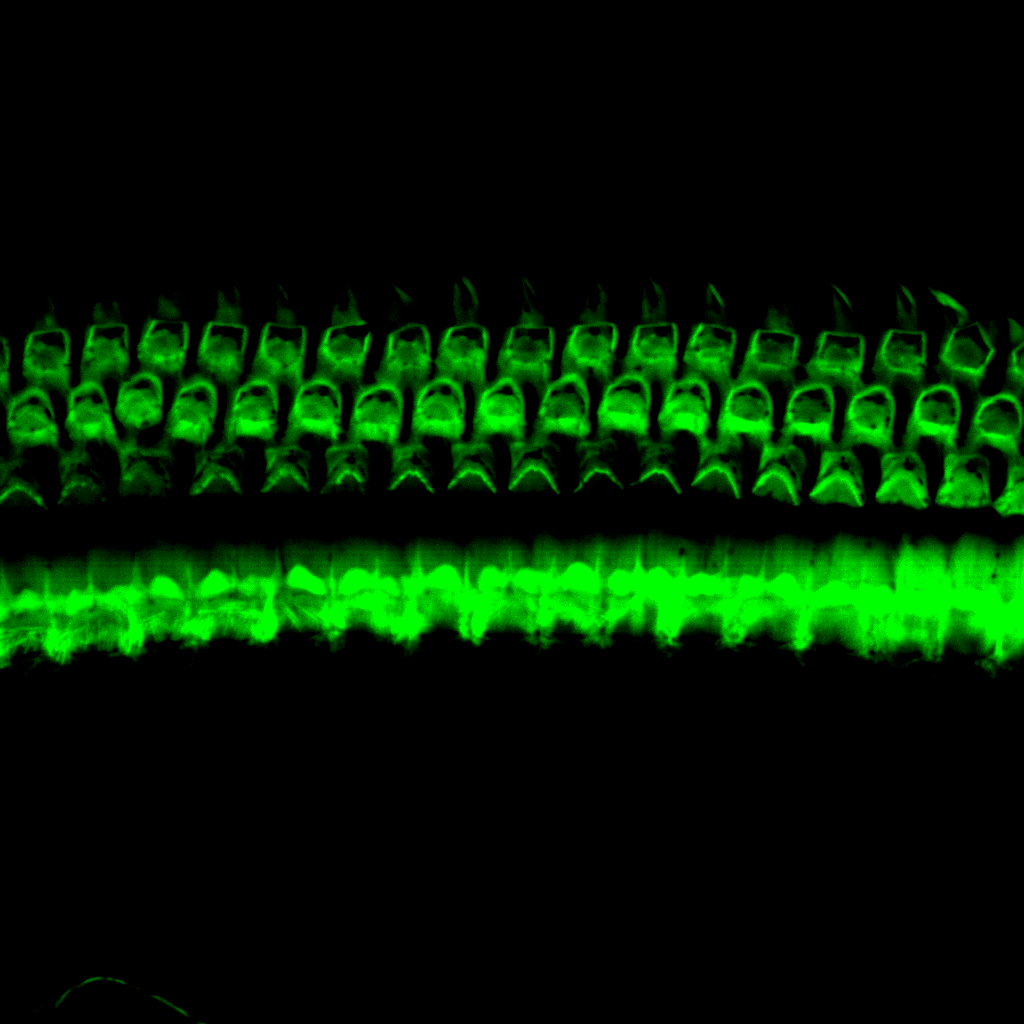

Supplement: Figure 2—figure supplement 1—source data 1. [file elife-76754-fig2-figsupp1-data1.zip › Figure 2 - figure supplement 1 Source data/Figure 2 - figure supplement 1 B/WT P7 middle.tif]

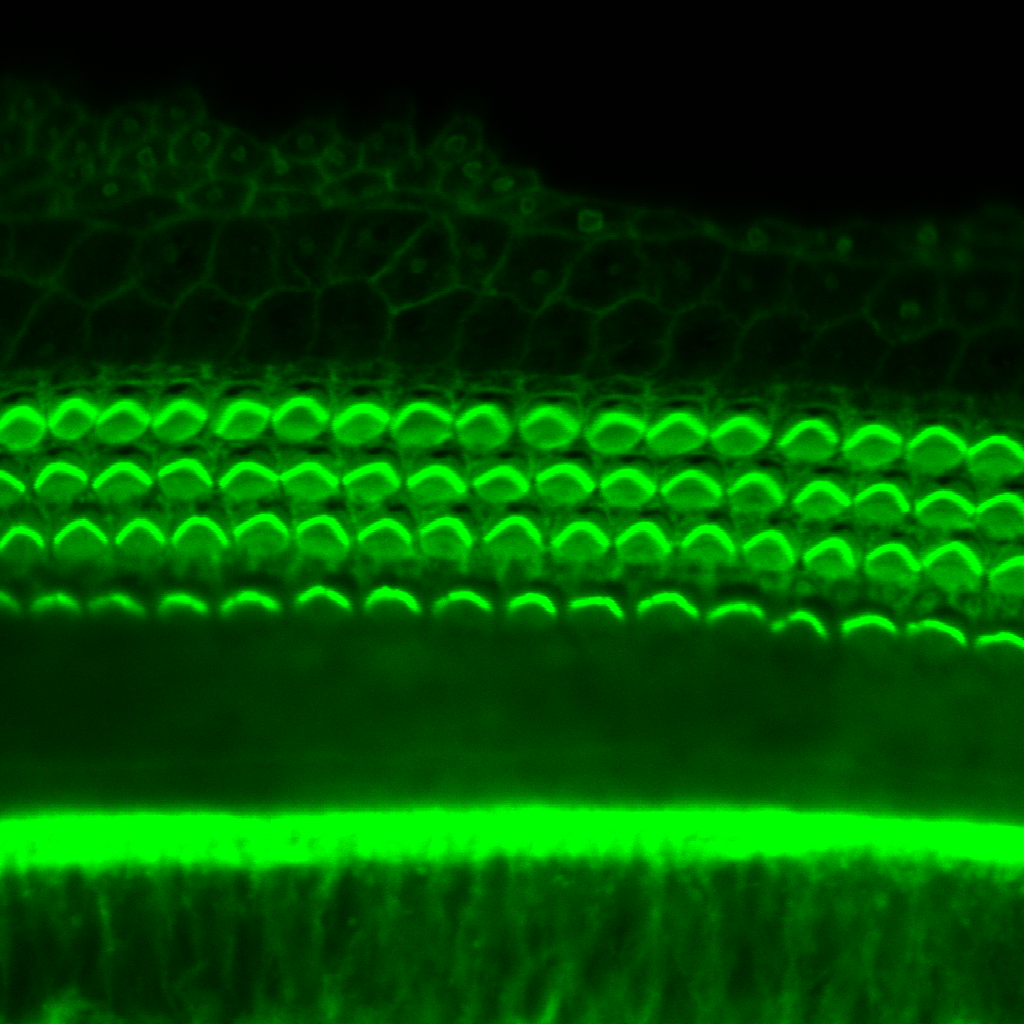

Supplement: Figure 2—figure supplement 1—source data 1. [file elife-76754-fig2-figsupp1-data1.zip › Figure 2 - figure supplement 1 Source data/Figure 2 - figure supplement 1 C/Rest-cko P1 base.tif]

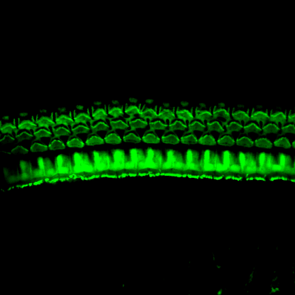

Supplement: Figure 2—figure supplement 1—source data 1. [file elife-76754-fig2-figsupp1-data1.zip › Figure 2 - figure supplement 1 Source data/Figure 2 - figure supplement 1 C/Rest-cko P14 base.tif]

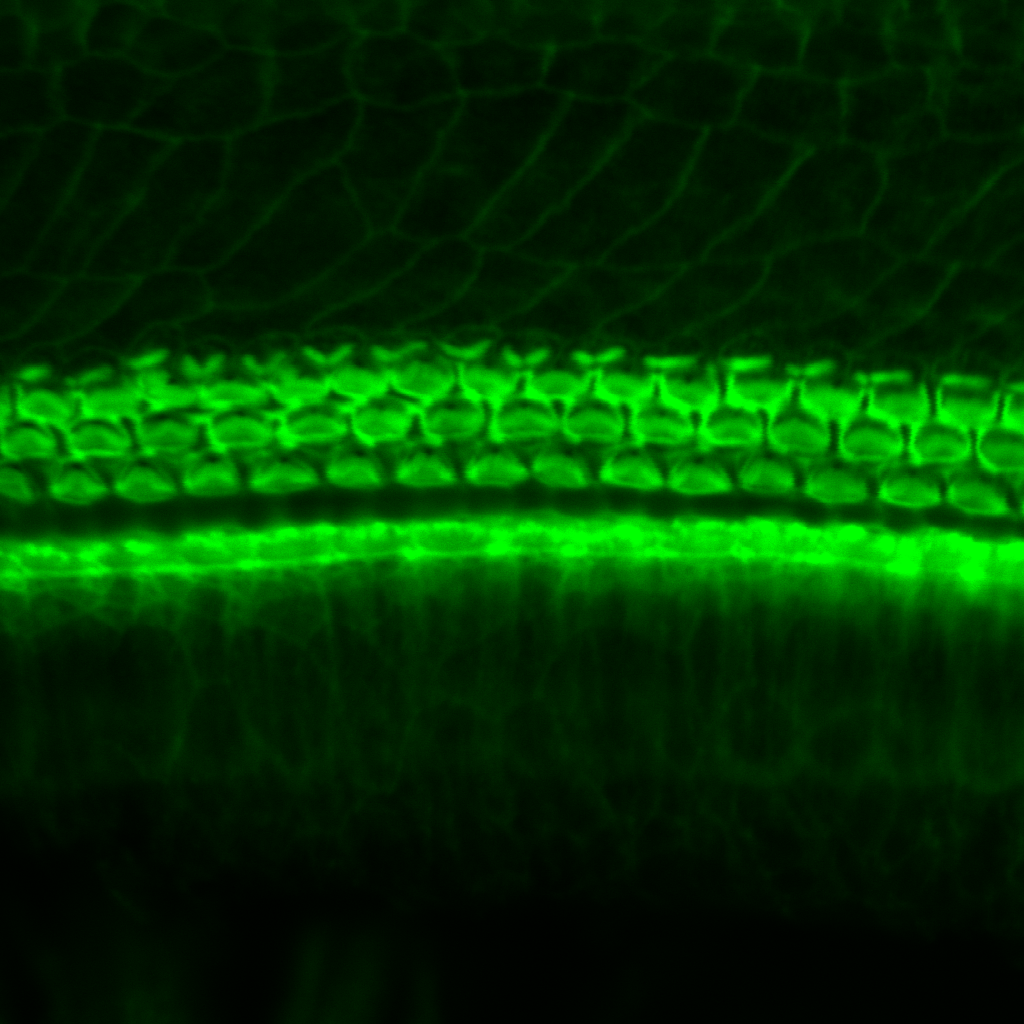

Supplement: Figure 2—figure supplement 1—source data 1. [file elife-76754-fig2-figsupp1-data1.zip › Figure 2 - figure supplement 1 Source data/Figure 2 - figure supplement 1 C/Rest-cko P7 base.tif]

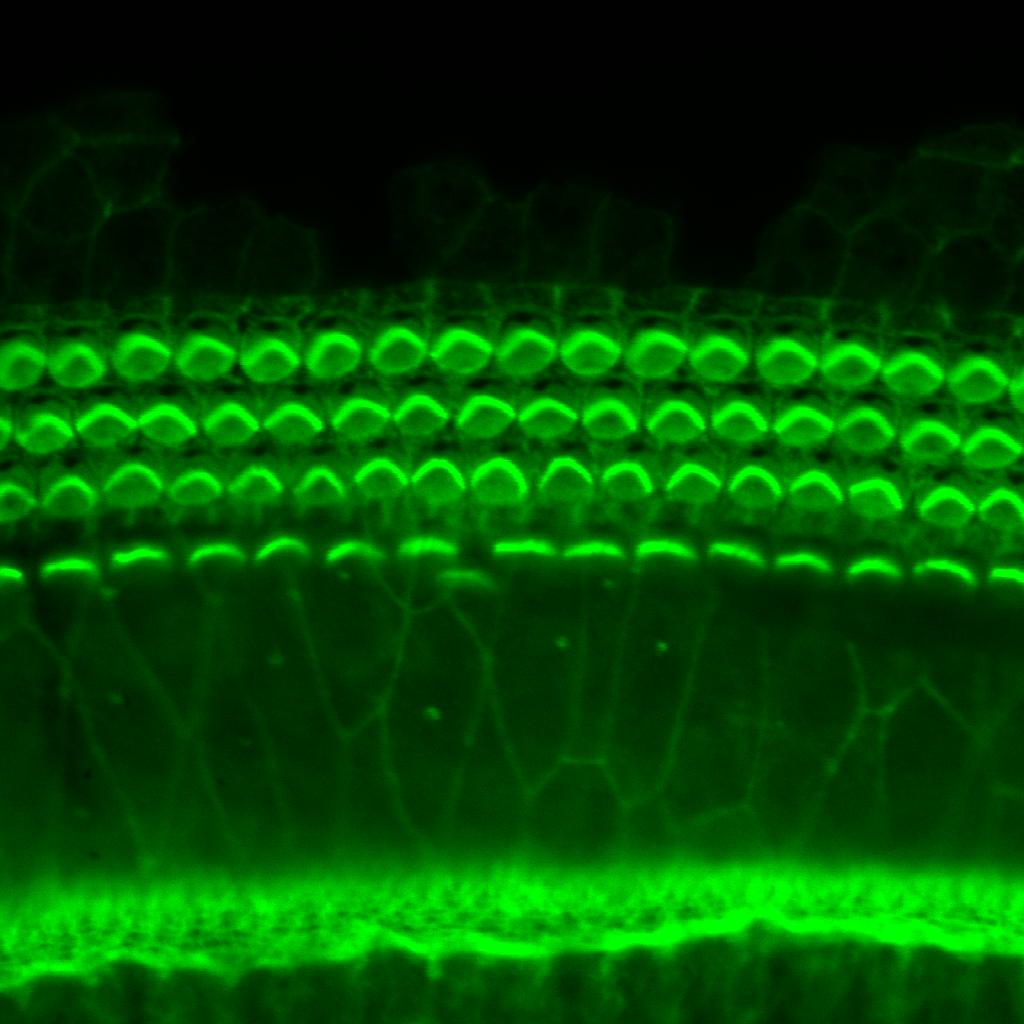

Supplement: Figure 2—figure supplement 1—source data 1. [file elife-76754-fig2-figsupp1-data1.zip › Figure 2 - figure supplement 1 Source data/Figure 2 - figure supplement 1 C/WT P1 base.tif]

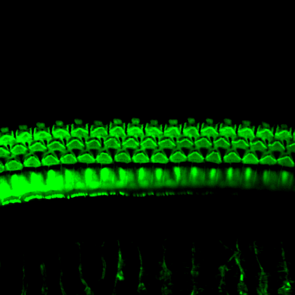

Supplement: Figure 2—figure supplement 1—source data 1. [file elife-76754-fig2-figsupp1-data1.zip › Figure 2 - figure supplement 1 Source data/Figure 2 - figure supplement 1 C/WT P14 base.tif]

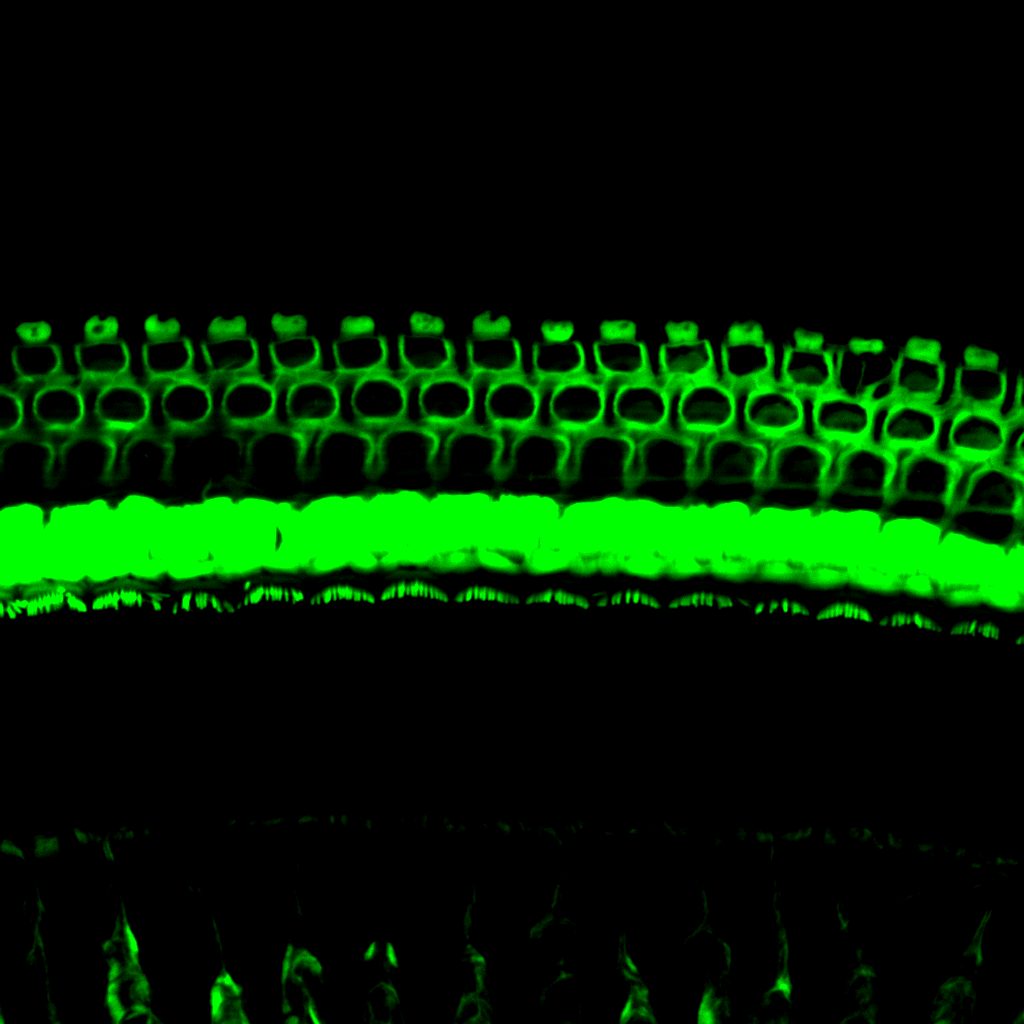

Supplement: Figure 2—figure supplement 1—source data 1. [file elife-76754-fig2-figsupp1-data1.zip › Figure 2 - figure supplement 1 Source data/Figure 2 - figure supplement 1 C/WT P7 base.tif]

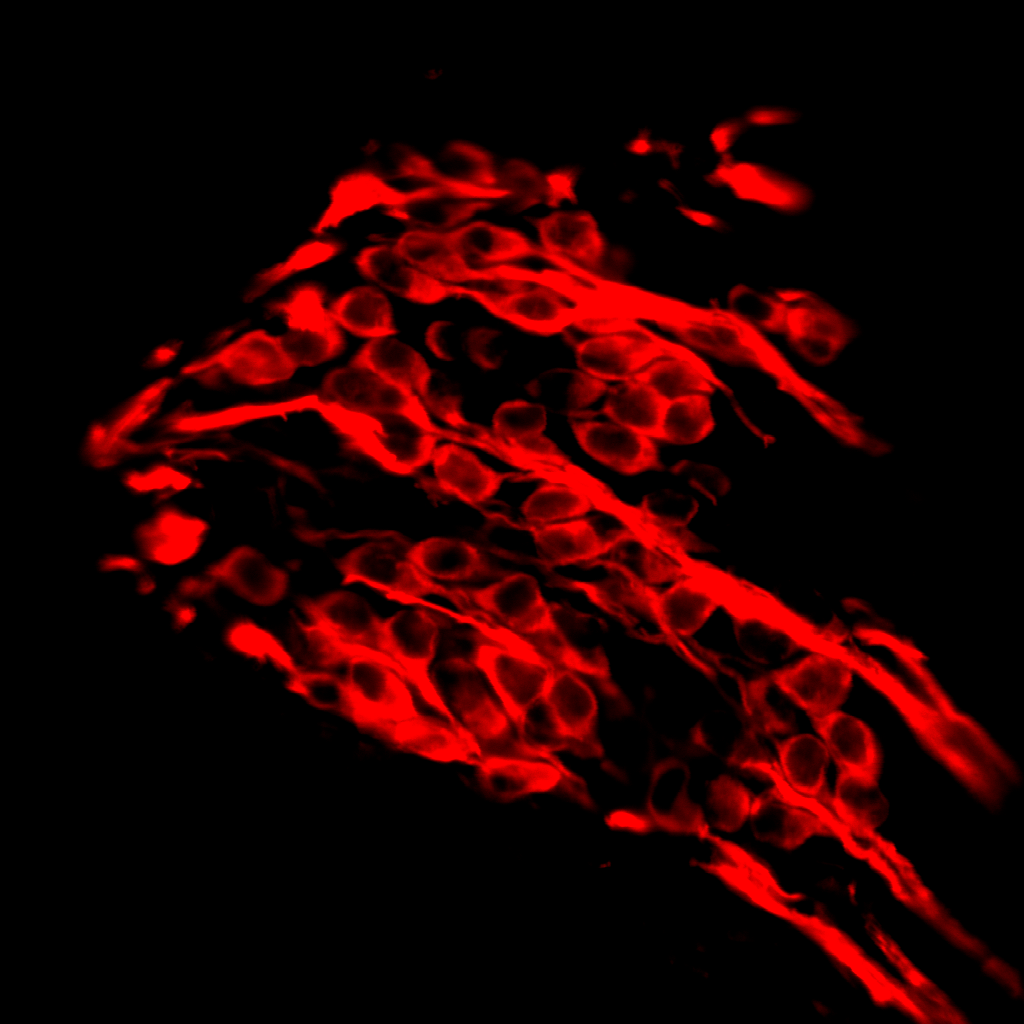

Supplement: Figure 2—figure supplement 1—source data 1. [file elife-76754-fig2-figsupp1-data1.zip › Figure 2 - figure supplement 1 Source data/Figure 2 - figure supplement 1 D/Rest cKO P1 apex SGN.tif]

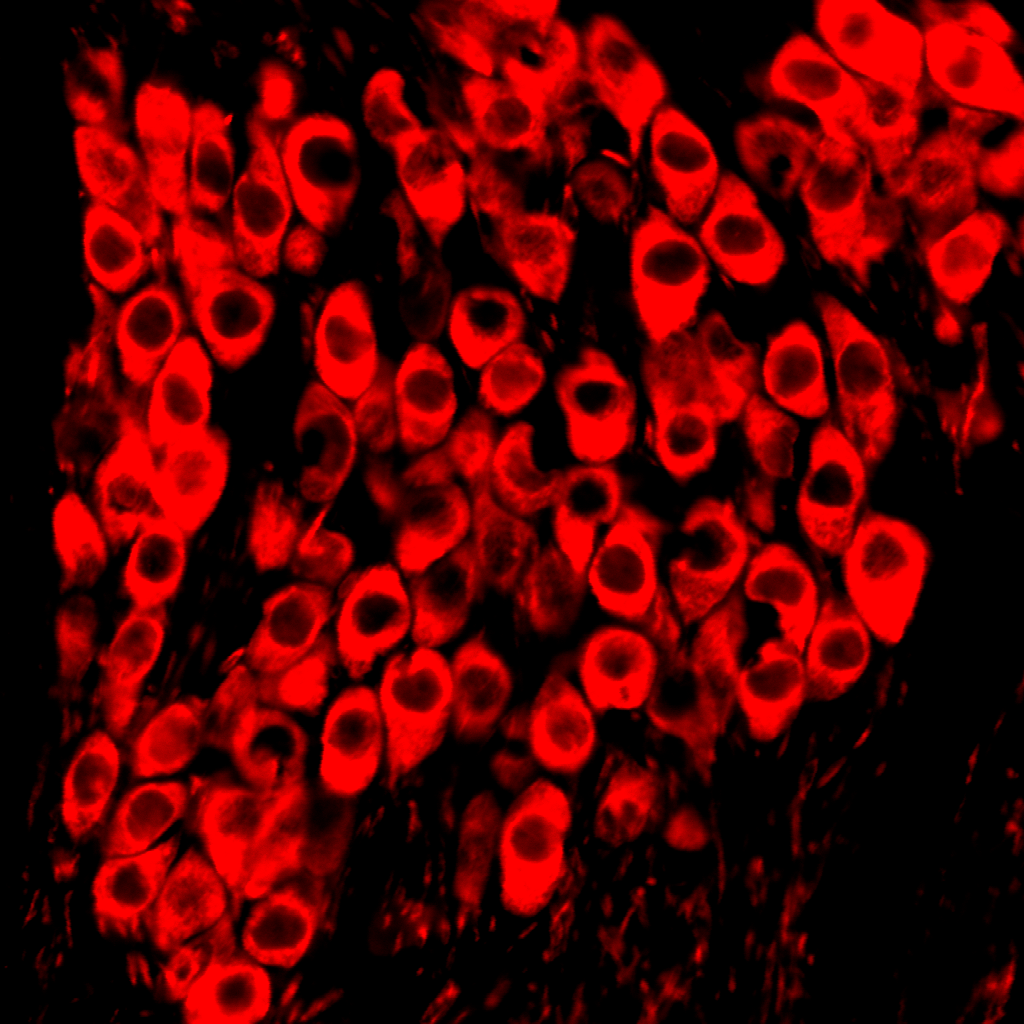

Supplement: Figure 2—figure supplement 1—source data 1. [file elife-76754-fig2-figsupp1-data1.zip › Figure 2 - figure supplement 1 Source data/Figure 2 - figure supplement 1 D/Rest cKO P14 apex SGN.tif]

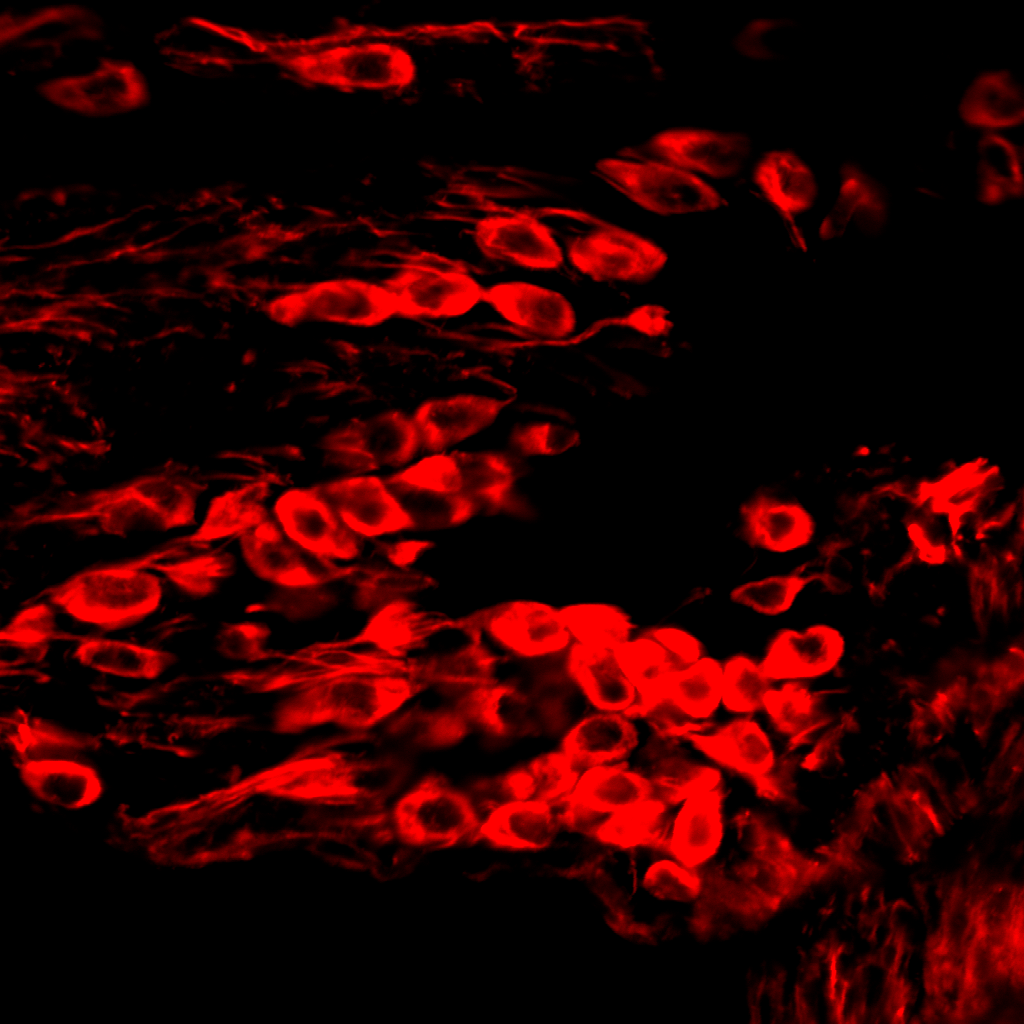

Supplement: Figure 2—figure supplement 1—source data 1. [file elife-76754-fig2-figsupp1-data1.zip › Figure 2 - figure supplement 1 Source data/Figure 2 - figure supplement 1 D/Rest cKO P7 apex SGN.tif]

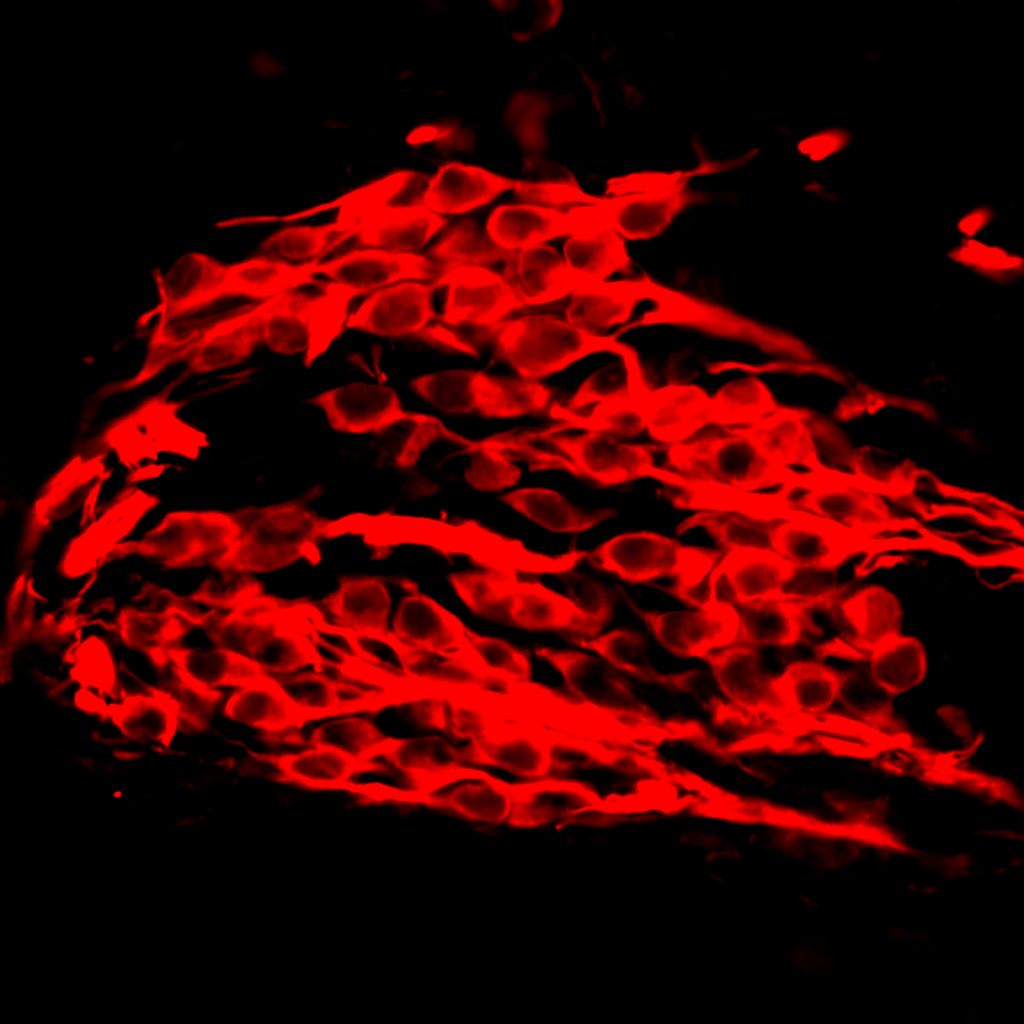

Supplement: Figure 2—figure supplement 1—source data 1. [file elife-76754-fig2-figsupp1-data1.zip › Figure 2 - figure supplement 1 Source data/Figure 2 - figure supplement 1 D/WT P1 apex SGN.tif]

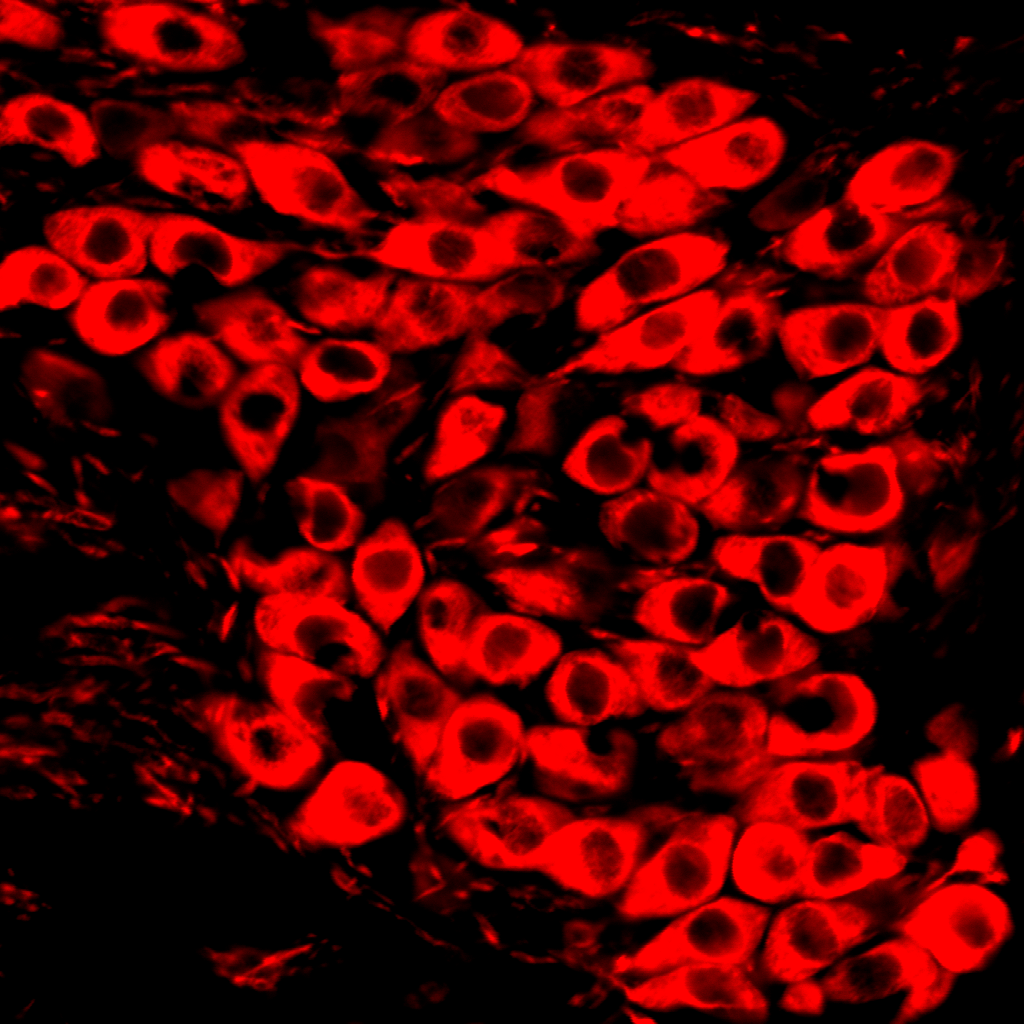

Supplement: Figure 2—figure supplement 1—source data 1. [file elife-76754-fig2-figsupp1-data1.zip › Figure 2 - figure supplement 1 Source data/Figure 2 - figure supplement 1 D/WT P14 apex SGN.tif]

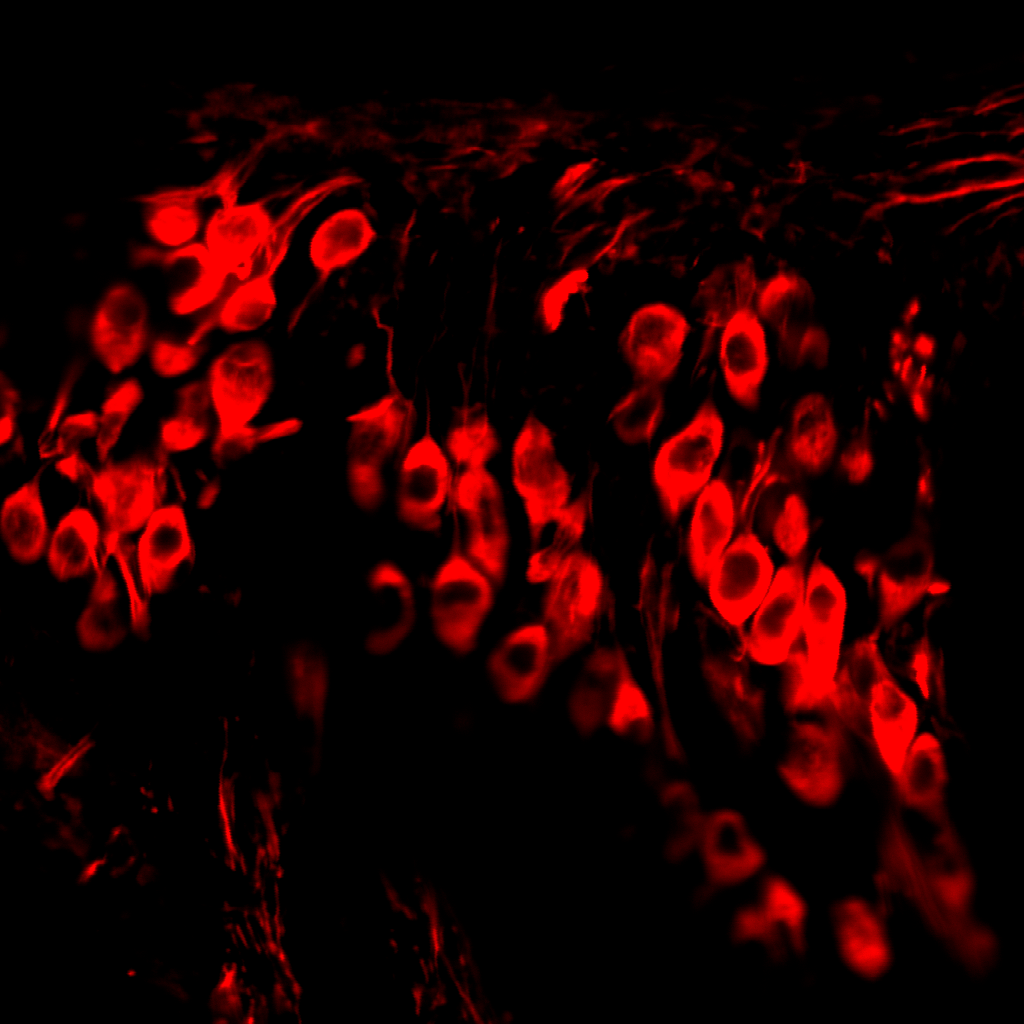

Supplement: Figure 2—figure supplement 1—source data 1. [file elife-76754-fig2-figsupp1-data1.zip › Figure 2 - figure supplement 1 Source data/Figure 2 - figure supplement 1 D/WT P7 apex SGN.tif]

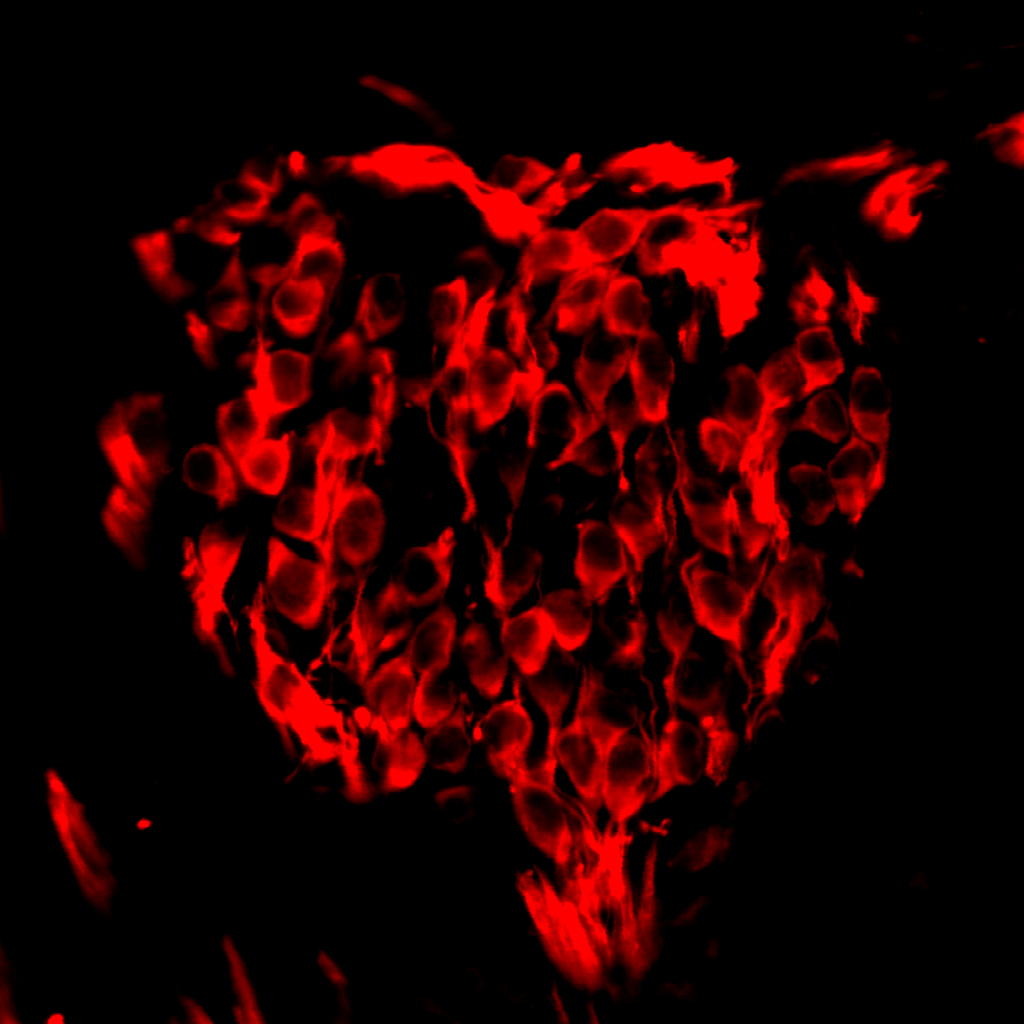

Supplement: Figure 2—figure supplement 1—source data 1. [file elife-76754-fig2-figsupp1-data1.zip › Figure 2 - figure supplement 1 Source data/Figure 2 - figure supplement 1 E/Rest cKO P1 middle SGN.tif]

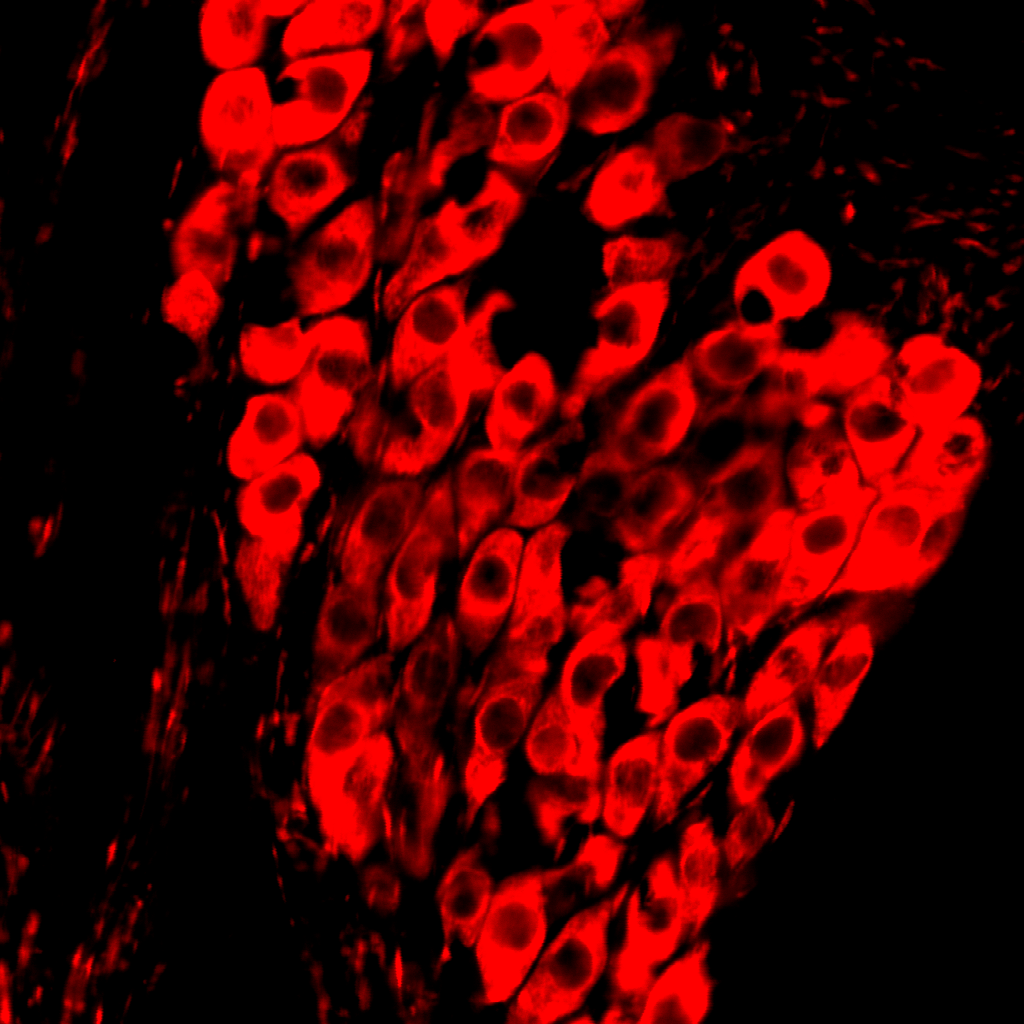

Supplement: Figure 2—figure supplement 1—source data 1. [file elife-76754-fig2-figsupp1-data1.zip › Figure 2 - figure supplement 1 Source data/Figure 2 - figure supplement 1 E/Rest cKO P14 middle SGN.tif]

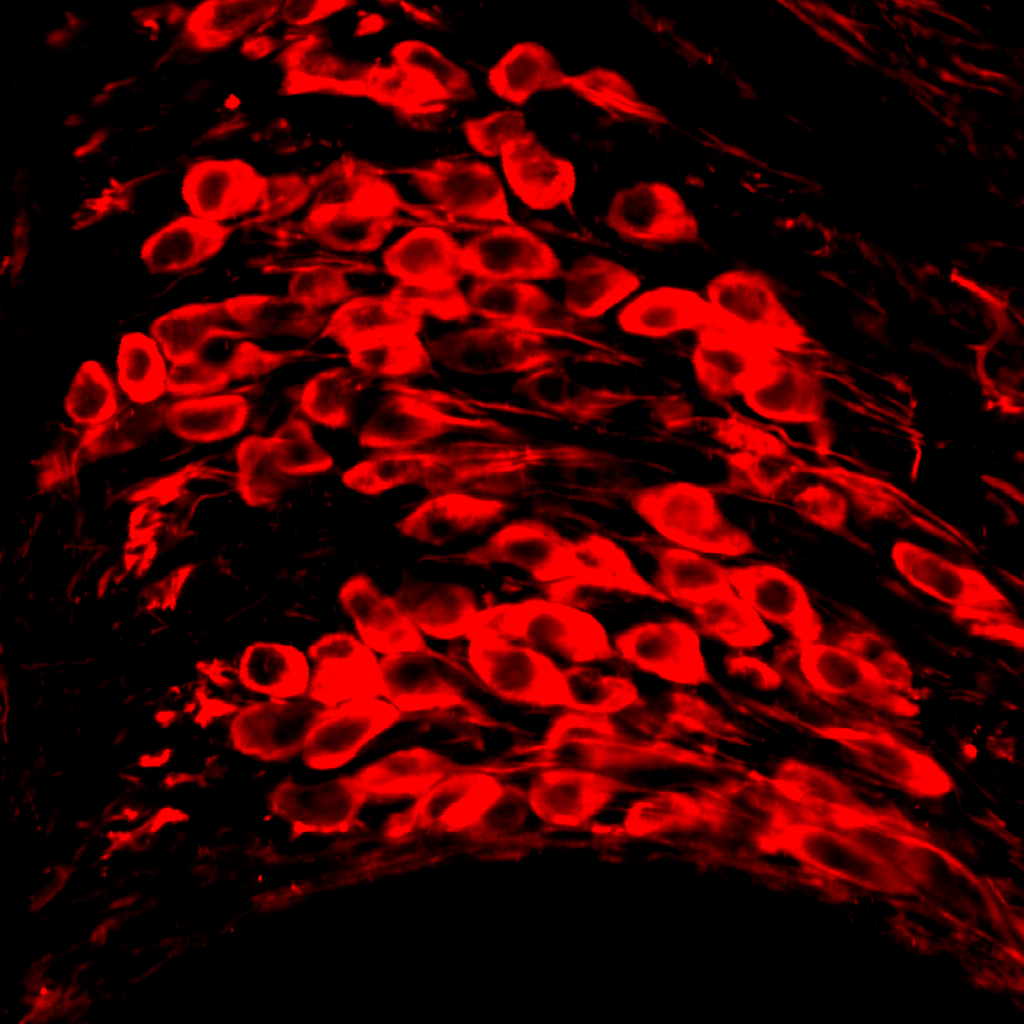

Supplement: Figure 2—figure supplement 1—source data 1. [file elife-76754-fig2-figsupp1-data1.zip › Figure 2 - figure supplement 1 Source data/Figure 2 - figure supplement 1 E/Rest cKO P7 middle SGN.tif]

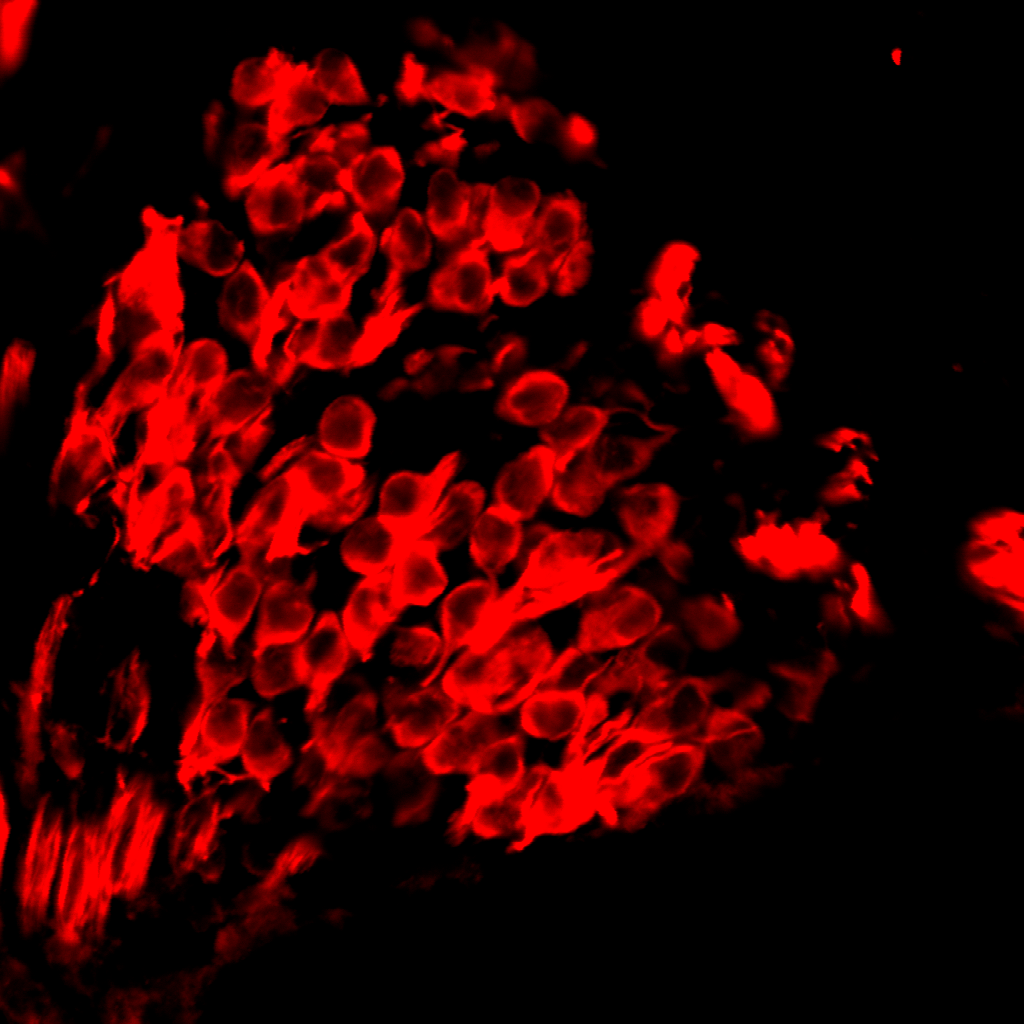

Supplement: Figure 2—figure supplement 1—source data 1. [file elife-76754-fig2-figsupp1-data1.zip › Figure 2 - figure supplement 1 Source data/Figure 2 - figure supplement 1 E/WT P1 middle SGN.tif]

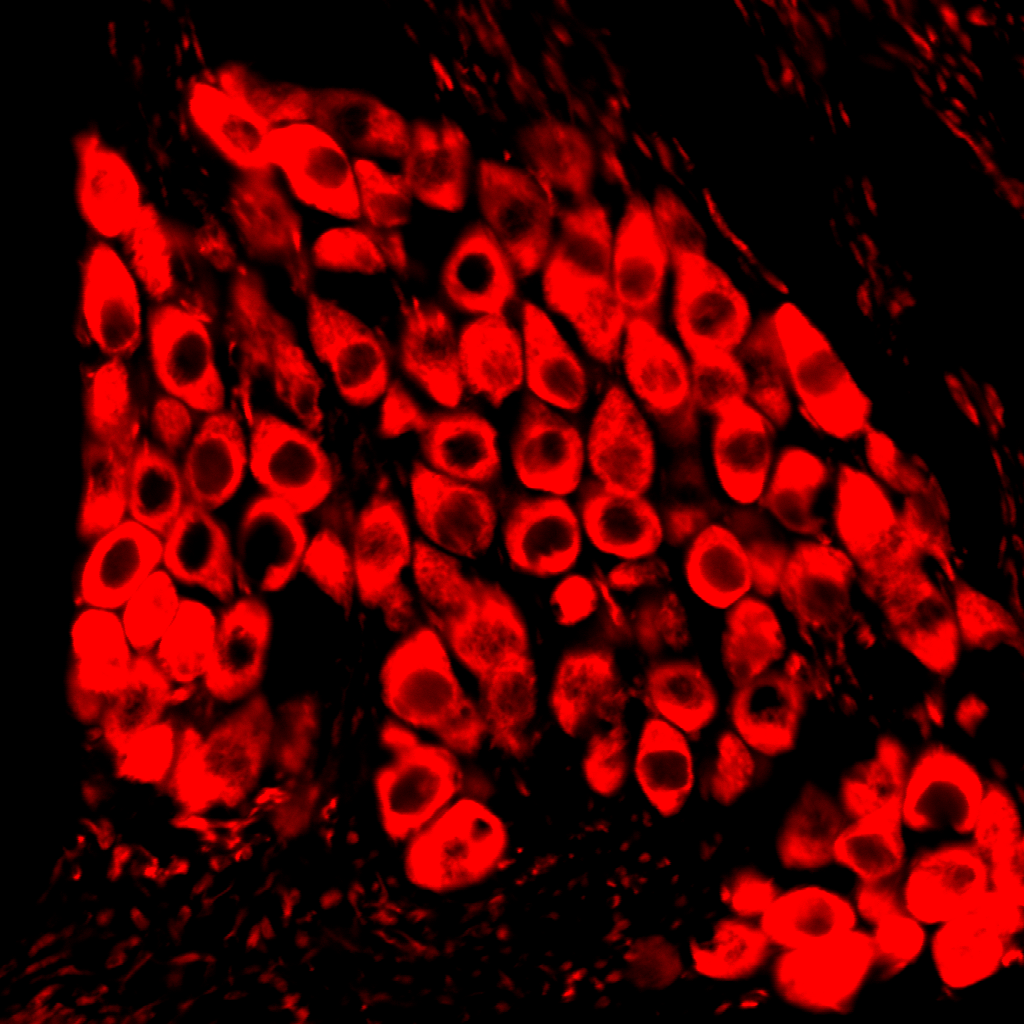

Supplement: Figure 2—figure supplement 1—source data 1. [file elife-76754-fig2-figsupp1-data1.zip › Figure 2 - figure supplement 1 Source data/Figure 2 - figure supplement 1 E/WT P14 middle SGN.tif]
