# Supplementary figures and images for: Repressor element 1-silencing transcription factor deficiency yields profound hearing loss through Kv7.4 channel upsurge in auditory neurons and hair cells (part 2 of 3)
Source: eLife. 2022 Sep 20;11:e76754. doi: 10.7554/eLife.76754 (PMC9525063; doi:10.7554/eLife.76754)

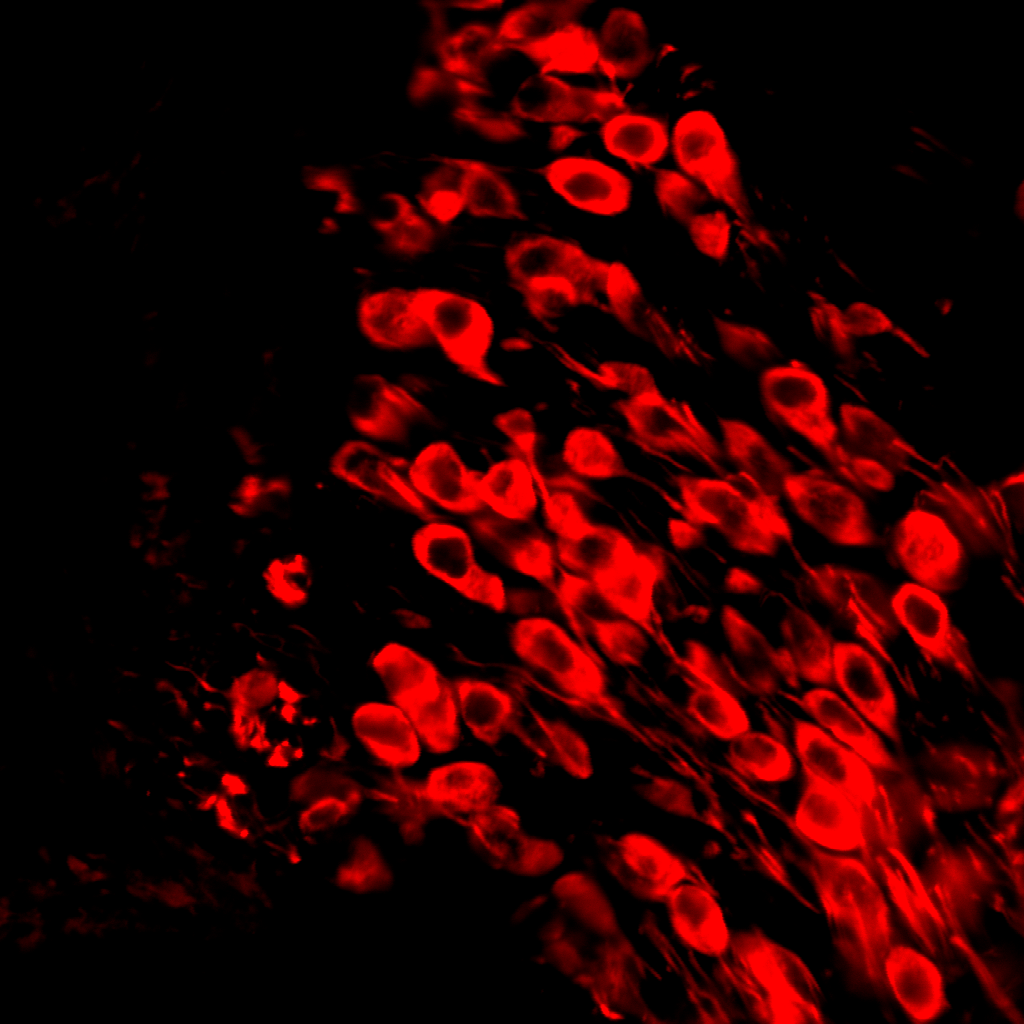

Supplement: Figure 2—figure supplement 1—source data 1. [file elife-76754-fig2-figsupp1-data1.zip › Figure 2 - figure supplement 1 Source data/Figure 2 - figure supplement 1 E/WT P7 middle SGN.tif]

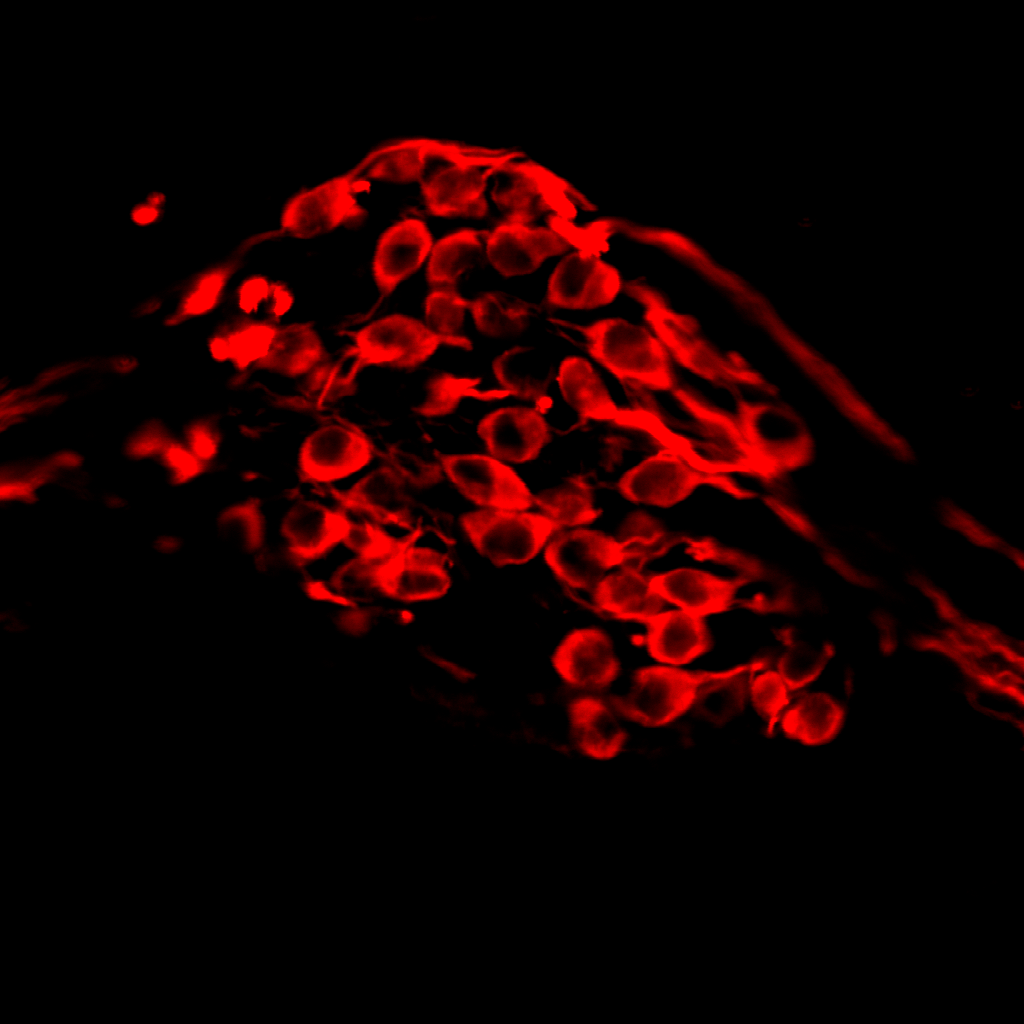

Supplement: Figure 2—figure supplement 1—source data 1. [file elife-76754-fig2-figsupp1-data1.zip › Figure 2 - figure supplement 1 Source data/Figure 2 - figure supplement 1 F/Rest cKO P1 base SGN.tif]

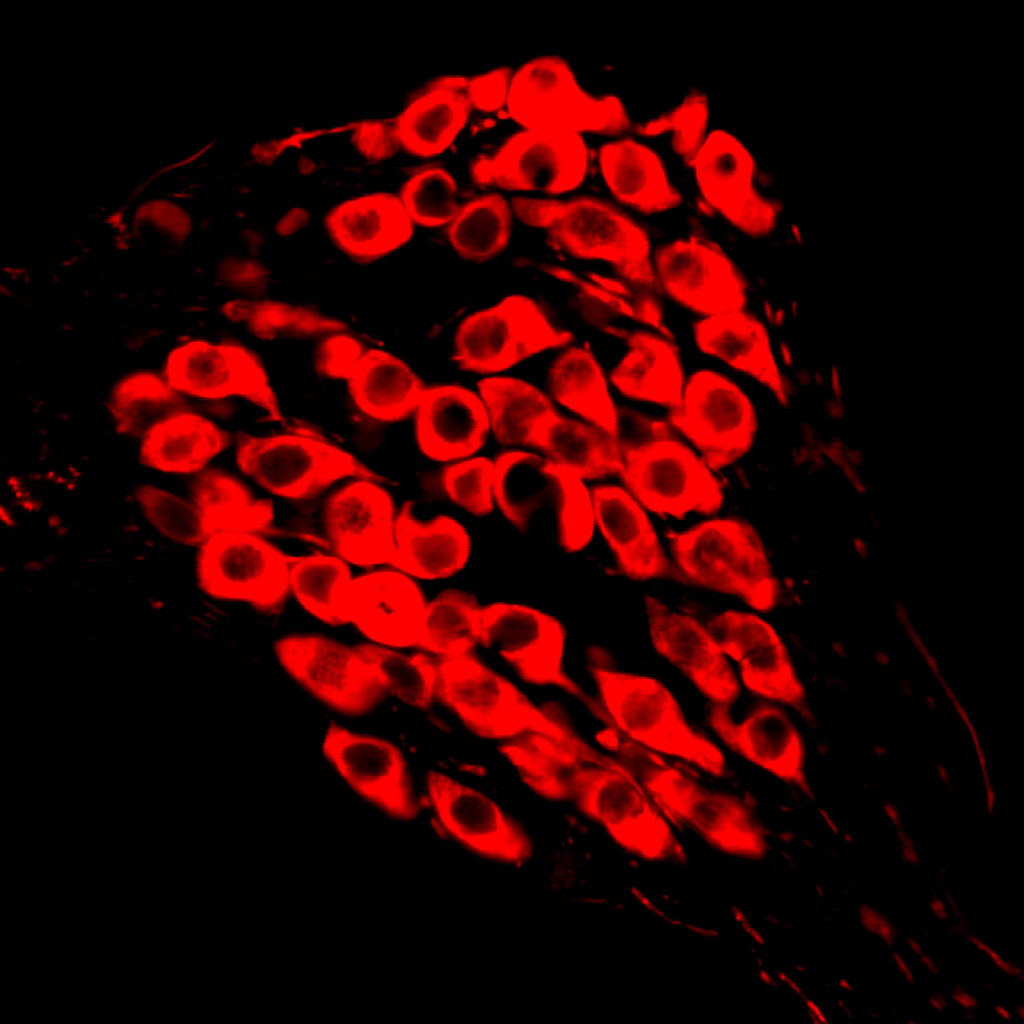

Supplement: Figure 2—figure supplement 1—source data 1. [file elife-76754-fig2-figsupp1-data1.zip › Figure 2 - figure supplement 1 Source data/Figure 2 - figure supplement 1 F/Rest cKO P14 base SGN.tif]

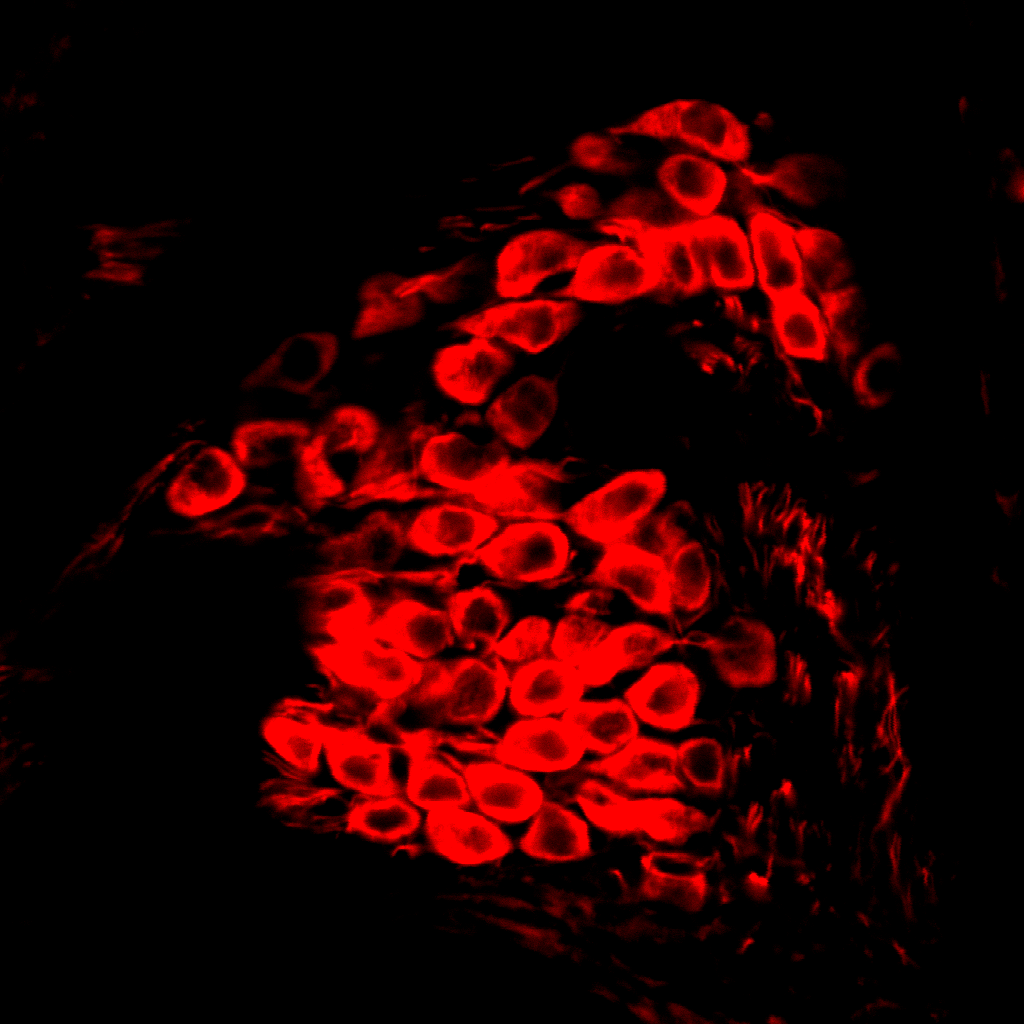

Supplement: Figure 2—figure supplement 1—source data 1. [file elife-76754-fig2-figsupp1-data1.zip › Figure 2 - figure supplement 1 Source data/Figure 2 - figure supplement 1 F/Rest cKO P7 base SGN.tif]

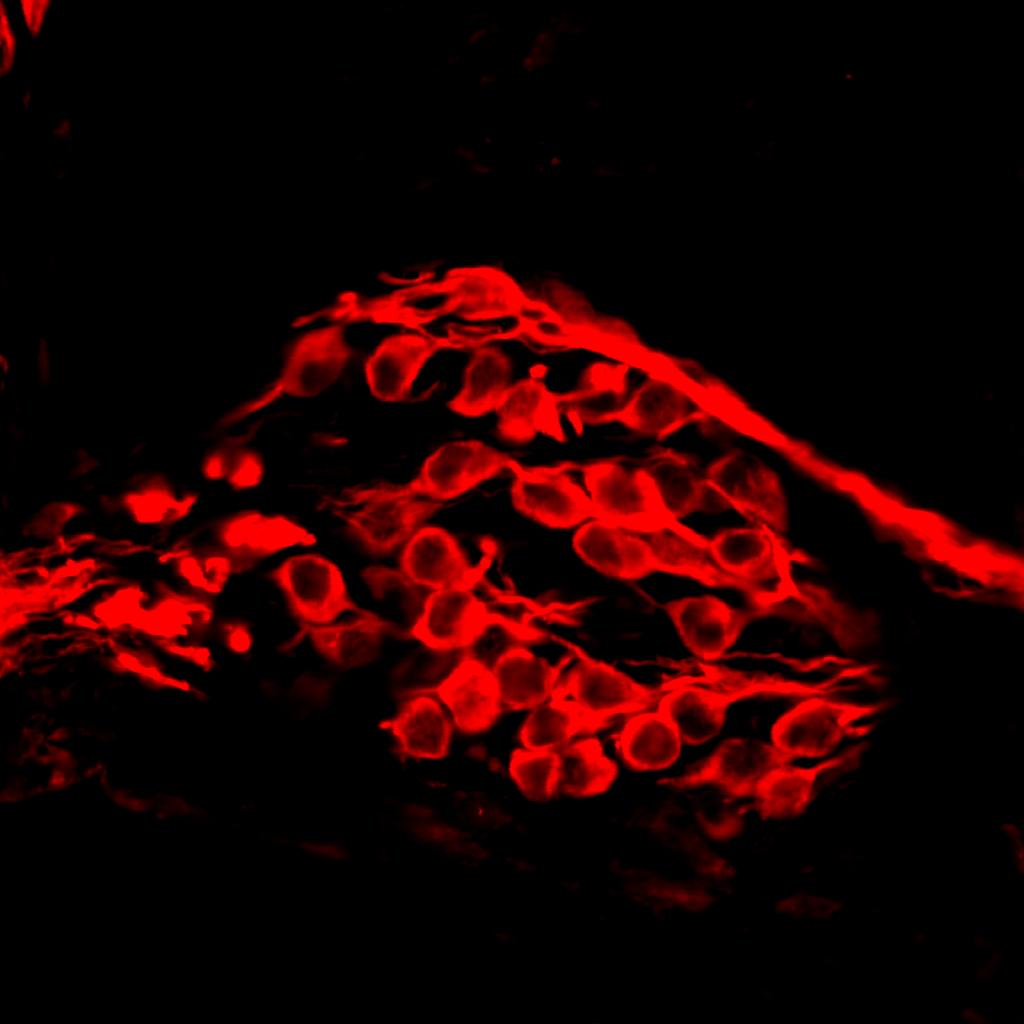

Supplement: Figure 2—figure supplement 1—source data 1. [file elife-76754-fig2-figsupp1-data1.zip › Figure 2 - figure supplement 1 Source data/Figure 2 - figure supplement 1 F/WT P1 base SGN.tif]

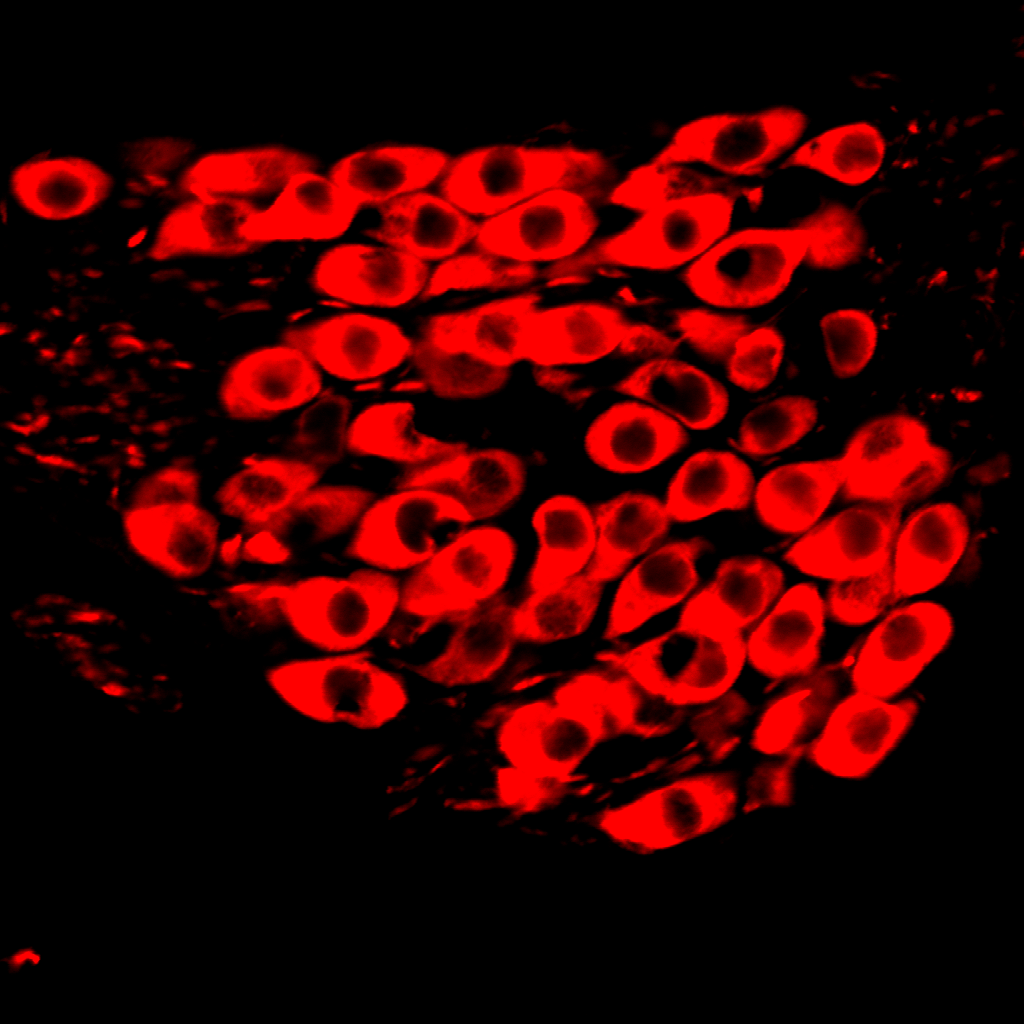

Supplement: Figure 2—figure supplement 1—source data 1. [file elife-76754-fig2-figsupp1-data1.zip › Figure 2 - figure supplement 1 Source data/Figure 2 - figure supplement 1 F/WT P14 base SGN.tif]

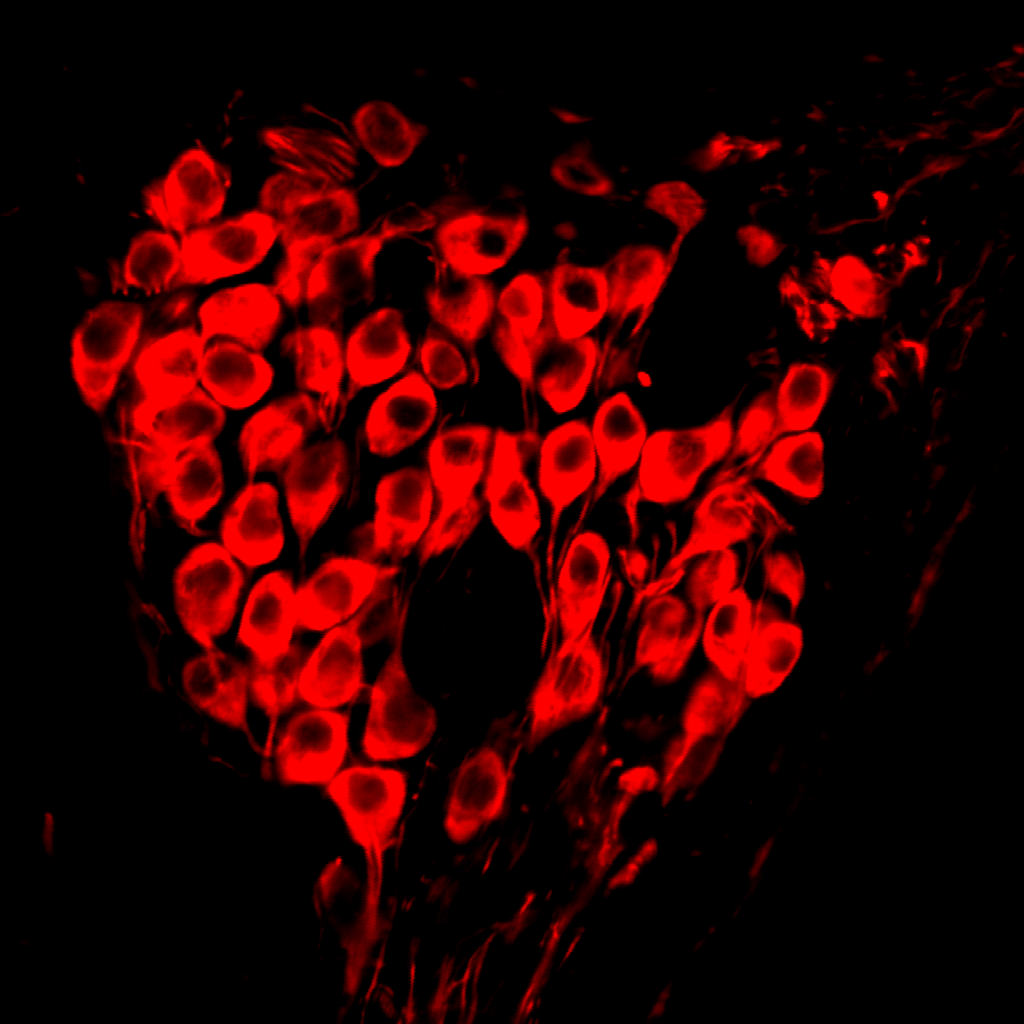

Supplement: Figure 2—figure supplement 1—source data 1. [file elife-76754-fig2-figsupp1-data1.zip › Figure 2 - figure supplement 1 Source data/Figure 2 - figure supplement 1 F/WT P7 base SGN.tif]

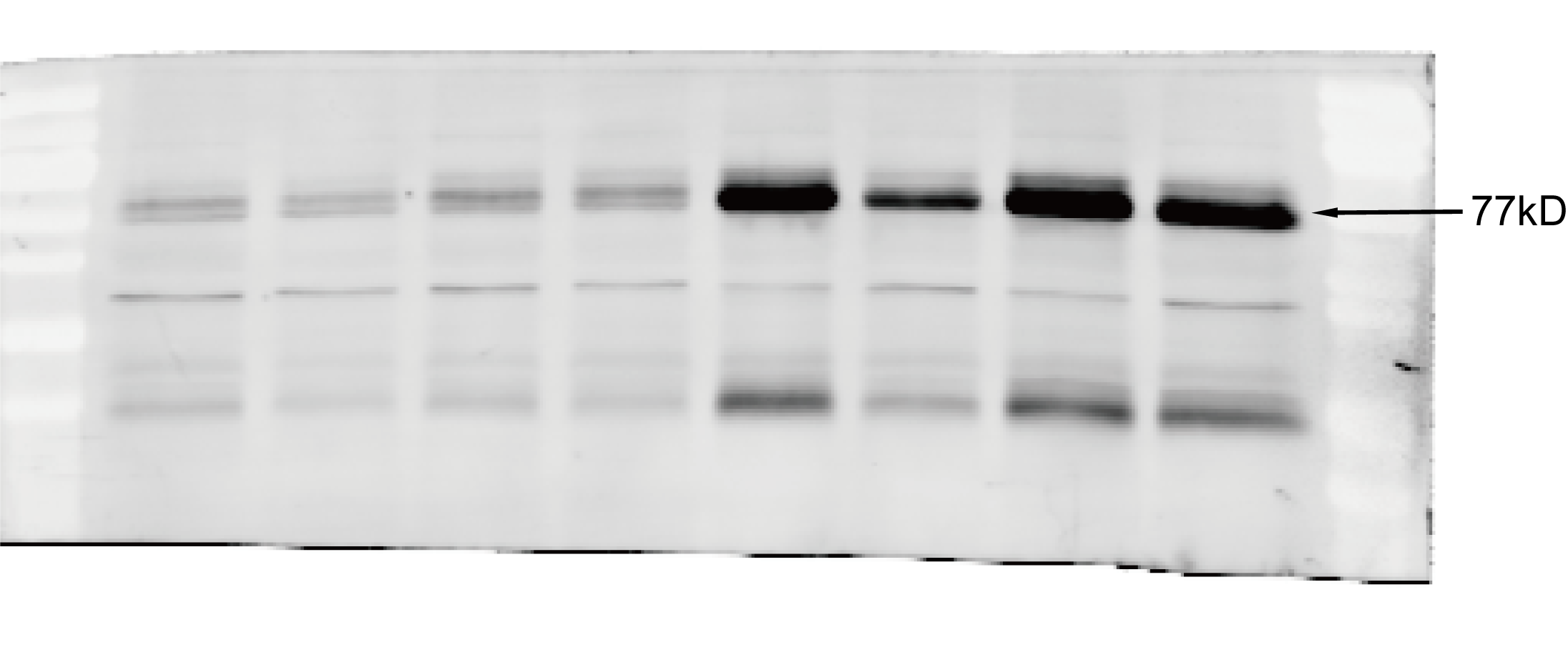

Supplement: Figure 4—source data 1. [file elife-76754-fig4-data1.zip › Figure_4_source_data/Fig.4 B/Figure_4 B-Kv7.4 protein expression.tif]

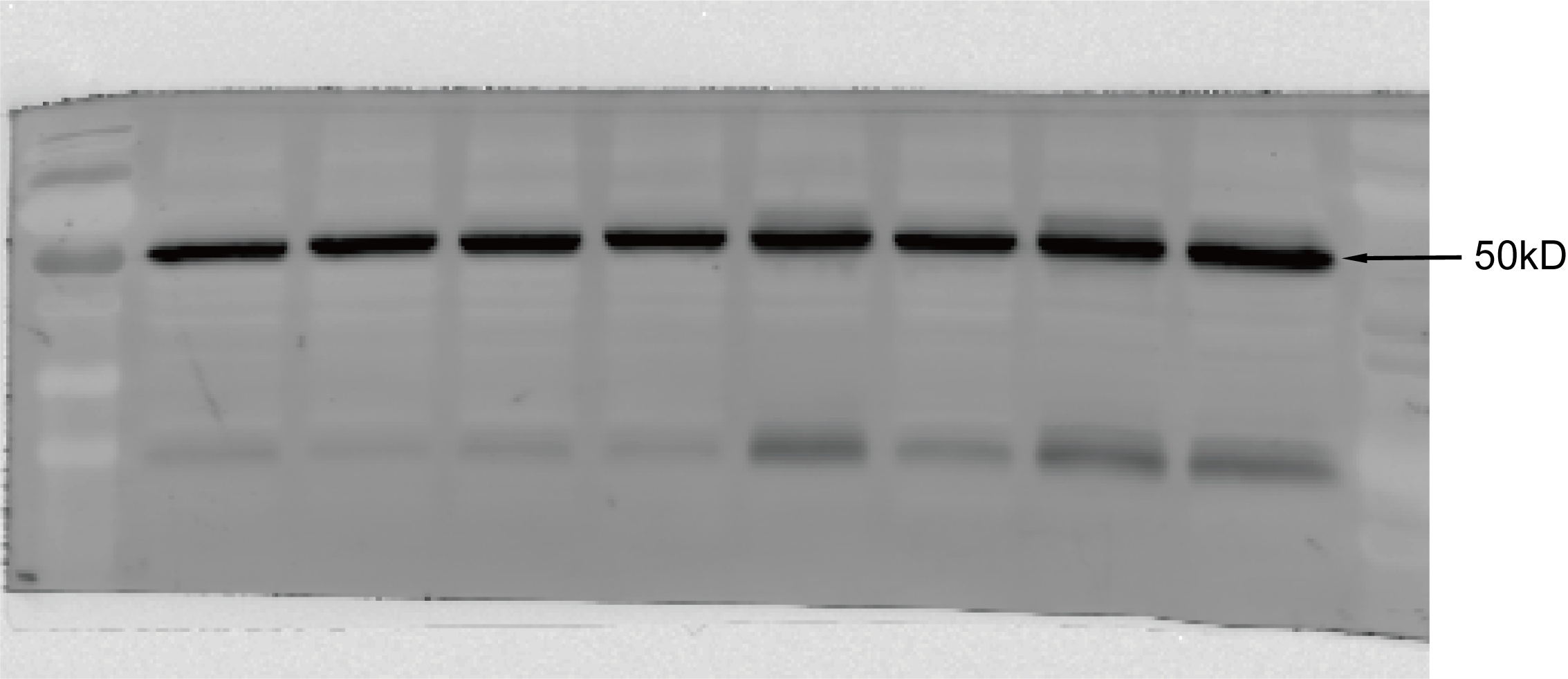

Supplement: Figure 4—source data 1. [file elife-76754-fig4-data1.zip › Figure_4_source_data/Fig.4 B/Figure_4 B-Tublin protein expression.tif]

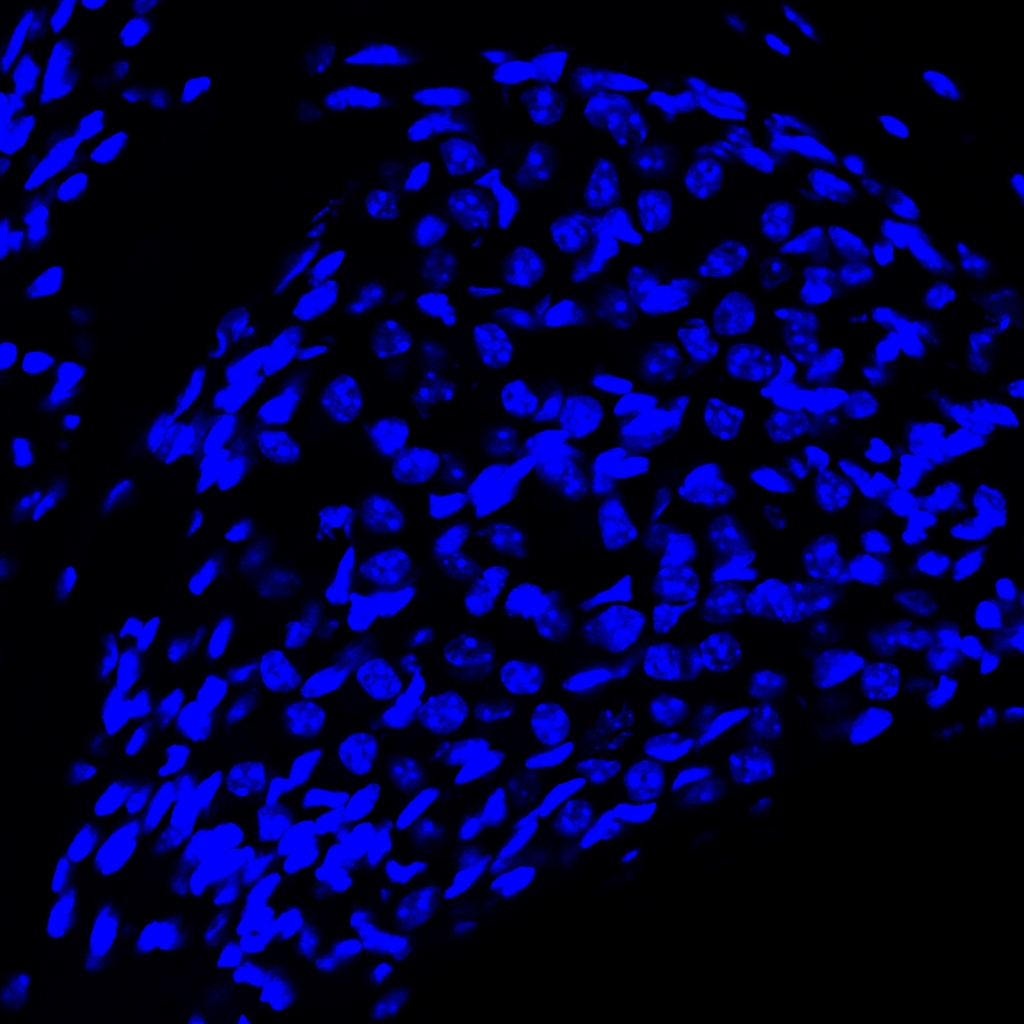

Supplement: Figure 4—source data 1. [file elife-76754-fig4-data1.zip › Figure_4_source_data/Fig.4 C/REST cKO DAPI Apex.tif]

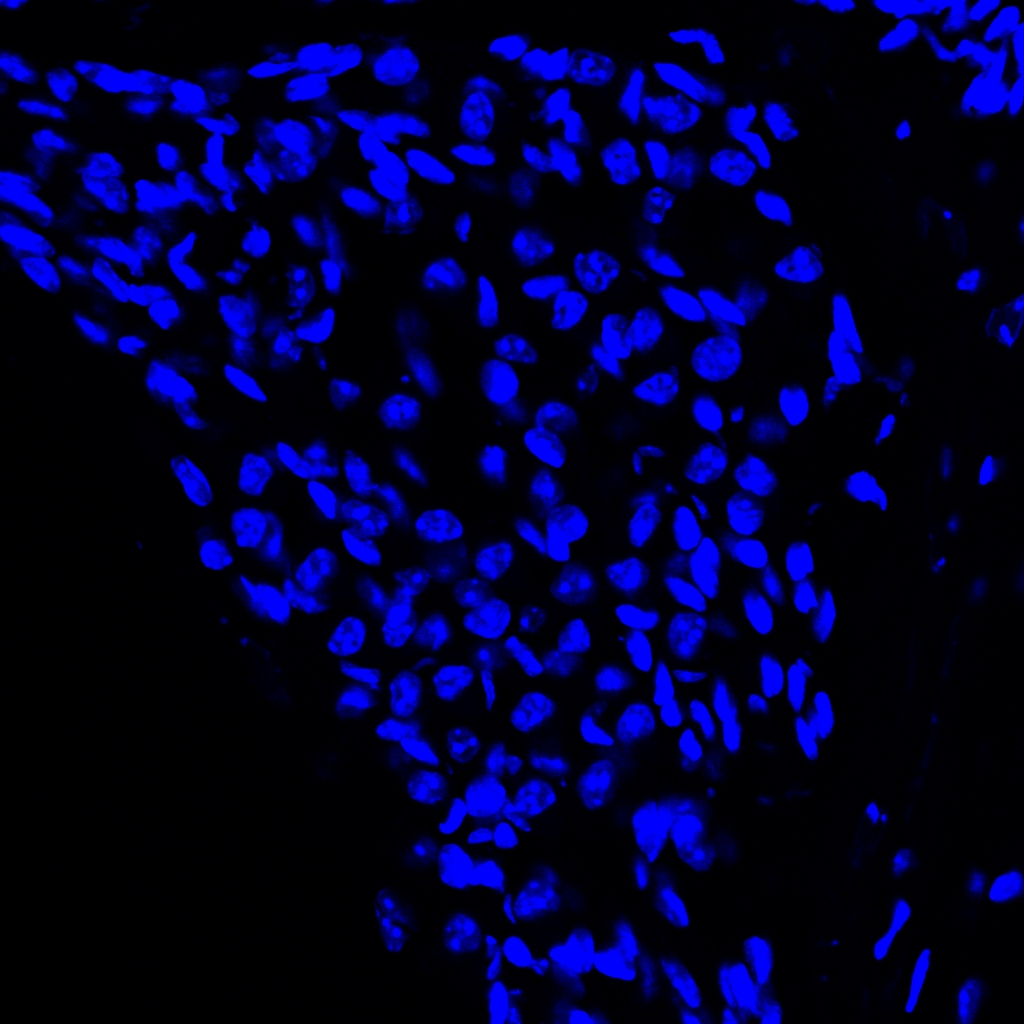

Supplement: Figure 4—source data 1. [file elife-76754-fig4-data1.zip › Figure_4_source_data/Fig.4 C/REST cKO DAPI Base.tif]

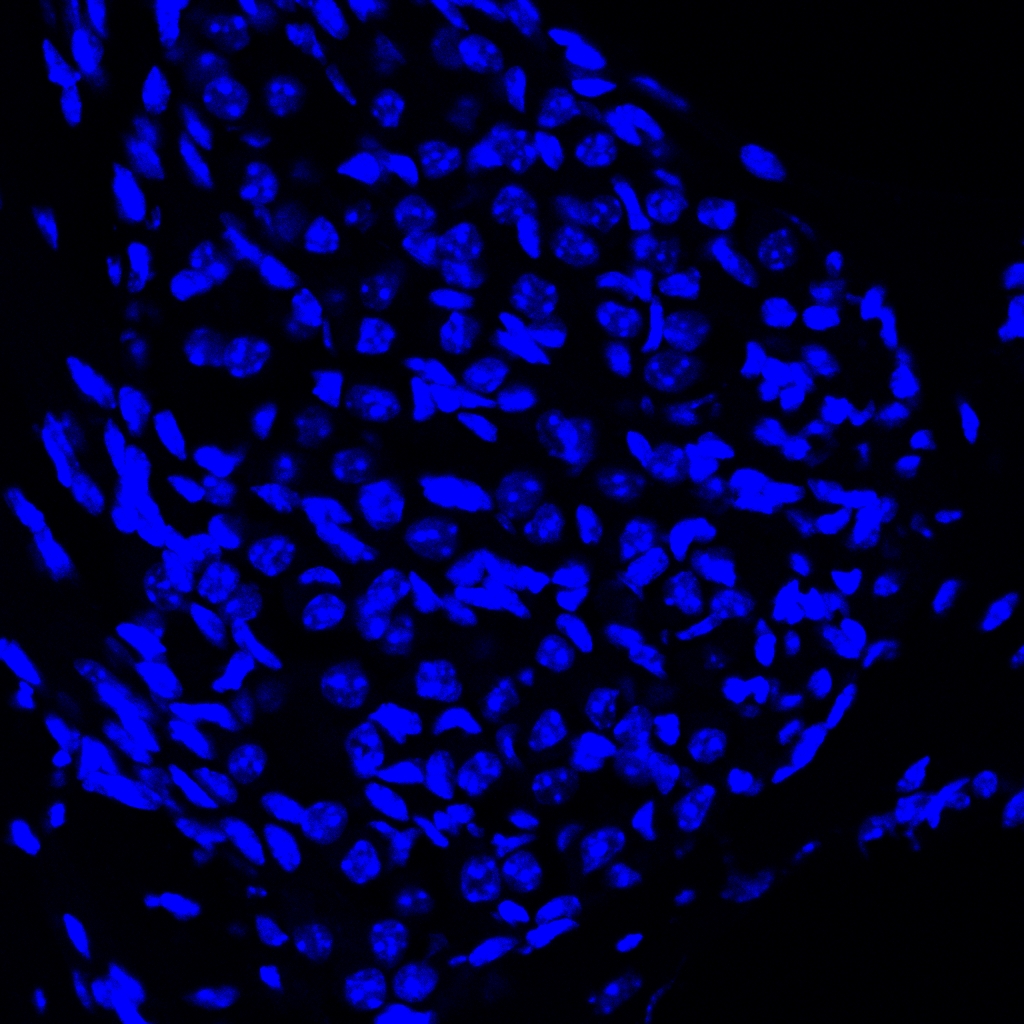

Supplement: Figure 4—source data 1. [file elife-76754-fig4-data1.zip › Figure_4_source_data/Fig.4 C/REST cKO DAPI Middle.tif]

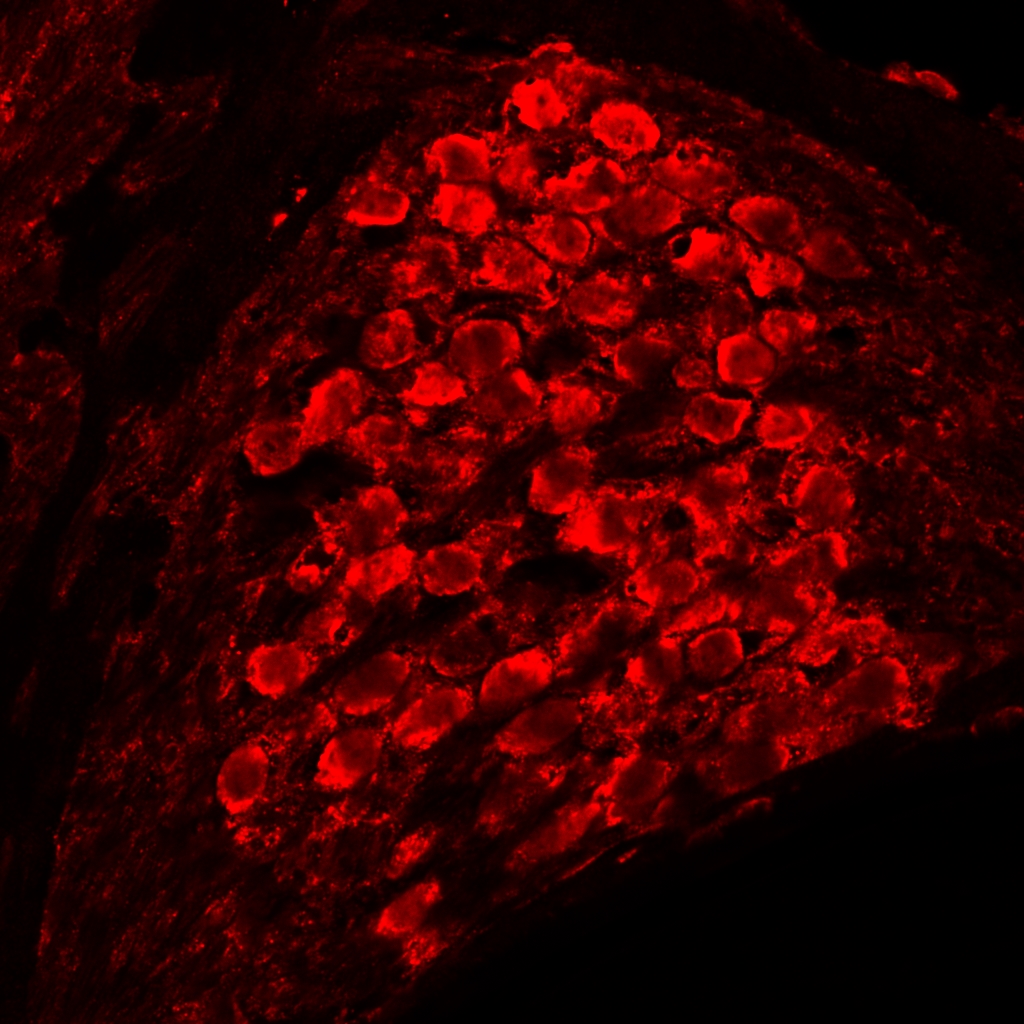

Supplement: Figure 4—source data 1. [file elife-76754-fig4-data1.zip › Figure_4_source_data/Fig.4 C/REST cKO Kv7.4 Apex.tif]

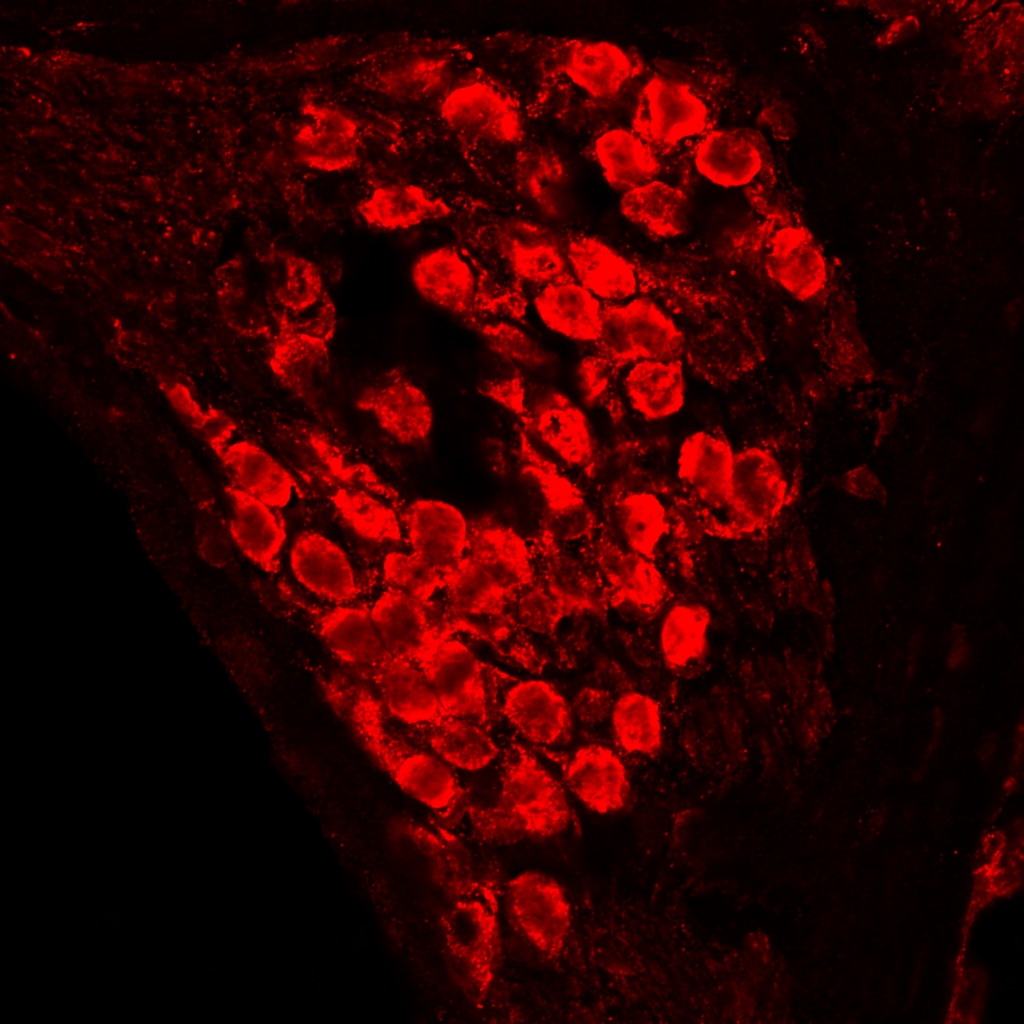

Supplement: Figure 4—source data 1. [file elife-76754-fig4-data1.zip › Figure_4_source_data/Fig.4 C/REST cKO Kv7.4 Base.tif]

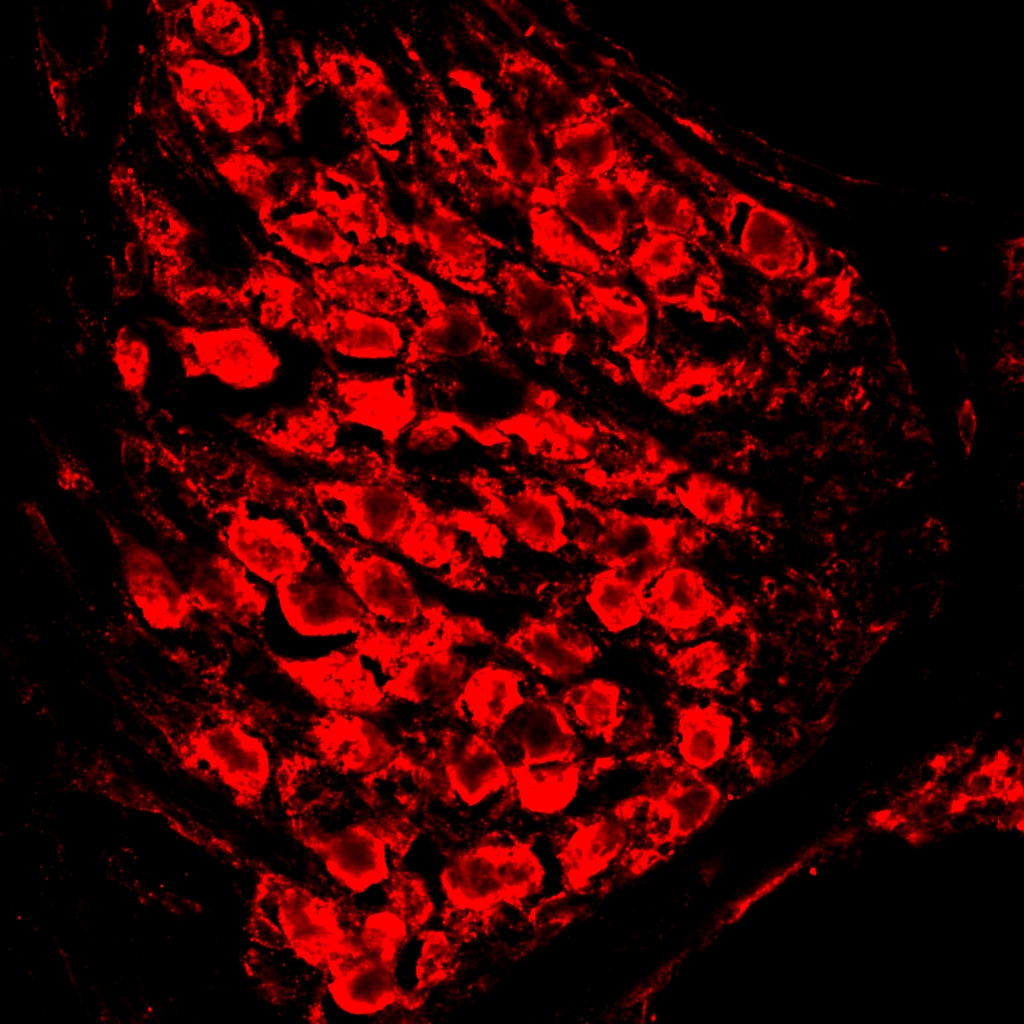

Supplement: Figure 4—source data 1. [file elife-76754-fig4-data1.zip › Figure_4_source_data/Fig.4 C/REST cKO Kv7.4 Middle.tif]

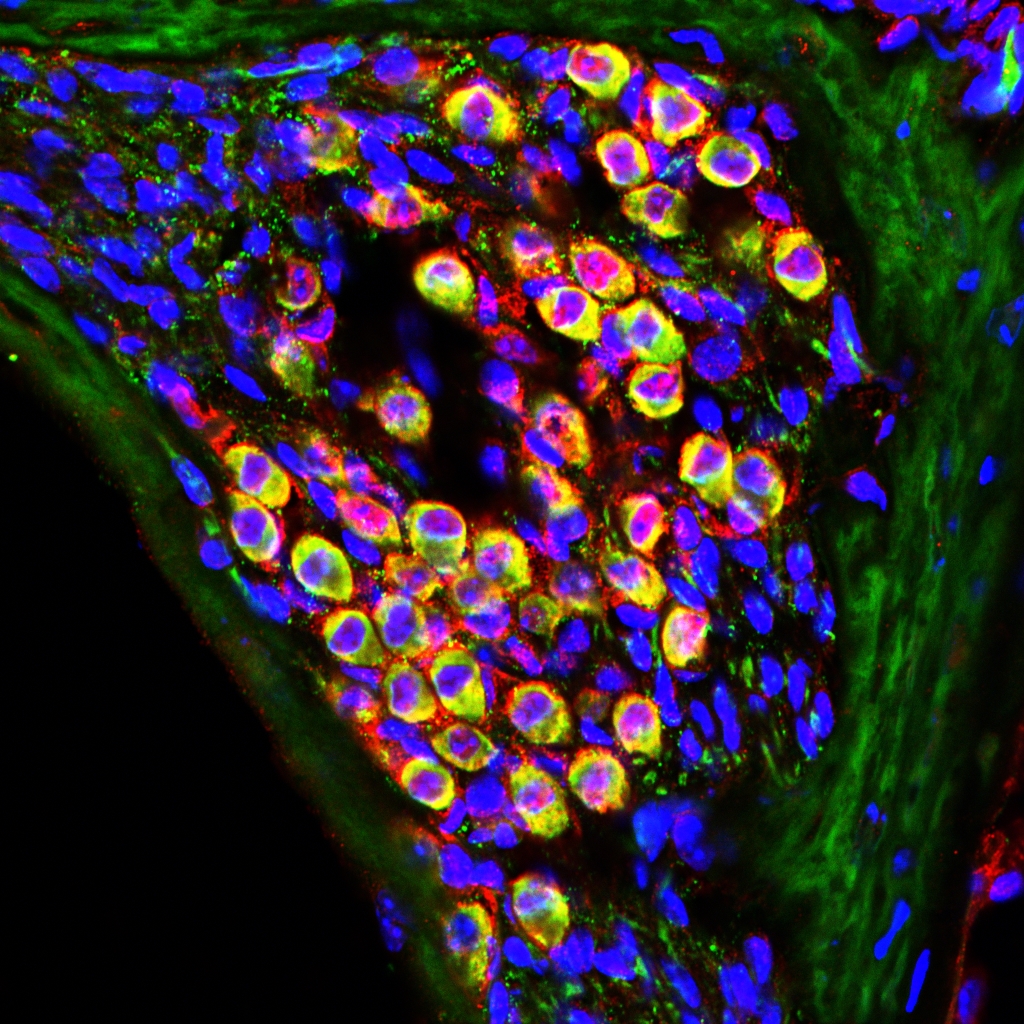

Supplement: Figure 4—source data 1. [file elife-76754-fig4-data1.zip › Figure_4_source_data/Fig.4 C/REST cKO Merge Base.tif]

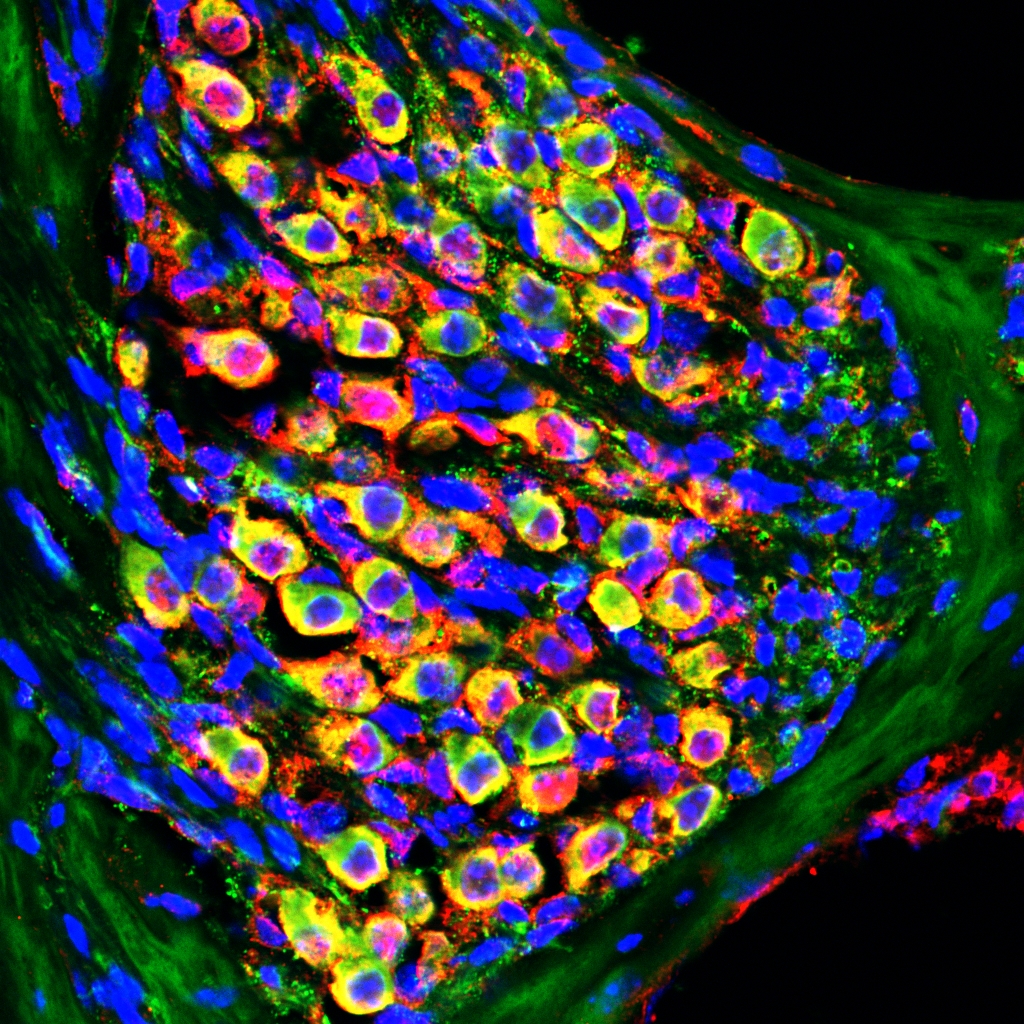

Supplement: Figure 4—source data 1. [file elife-76754-fig4-data1.zip › Figure_4_source_data/Fig.4 C/REST cKO Merge Middle.tif]

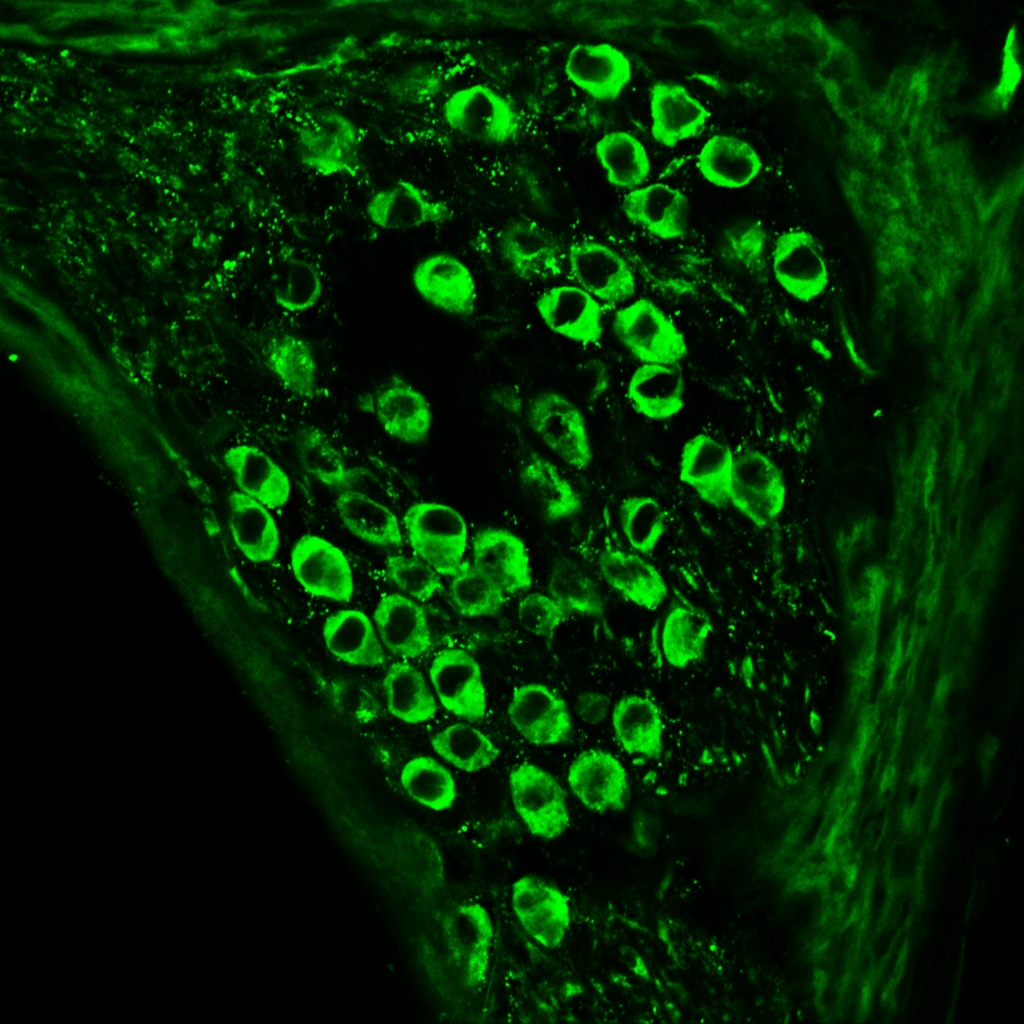

Supplement: Figure 4—source data 1. [file elife-76754-fig4-data1.zip › Figure_4_source_data/Fig.4 C/REST cKO Tuj1 Base.tif]

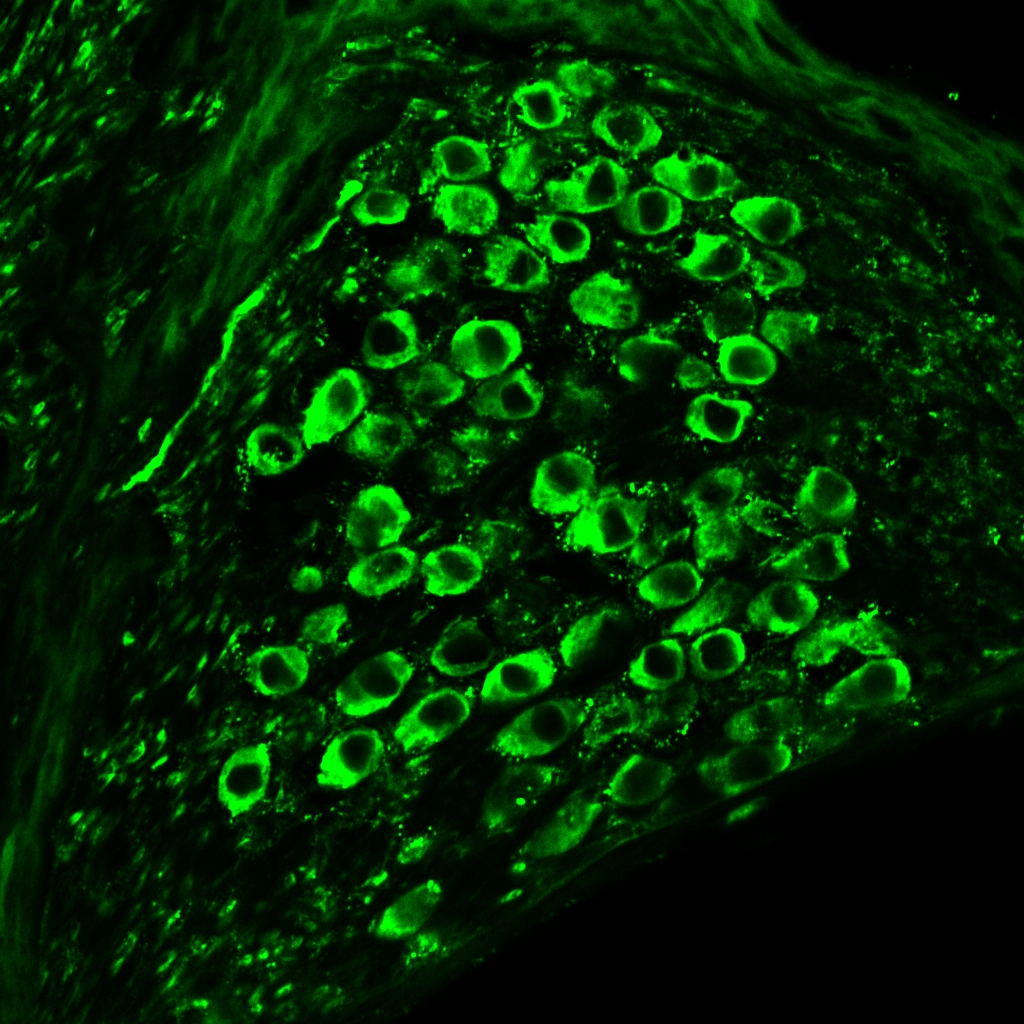

Supplement: Figure 4—source data 1. [file elife-76754-fig4-data1.zip › Figure_4_source_data/Fig.4 C/REST cKO Tuj1 Apex.tif]

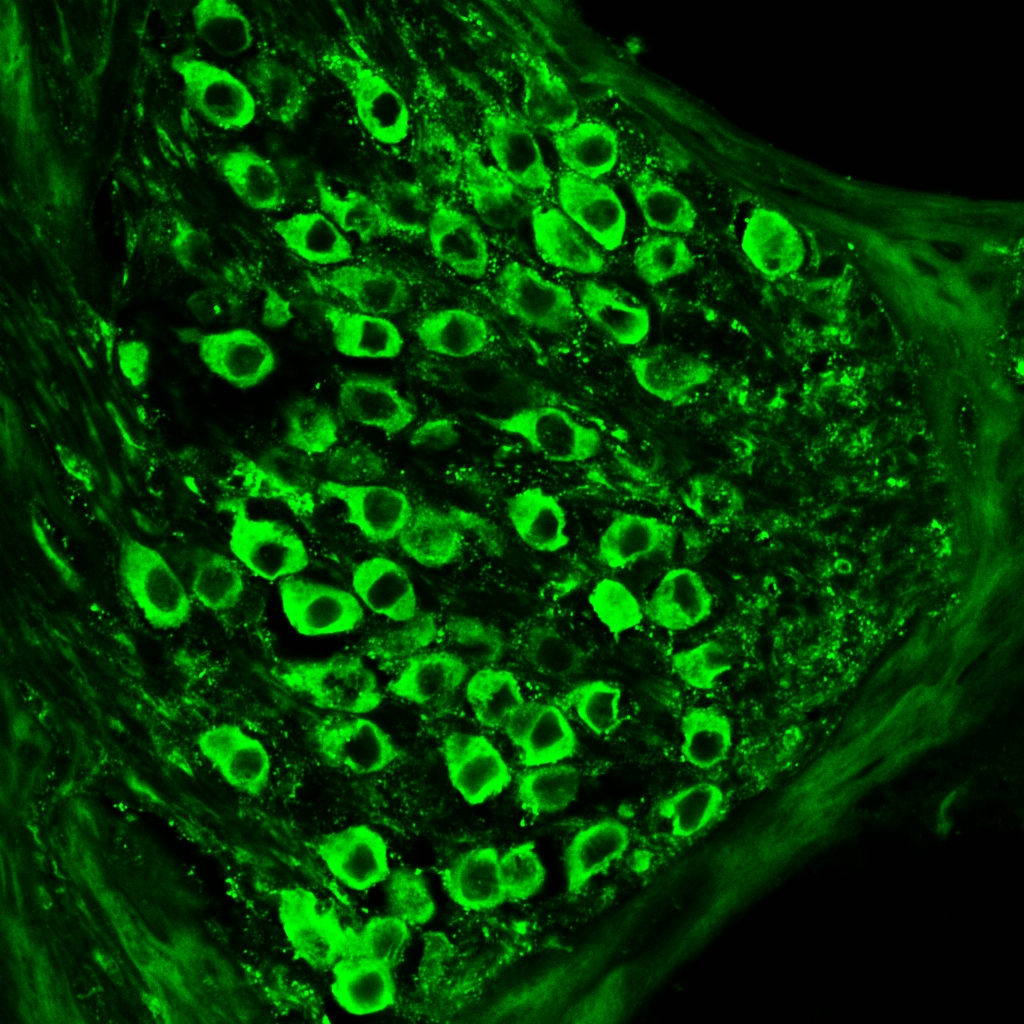

Supplement: Figure 4—source data 1. [file elife-76754-fig4-data1.zip › Figure_4_source_data/Fig.4 C/REST cKO Tuj1 Middle.tif]

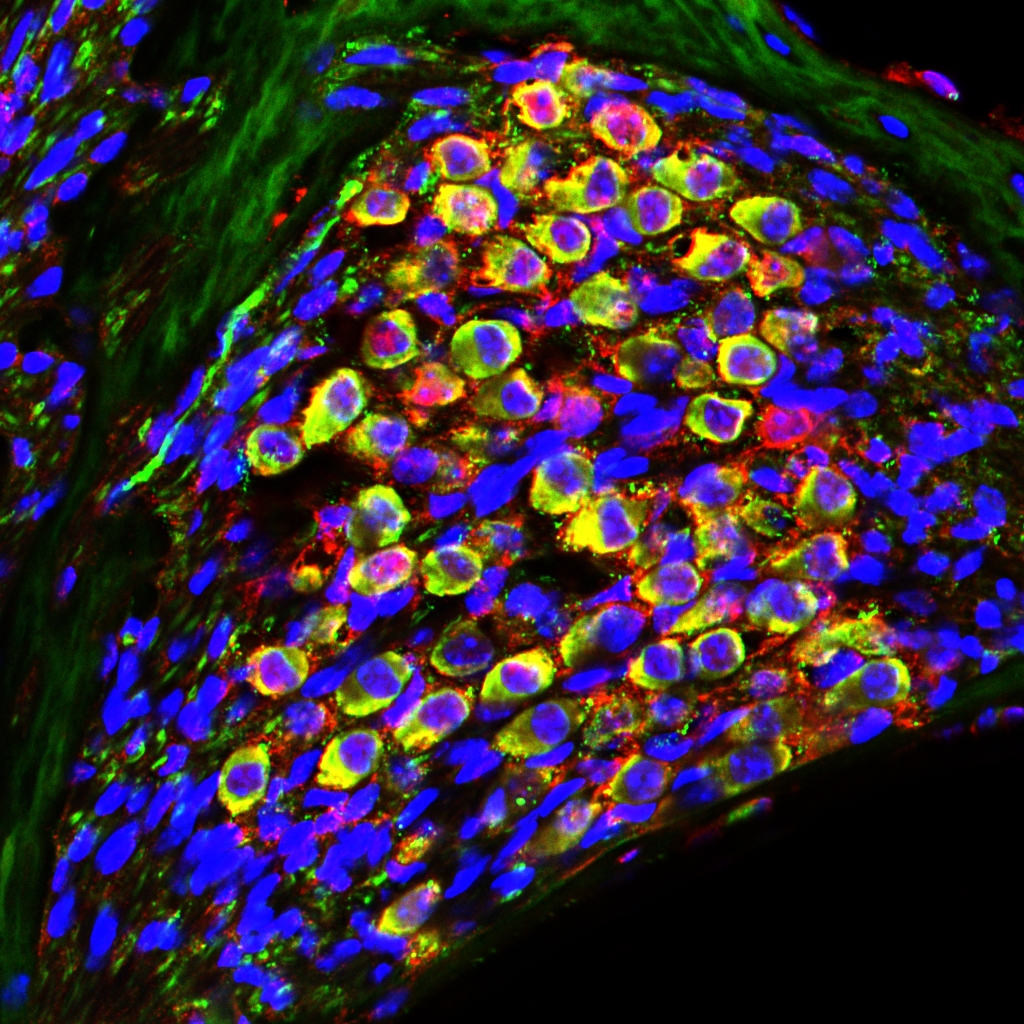

Supplement: Figure 4—source data 1. [file elife-76754-fig4-data1.zip › Figure_4_source_data/Fig.4 C/REST cKO merge Apex.tif]

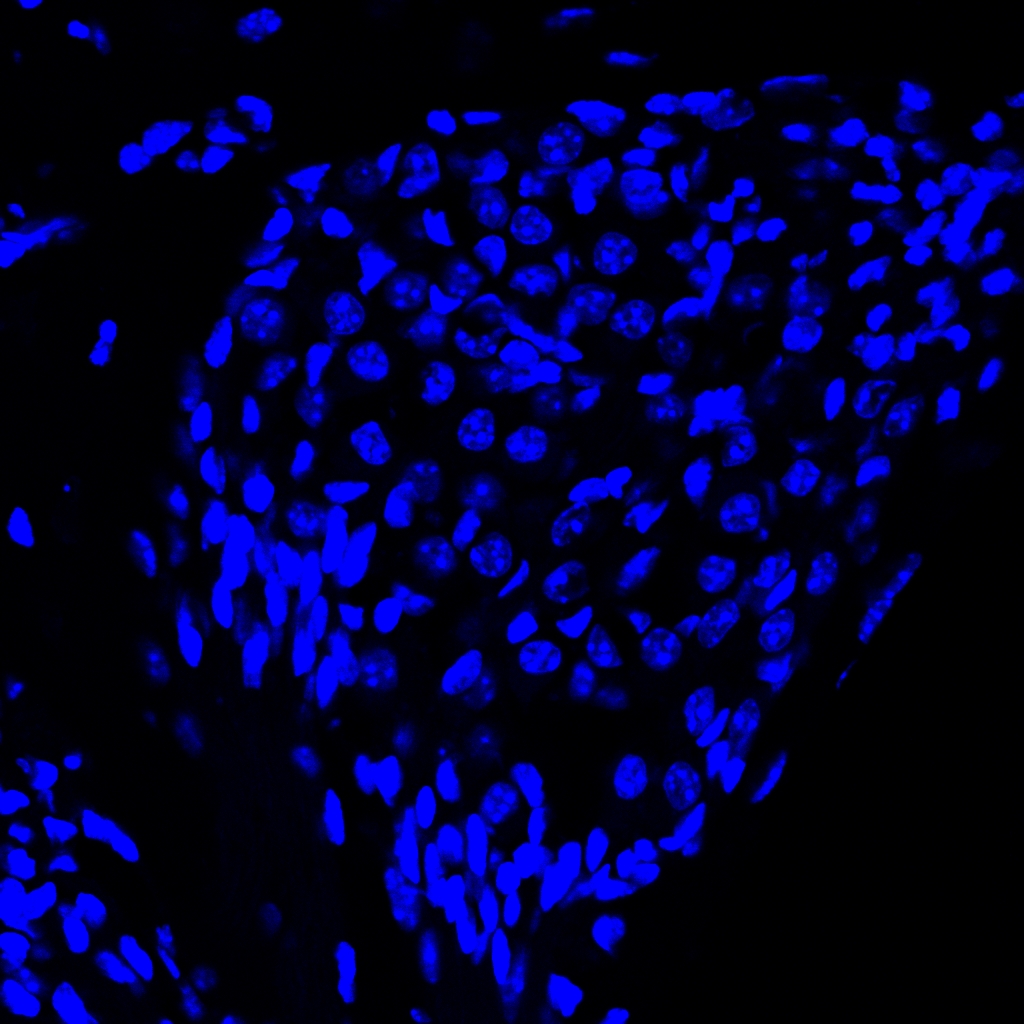

Supplement: Figure 4—source data 1. [file elife-76754-fig4-data1.zip › Figure_4_source_data/Fig.4 C/WT DAPI Base.tif]

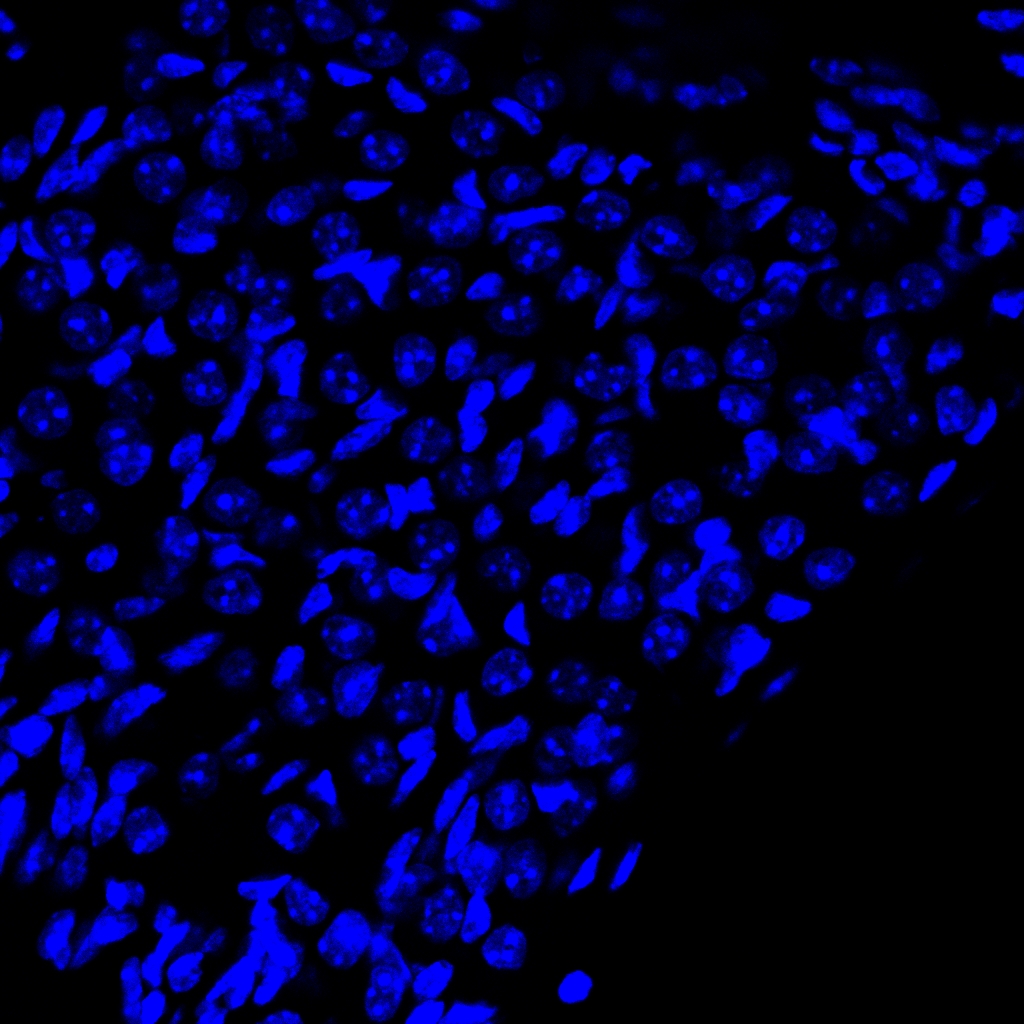

Supplement: Figure 4—source data 1. [file elife-76754-fig4-data1.zip › Figure_4_source_data/Fig.4 C/WT DAPI Apex.tif]

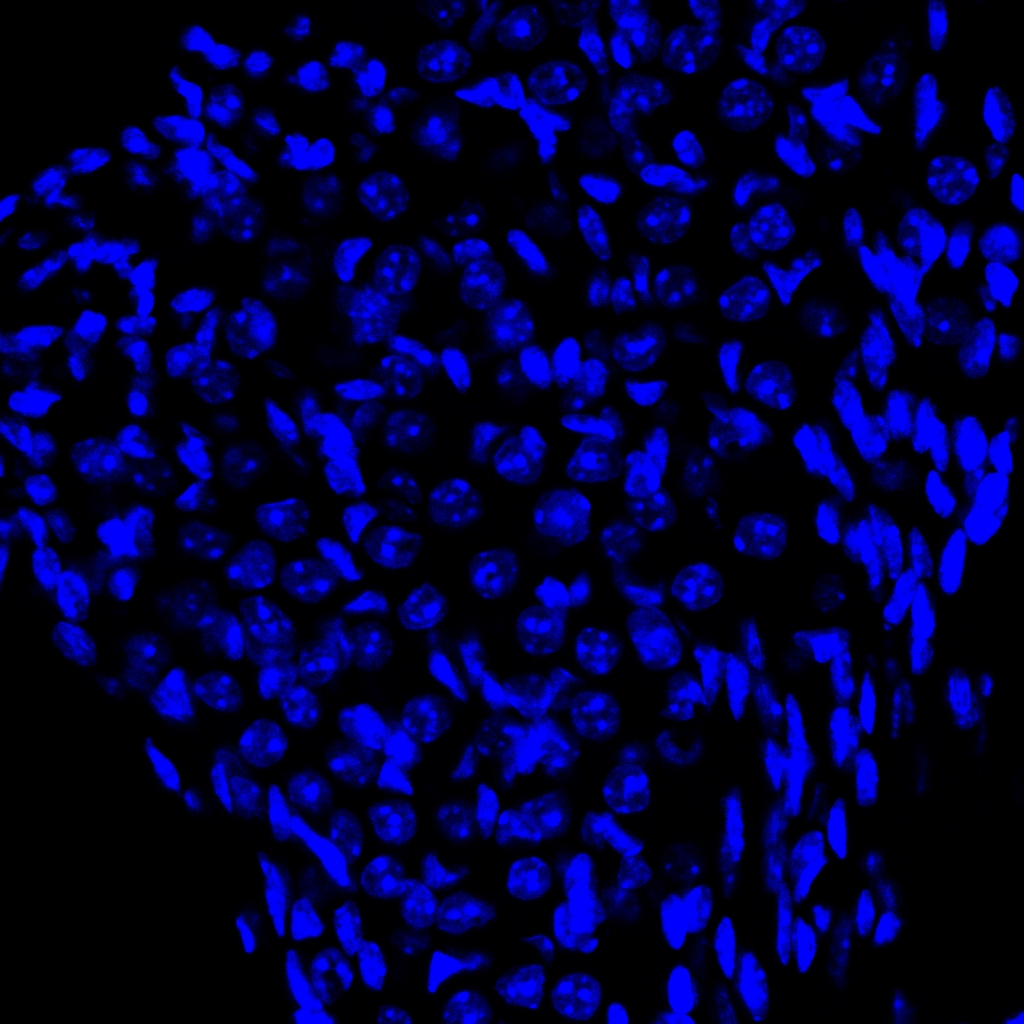

Supplement: Figure 4—source data 1. [file elife-76754-fig4-data1.zip › Figure_4_source_data/Fig.4 C/WT DAPI Middle.tif]

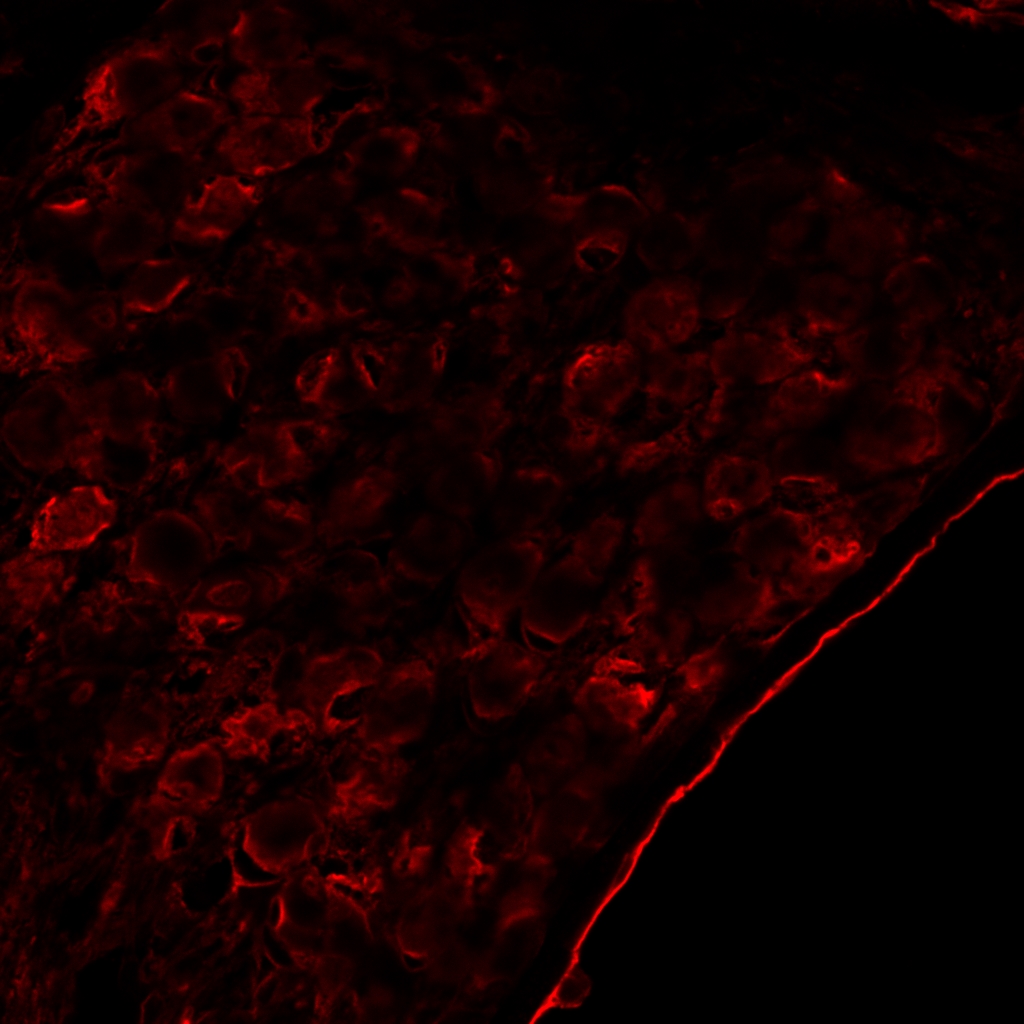

Supplement: Figure 4—source data 1. [file elife-76754-fig4-data1.zip › Figure_4_source_data/Fig.4 C/WT Kv7.4 Apex.tif]

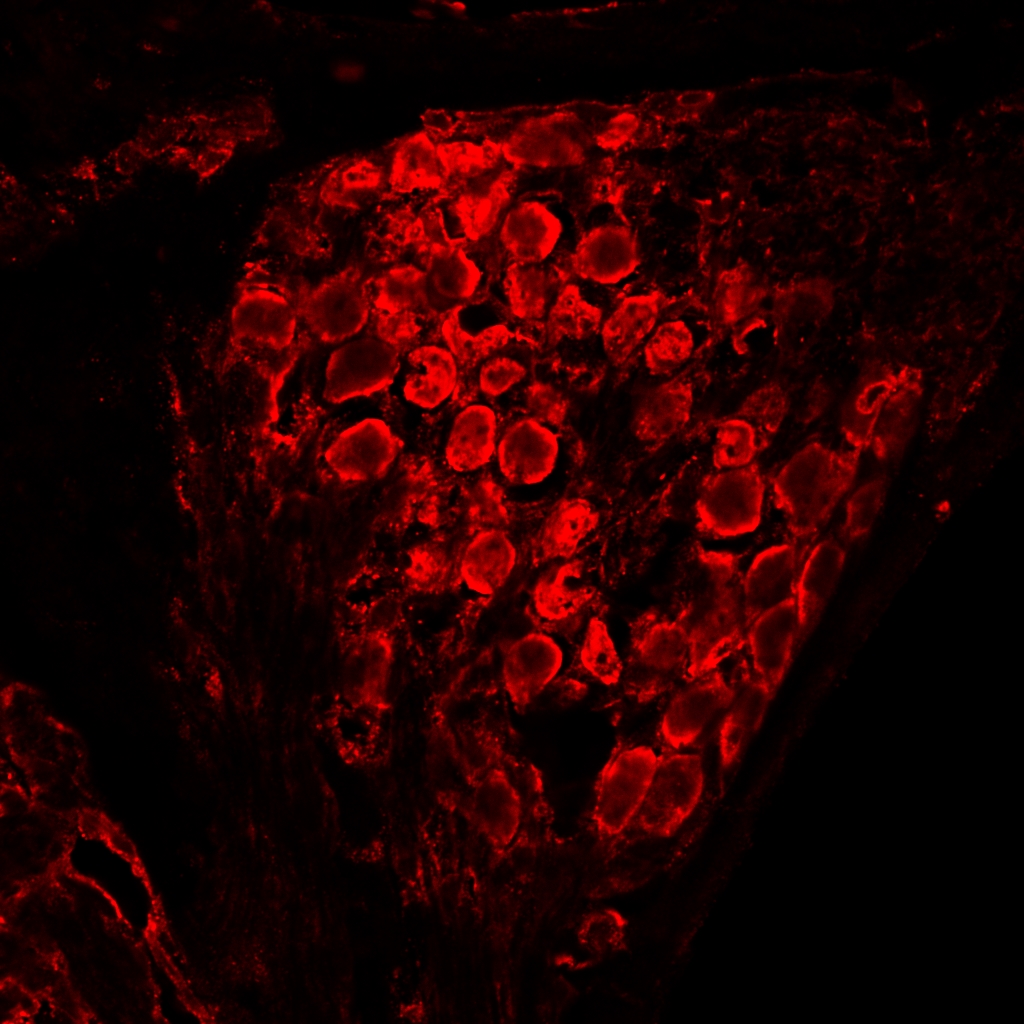

Supplement: Figure 4—source data 1. [file elife-76754-fig4-data1.zip › Figure_4_source_data/Fig.4 C/WT Kv7.4 Base.tif]

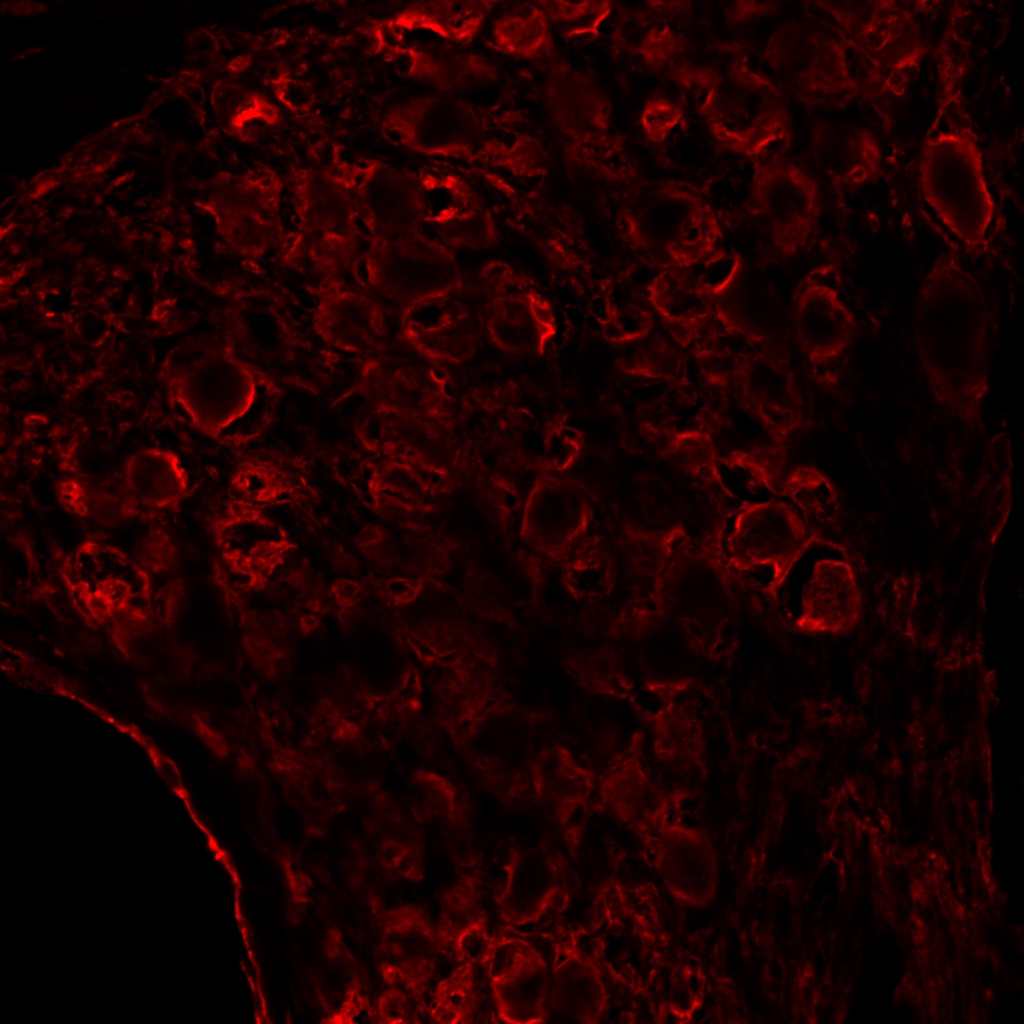

Supplement: Figure 4—source data 1. [file elife-76754-fig4-data1.zip › Figure_4_source_data/Fig.4 C/WT Kv7.4 Middle.tif]

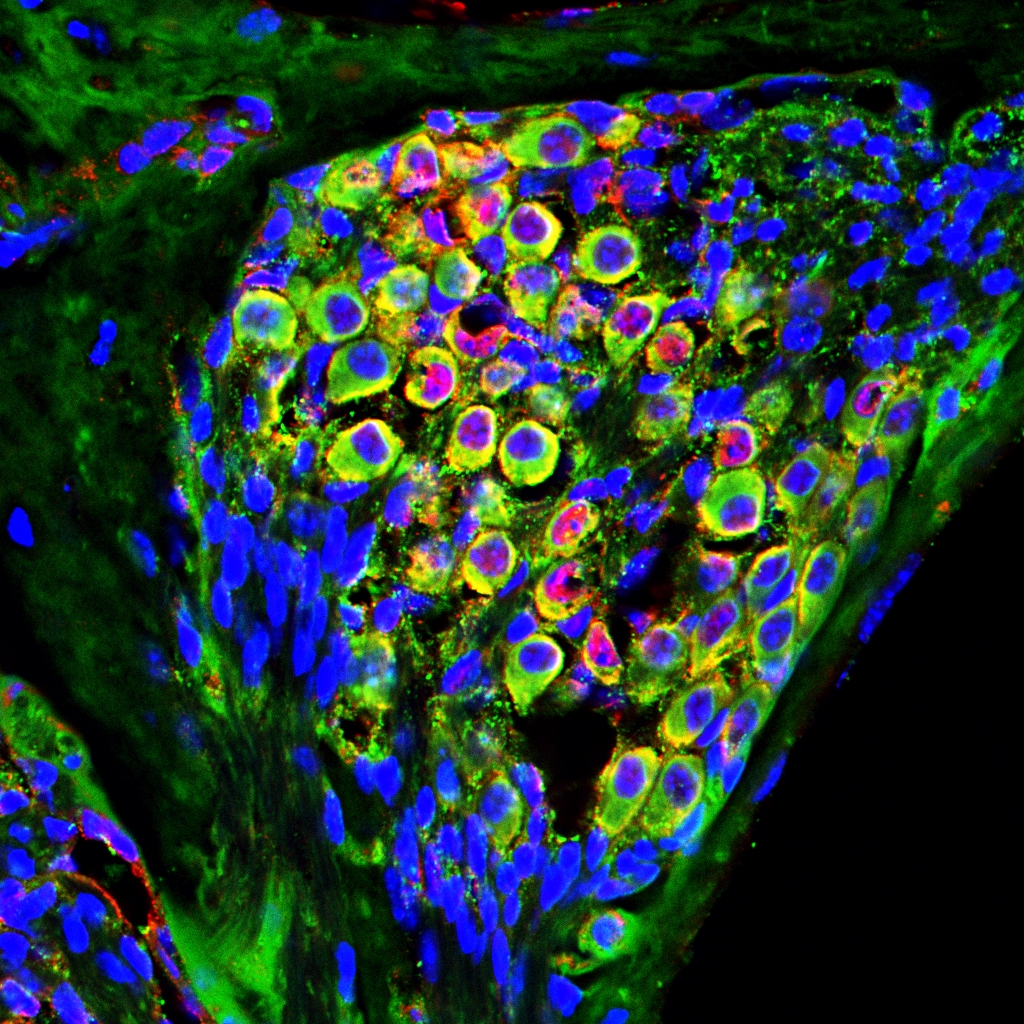

Supplement: Figure 4—source data 1. [file elife-76754-fig4-data1.zip › Figure_4_source_data/Fig.4 C/WT Merge Base.tif]

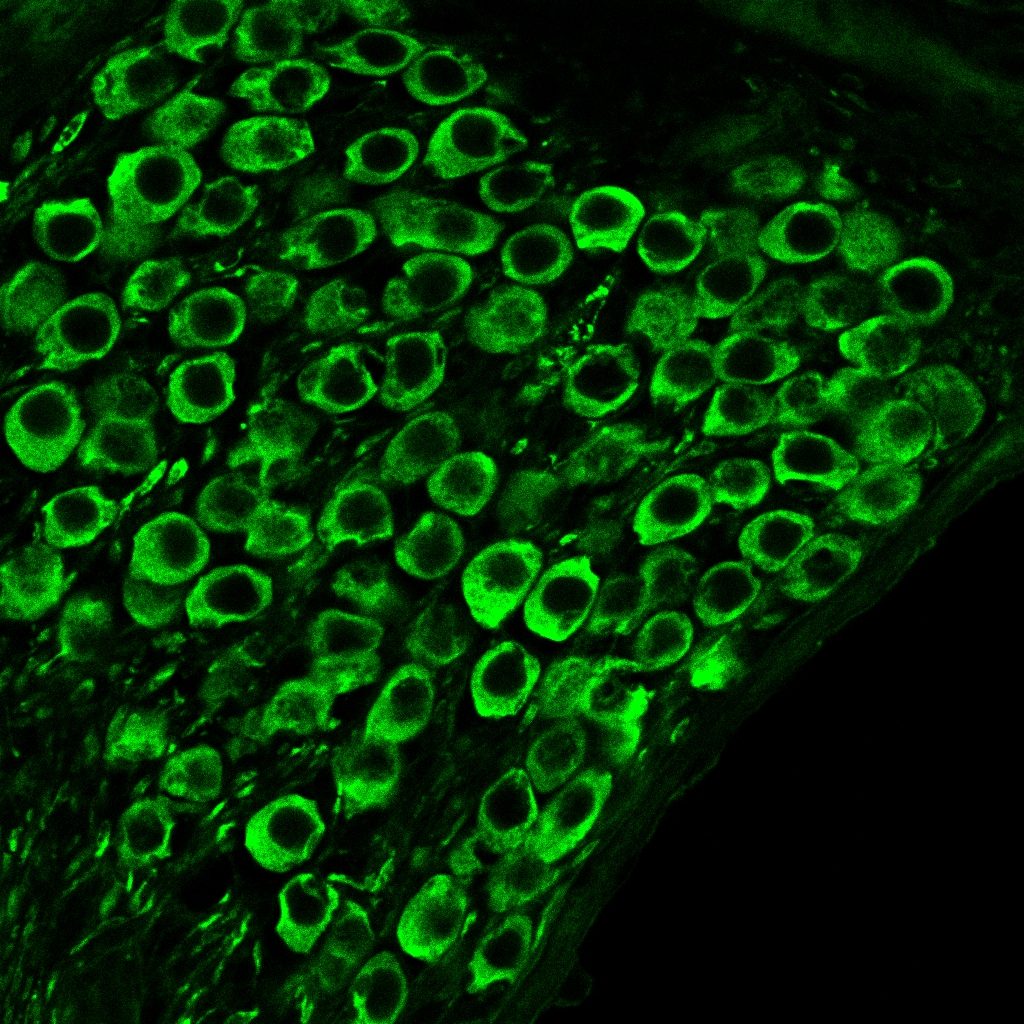

Supplement: Figure 4—source data 1. [file elife-76754-fig4-data1.zip › Figure_4_source_data/Fig.4 C/WT Tuj1 Apex.tif]

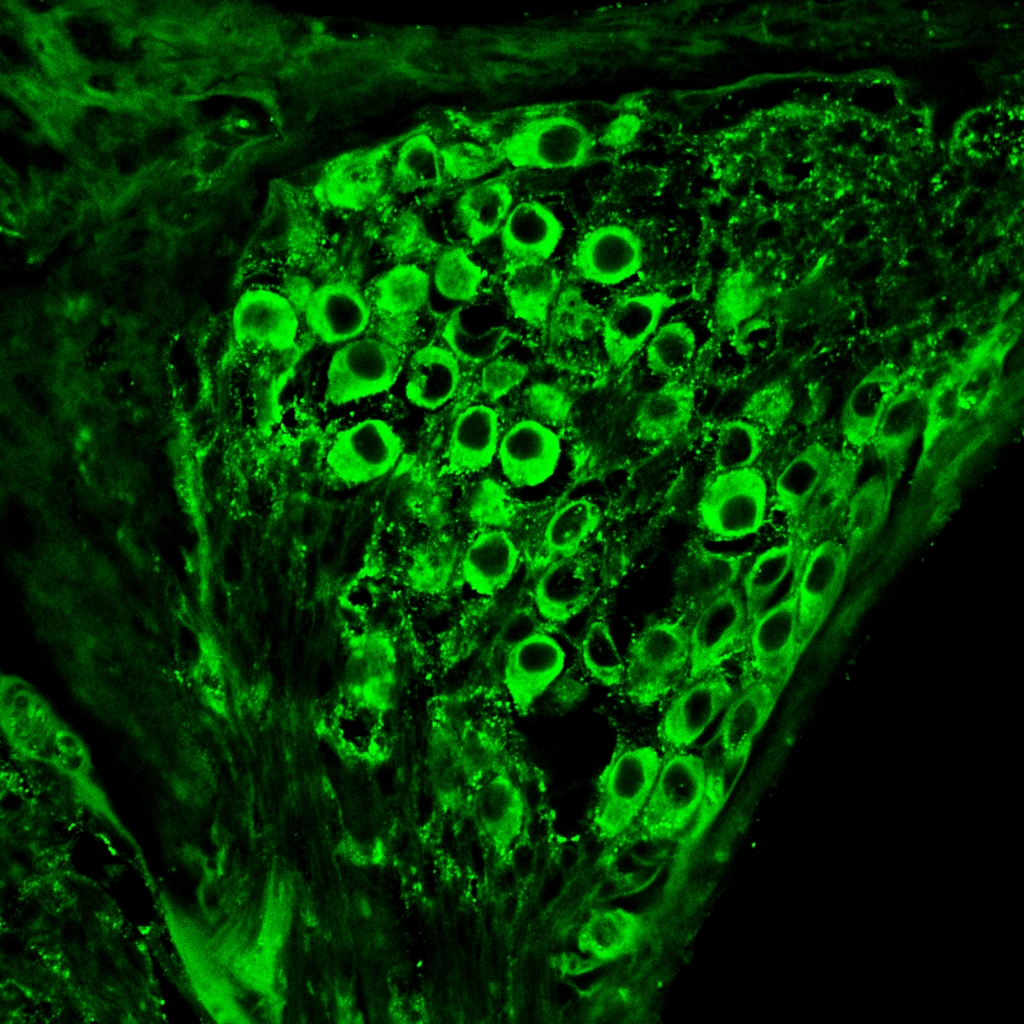

Supplement: Figure 4—source data 1. [file elife-76754-fig4-data1.zip › Figure_4_source_data/Fig.4 C/WT Tuj1 Base.tif]

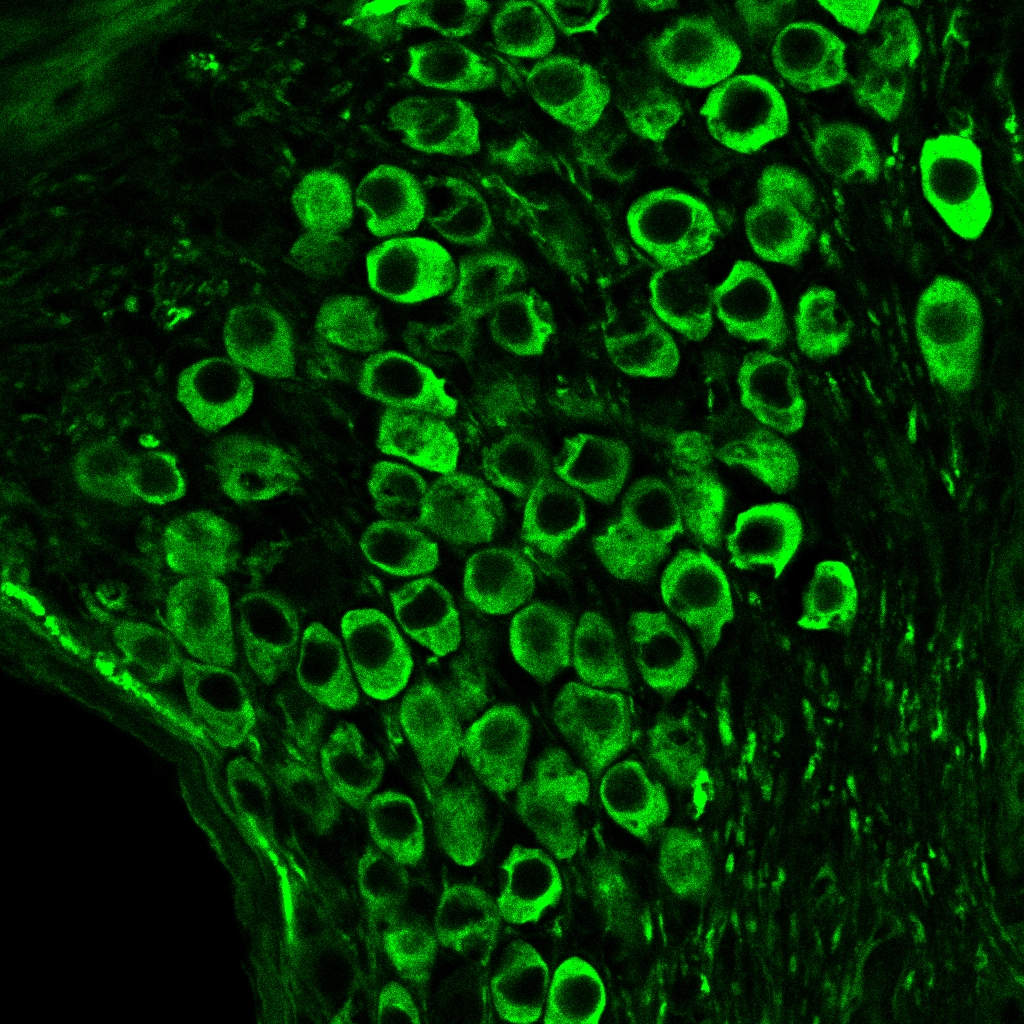

Supplement: Figure 4—source data 1. [file elife-76754-fig4-data1.zip › Figure_4_source_data/Fig.4 C/WT Tuj1 Middle.tif]

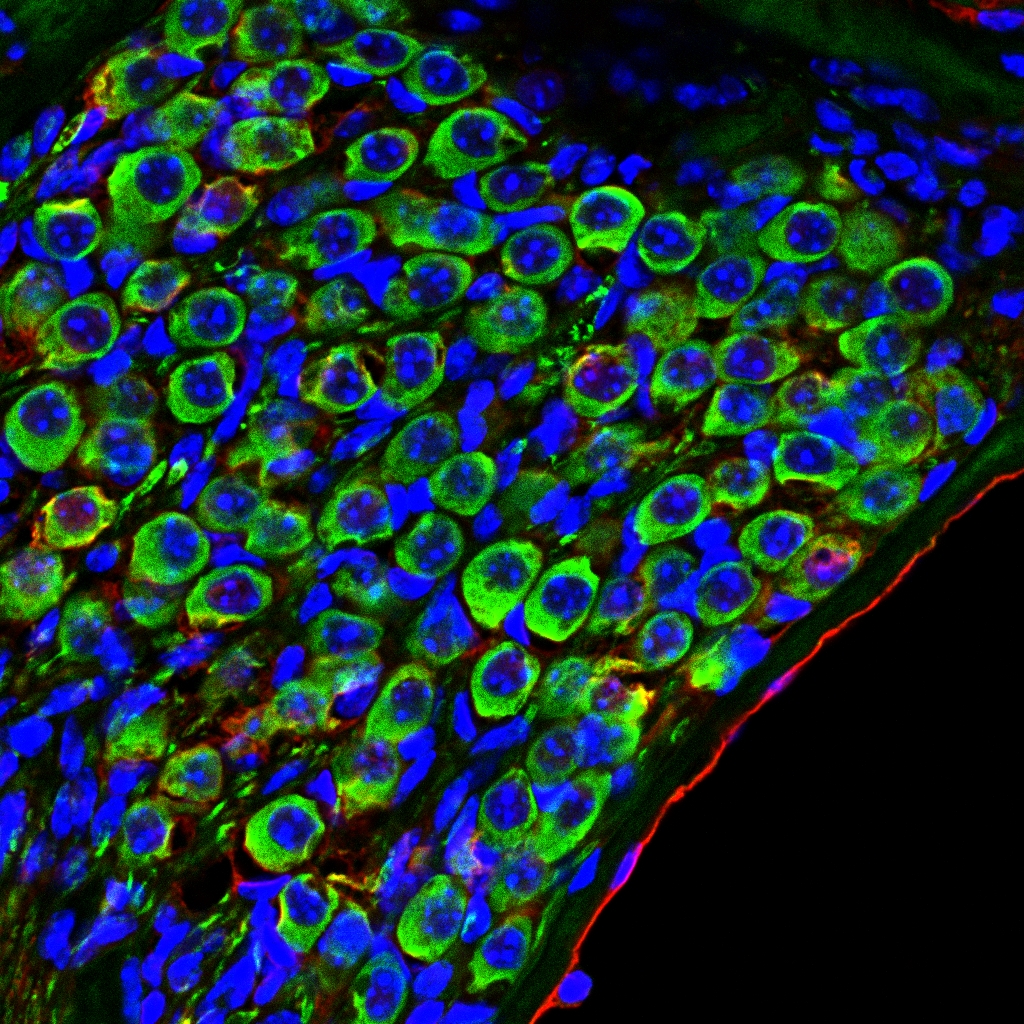

Supplement: Figure 4—source data 1. [file elife-76754-fig4-data1.zip › Figure_4_source_data/Fig.4 C/WT merge Apex.tif]

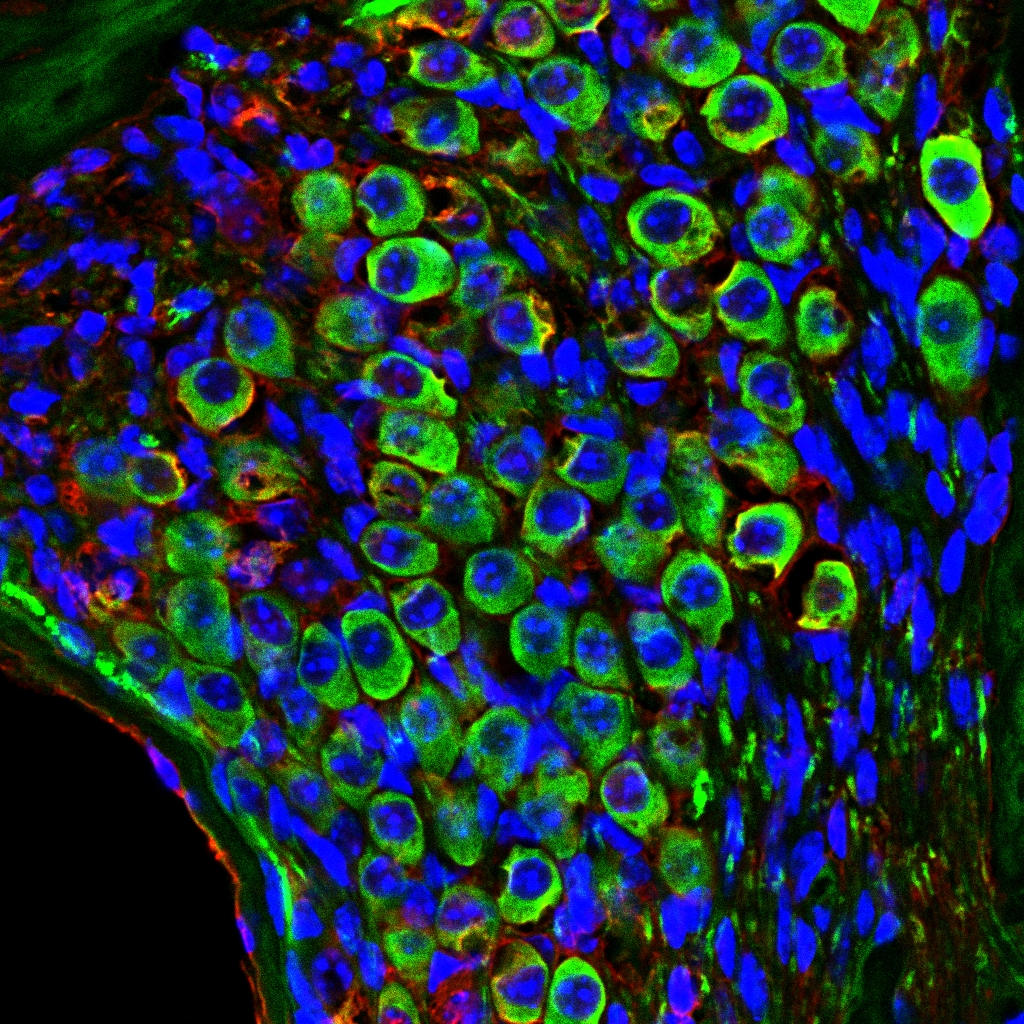

Supplement: Figure 4—source data 1. [file elife-76754-fig4-data1.zip › Figure_4_source_data/Fig.4 C/WT merge Middle.tif]

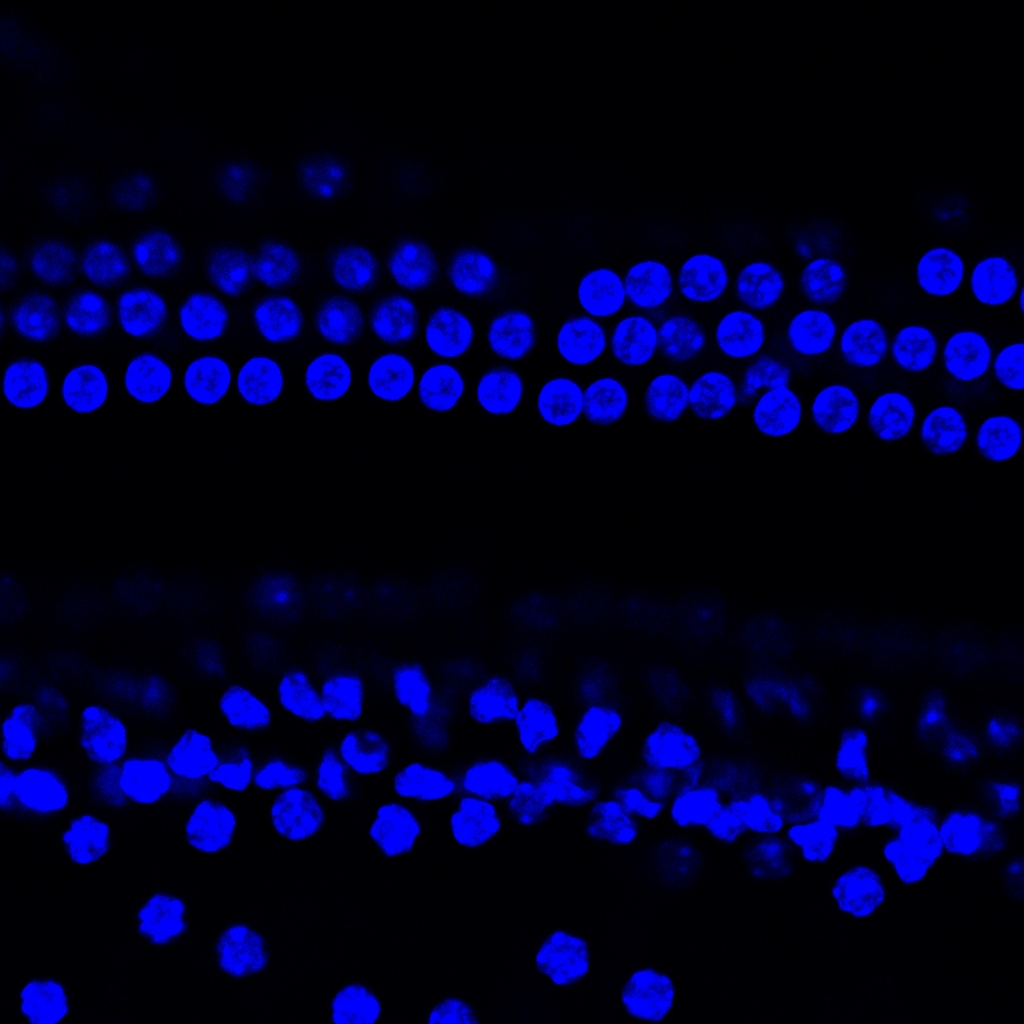

Supplement: Figure 4—source data 1. [file elife-76754-fig4-data1.zip › Figure_4_source_data/Fig.4 G/Rest cKO DAPI Apex.tif]

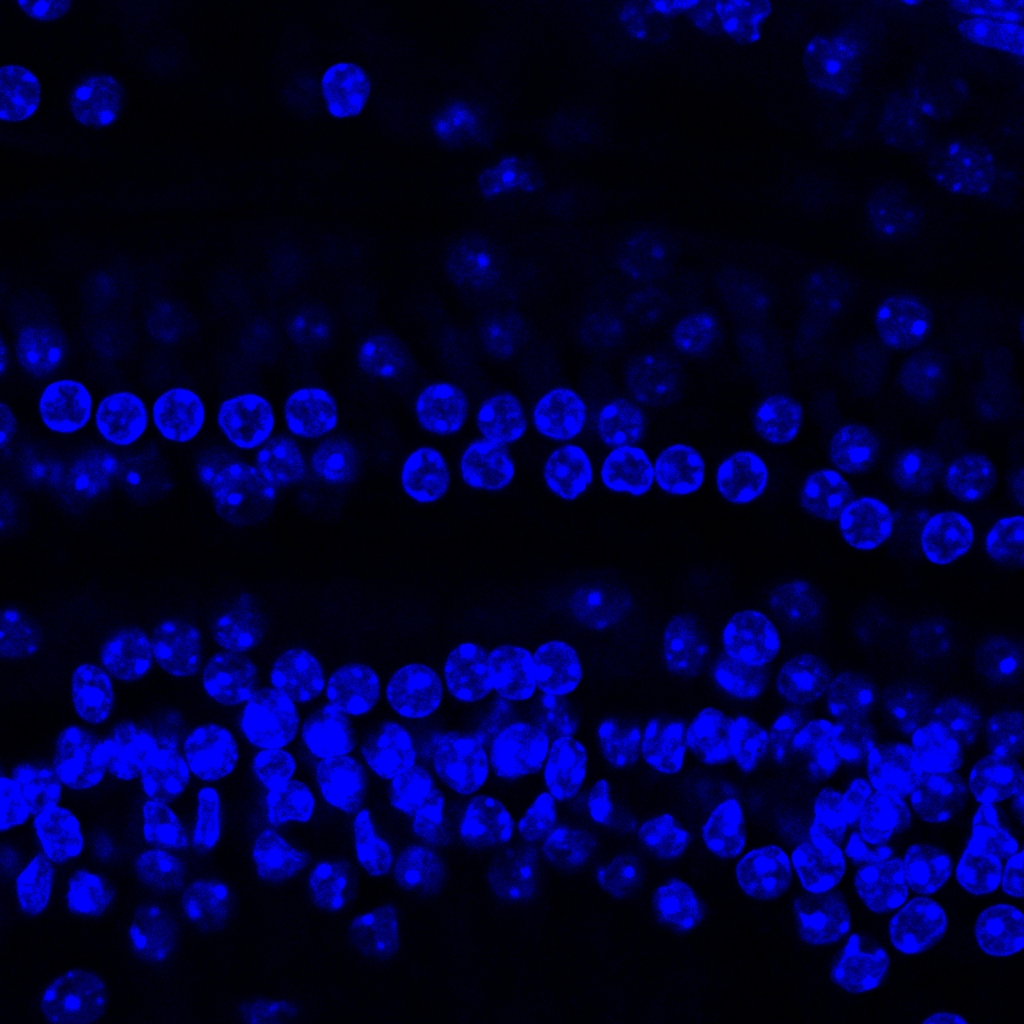

Supplement: Figure 4—source data 1. [file elife-76754-fig4-data1.zip › Figure_4_source_data/Fig.4 G/Rest cKO DAPI Base.tif]

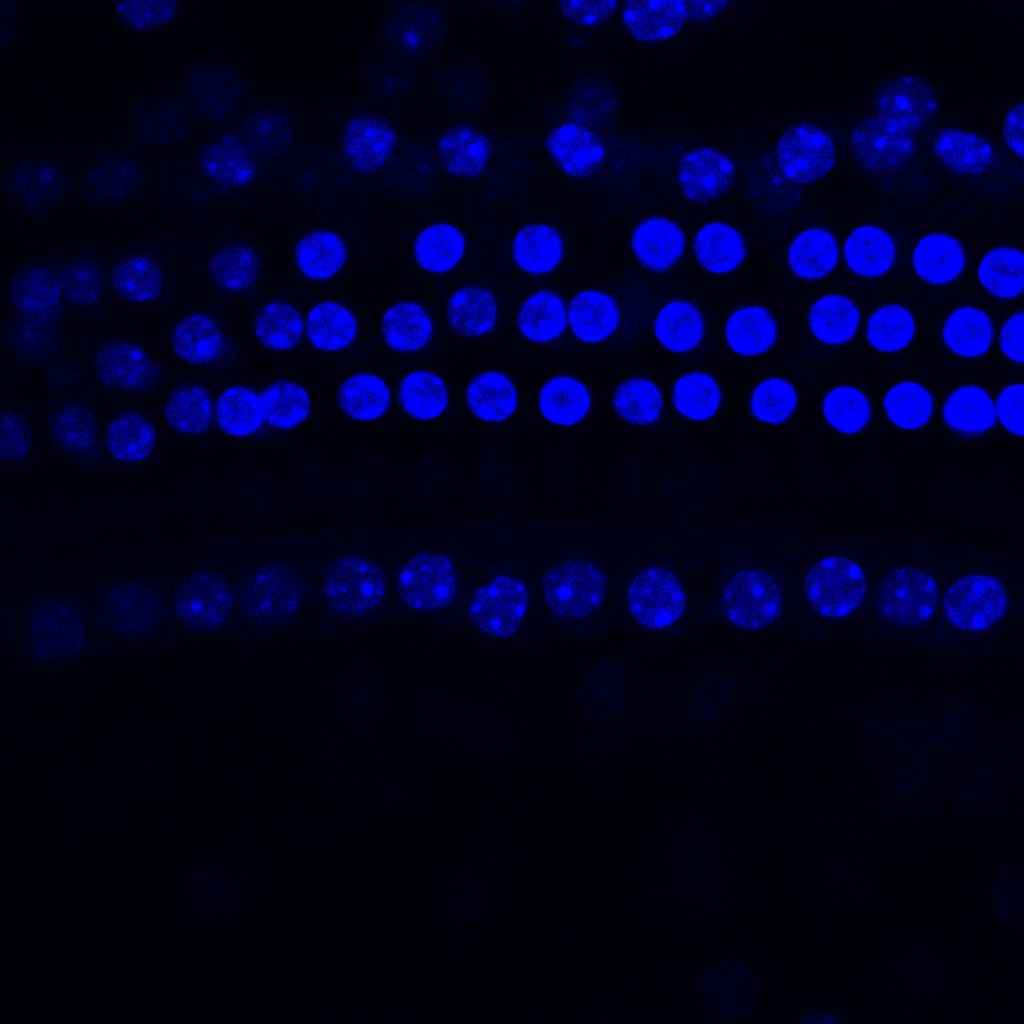

Supplement: Figure 4—source data 1. [file elife-76754-fig4-data1.zip › Figure_4_source_data/Fig.4 G/Rest cKO DAPI Middle.tif]

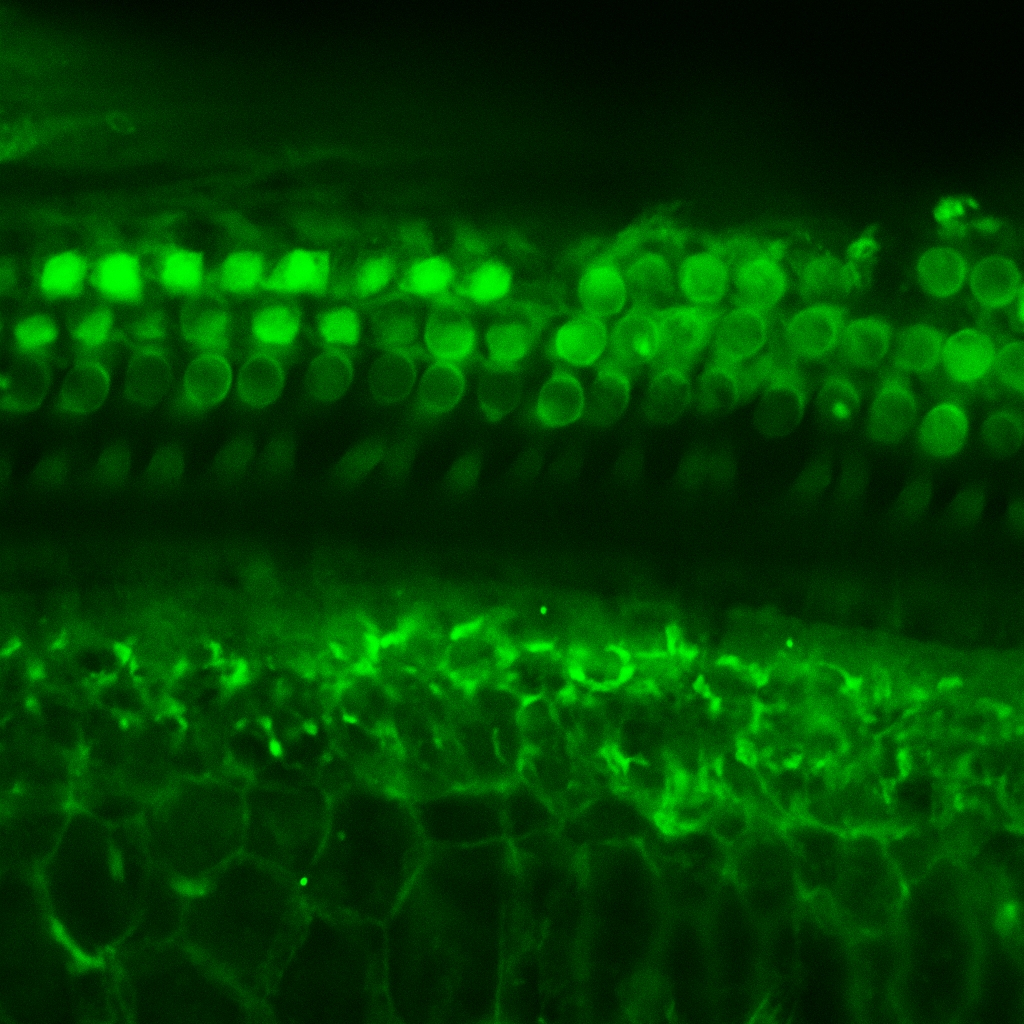

Supplement: Figure 4—source data 1. [file elife-76754-fig4-data1.zip › Figure_4_source_data/Fig.4 G/Rest cKO Kv7.4 Apex.tif]

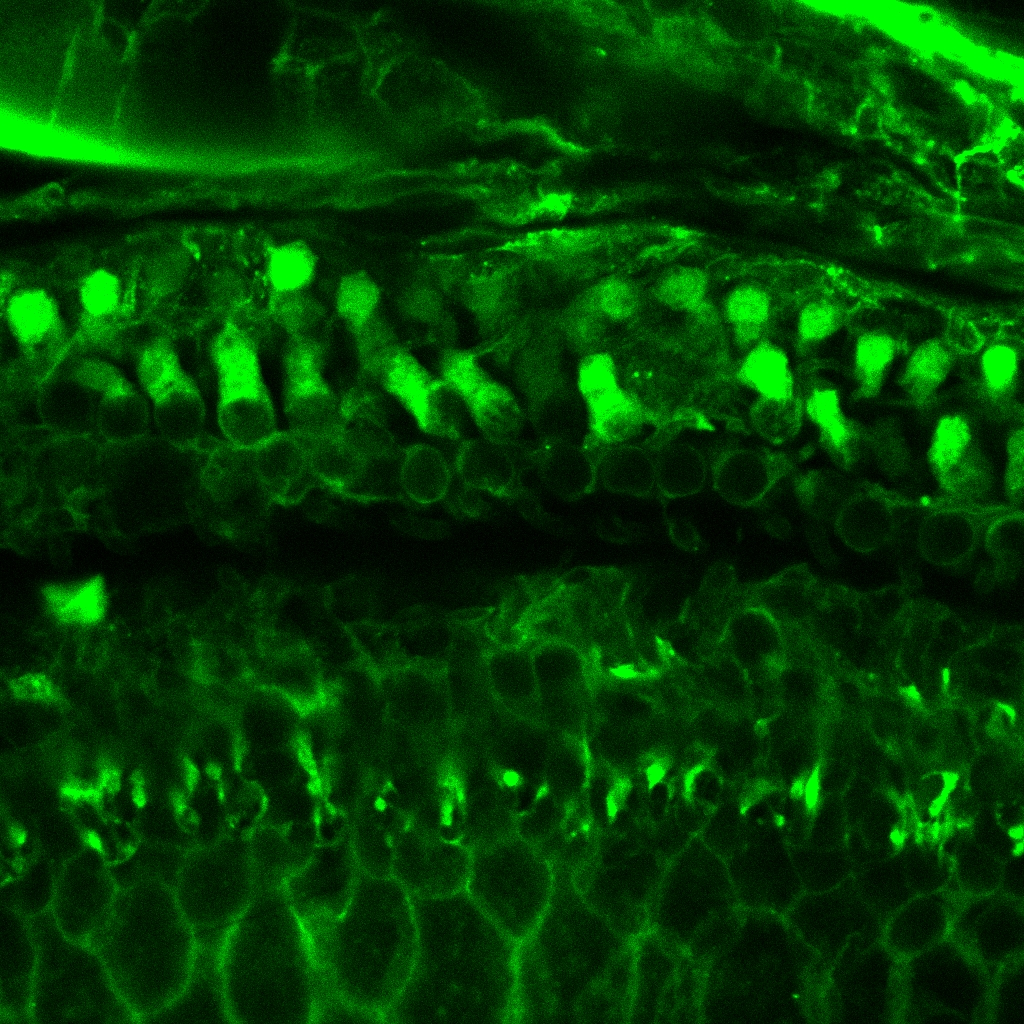

Supplement: Figure 4—source data 1. [file elife-76754-fig4-data1.zip › Figure_4_source_data/Fig.4 G/Rest cKO Kv7.4 Base.tif]

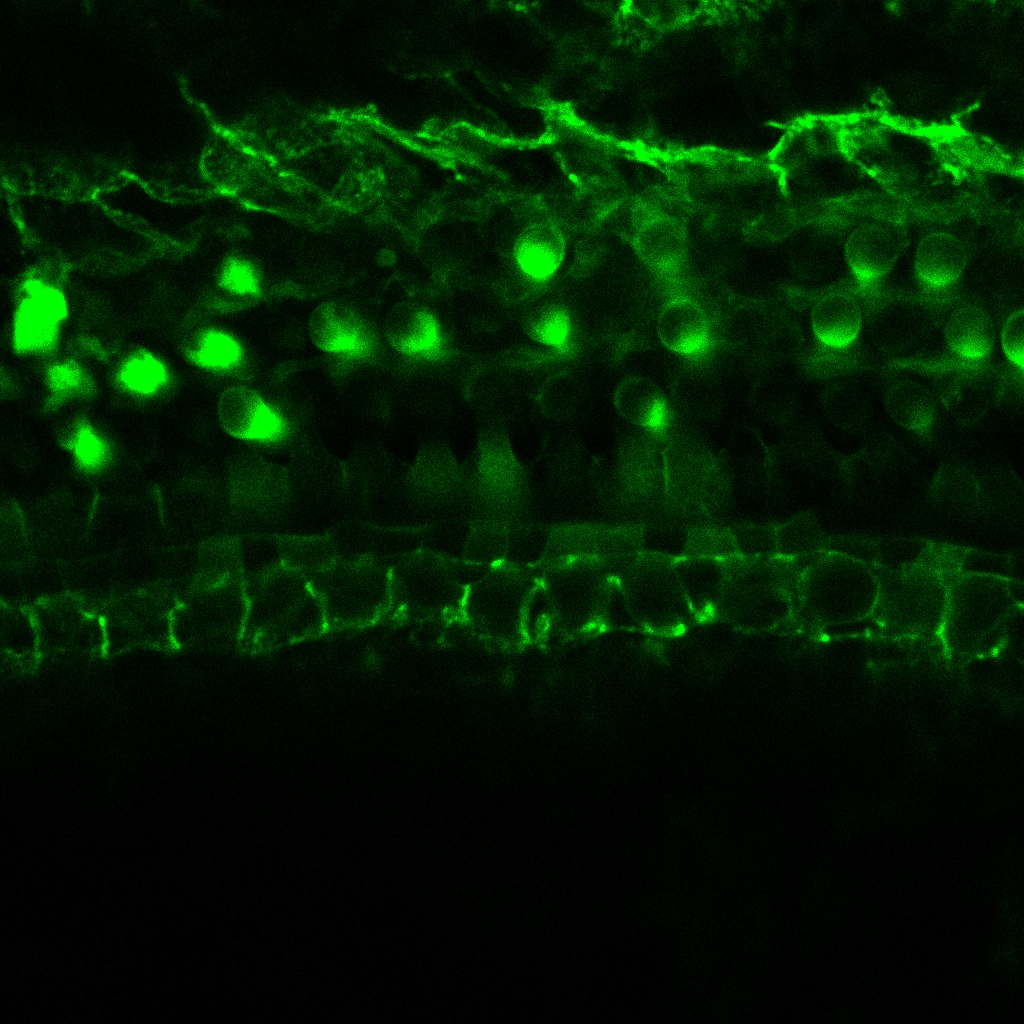

Supplement: Figure 4—source data 1. [file elife-76754-fig4-data1.zip › Figure_4_source_data/Fig.4 G/Rest cKO Kv7.4 Middle.tif]

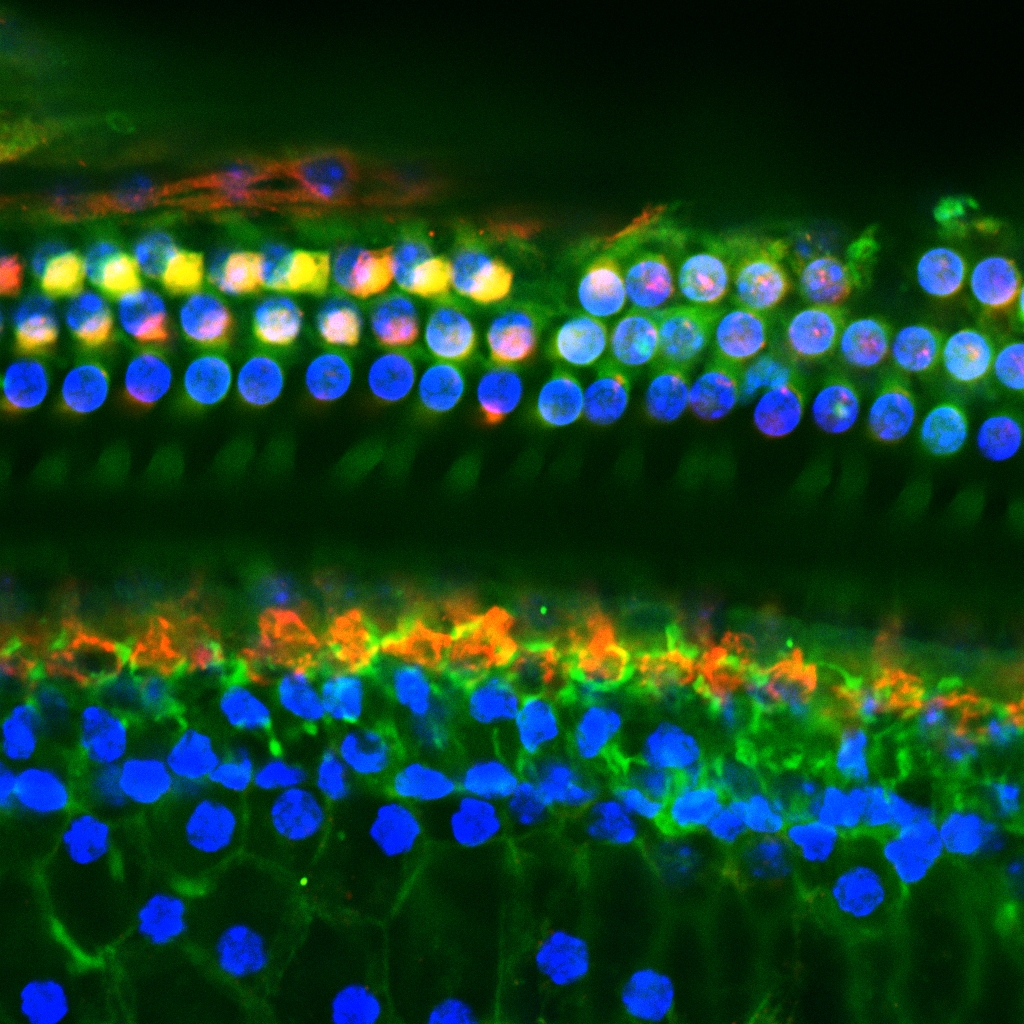

Supplement: Figure 4—source data 1. [file elife-76754-fig4-data1.zip › Figure_4_source_data/Fig.4 G/Rest cKO Merge Apex.tif]

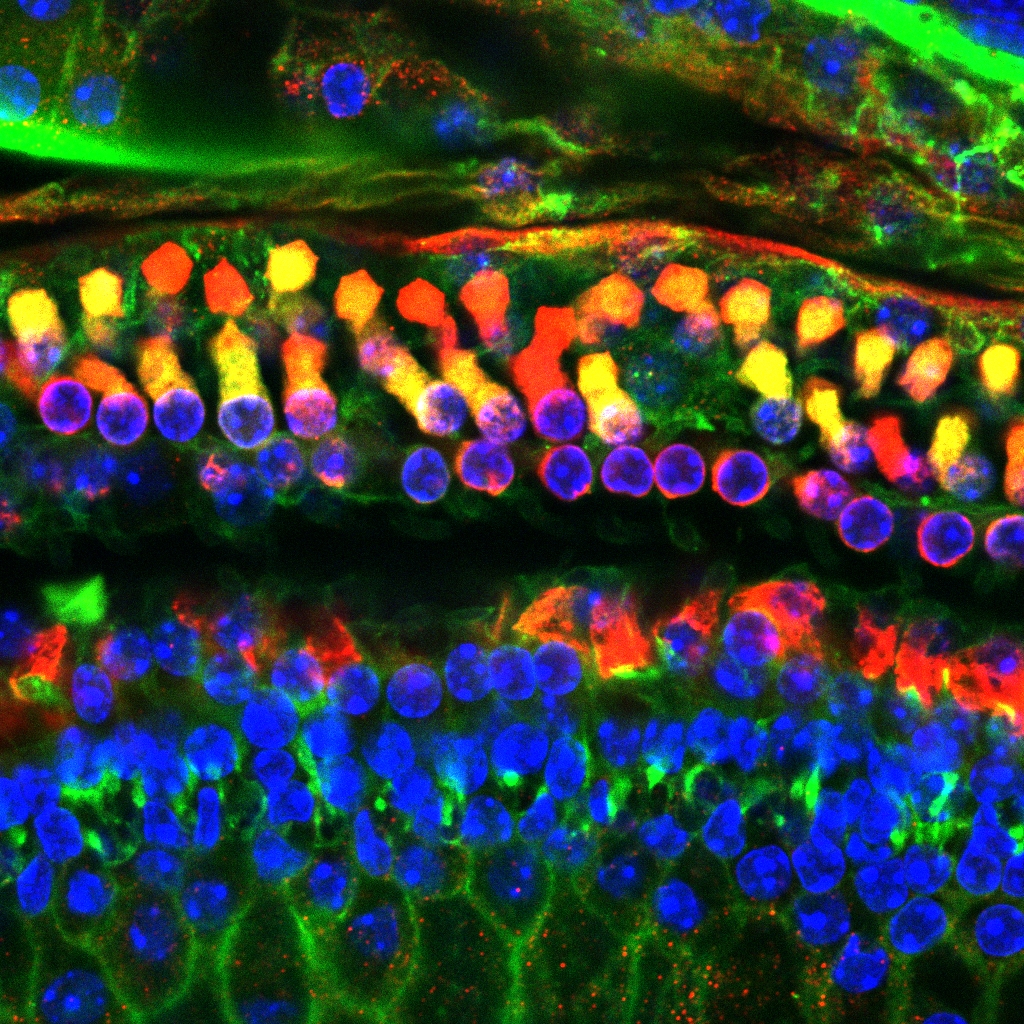

Supplement: Figure 4—source data 1. [file elife-76754-fig4-data1.zip › Figure_4_source_data/Fig.4 G/Rest cKO Merge Base.tif]

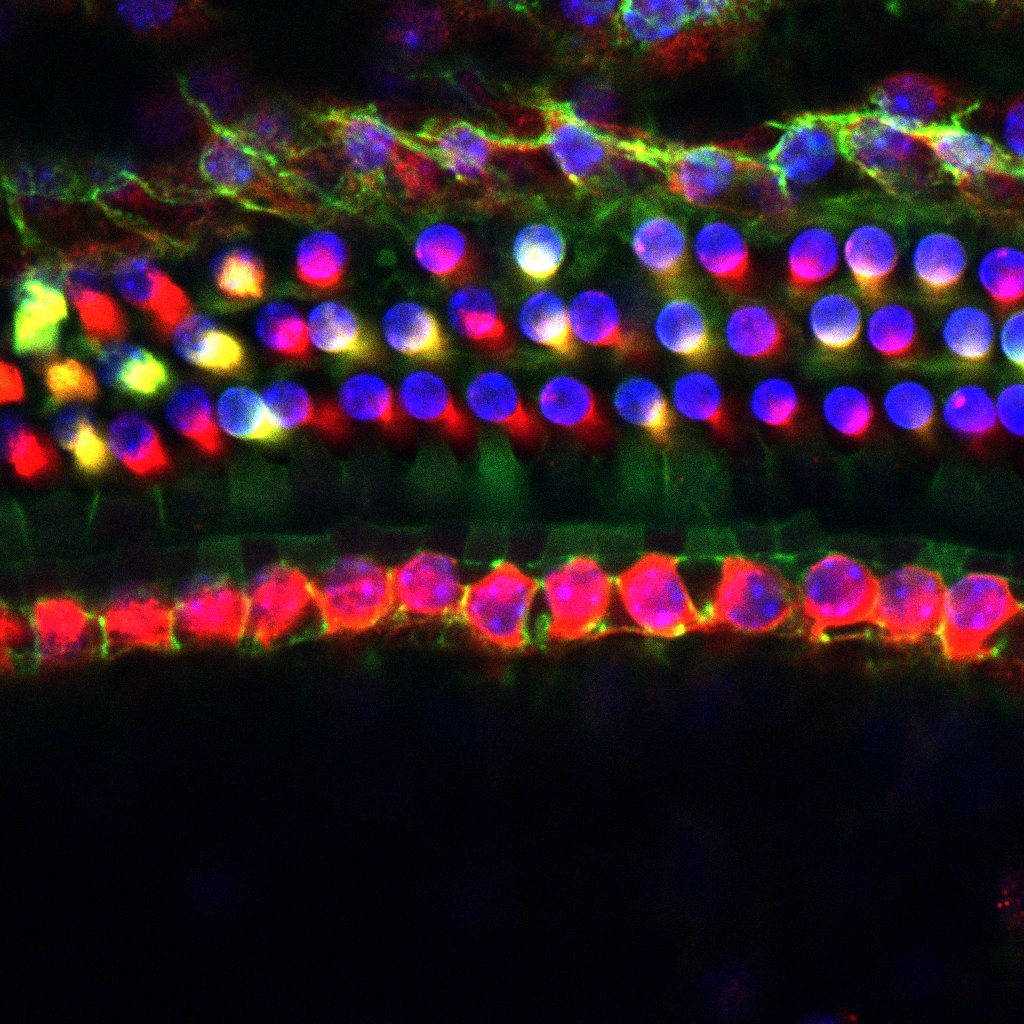

Supplement: Figure 4—source data 1. [file elife-76754-fig4-data1.zip › Figure_4_source_data/Fig.4 G/Rest cKO Merge Middle.tif]

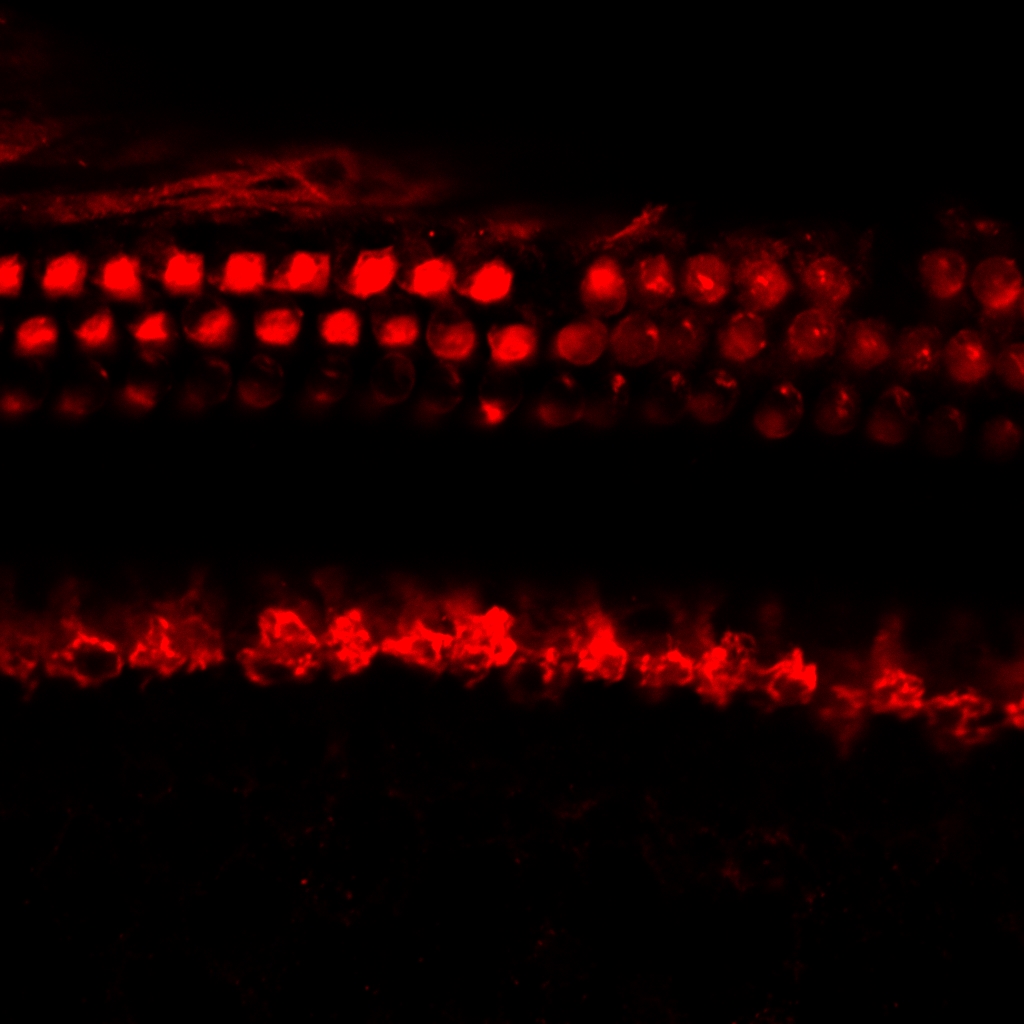

Supplement: Figure 4—source data 1. [file elife-76754-fig4-data1.zip › Figure_4_source_data/Fig.4 G/Rest cKO myo7a Apex.tif]

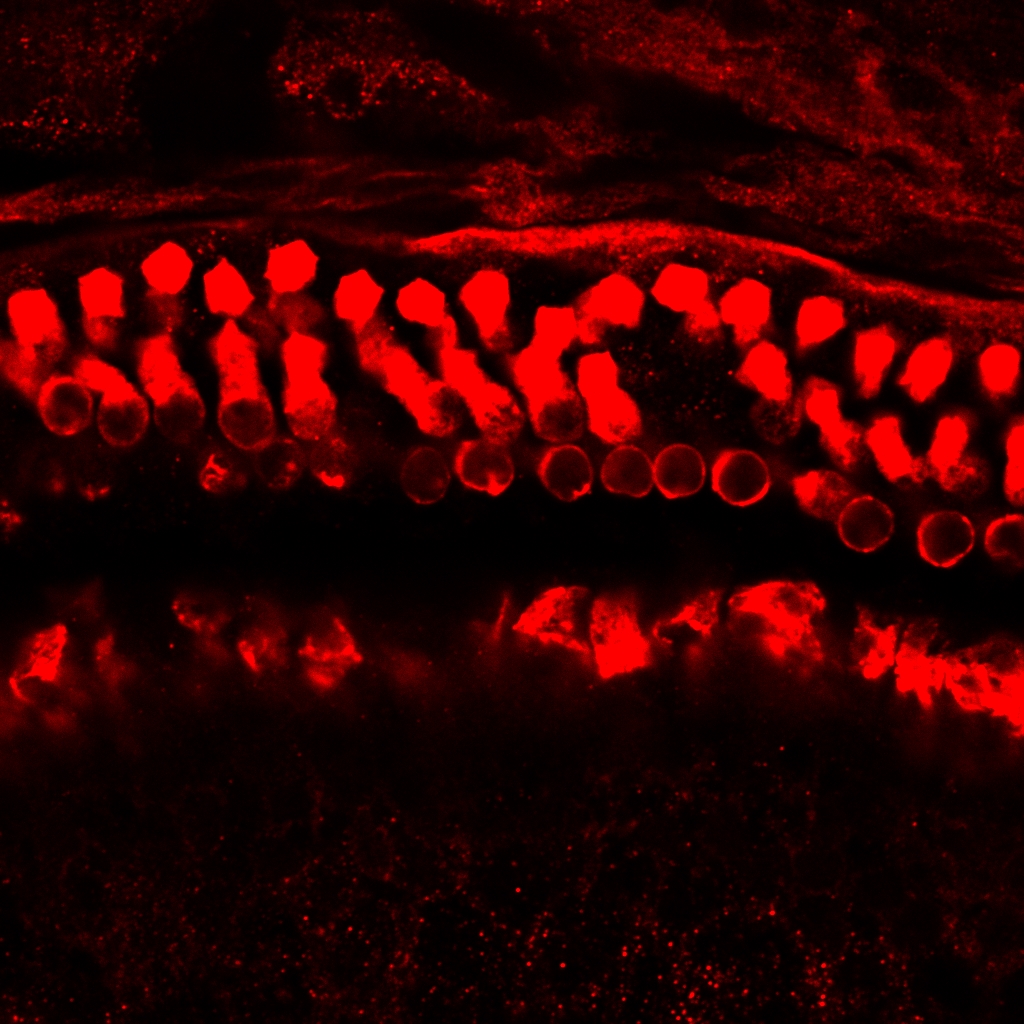

Supplement: Figure 4—source data 1. [file elife-76754-fig4-data1.zip › Figure_4_source_data/Fig.4 G/Rest cKO myo7a Base.tif]

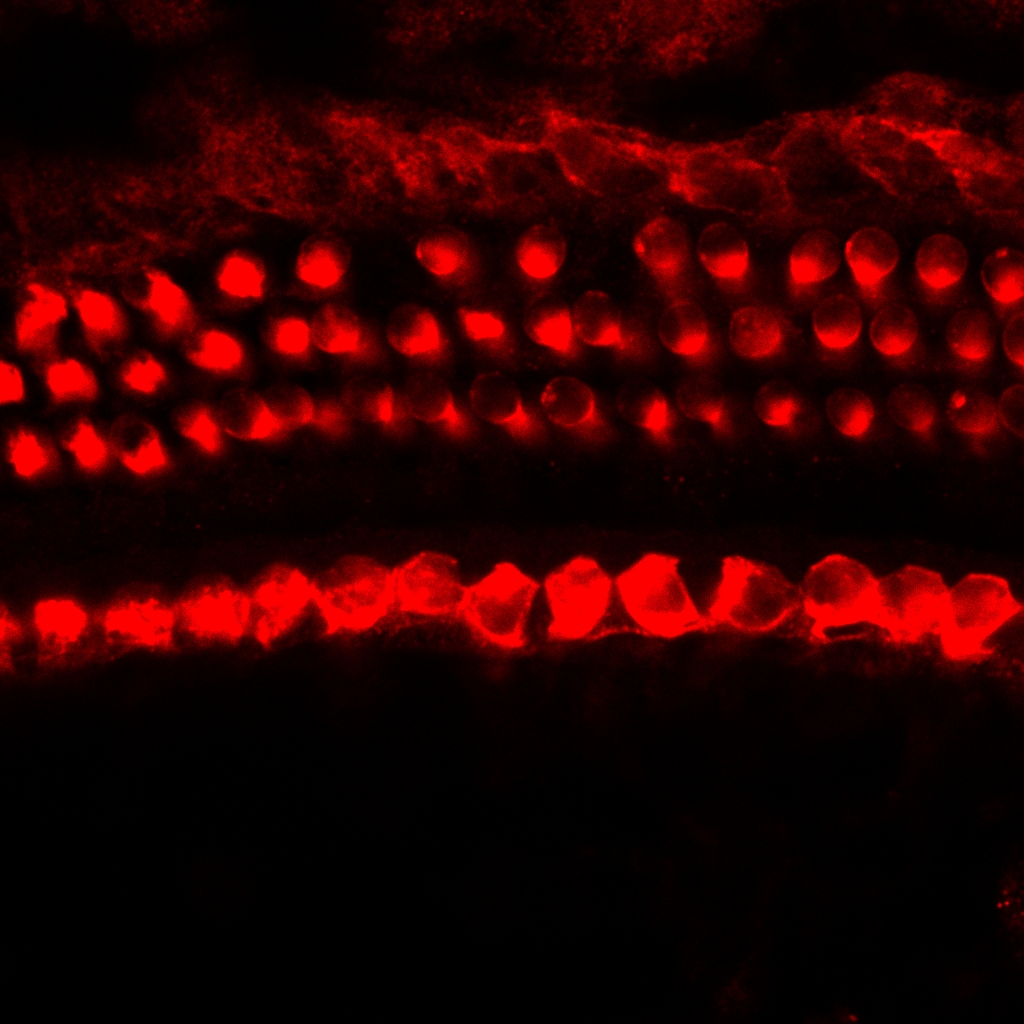

Supplement: Figure 4—source data 1. [file elife-76754-fig4-data1.zip › Figure_4_source_data/Fig.4 G/Rest cKO myo7a Middle.tif]

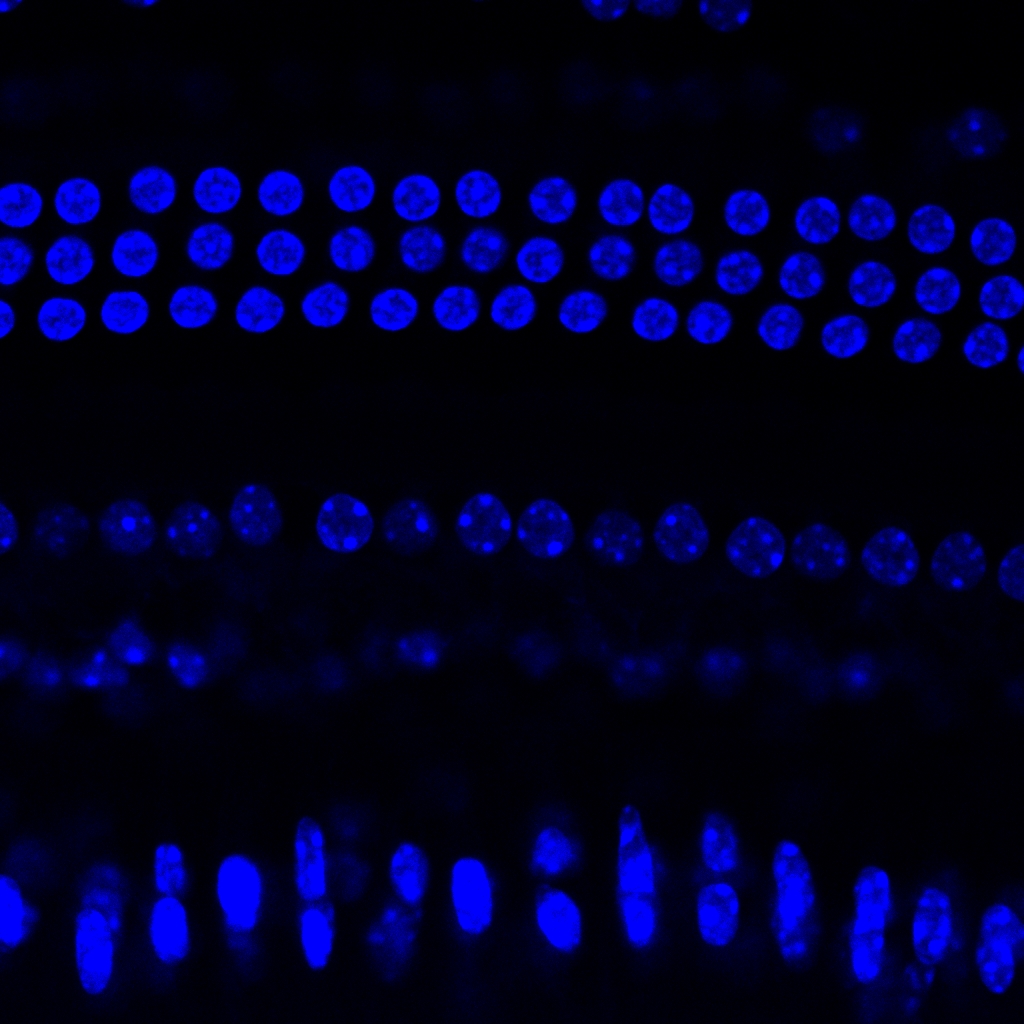

Supplement: Figure 4—source data 1. [file elife-76754-fig4-data1.zip › Figure_4_source_data/Fig.4 G/WT DAPI Apex.tif]

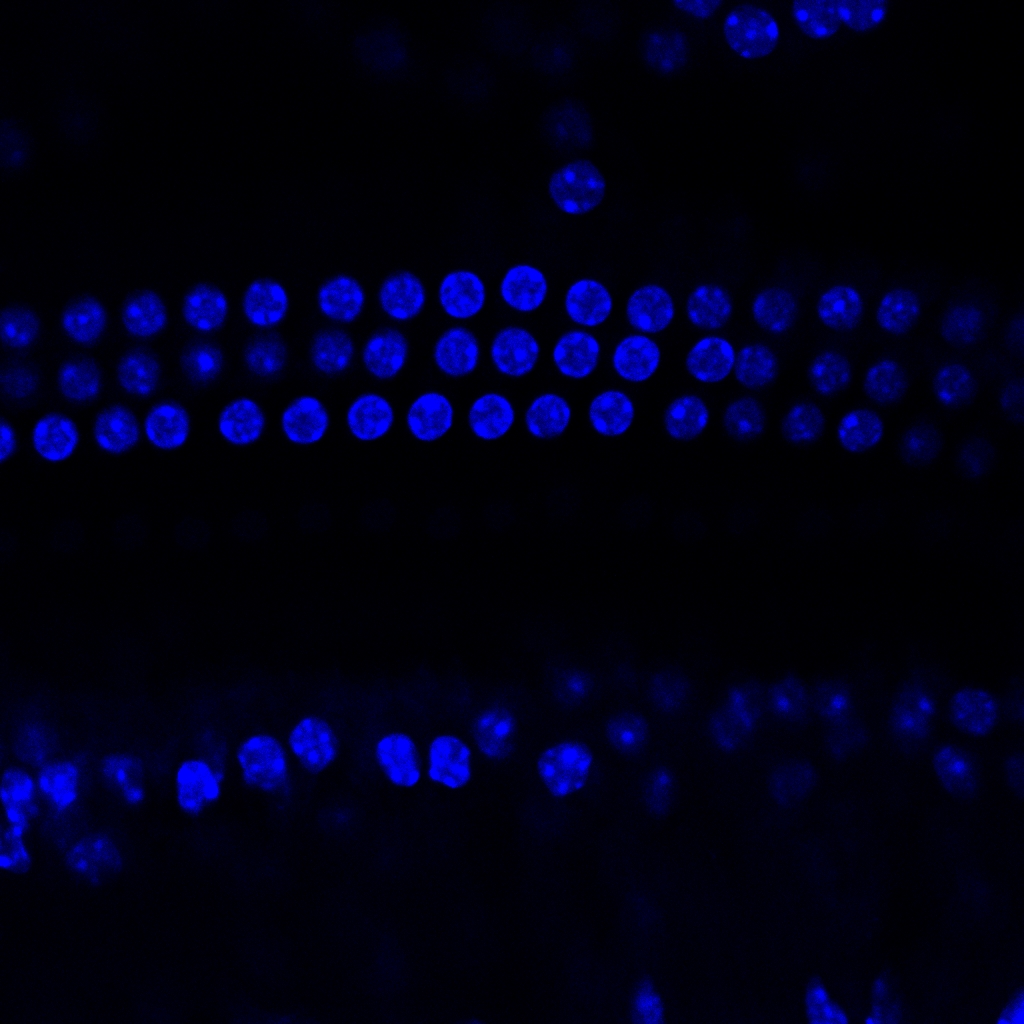

Supplement: Figure 4—source data 1. [file elife-76754-fig4-data1.zip › Figure_4_source_data/Fig.4 G/WT DAPI Base.tif]

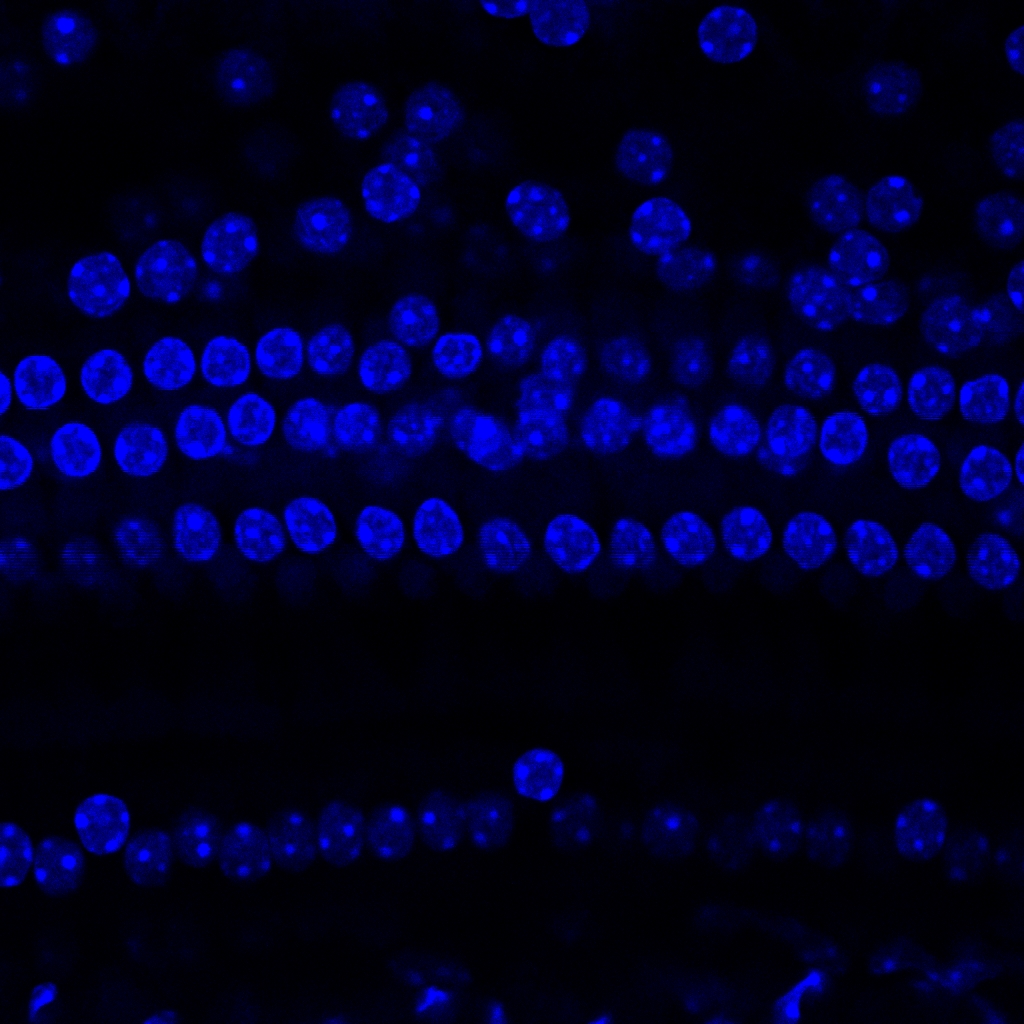

Supplement: Figure 4—source data 1. [file elife-76754-fig4-data1.zip › Figure_4_source_data/Fig.4 G/WT DAPI Middle.tif]

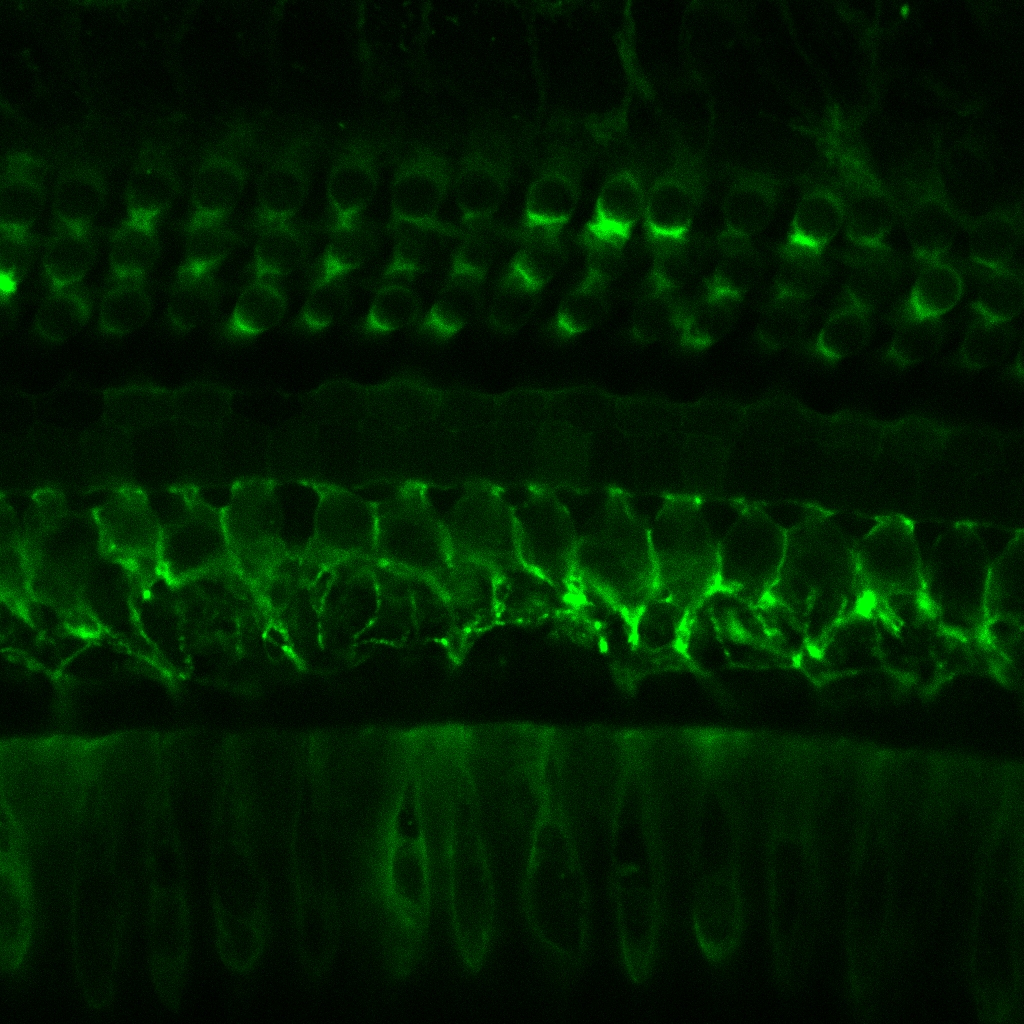

Supplement: Figure 4—source data 1. [file elife-76754-fig4-data1.zip › Figure_4_source_data/Fig.4 G/WT Kv7.4 Apex.tif]

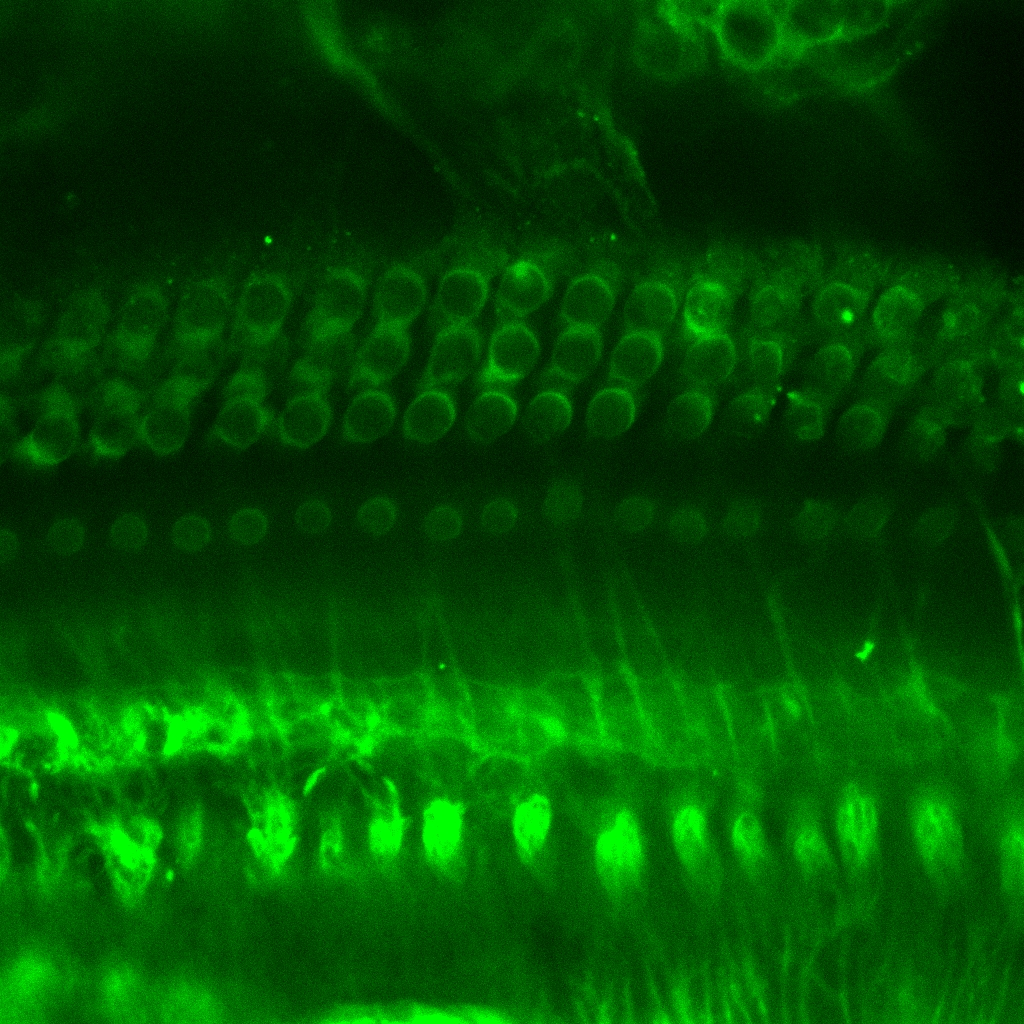

Supplement: Figure 4—source data 1. [file elife-76754-fig4-data1.zip › Figure_4_source_data/Fig.4 G/WT Kv7.4 Base.tif]

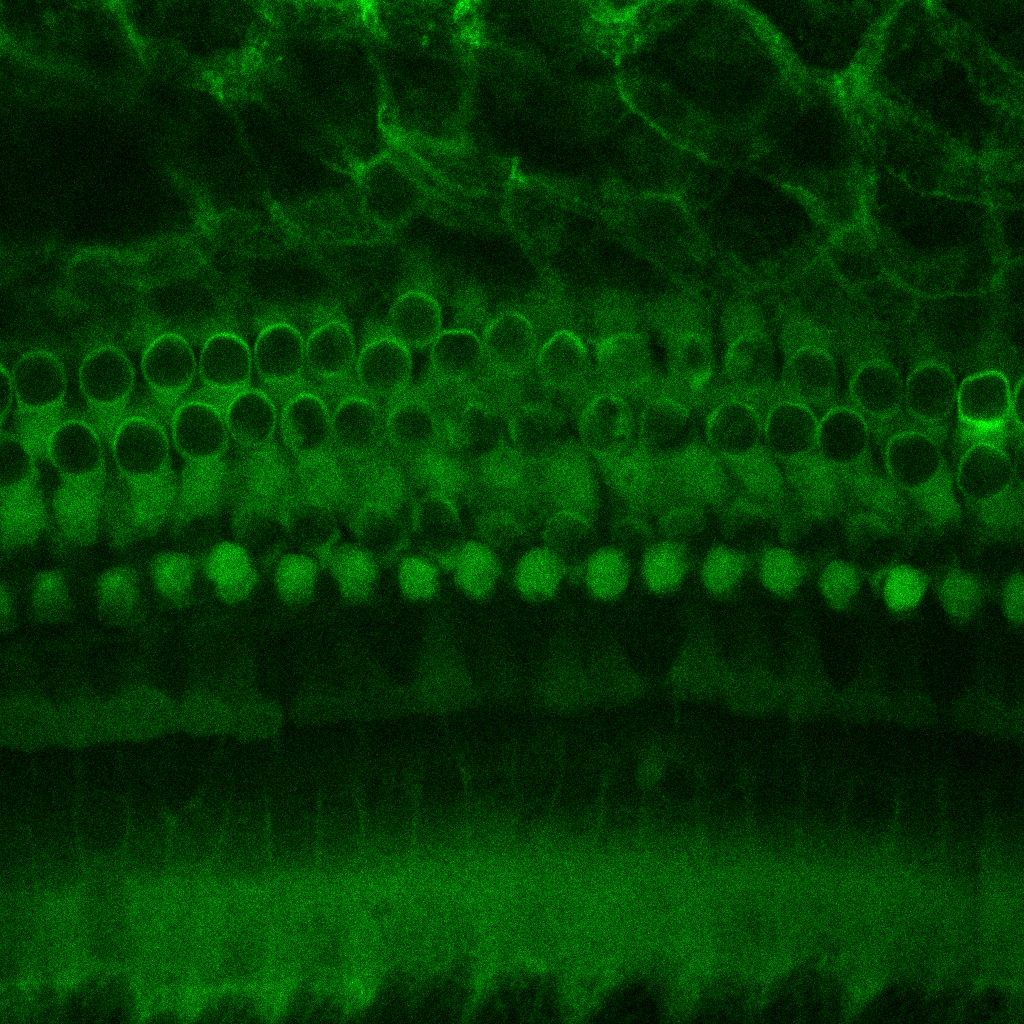

Supplement: Figure 4—source data 1. [file elife-76754-fig4-data1.zip › Figure_4_source_data/Fig.4 G/WT Kv7.4 Middle.tif]

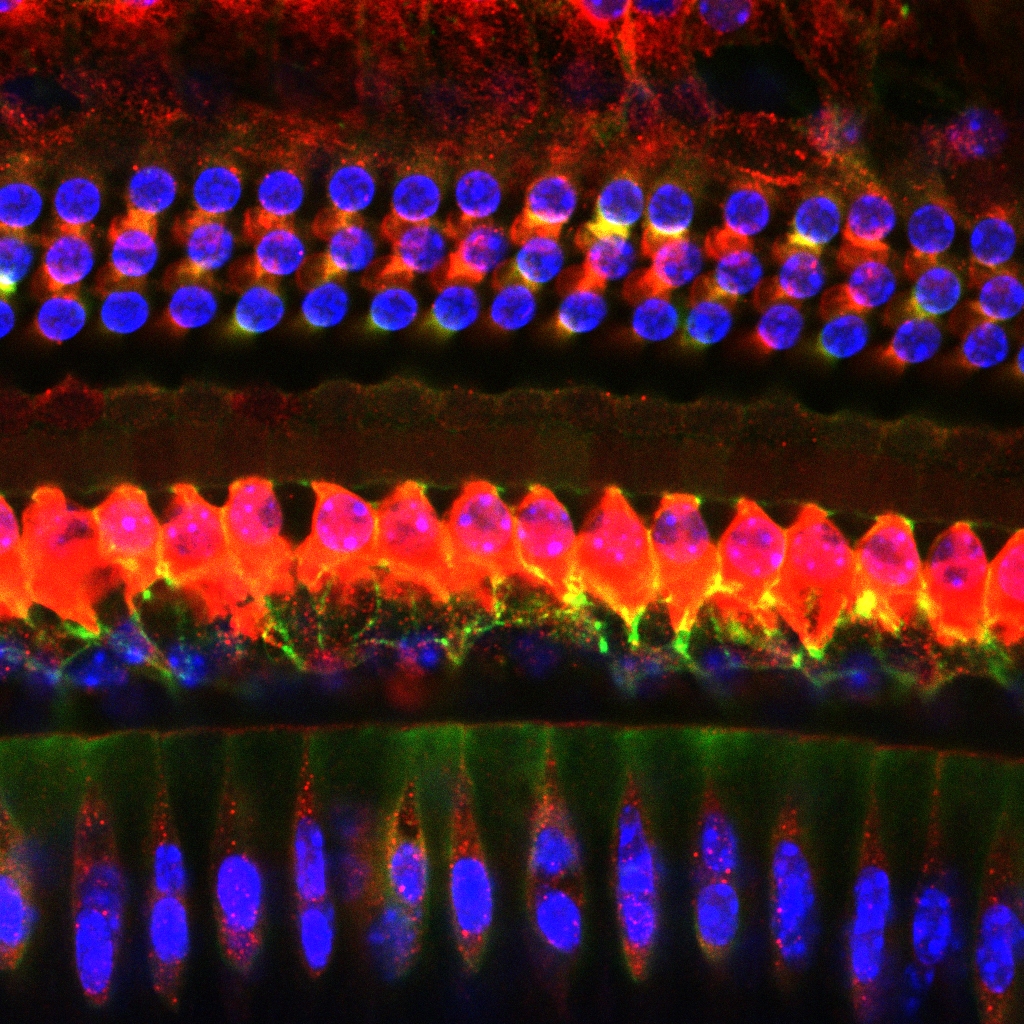

Supplement: Figure 4—source data 1. [file elife-76754-fig4-data1.zip › Figure_4_source_data/Fig.4 G/WT Merge Apex.tif]

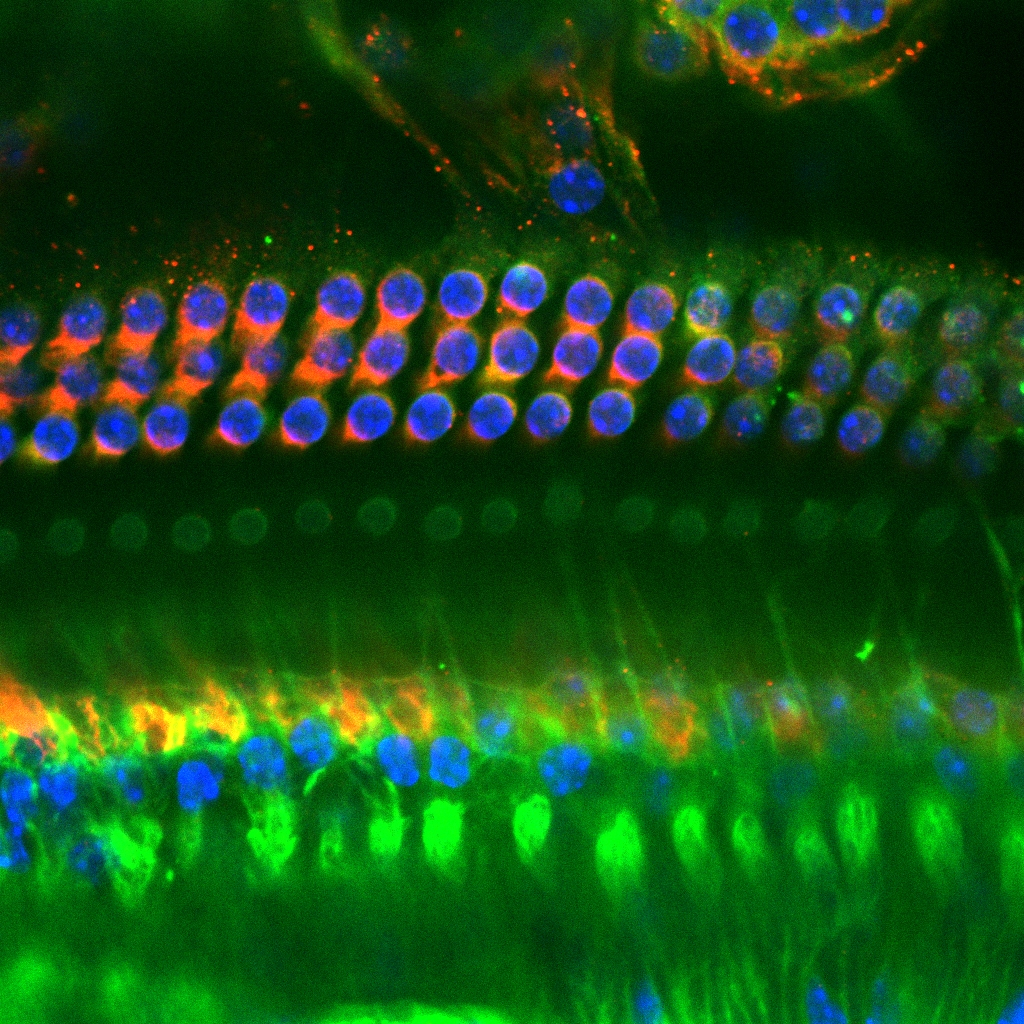

Supplement: Figure 4—source data 1. [file elife-76754-fig4-data1.zip › Figure_4_source_data/Fig.4 G/WT Merge Base.tif]

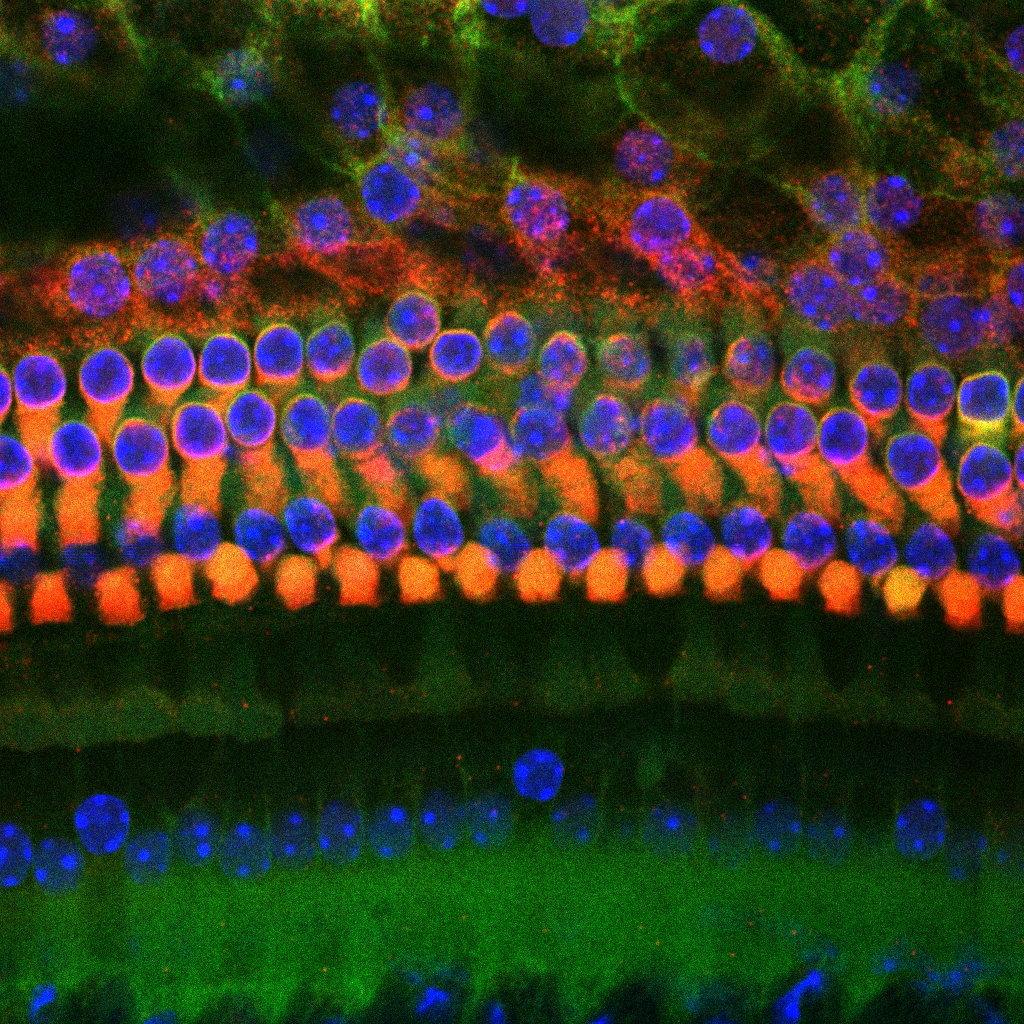

Supplement: Figure 4—source data 1. [file elife-76754-fig4-data1.zip › Figure_4_source_data/Fig.4 G/WT Merge Middle.tif]

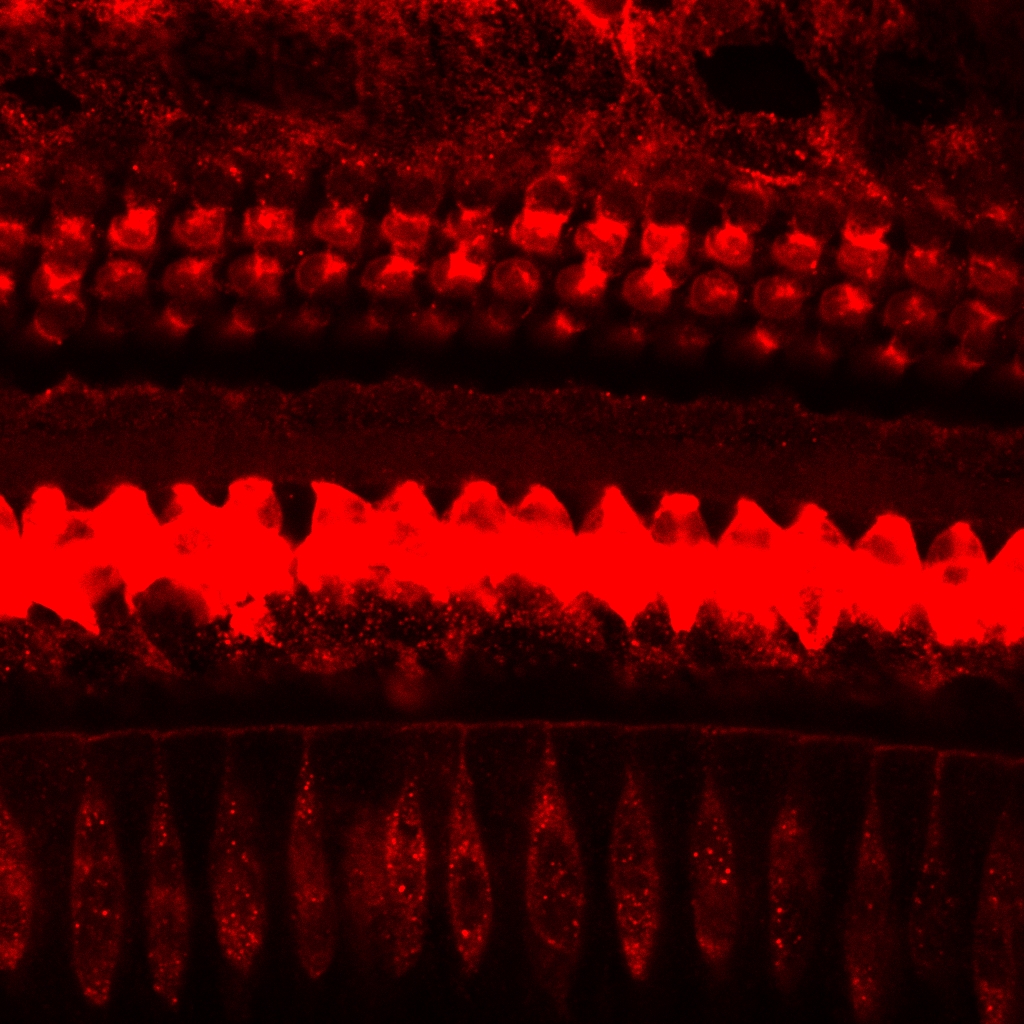

Supplement: Figure 4—source data 1. [file elife-76754-fig4-data1.zip › Figure_4_source_data/Fig.4 G/WT myo7a Apex.tif]

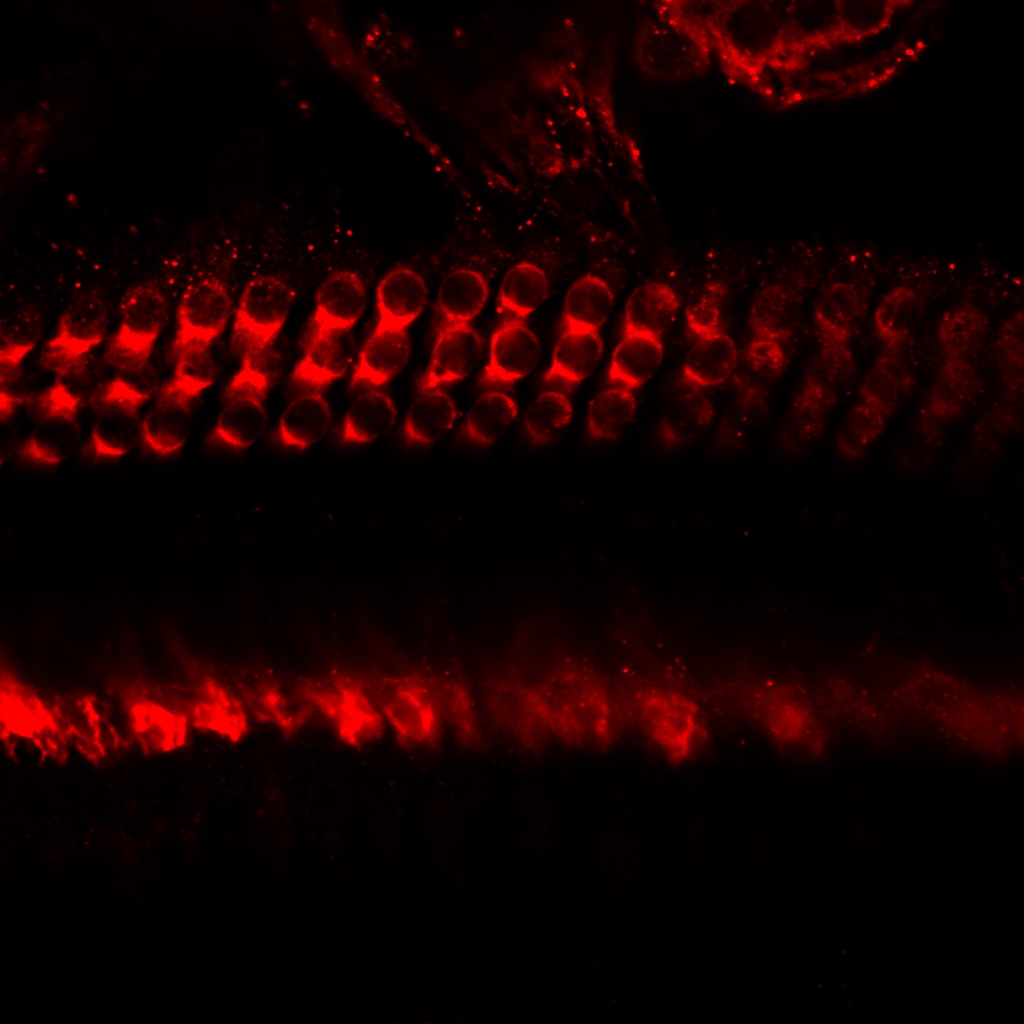

Supplement: Figure 4—source data 1. [file elife-76754-fig4-data1.zip › Figure_4_source_data/Fig.4 G/WT myo7a Base.tif]

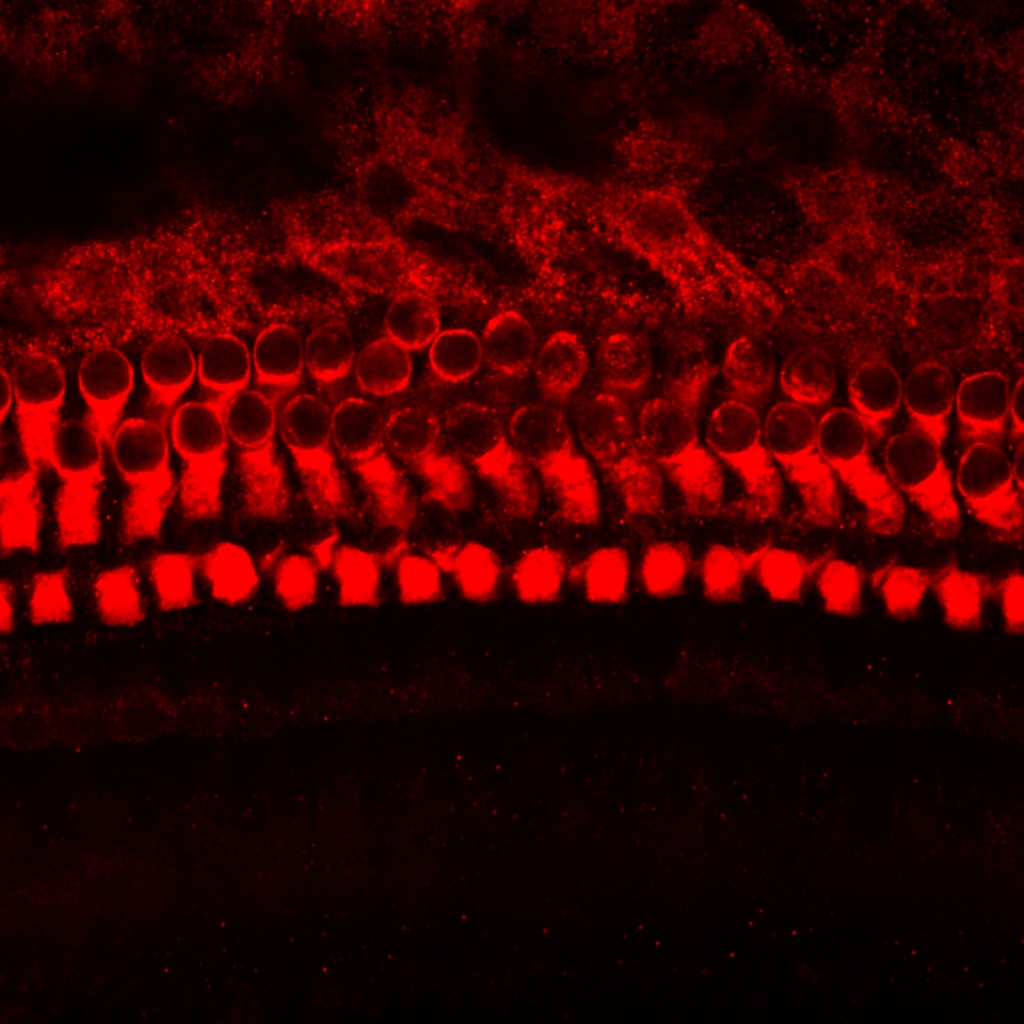

Supplement: Figure 4—source data 1. [file elife-76754-fig4-data1.zip › Figure_4_source_data/Fig.4 G/WT myo7a Middle.tif]

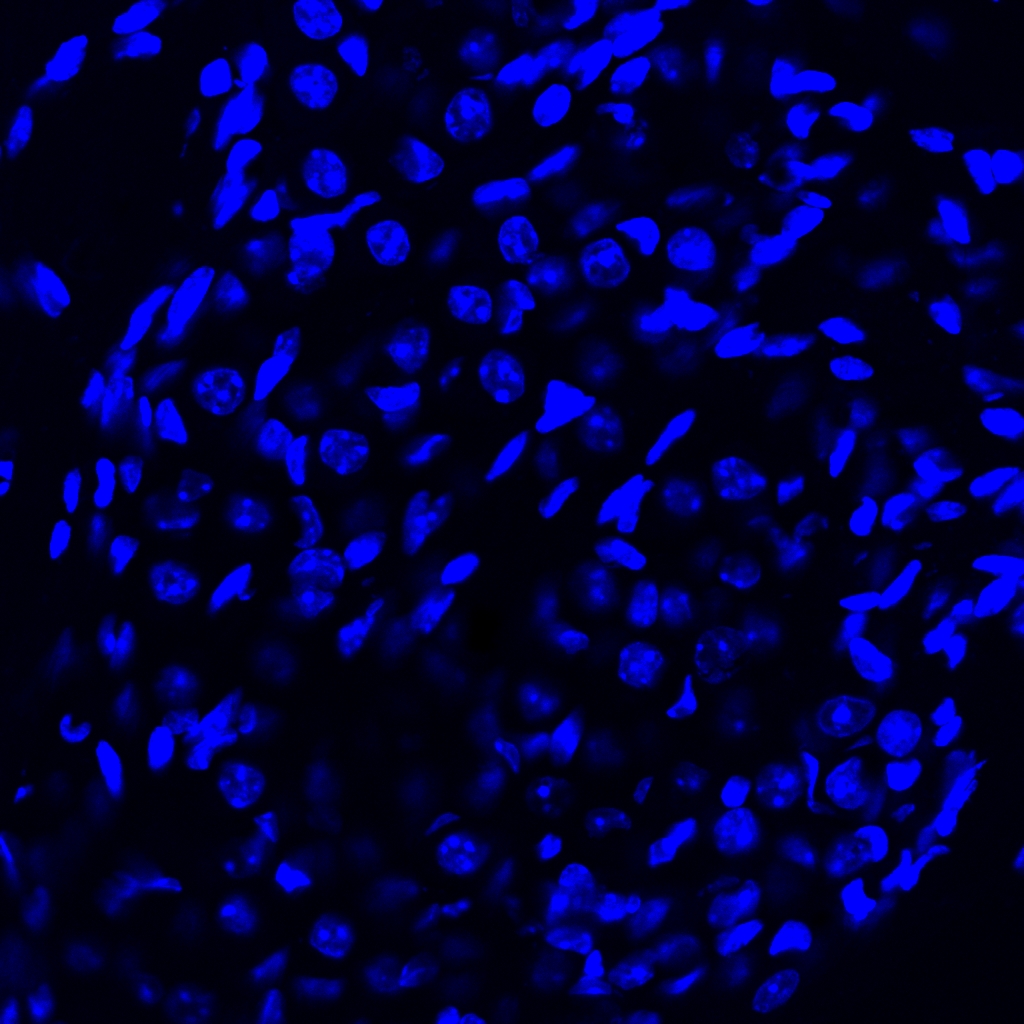

Supplement: Figure 5—figure supplement 1—source data 1. [file elife-76754-fig5-figsupp1-data1.zip › Figure 5 - figure supplement 1 Source data/Figure 5 - figure supplement 1A/RESTcKO DAPI Base.tif]

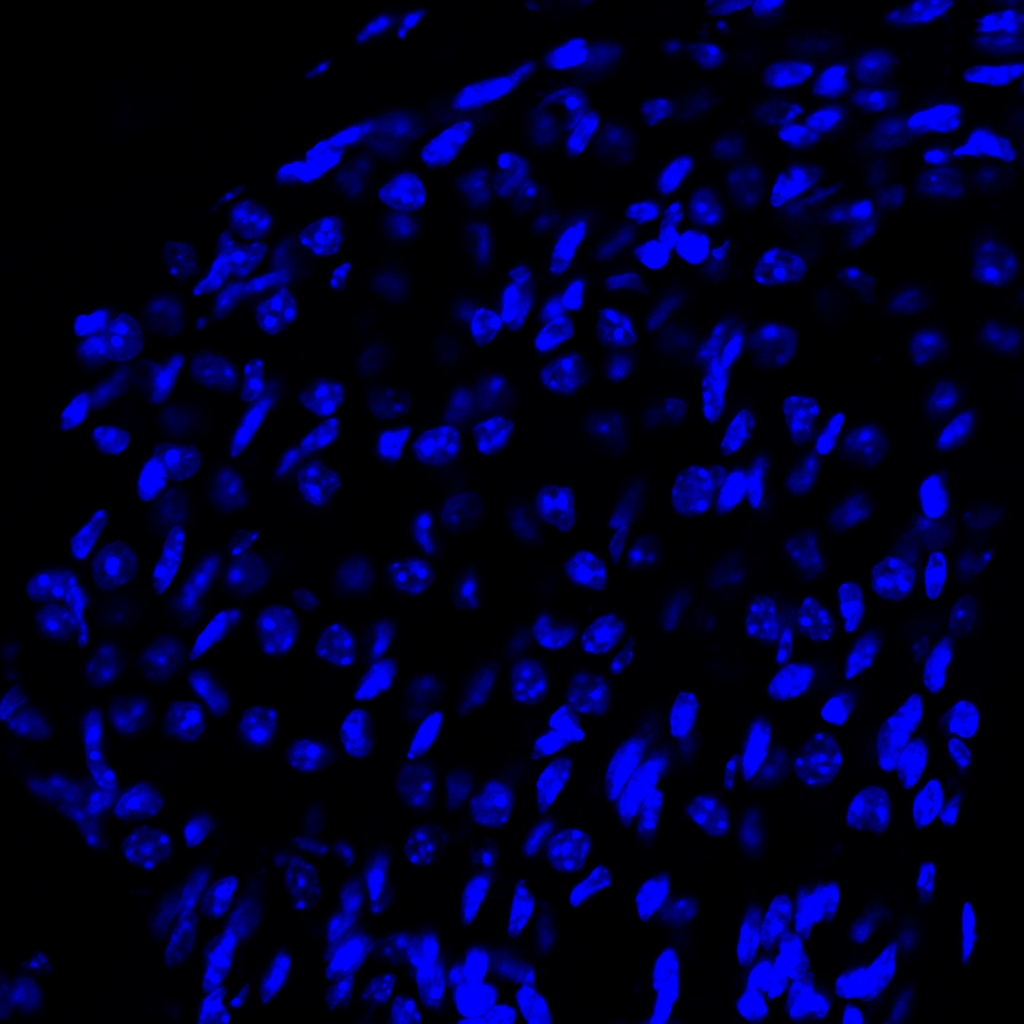

Supplement: Figure 5—figure supplement 1—source data 1. [file elife-76754-fig5-figsupp1-data1.zip › Figure 5 - figure supplement 1 Source data/Figure 5 - figure supplement 1A/RESTcKO DAPI Apex.tif]

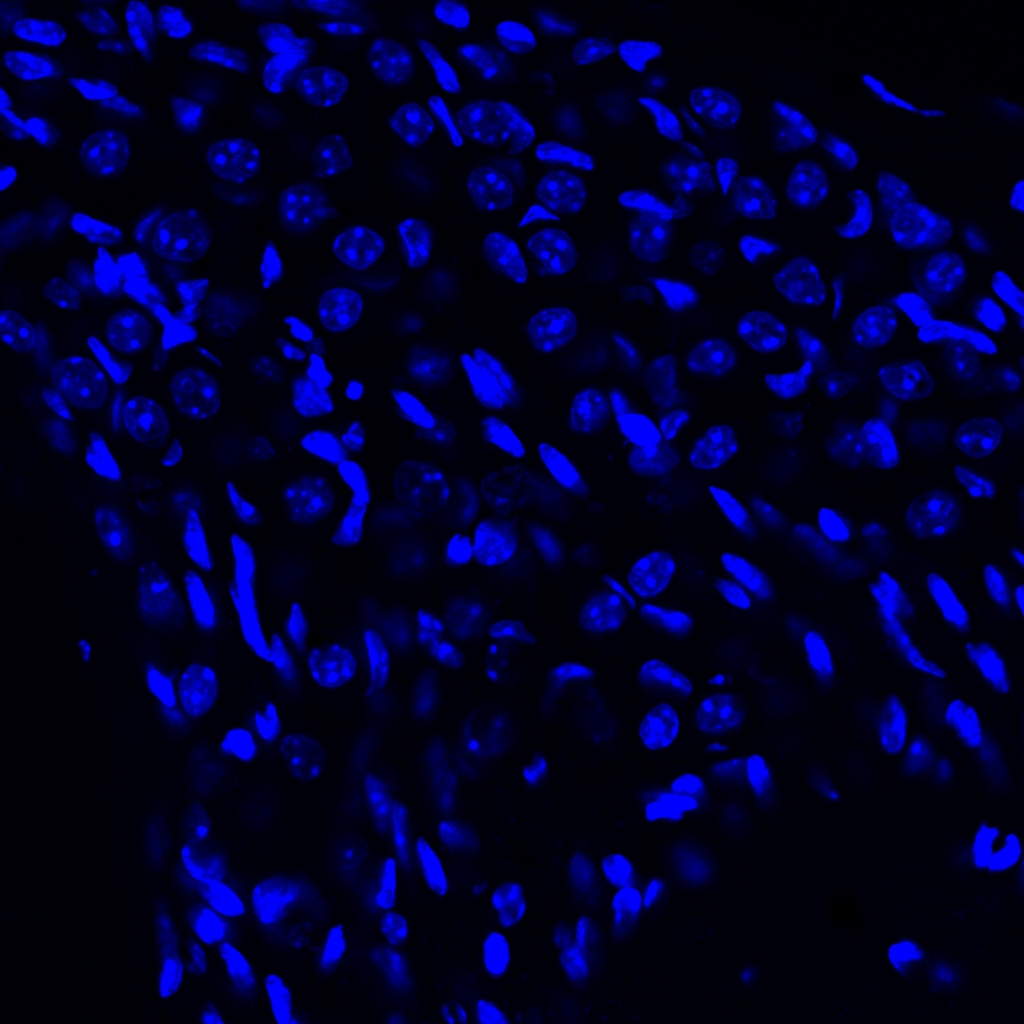

Supplement: Figure 5—figure supplement 1—source data 1. [file elife-76754-fig5-figsupp1-data1.zip › Figure 5 - figure supplement 1 Source data/Figure 5 - figure supplement 1A/RESTcKO DAPI Middle.tif]

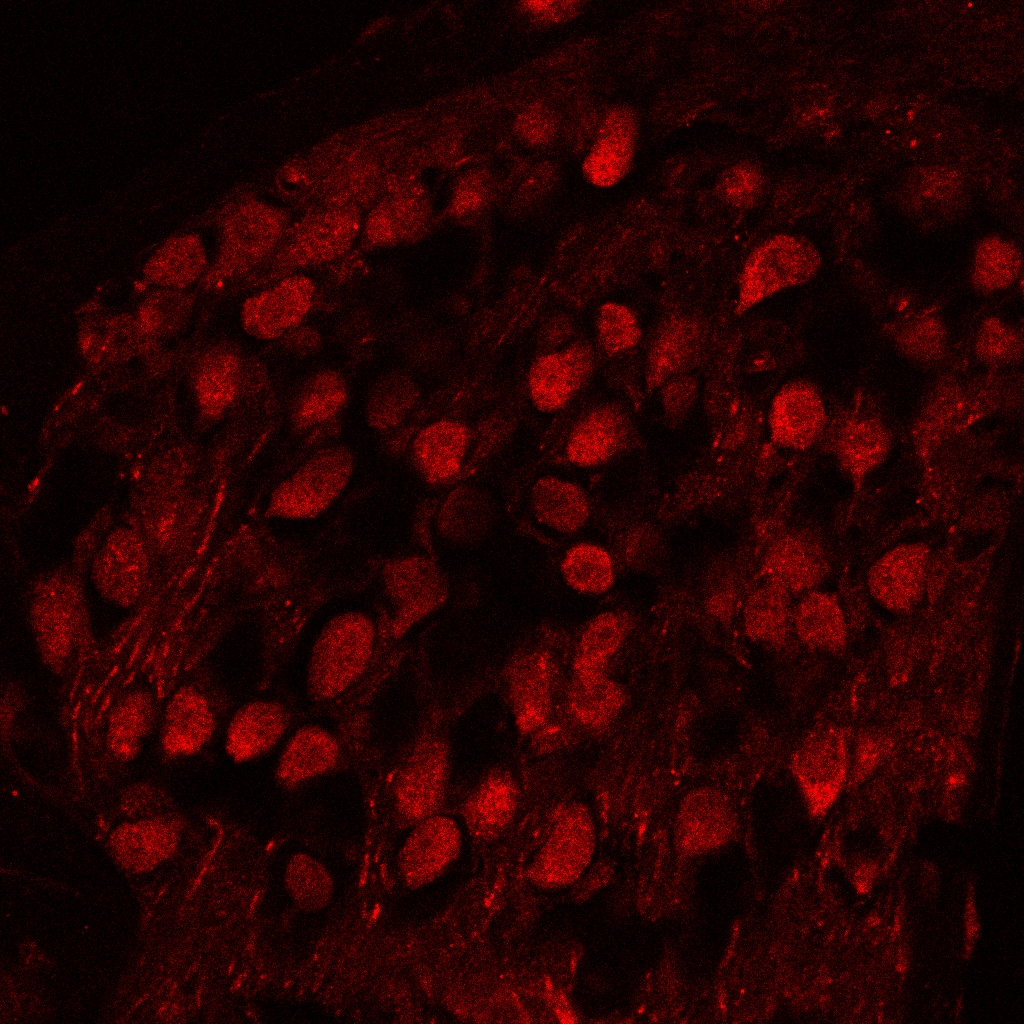

Supplement: Figure 5—figure supplement 1—source data 1. [file elife-76754-fig5-figsupp1-data1.zip › Figure 5 - figure supplement 1 Source data/Figure 5 - figure supplement 1A/RESTcKO Kv7.2 Apex.tif]

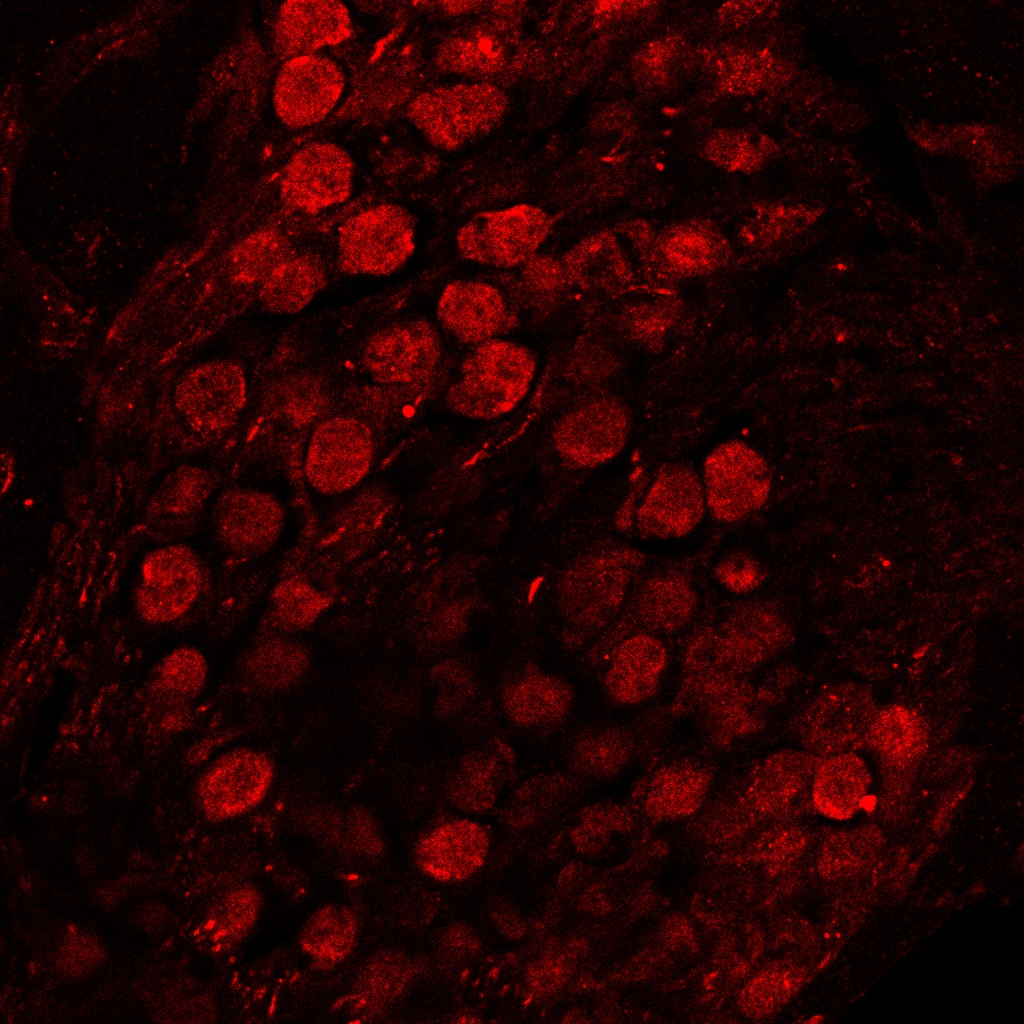

Supplement: Figure 5—figure supplement 1—source data 1. [file elife-76754-fig5-figsupp1-data1.zip › Figure 5 - figure supplement 1 Source data/Figure 5 - figure supplement 1A/RESTcKO Kv7.2 Base.tif]

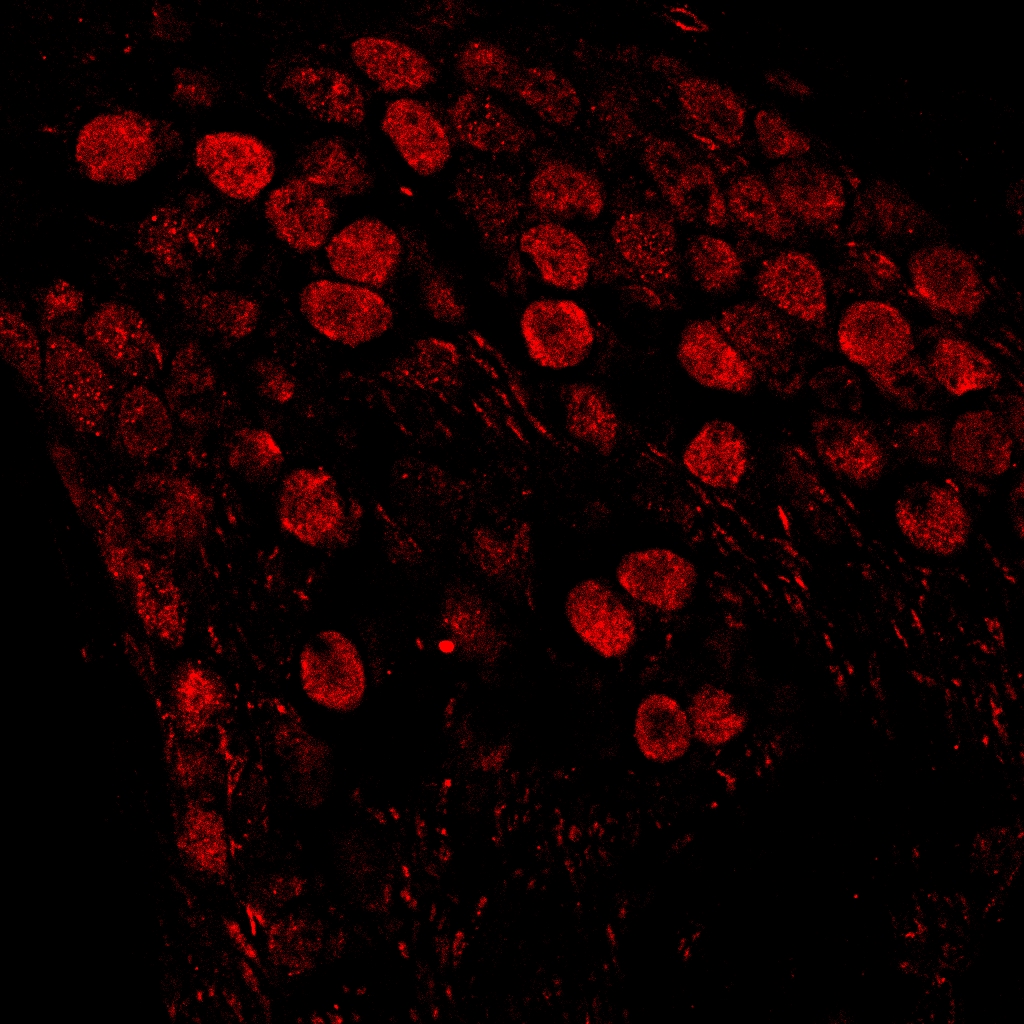

Supplement: Figure 5—figure supplement 1—source data 1. [file elife-76754-fig5-figsupp1-data1.zip › Figure 5 - figure supplement 1 Source data/Figure 5 - figure supplement 1A/RESTcKO Kv7.2 Middle.tif]

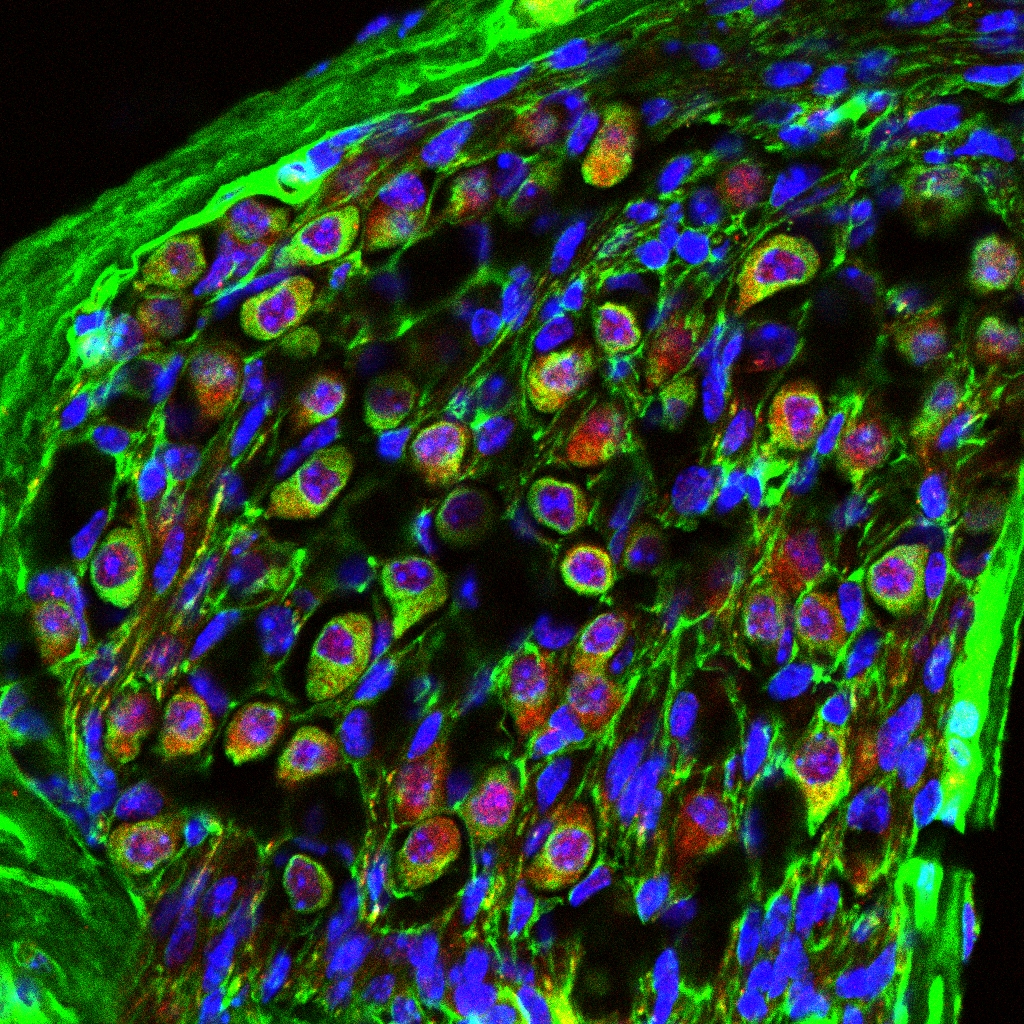

Supplement: Figure 5—figure supplement 1—source data 1. [file elife-76754-fig5-figsupp1-data1.zip › Figure 5 - figure supplement 1 Source data/Figure 5 - figure supplement 1A/RESTcKO Merge Apex.tif]

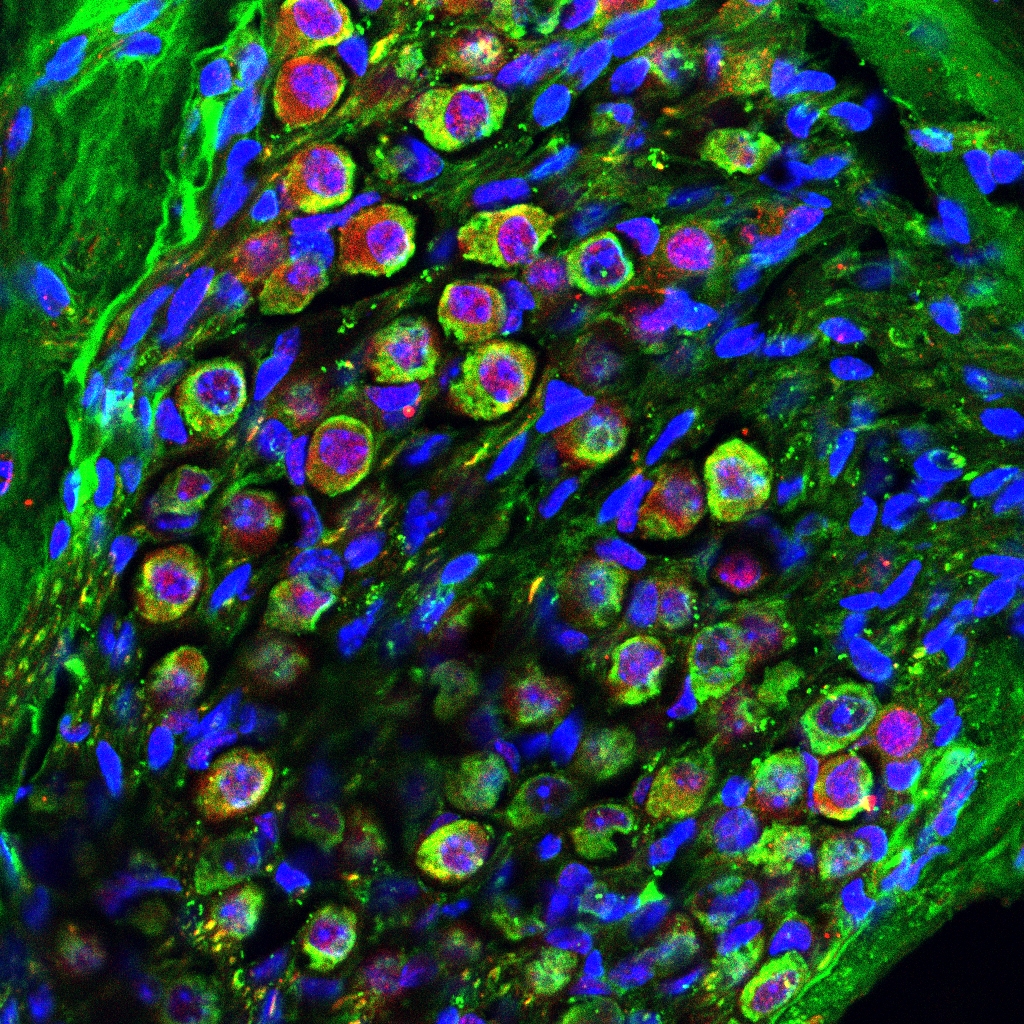

Supplement: Figure 5—figure supplement 1—source data 1. [file elife-76754-fig5-figsupp1-data1.zip › Figure 5 - figure supplement 1 Source data/Figure 5 - figure supplement 1A/RESTcKO Merge Base.tif]

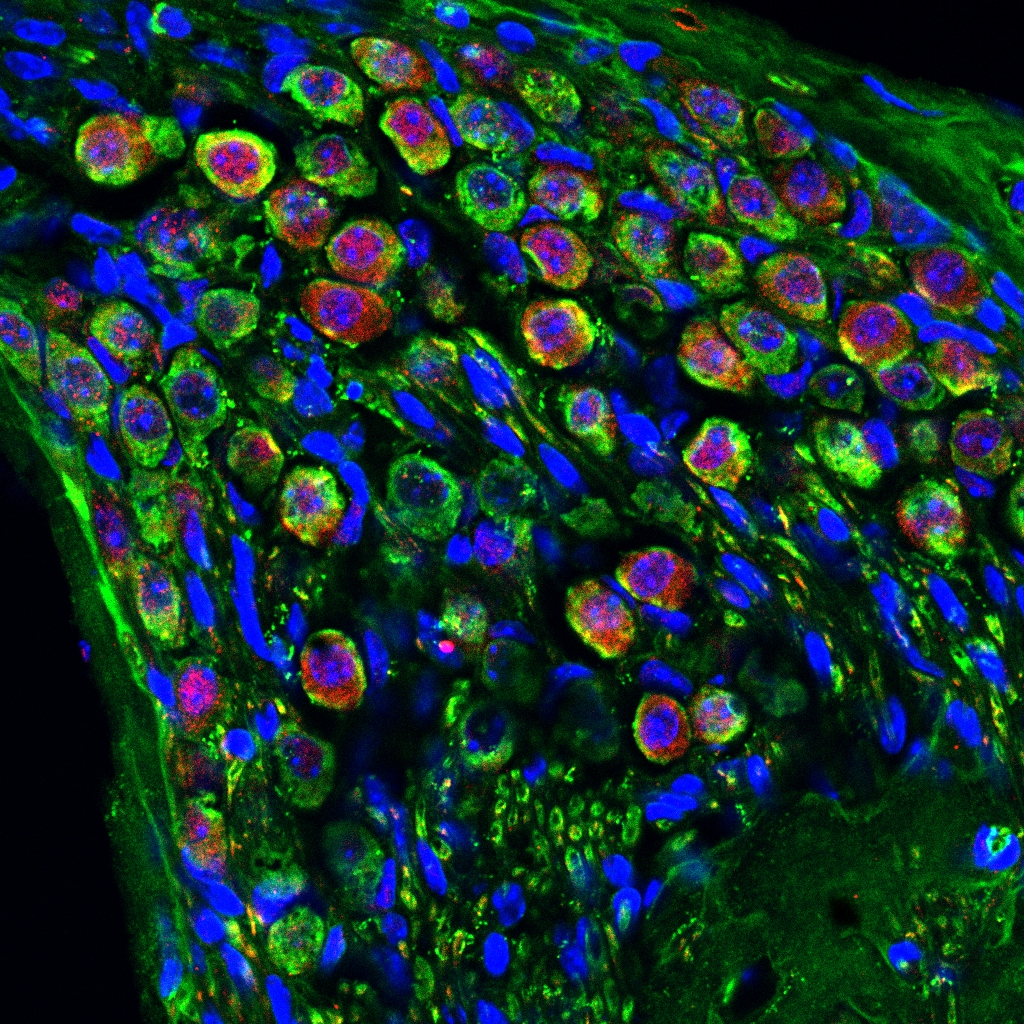

Supplement: Figure 5—figure supplement 1—source data 1. [file elife-76754-fig5-figsupp1-data1.zip › Figure 5 - figure supplement 1 Source data/Figure 5 - figure supplement 1A/RESTcKO Merge Middle.tif]

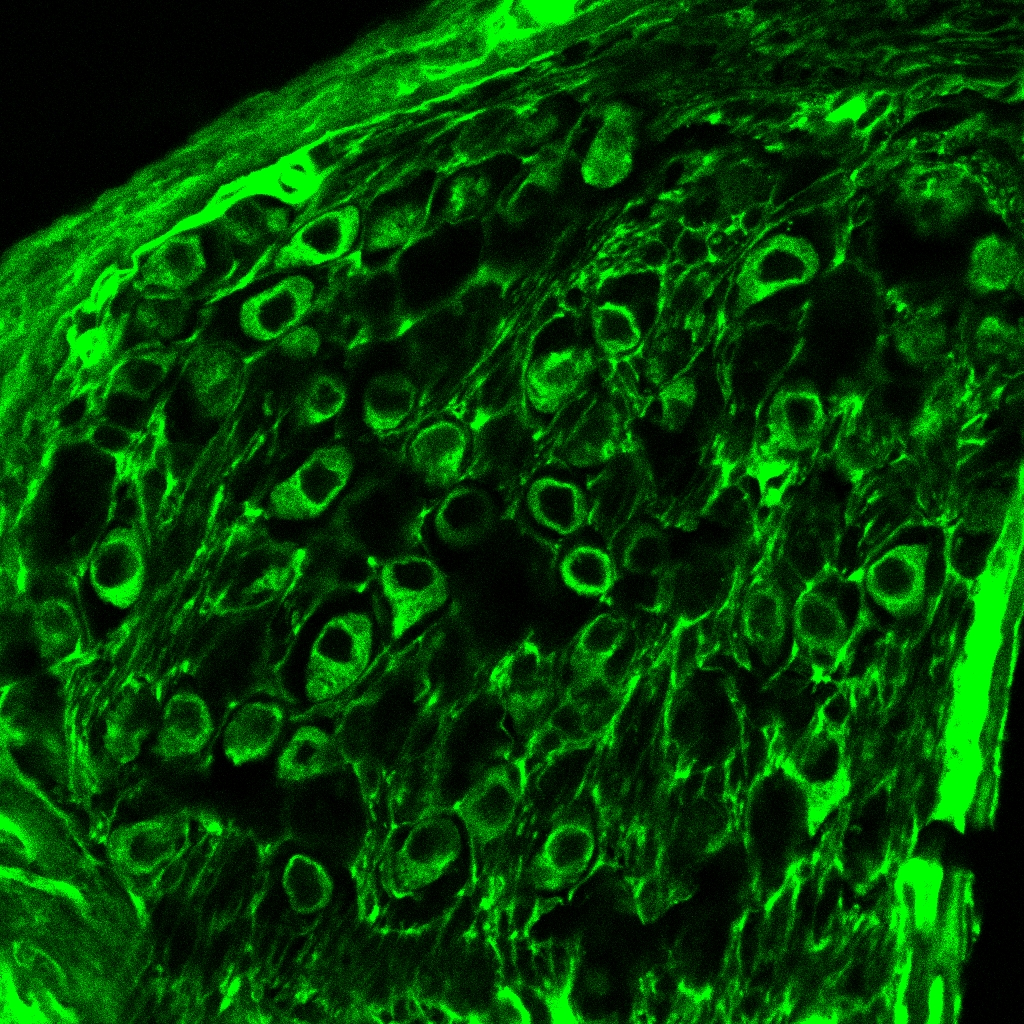

Supplement: Figure 5—figure supplement 1—source data 1. [file elife-76754-fig5-figsupp1-data1.zip › Figure 5 - figure supplement 1 Source data/Figure 5 - figure supplement 1A/RESTcKO Tuj1 Apex.tif]

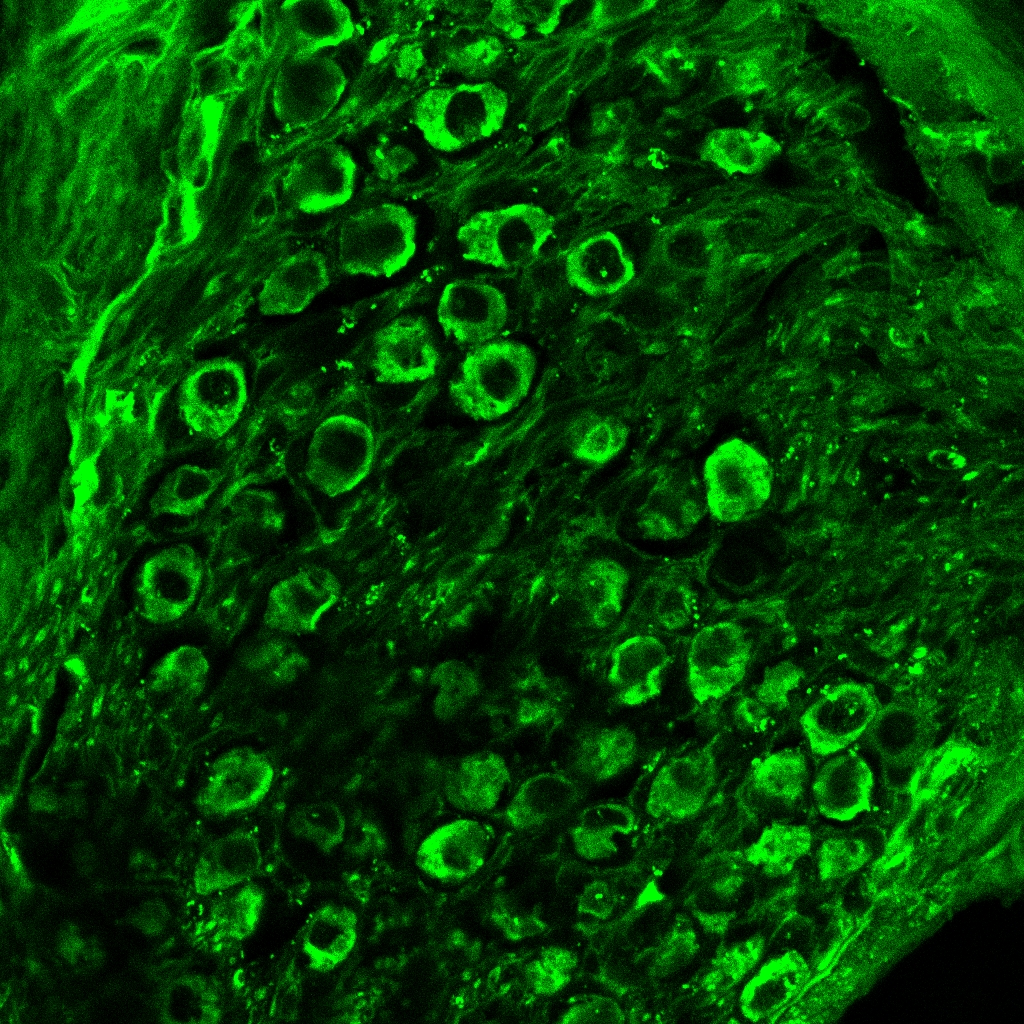

Supplement: Figure 5—figure supplement 1—source data 1. [file elife-76754-fig5-figsupp1-data1.zip › Figure 5 - figure supplement 1 Source data/Figure 5 - figure supplement 1A/RESTcKO Tuj1 Base.tif]

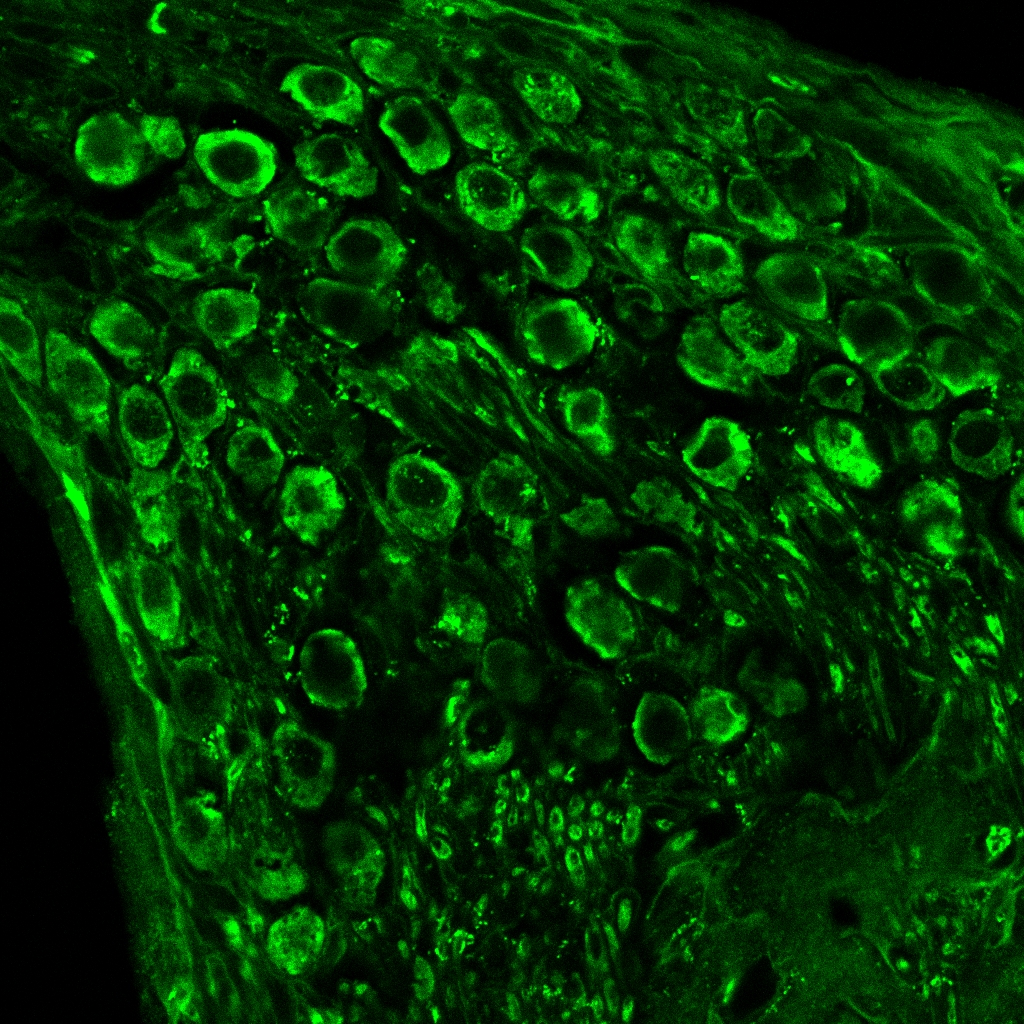

Supplement: Figure 5—figure supplement 1—source data 1. [file elife-76754-fig5-figsupp1-data1.zip › Figure 5 - figure supplement 1 Source data/Figure 5 - figure supplement 1A/RESTcKO Tuj1 Middle.tif]

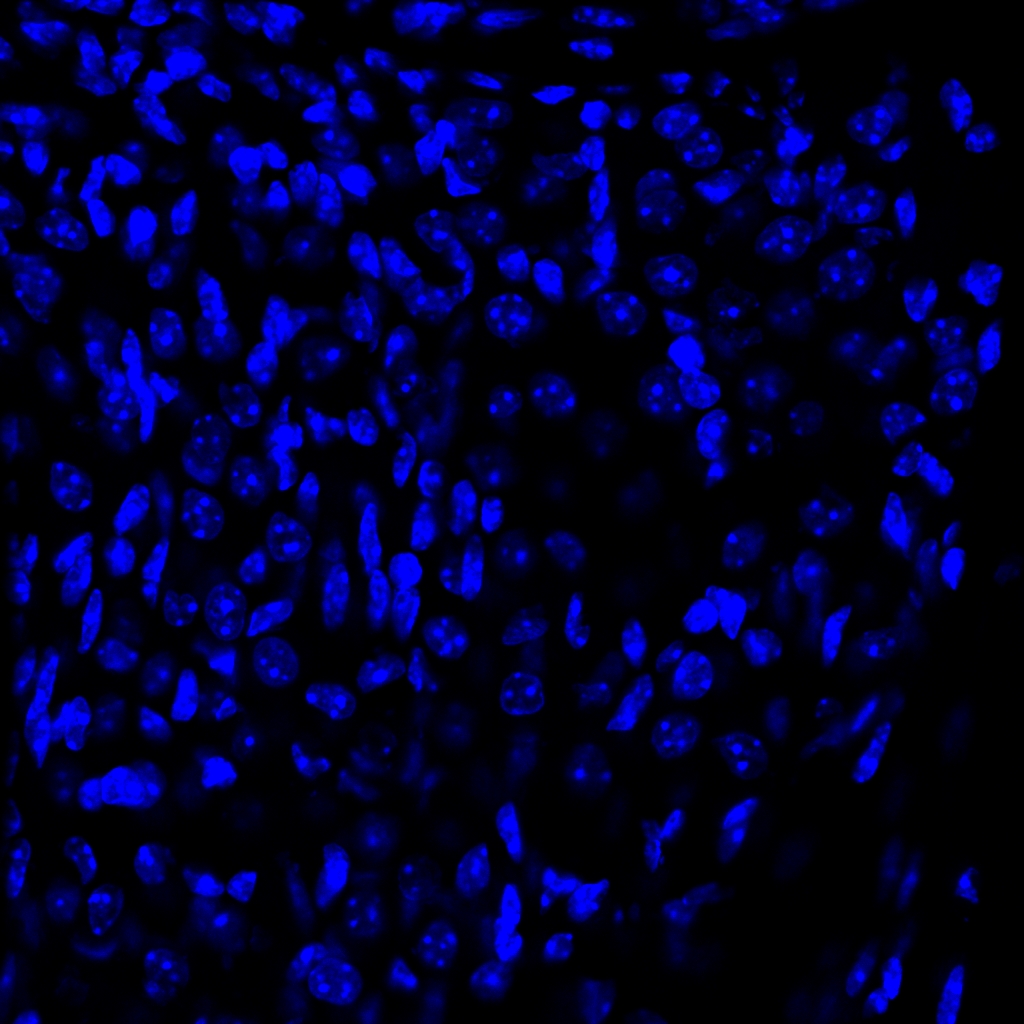

Supplement: Figure 5—figure supplement 1—source data 1. [file elife-76754-fig5-figsupp1-data1.zip › Figure 5 - figure supplement 1 Source data/Figure 5 - figure supplement 1A/WT DAPI Apex.tif]

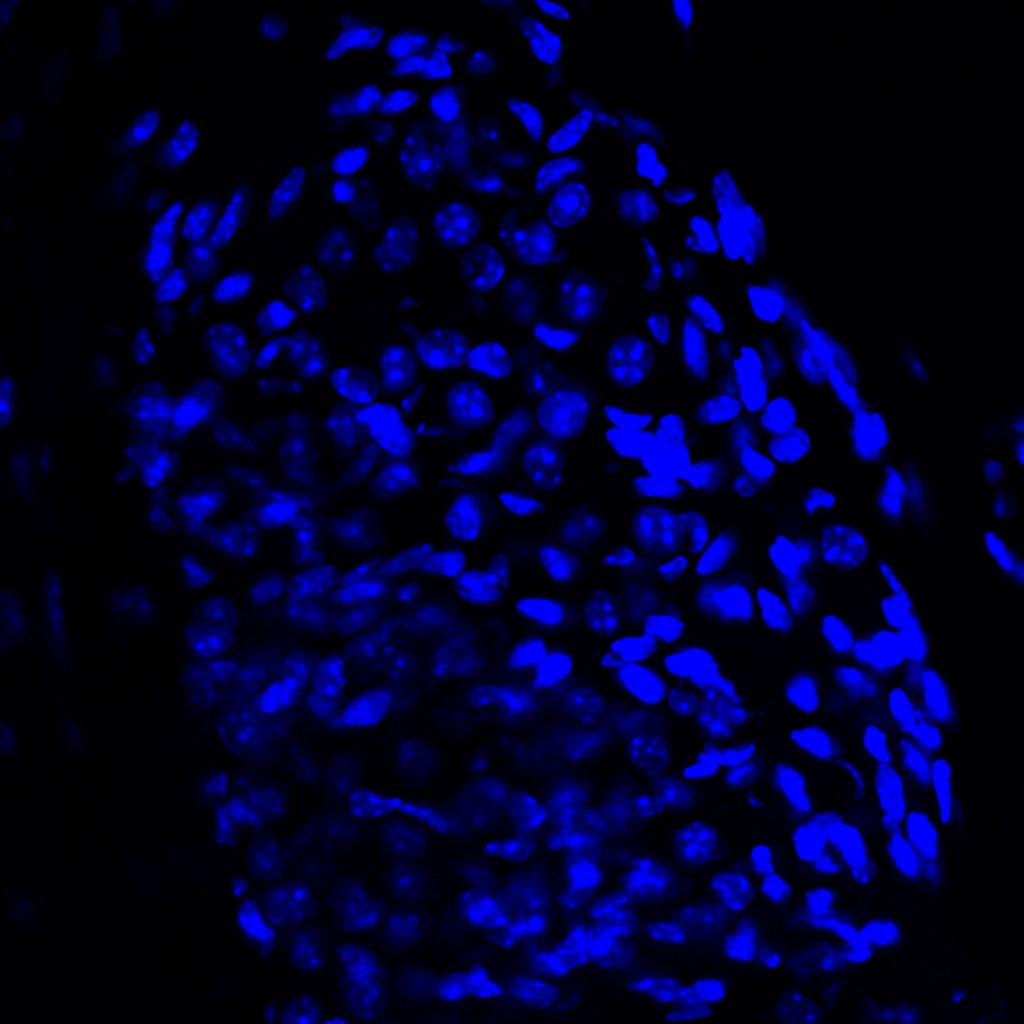

Supplement: Figure 5—figure supplement 1—source data 1. [file elife-76754-fig5-figsupp1-data1.zip › Figure 5 - figure supplement 1 Source data/Figure 5 - figure supplement 1A/WT DAPI Middle.tif]

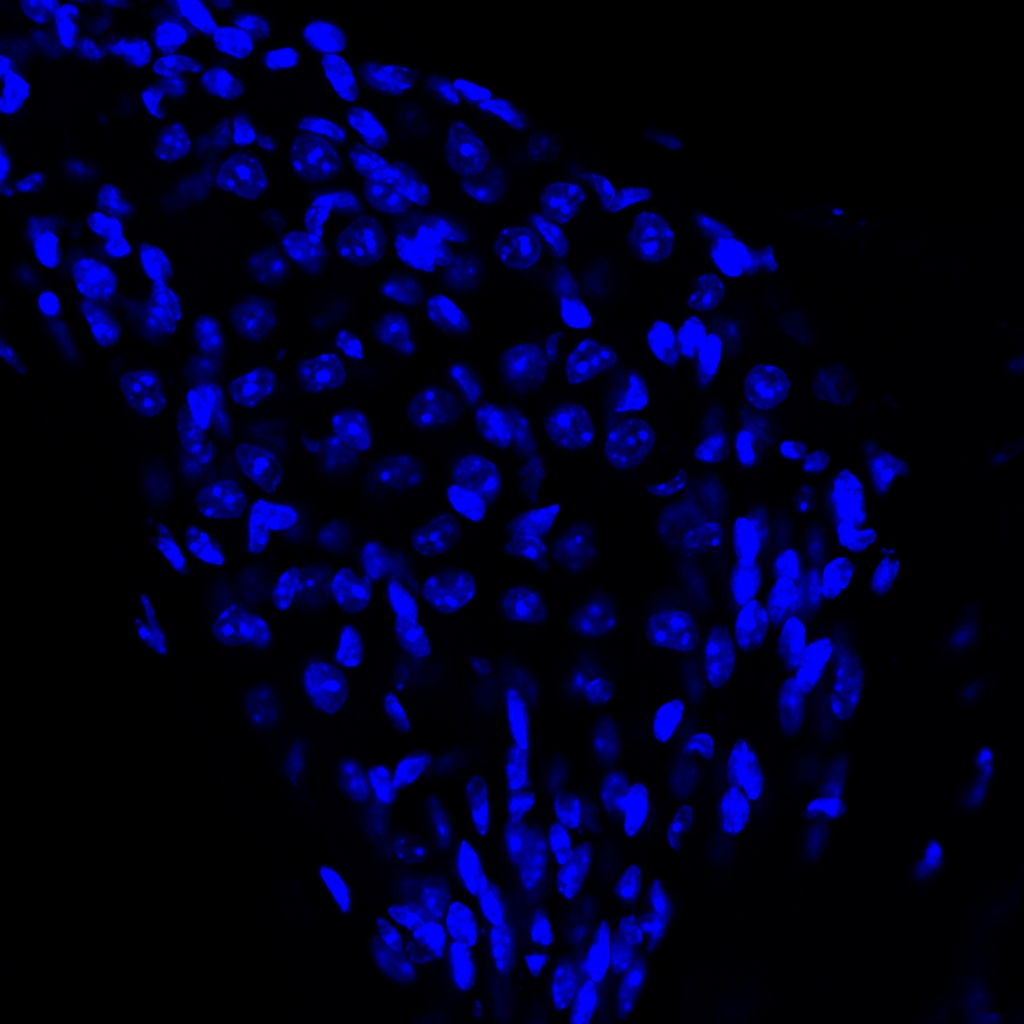

Supplement: Figure 5—figure supplement 1—source data 1. [file elife-76754-fig5-figsupp1-data1.zip › Figure 5 - figure supplement 1 Source data/Figure 5 - figure supplement 1A/WT DAPI Base.tif]

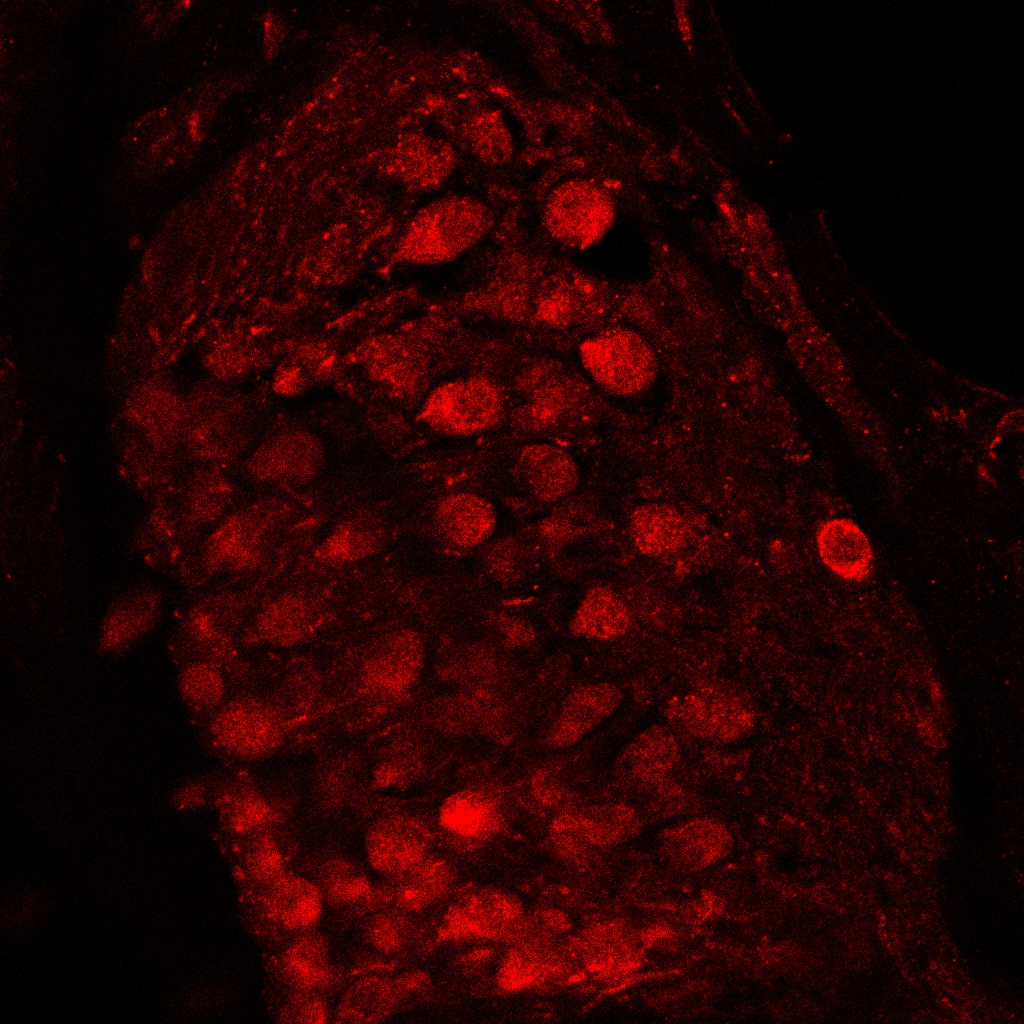

Supplement: Figure 5—figure supplement 1—source data 1. [file elife-76754-fig5-figsupp1-data1.zip › Figure 5 - figure supplement 1 Source data/Figure 5 - figure supplement 1A/WT Kv7.2 Middle.tif]

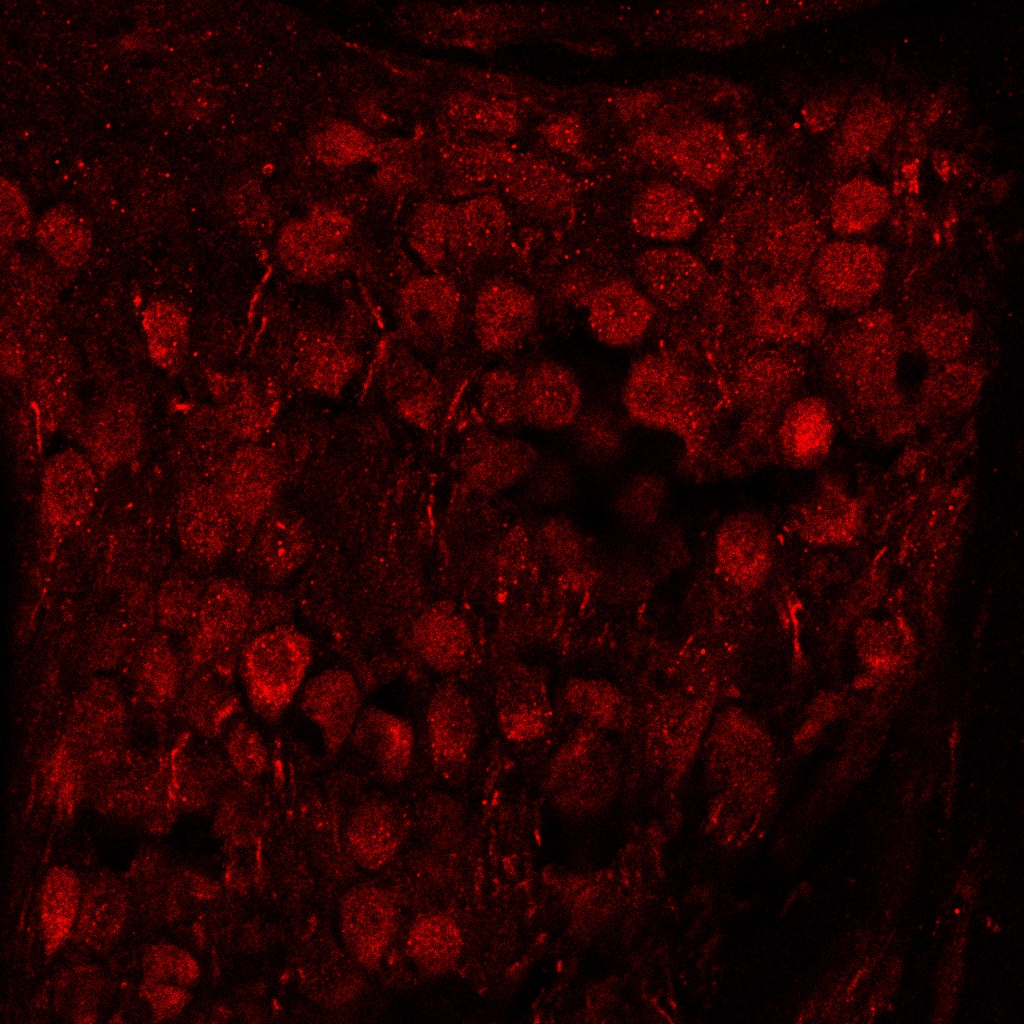

Supplement: Figure 5—figure supplement 1—source data 1. [file elife-76754-fig5-figsupp1-data1.zip › Figure 5 - figure supplement 1 Source data/Figure 5 - figure supplement 1A/WT Kv7.2 Apex.tif]

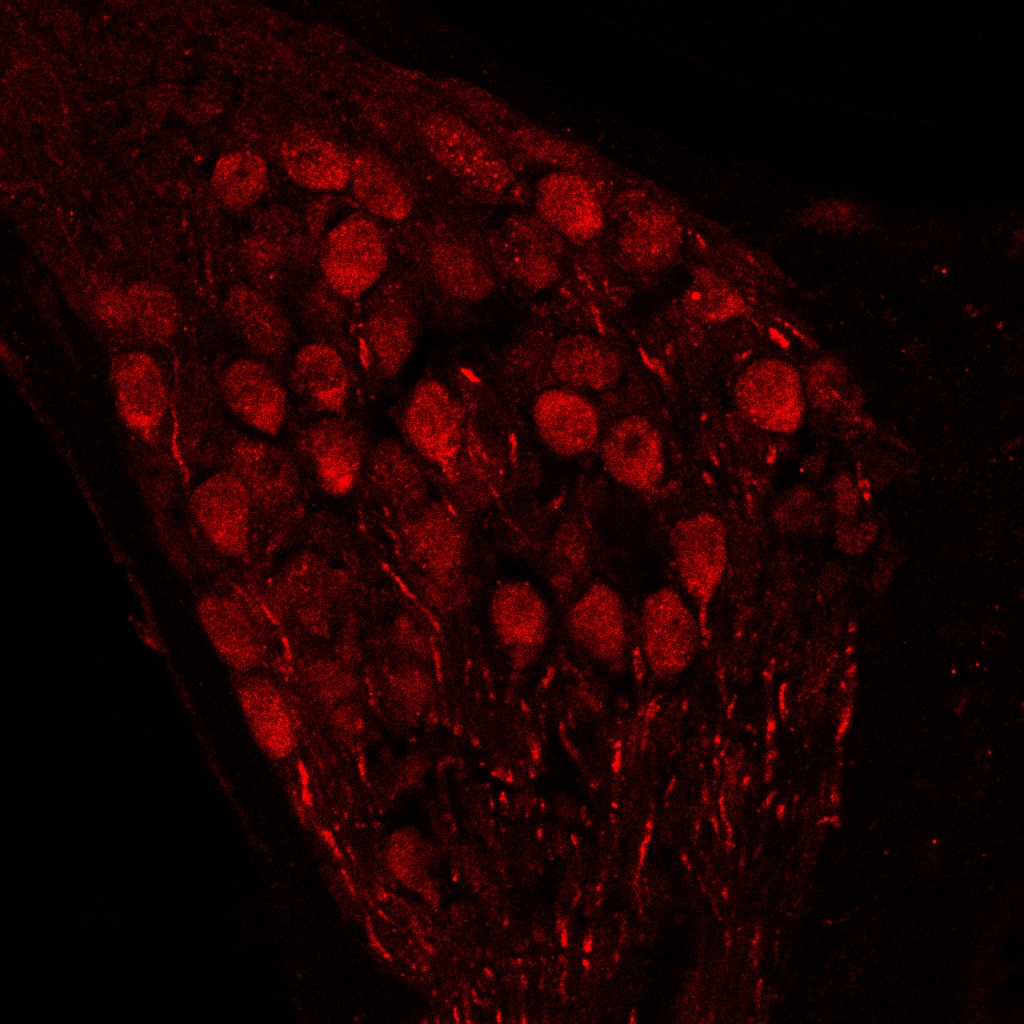

Supplement: Figure 5—figure supplement 1—source data 1. [file elife-76754-fig5-figsupp1-data1.zip › Figure 5 - figure supplement 1 Source data/Figure 5 - figure supplement 1A/WT Kv7.2 Base.tif]

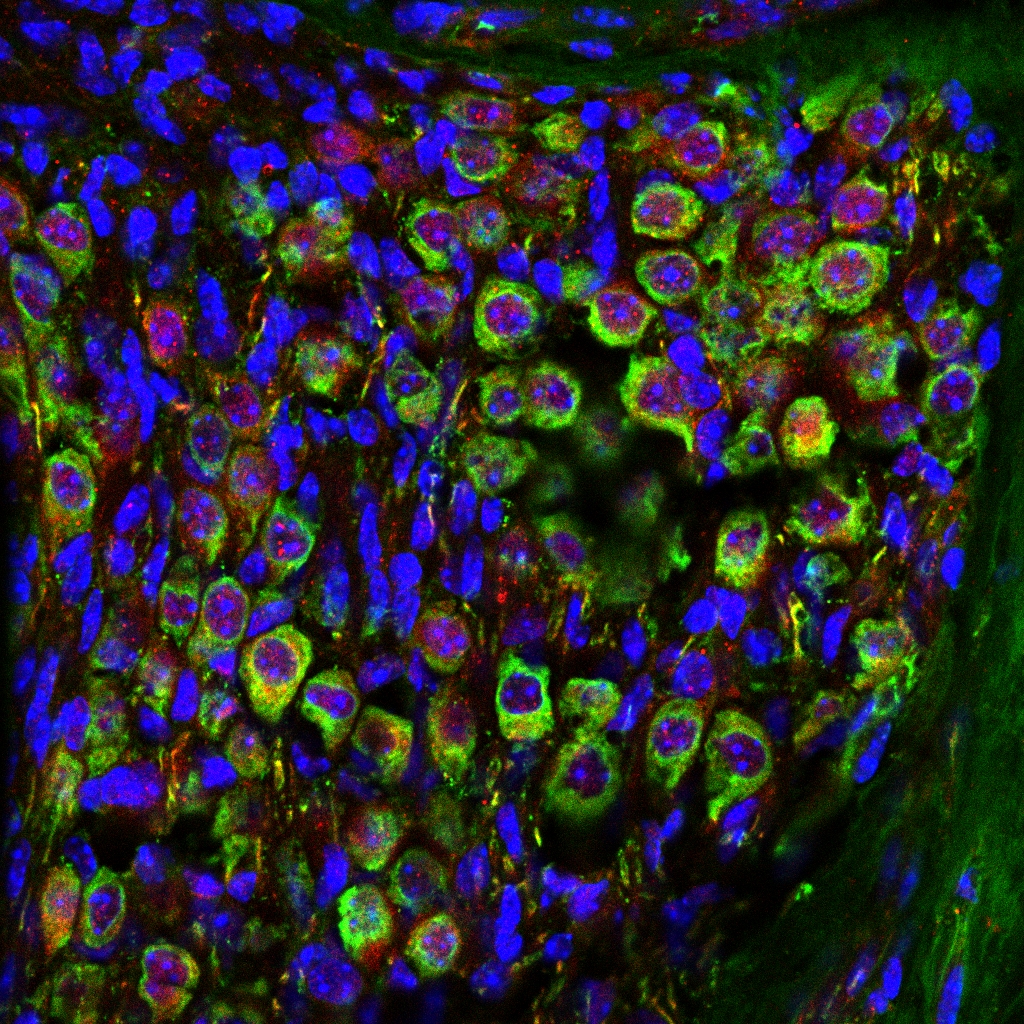

Supplement: Figure 5—figure supplement 1—source data 1. [file elife-76754-fig5-figsupp1-data1.zip › Figure 5 - figure supplement 1 Source data/Figure 5 - figure supplement 1A/WT Merge Apex.tif]

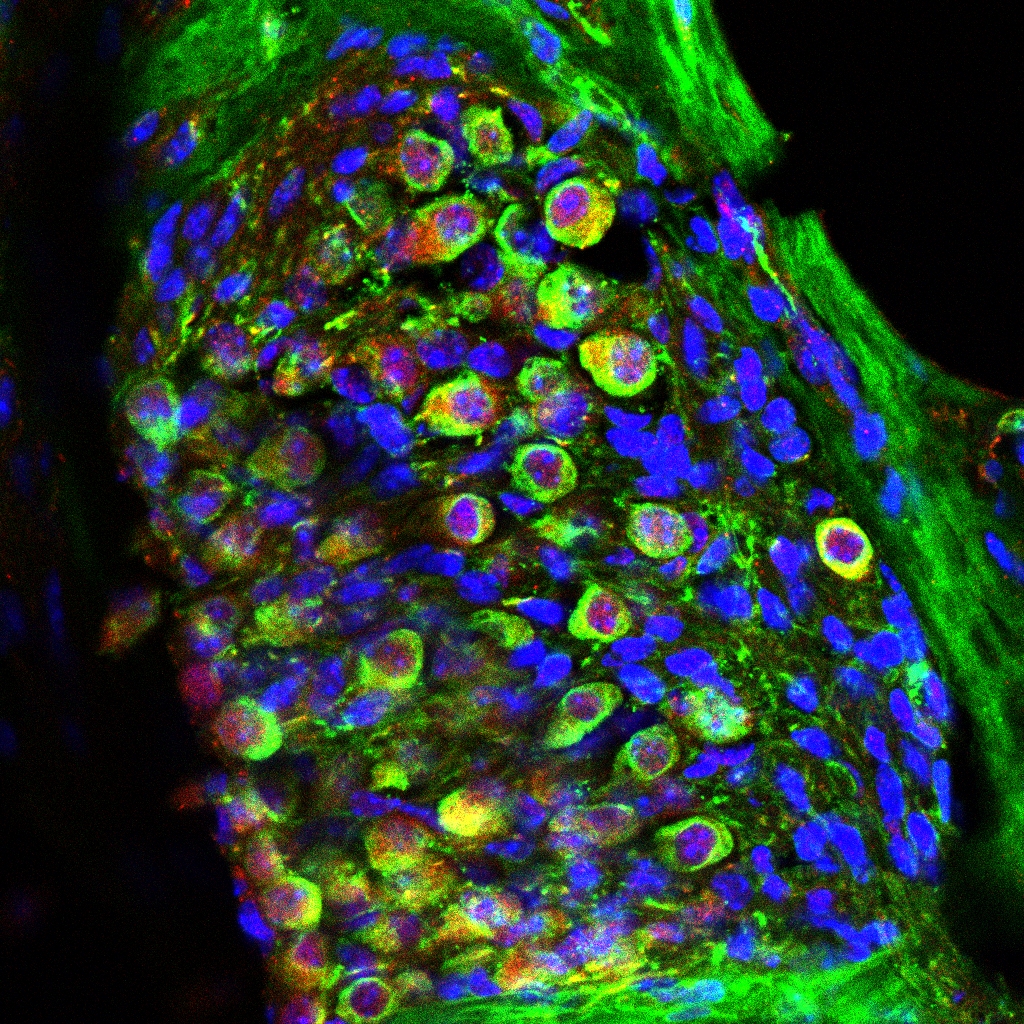

Supplement: Figure 5—figure supplement 1—source data 1. [file elife-76754-fig5-figsupp1-data1.zip › Figure 5 - figure supplement 1 Source data/Figure 5 - figure supplement 1A/WT Merge Middle.tif]

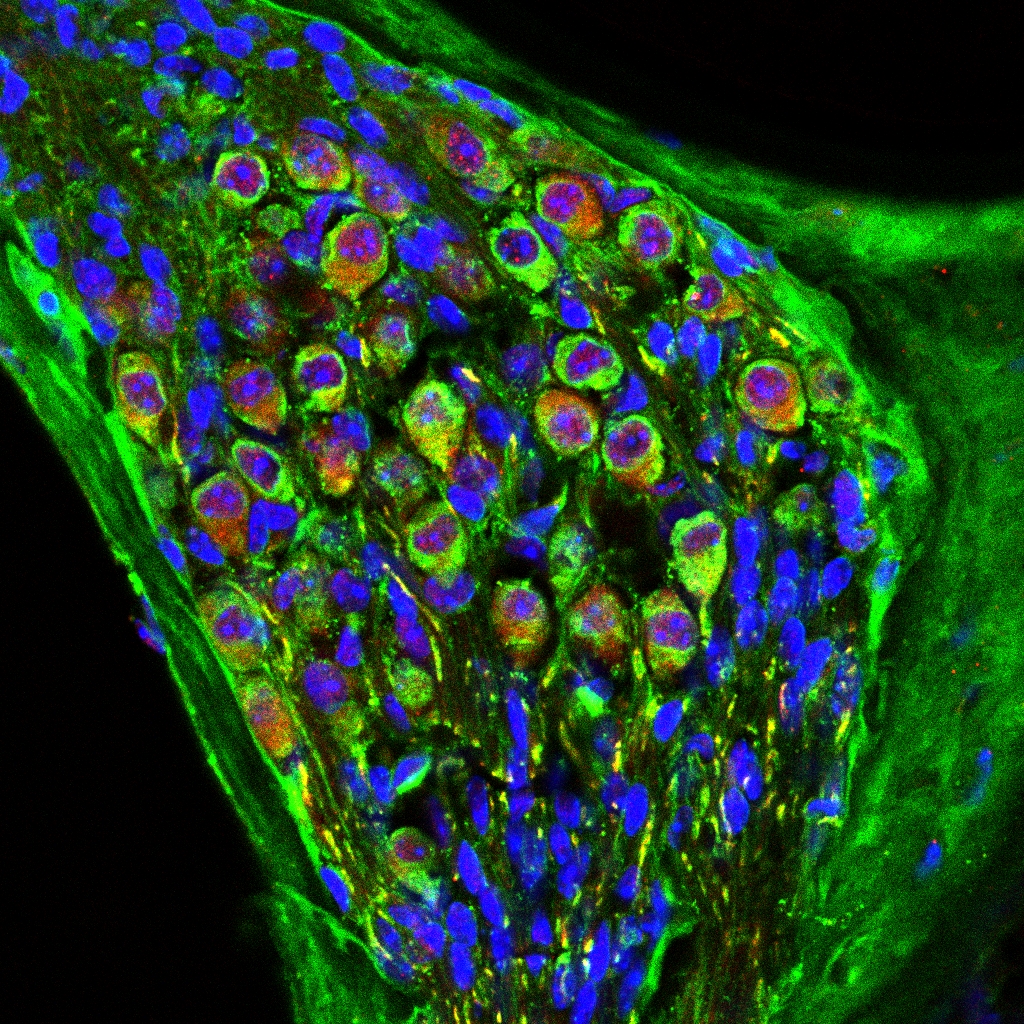

Supplement: Figure 5—figure supplement 1—source data 1. [file elife-76754-fig5-figsupp1-data1.zip › Figure 5 - figure supplement 1 Source data/Figure 5 - figure supplement 1A/WT Merge Base.tif]

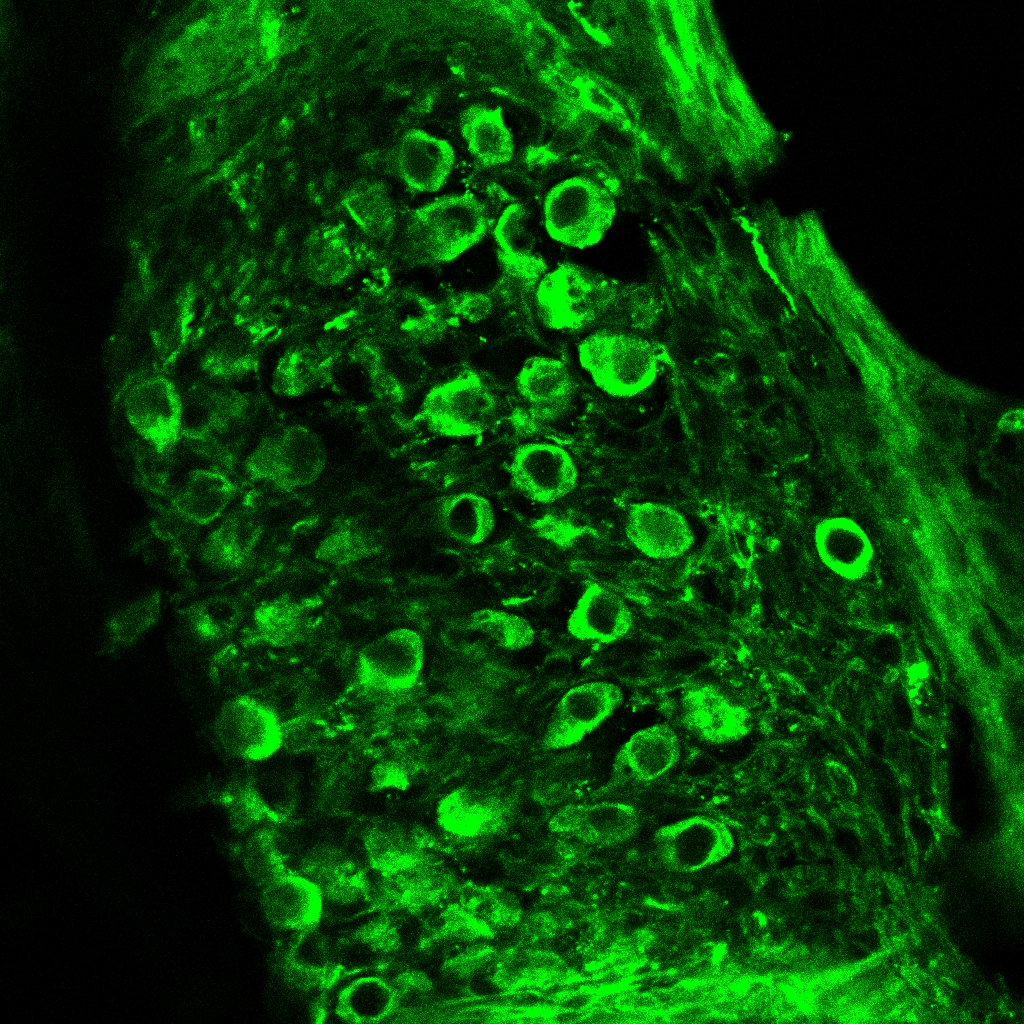

Supplement: Figure 5—figure supplement 1—source data 1. [file elife-76754-fig5-figsupp1-data1.zip › Figure 5 - figure supplement 1 Source data/Figure 5 - figure supplement 1A/WT Tuj1 Middle.tif]

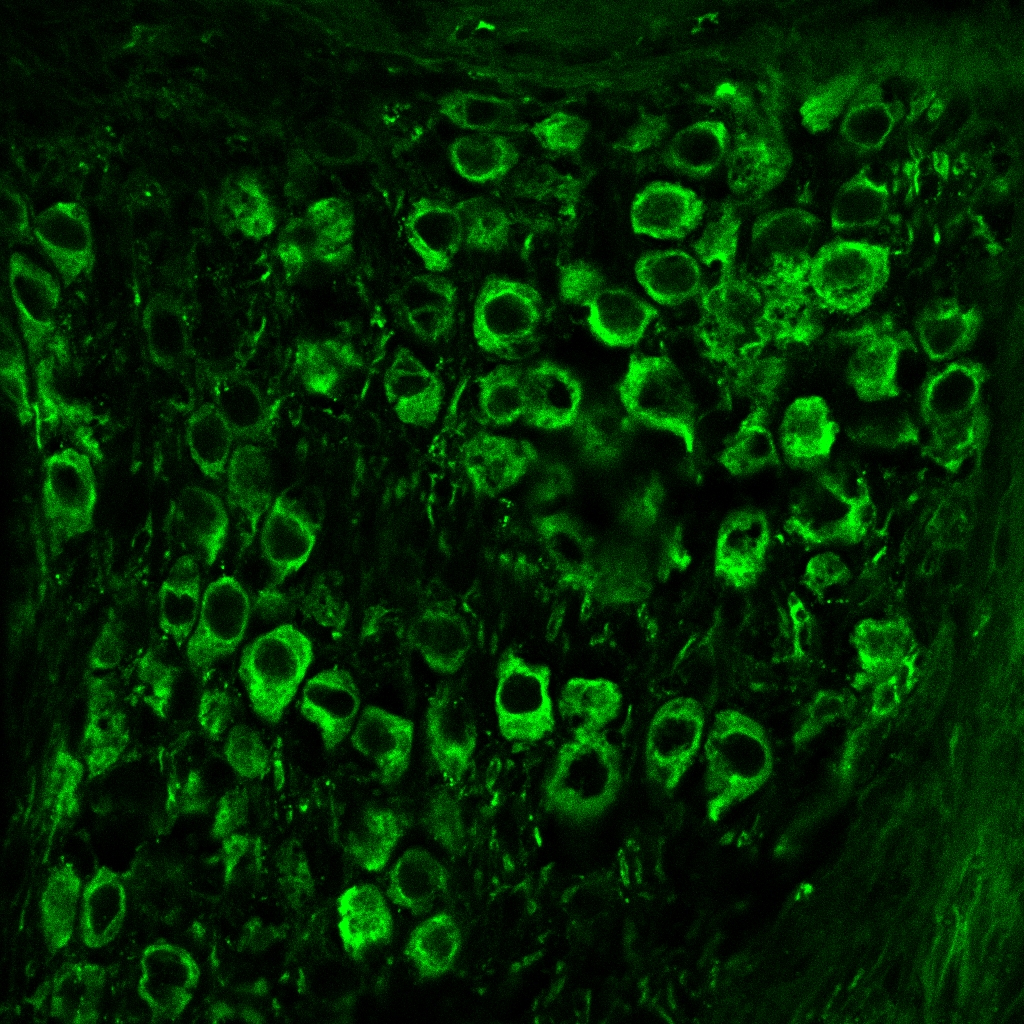

Supplement: Figure 5—figure supplement 1—source data 1. [file elife-76754-fig5-figsupp1-data1.zip › Figure 5 - figure supplement 1 Source data/Figure 5 - figure supplement 1A/WT Tuj1 Apex.tif]

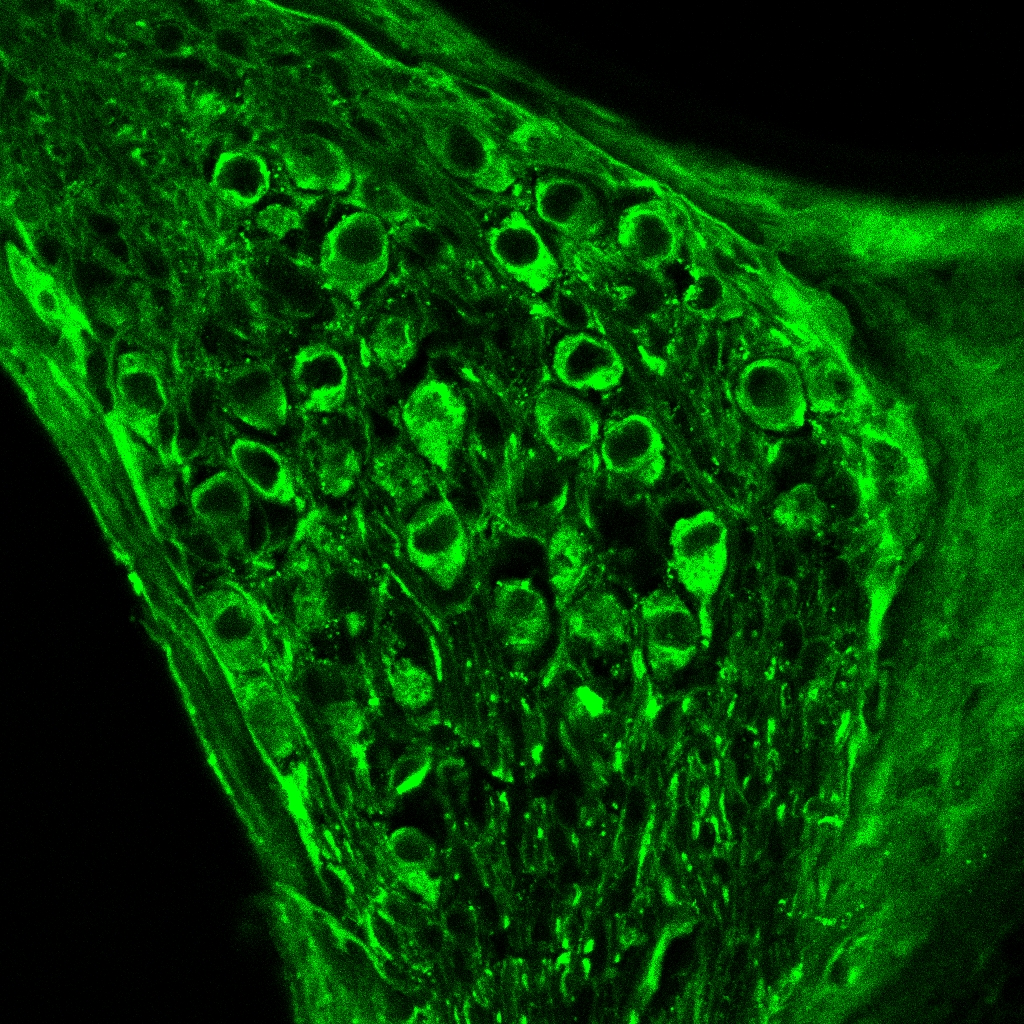

Supplement: Figure 5—figure supplement 1—source data 1. [file elife-76754-fig5-figsupp1-data1.zip › Figure 5 - figure supplement 1 Source data/Figure 5 - figure supplement 1A/WT Tuj1 Base.tif]

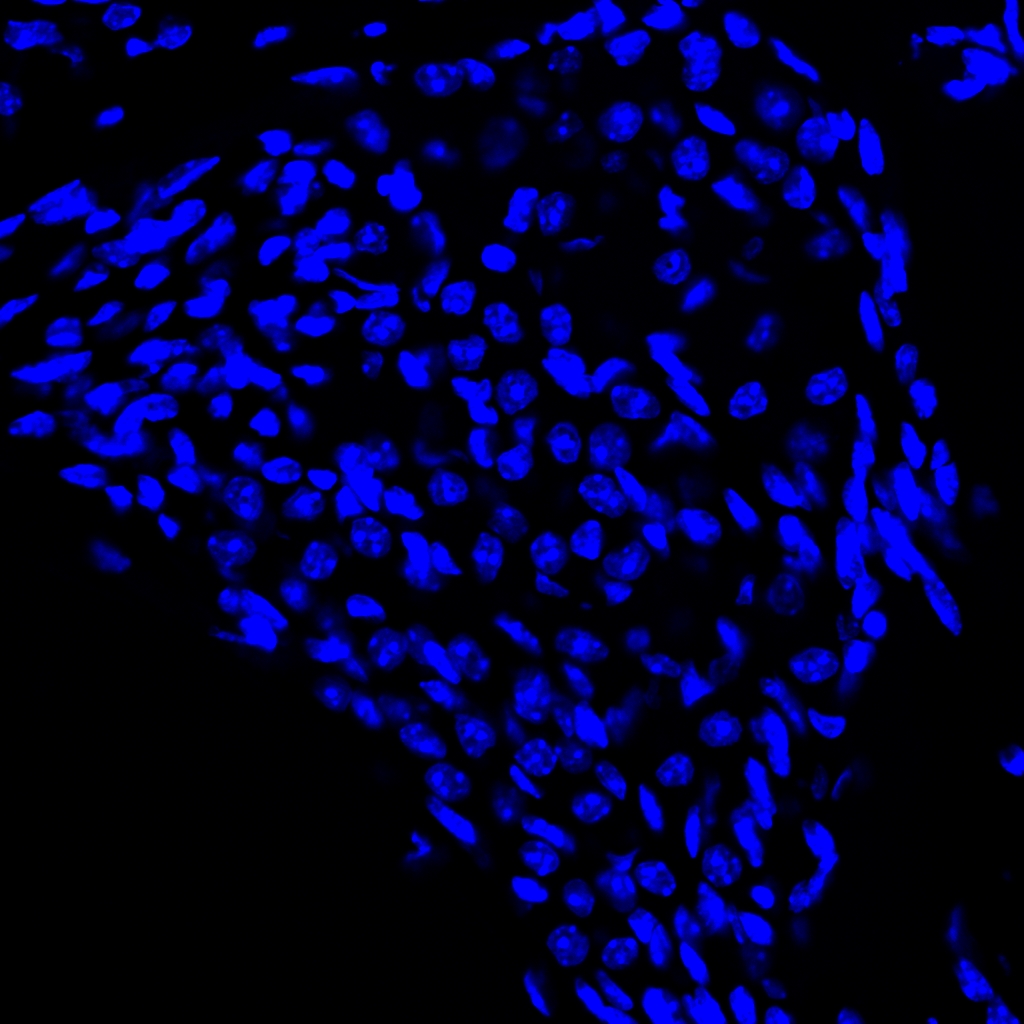

Supplement: Figure 5—figure supplement 1—source data 1. [file elife-76754-fig5-figsupp1-data1.zip › Figure 5 - figure supplement 1 Source data/Figure 5 - figure supplement 1C/Rest cKO DAPI Apex.tif]

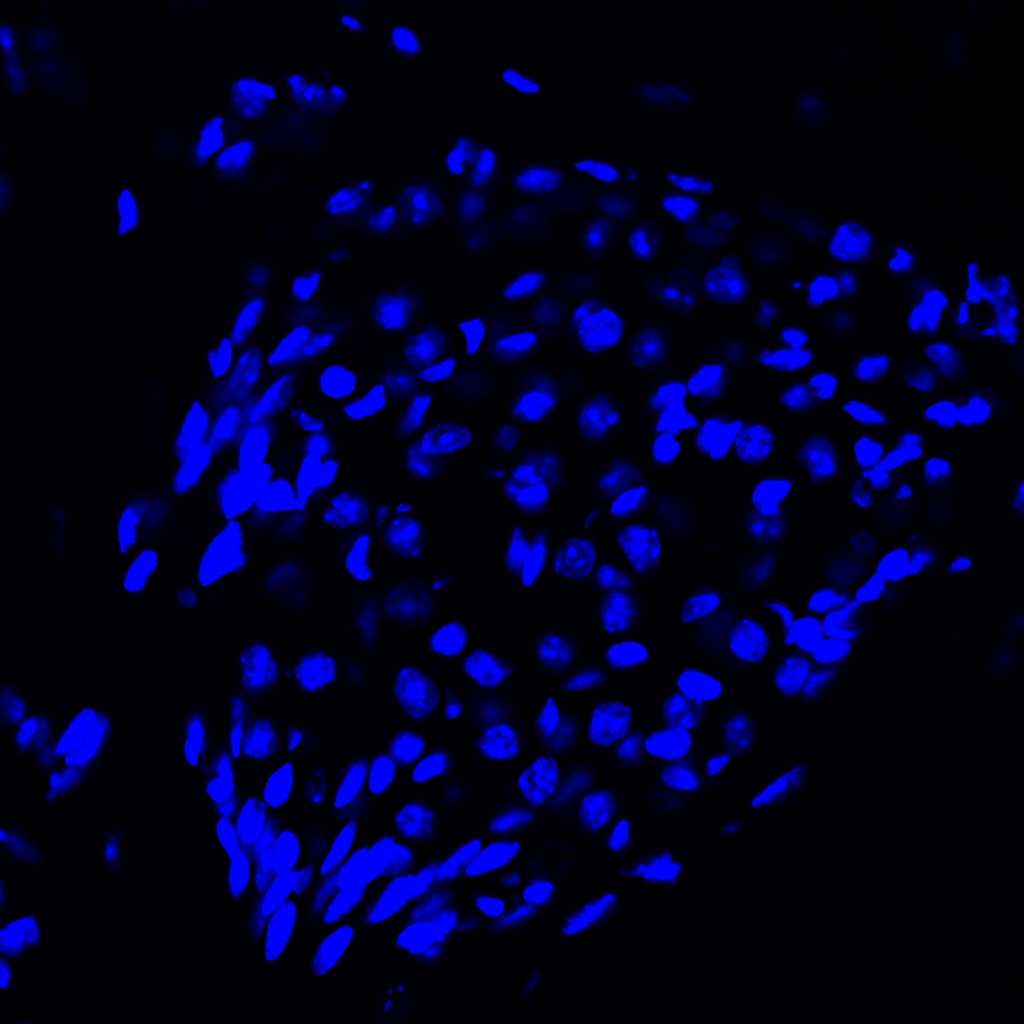

Supplement: Figure 5—figure supplement 1—source data 1. [file elife-76754-fig5-figsupp1-data1.zip › Figure 5 - figure supplement 1 Source data/Figure 5 - figure supplement 1C/Rest cKO DAPI Base.tif]

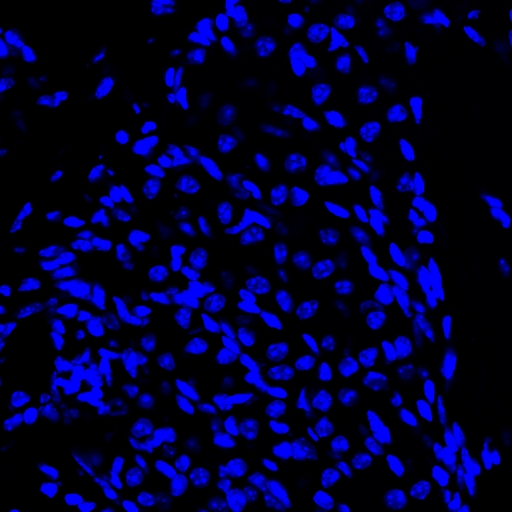

Supplement: Figure 5—figure supplement 1—source data 1. [file elife-76754-fig5-figsupp1-data1.zip › Figure 5 - figure supplement 1 Source data/Figure 5 - figure supplement 1C/Rest cKO DAPI Middle.tif]

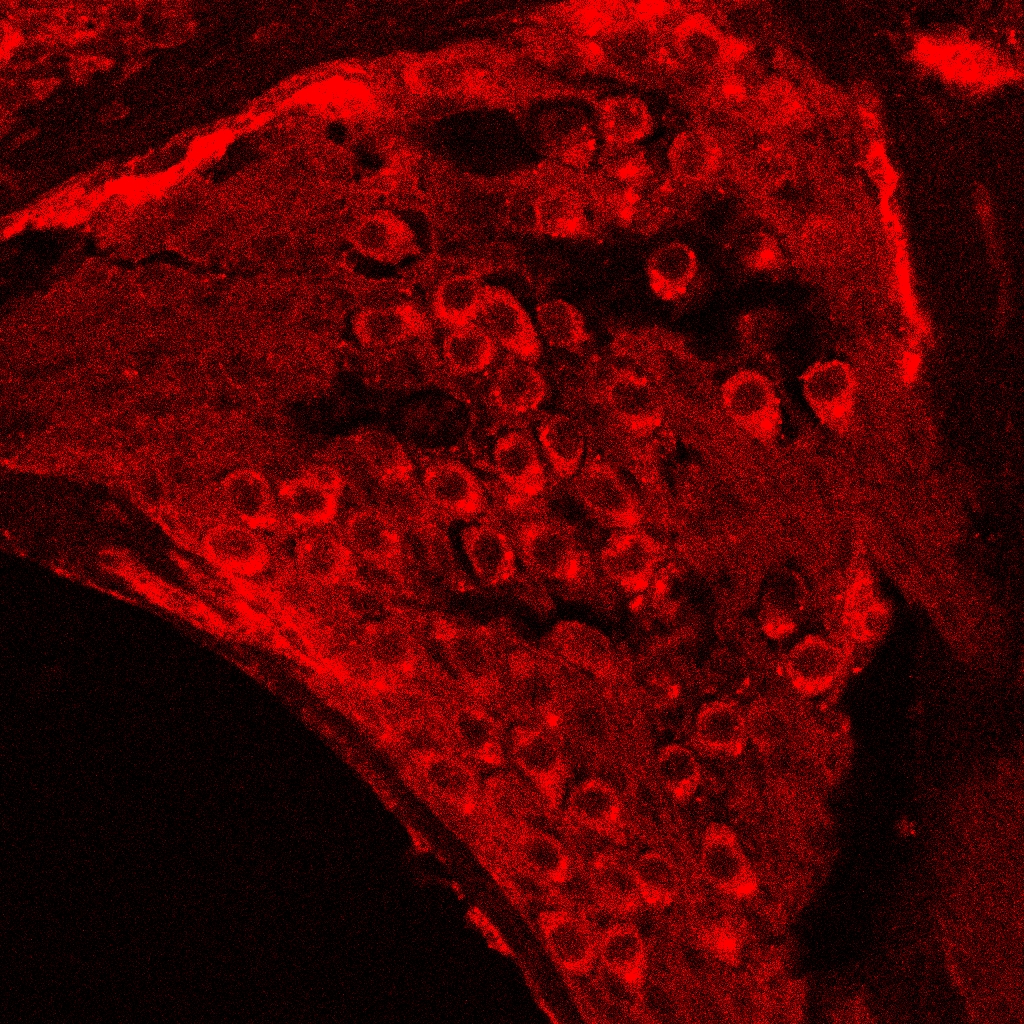

Supplement: Figure 5—figure supplement 1—source data 1. [file elife-76754-fig5-figsupp1-data1.zip › Figure 5 - figure supplement 1 Source data/Figure 5 - figure supplement 1C/Rest cKO Kv7.3 Apex.tif]

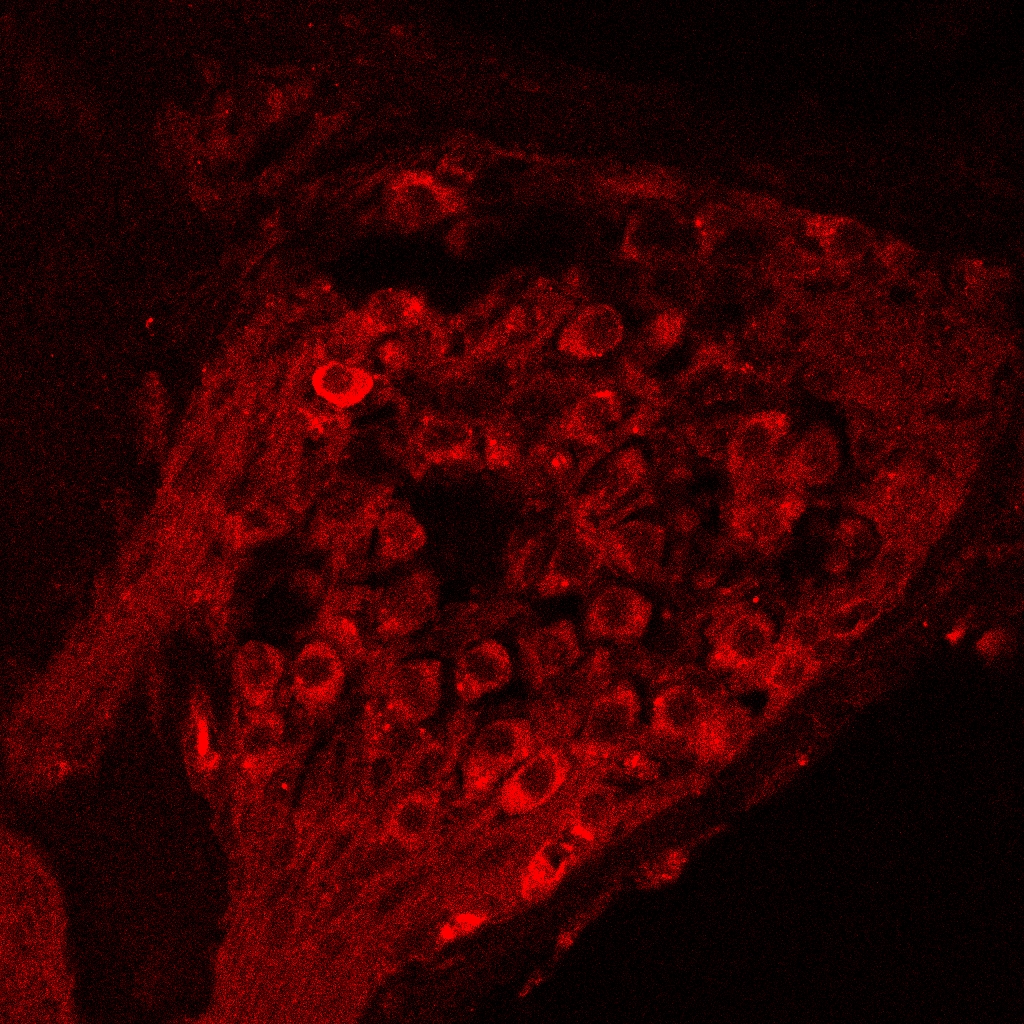

Supplement: Figure 5—figure supplement 1—source data 1. [file elife-76754-fig5-figsupp1-data1.zip › Figure 5 - figure supplement 1 Source data/Figure 5 - figure supplement 1C/Rest cKO Kv7.3 Base.tif]

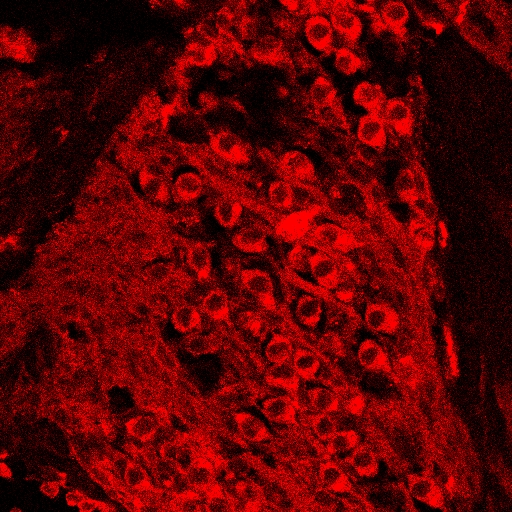

Supplement: Figure 5—figure supplement 1—source data 1. [file elife-76754-fig5-figsupp1-data1.zip › Figure 5 - figure supplement 1 Source data/Figure 5 - figure supplement 1C/Rest cKO Kv7.3 Middle.tif]

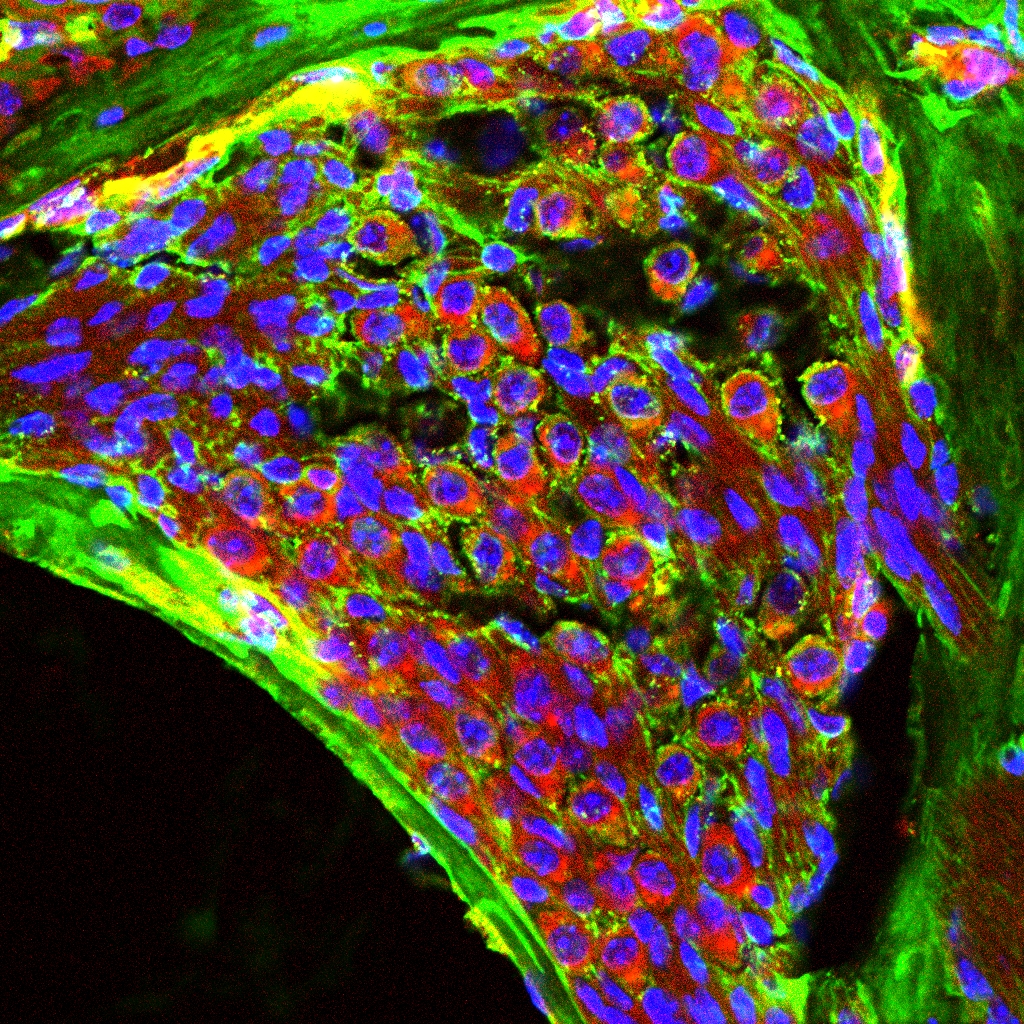

Supplement: Figure 5—figure supplement 1—source data 1. [file elife-76754-fig5-figsupp1-data1.zip › Figure 5 - figure supplement 1 Source data/Figure 5 - figure supplement 1C/Rest cKO Merge Apex.tif]

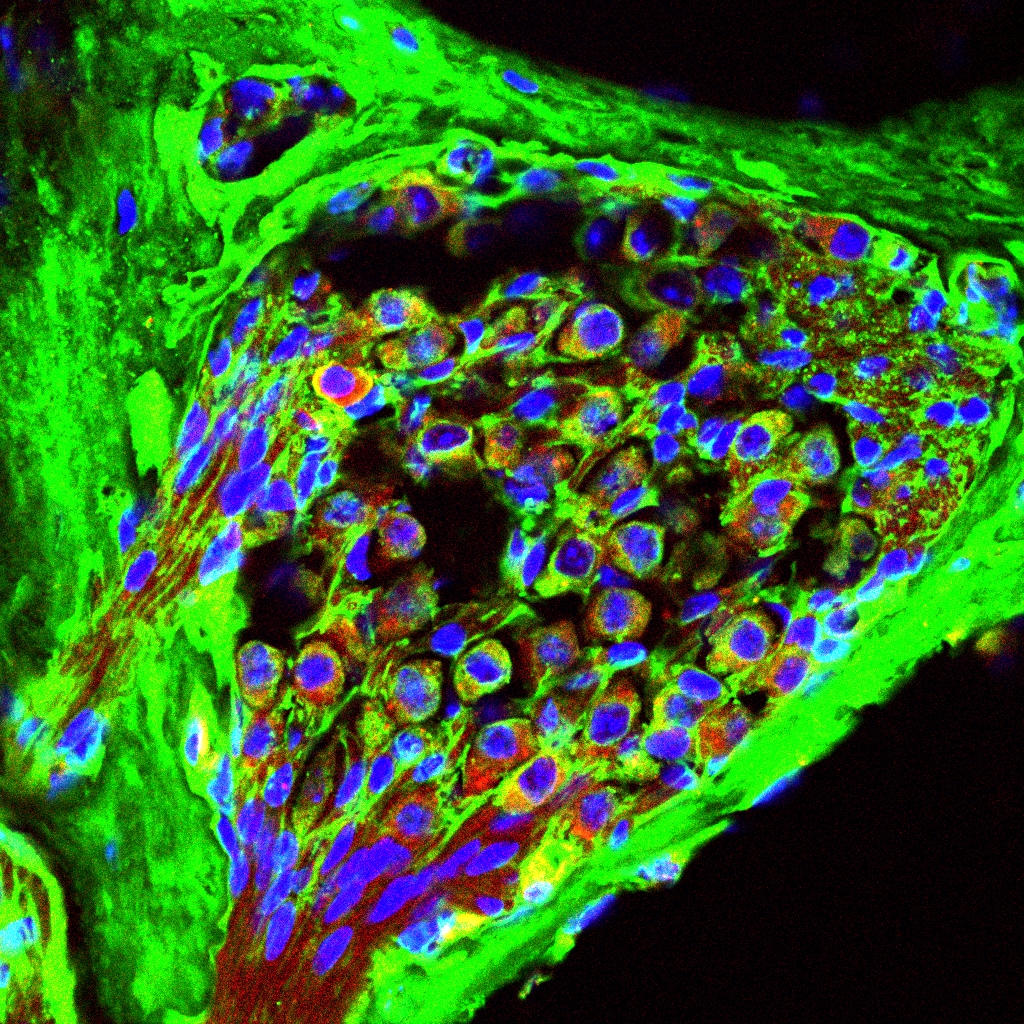

Supplement: Figure 5—figure supplement 1—source data 1. [file elife-76754-fig5-figsupp1-data1.zip › Figure 5 - figure supplement 1 Source data/Figure 5 - figure supplement 1C/Rest cKO Merge Base.tif]

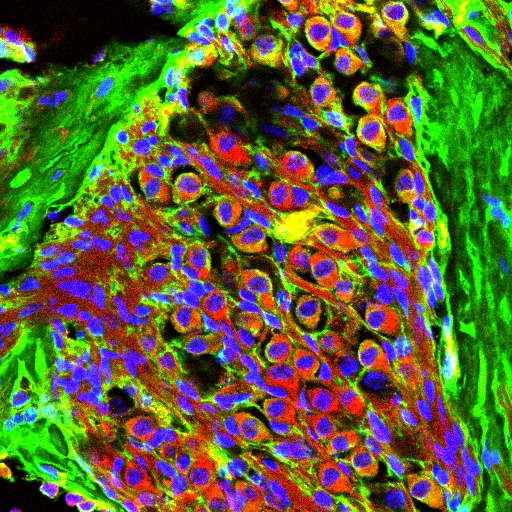

Supplement: Figure 5—figure supplement 1—source data 1. [file elife-76754-fig5-figsupp1-data1.zip › Figure 5 - figure supplement 1 Source data/Figure 5 - figure supplement 1C/Rest cKO Merge Middle.tif]

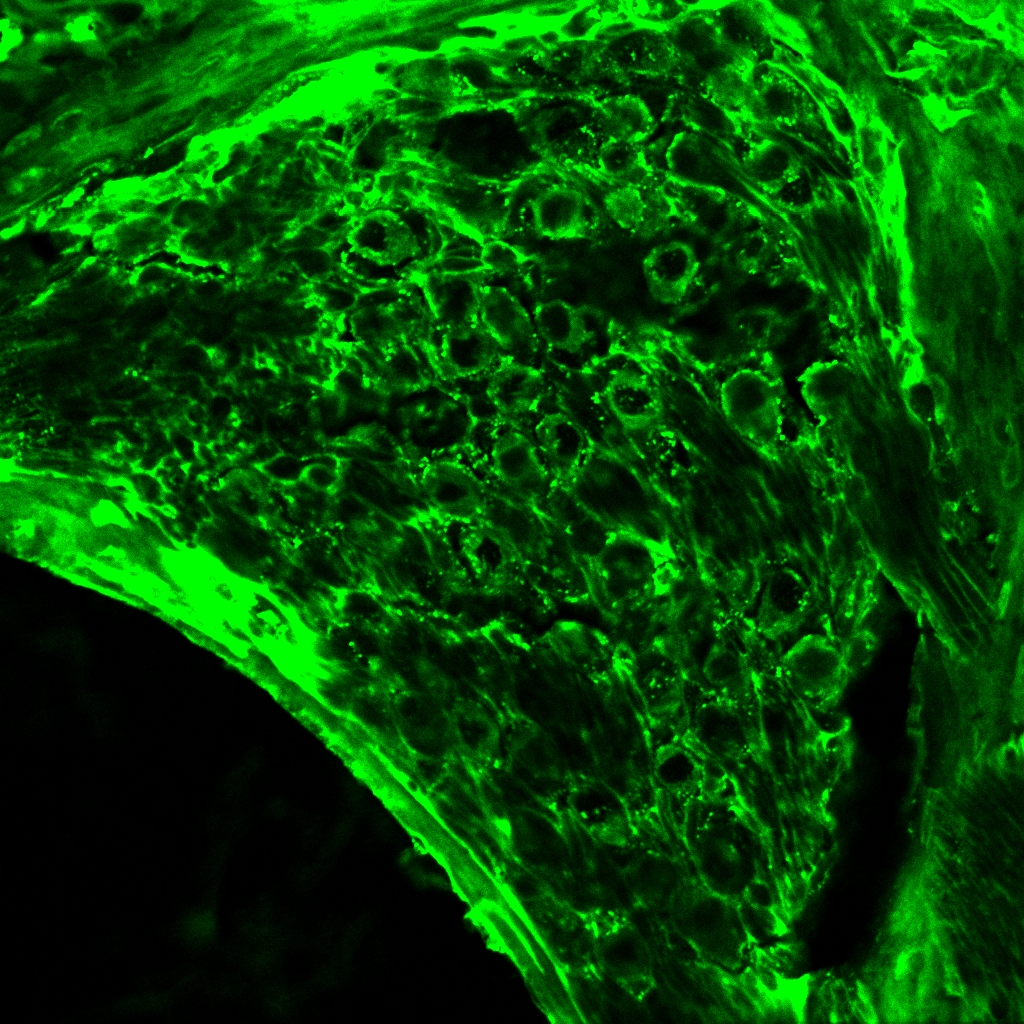

Supplement: Figure 5—figure supplement 1—source data 1. [file elife-76754-fig5-figsupp1-data1.zip › Figure 5 - figure supplement 1 Source data/Figure 5 - figure supplement 1C/Rest cKO Tuj1 Apex.tif]

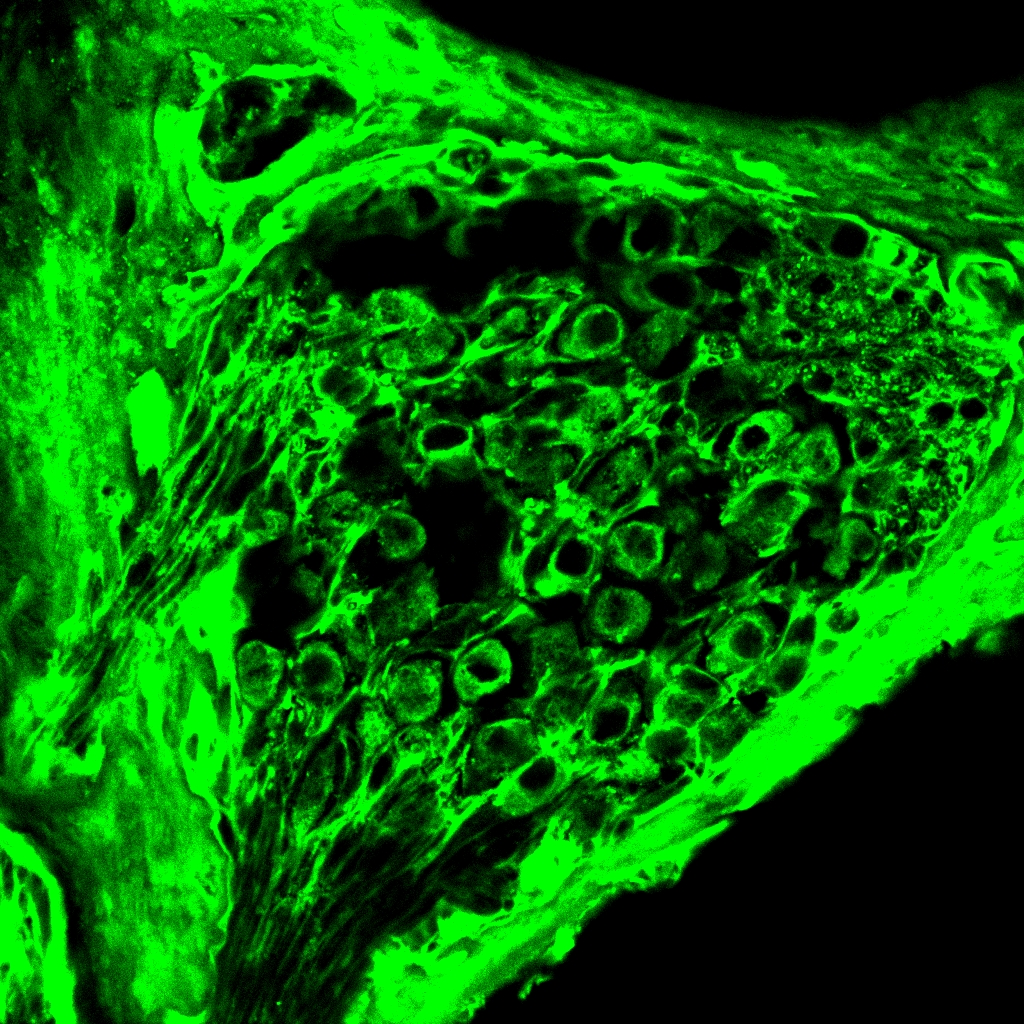

Supplement: Figure 5—figure supplement 1—source data 1. [file elife-76754-fig5-figsupp1-data1.zip › Figure 5 - figure supplement 1 Source data/Figure 5 - figure supplement 1C/Rest cKO Tuj1 Base.tif]

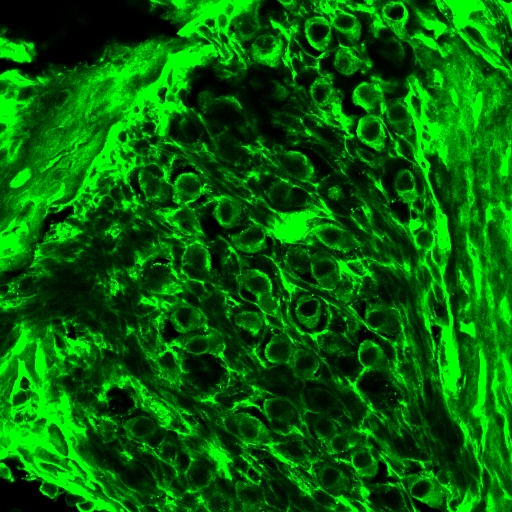

Supplement: Figure 5—figure supplement 1—source data 1. [file elife-76754-fig5-figsupp1-data1.zip › Figure 5 - figure supplement 1 Source data/Figure 5 - figure supplement 1C/Rest cKO Tuj1 Middle.tif]

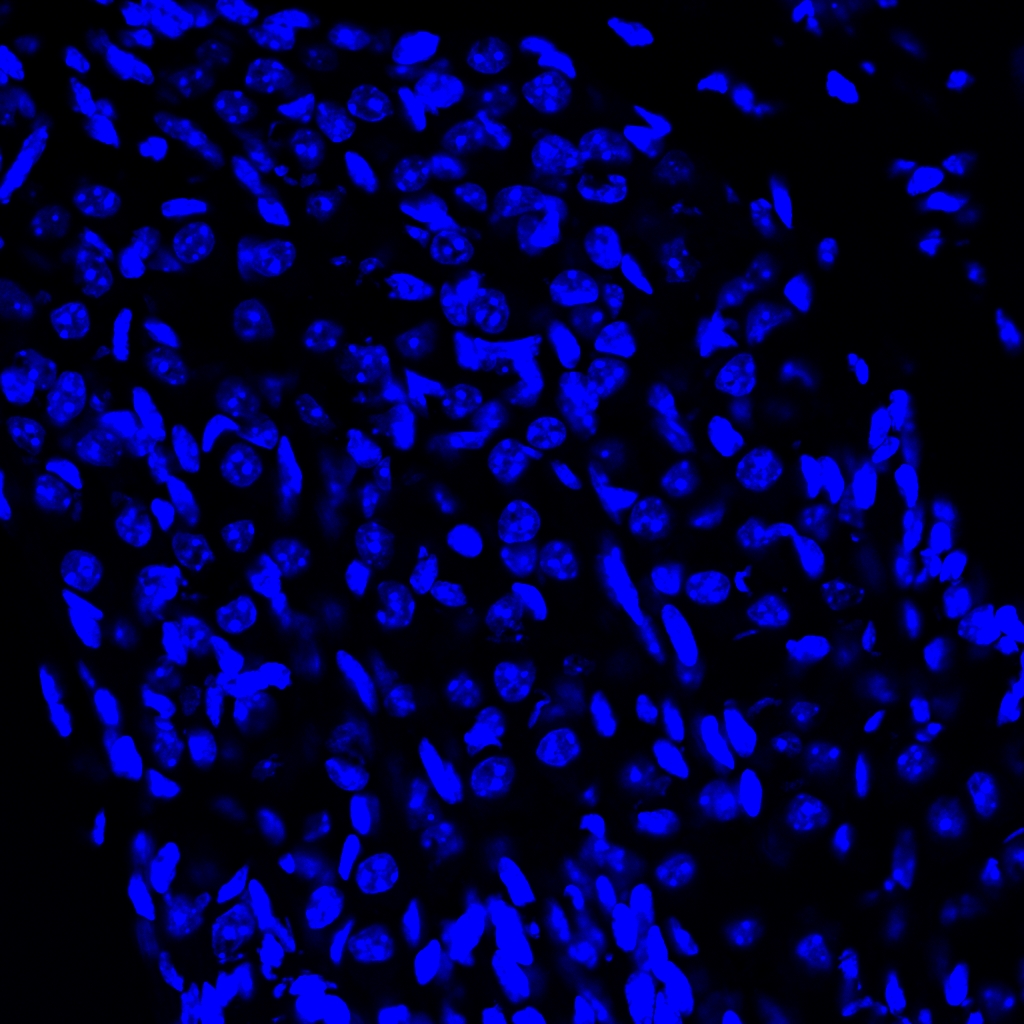

Supplement: Figure 5—figure supplement 1—source data 1. [file elife-76754-fig5-figsupp1-data1.zip › Figure 5 - figure supplement 1 Source data/Figure 5 - figure supplement 1C/WT DAPI Apex.tif]

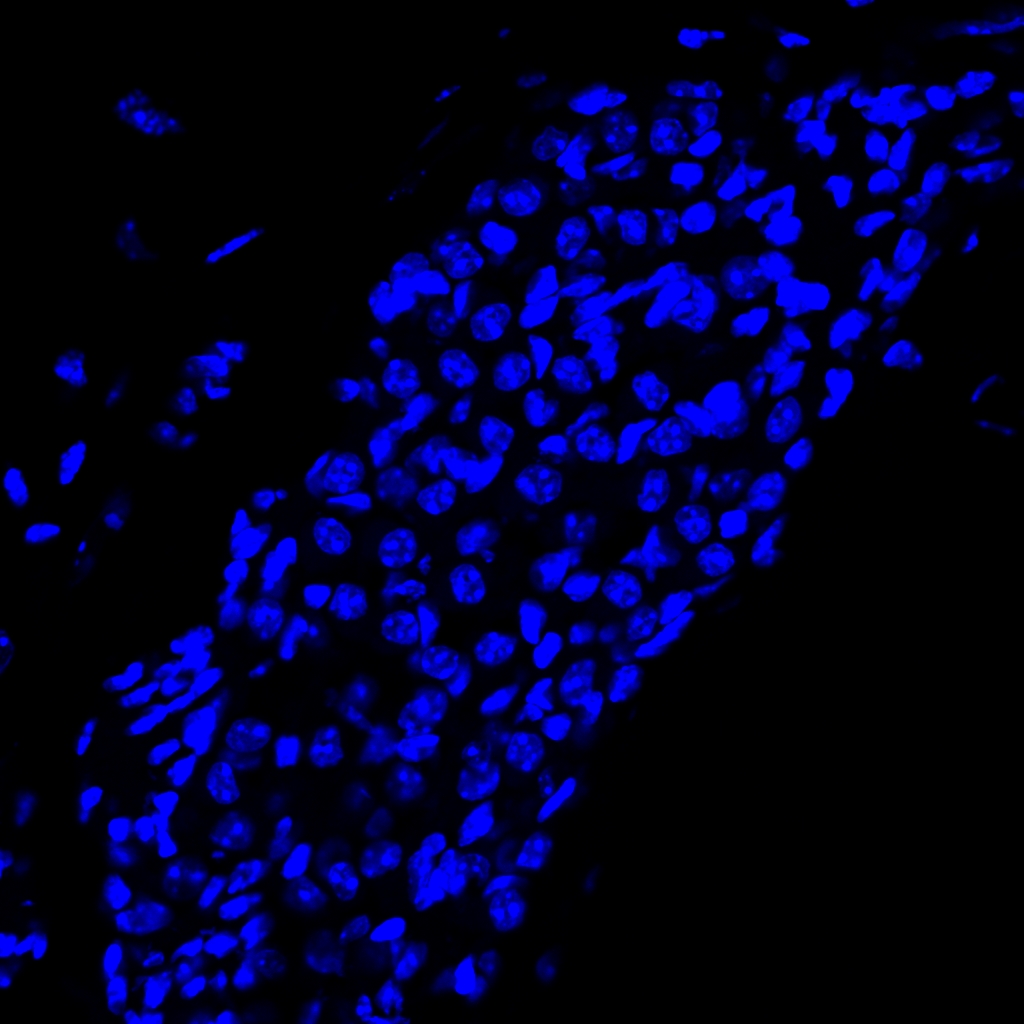

Supplement: Figure 5—figure supplement 1—source data 1. [file elife-76754-fig5-figsupp1-data1.zip › Figure 5 - figure supplement 1 Source data/Figure 5 - figure supplement 1C/WT DAPI Base.tif]

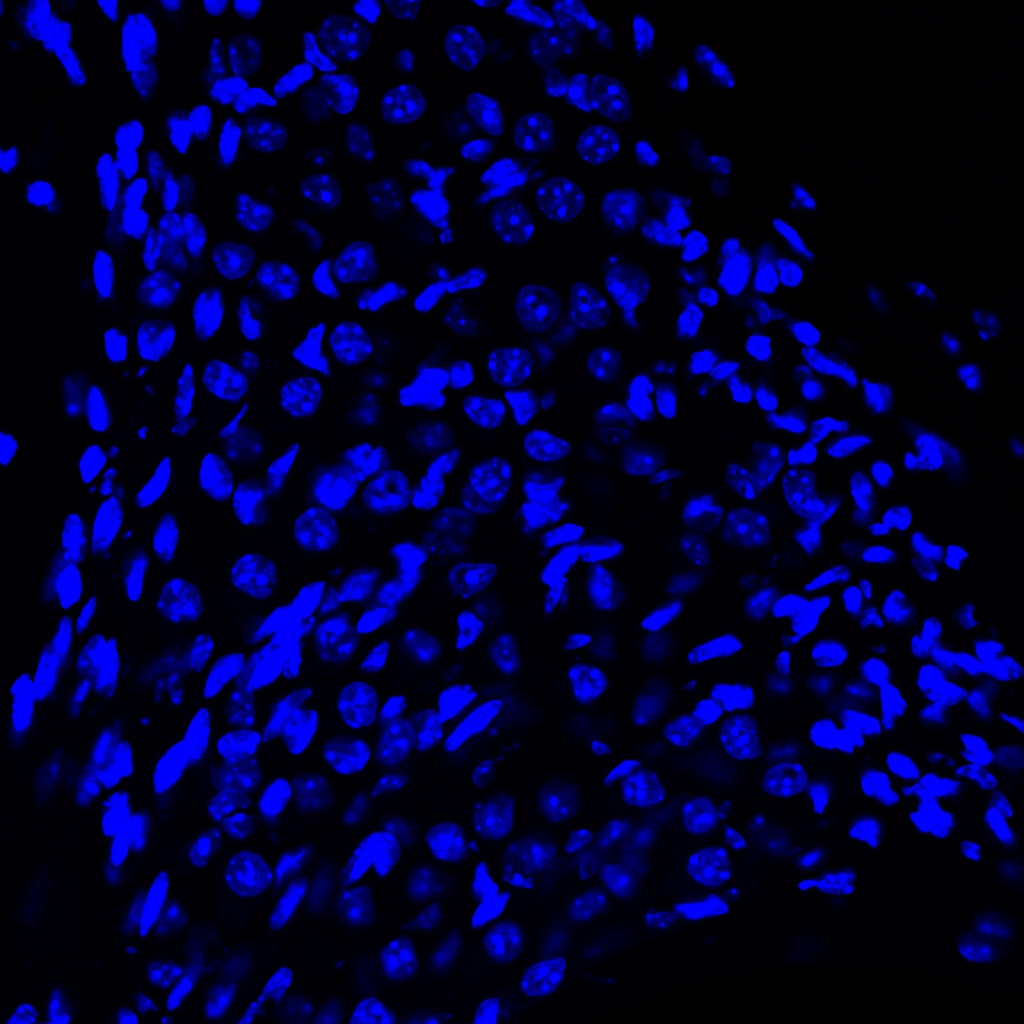

Supplement: Figure 5—figure supplement 1—source data 1. [file elife-76754-fig5-figsupp1-data1.zip › Figure 5 - figure supplement 1 Source data/Figure 5 - figure supplement 1C/WT DAPI Middle.tif]

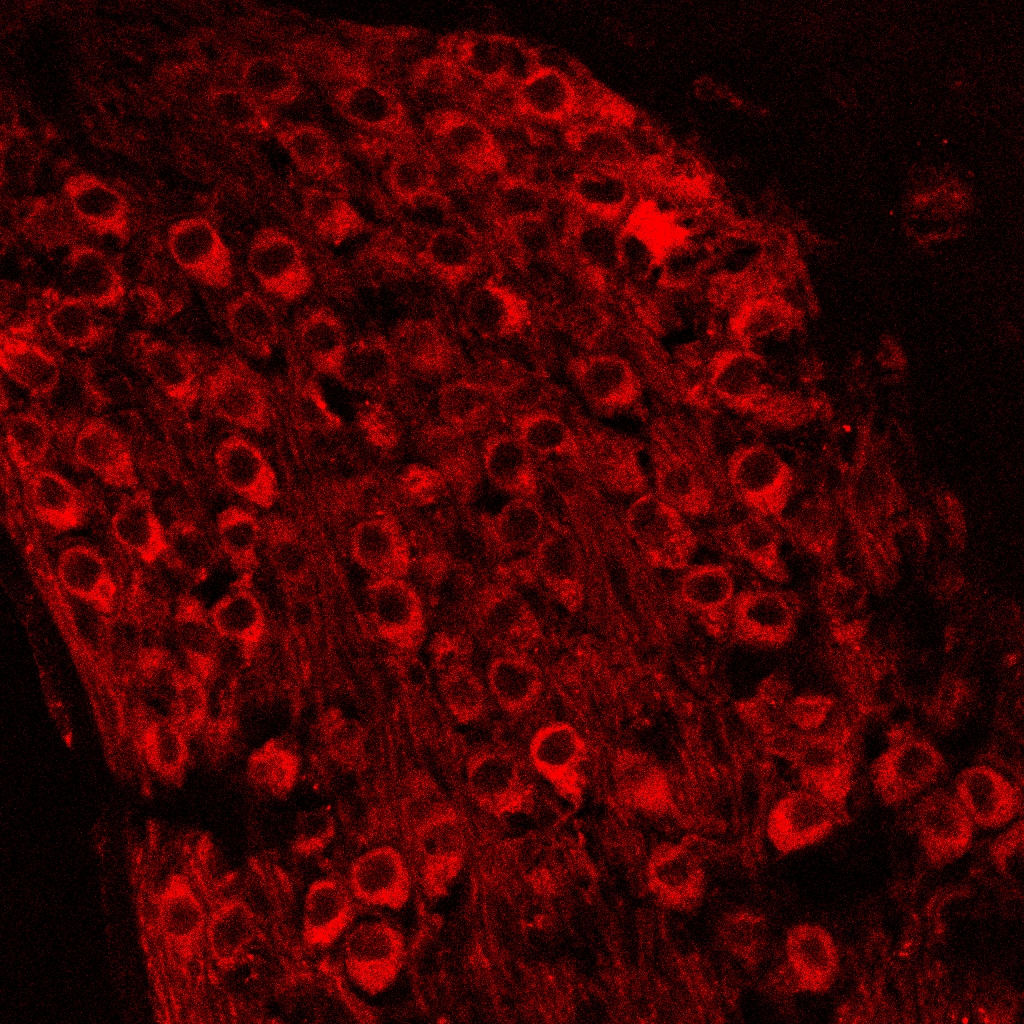

Supplement: Figure 5—figure supplement 1—source data 1. [file elife-76754-fig5-figsupp1-data1.zip › Figure 5 - figure supplement 1 Source data/Figure 5 - figure supplement 1C/WT Kv7.3 Apex.tif]

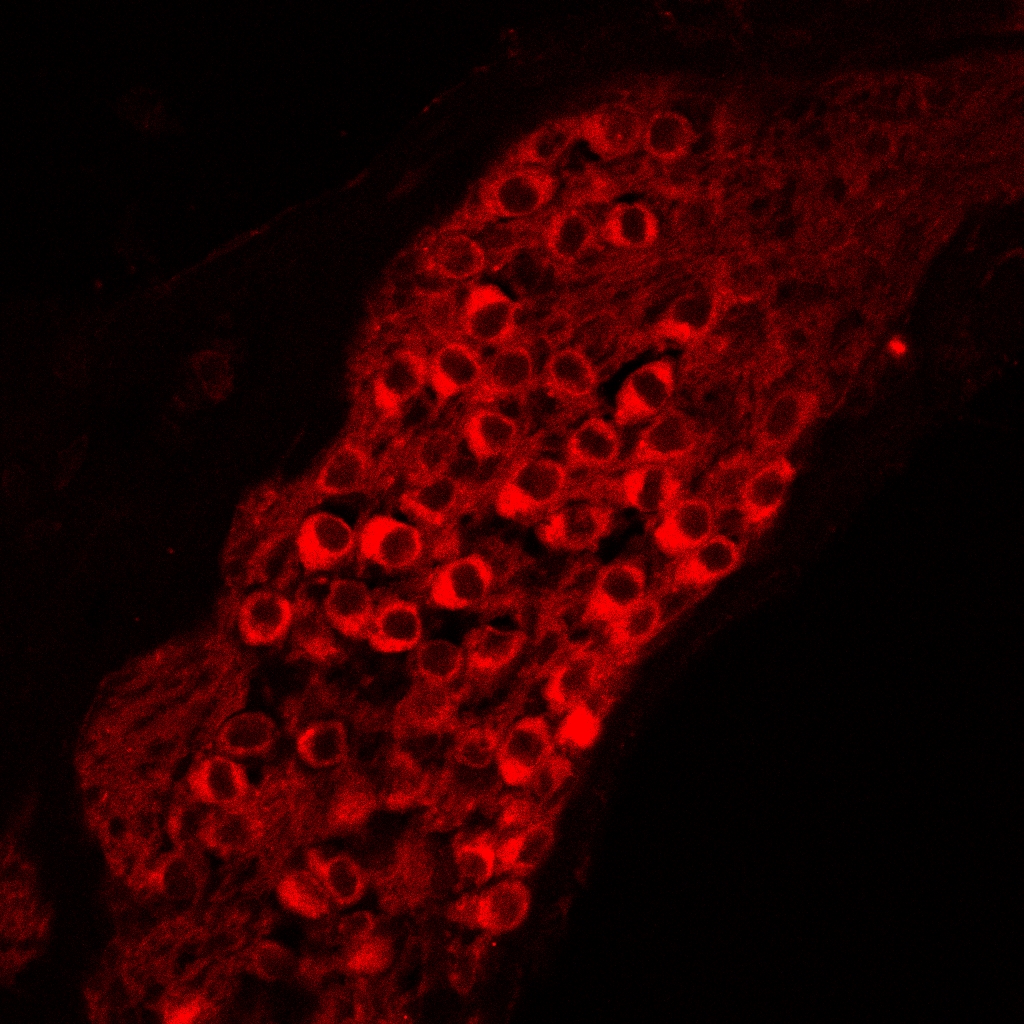

Supplement: Figure 5—figure supplement 1—source data 1. [file elife-76754-fig5-figsupp1-data1.zip › Figure 5 - figure supplement 1 Source data/Figure 5 - figure supplement 1C/WT Kv7.3 Base.tif]

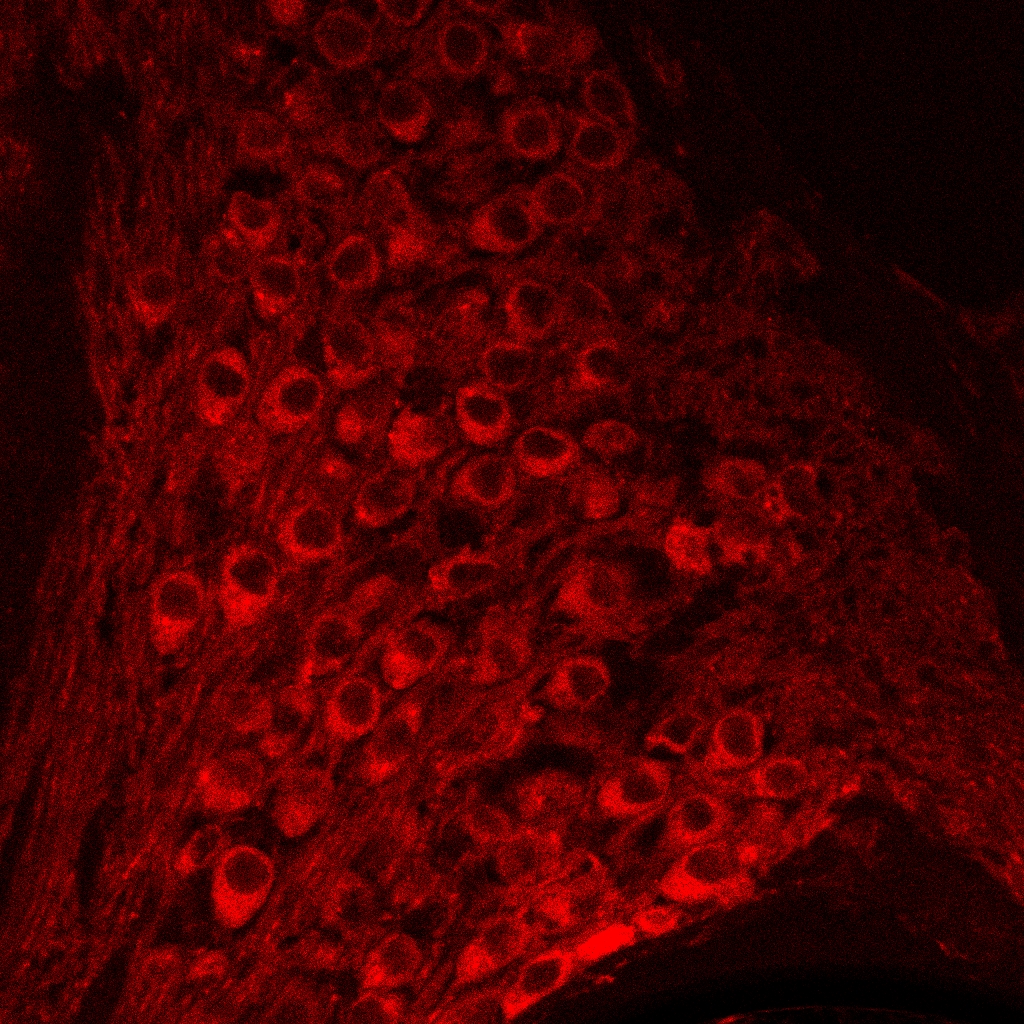

Supplement: Figure 5—figure supplement 1—source data 1. [file elife-76754-fig5-figsupp1-data1.zip › Figure 5 - figure supplement 1 Source data/Figure 5 - figure supplement 1C/WT Kv7.3 Middle.tif]

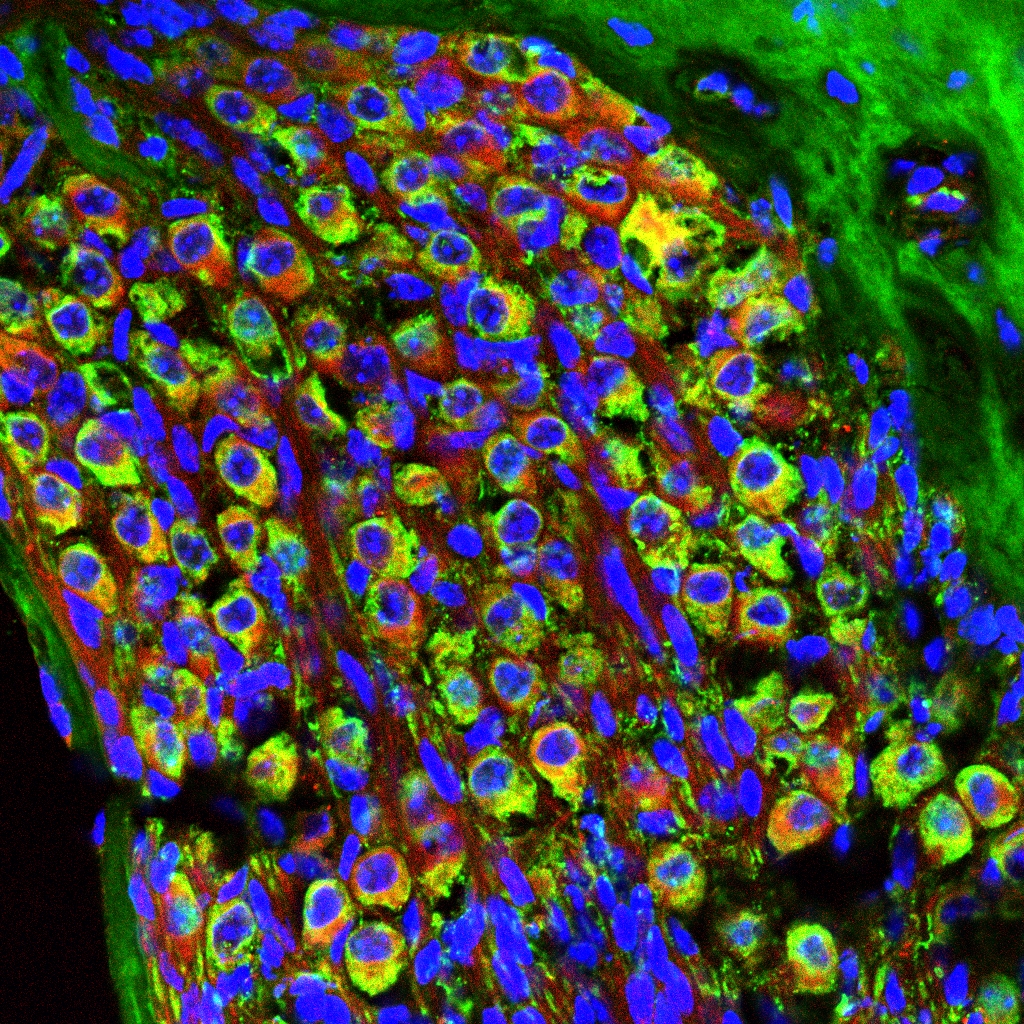

Supplement: Figure 5—figure supplement 1—source data 1. [file elife-76754-fig5-figsupp1-data1.zip › Figure 5 - figure supplement 1 Source data/Figure 5 - figure supplement 1C/WT Merge Apex.tif]
