# Supplementary figures and images for: Repressor element 1-silencing transcription factor deficiency yields profound hearing loss through Kv7.4 channel upsurge in auditory neurons and hair cells (part 3 of 3)
Source: eLife. 2022 Sep 20;11:e76754. doi: 10.7554/eLife.76754 (PMC9525063; doi:10.7554/eLife.76754)

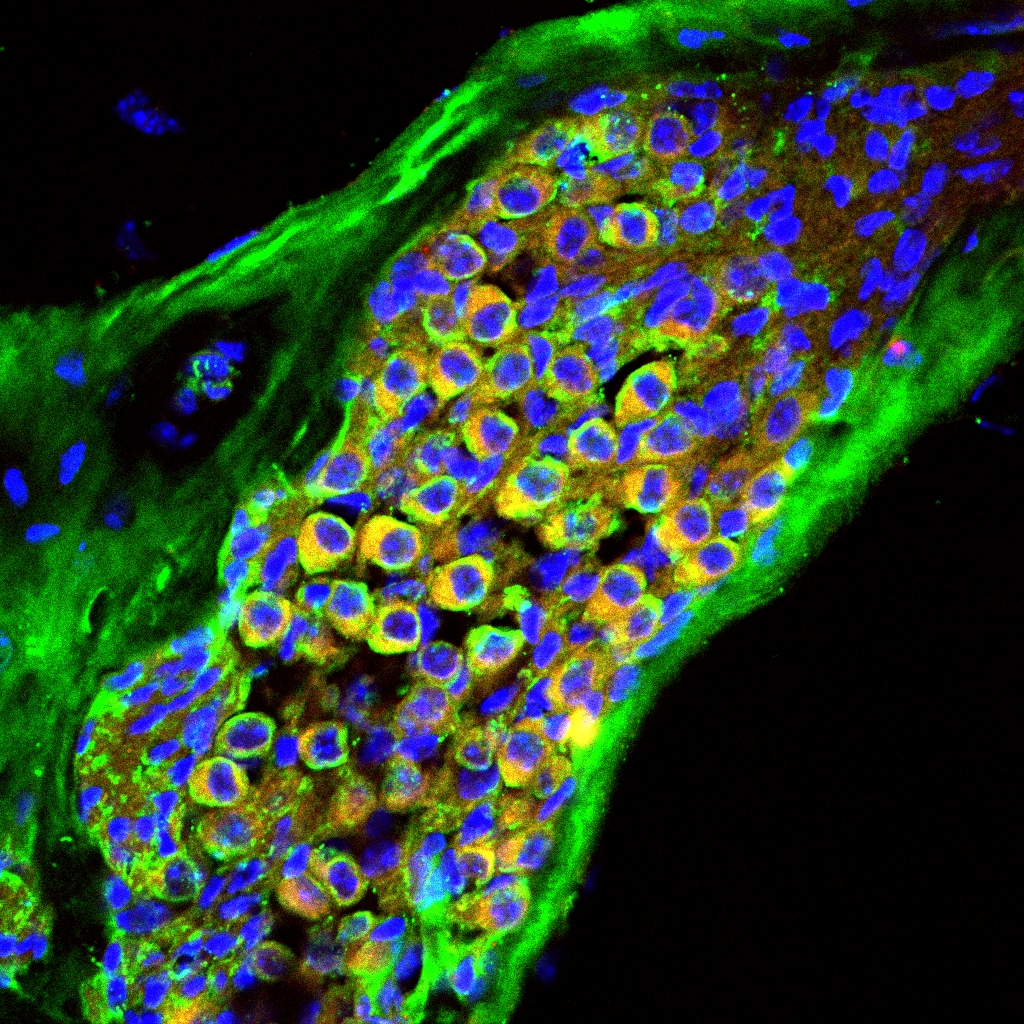

Supplement: Figure 5—figure supplement 1—source data 1. [file elife-76754-fig5-figsupp1-data1.zip › Figure 5 - figure supplement 1 Source data/Figure 5 - figure supplement 1C/WT Merge Base.tif]

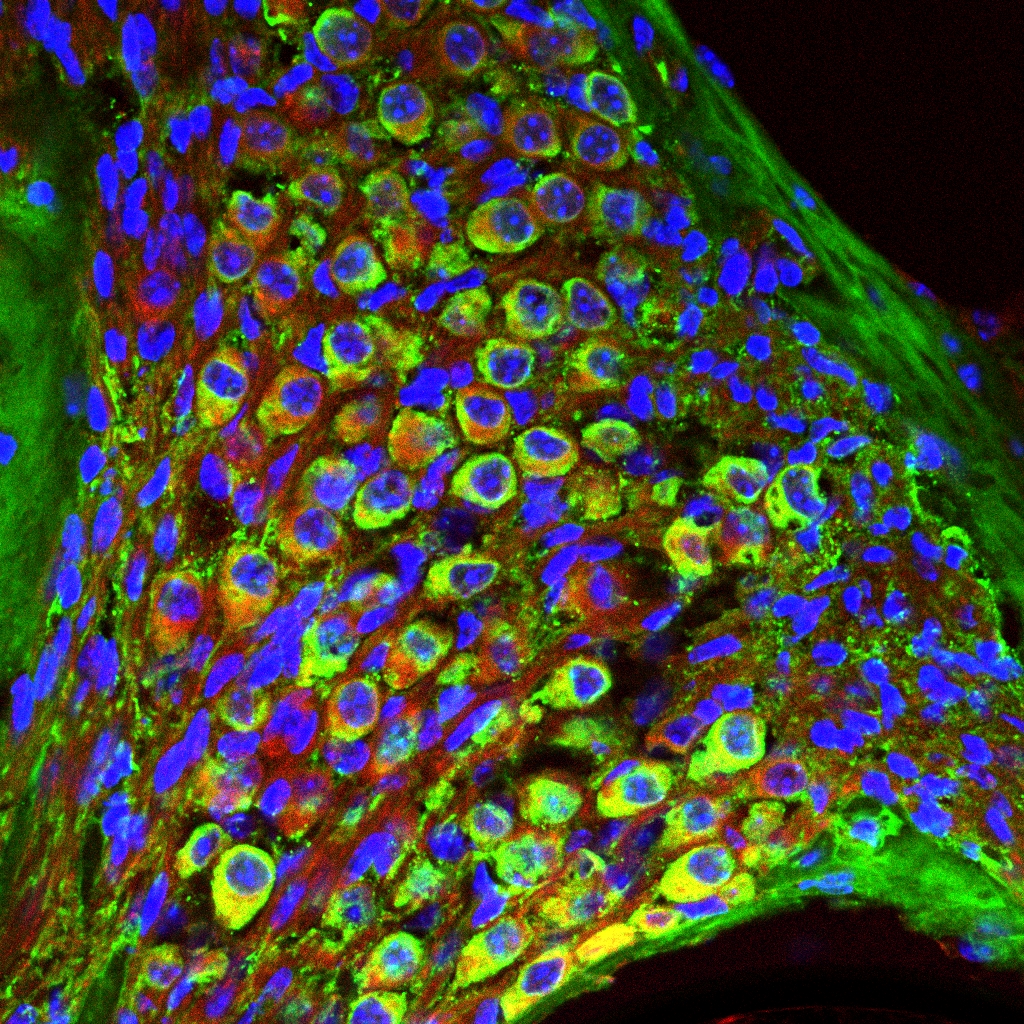

Supplement: Figure 5—figure supplement 1—source data 1. [file elife-76754-fig5-figsupp1-data1.zip › Figure 5 - figure supplement 1 Source data/Figure 5 - figure supplement 1C/WT Merge Middle.tif]

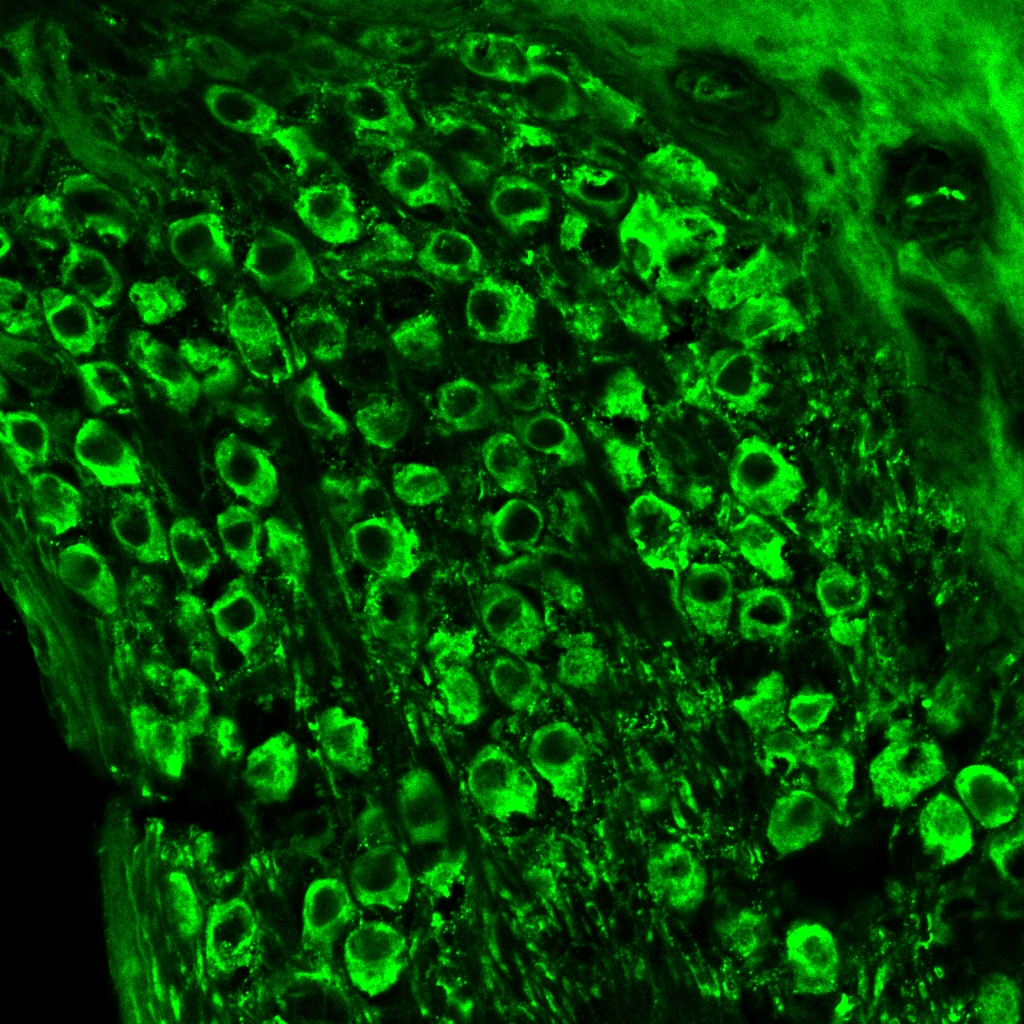

Supplement: Figure 5—figure supplement 1—source data 1. [file elife-76754-fig5-figsupp1-data1.zip › Figure 5 - figure supplement 1 Source data/Figure 5 - figure supplement 1C/WT Tuj1 Apex.tif]

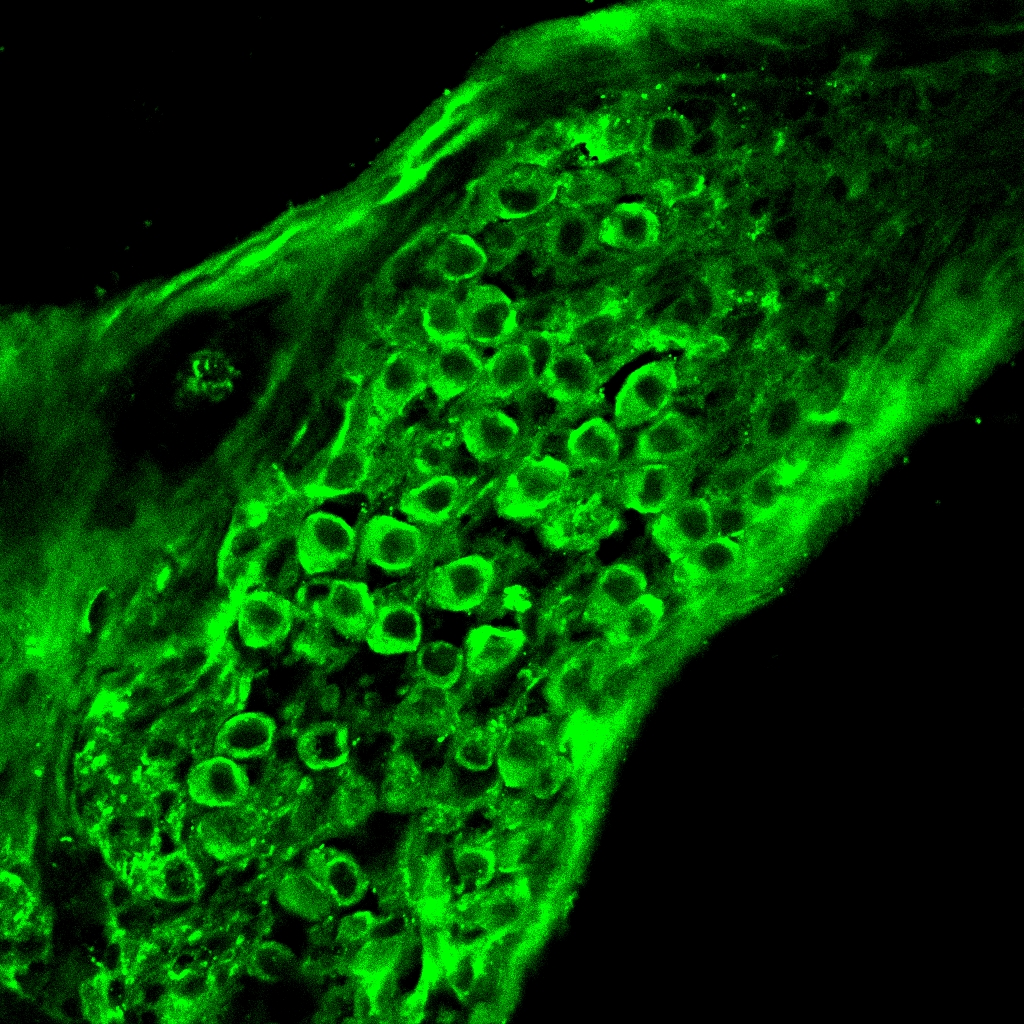

Supplement: Figure 5—figure supplement 1—source data 1. [file elife-76754-fig5-figsupp1-data1.zip › Figure 5 - figure supplement 1 Source data/Figure 5 - figure supplement 1C/WT Tuj1 Base.tif]

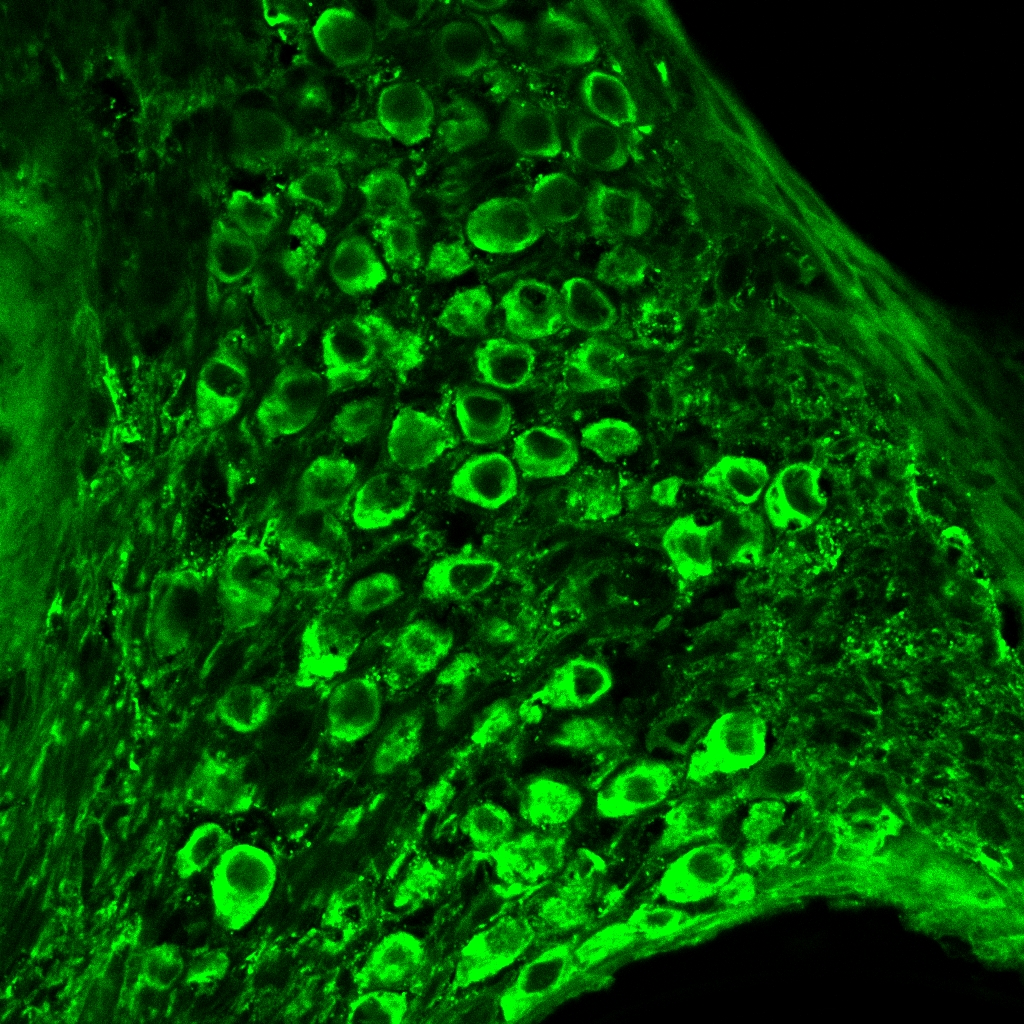

Supplement: Figure 5—figure supplement 1—source data 1. [file elife-76754-fig5-figsupp1-data1.zip › Figure 5 - figure supplement 1 Source data/Figure 5 - figure supplement 1C/WT Tuj1 Middle.tif]

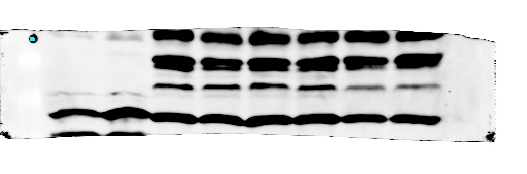

Supplement: Figure 5—figure supplement 2—source data 1. [file elife-76754-fig5-figsupp2-data1.zip › Figure 5 - figure supplement 2 Source data/Figure_5-figure supplement 2 C- Kv7.4 and Tublin protein expression(original).tif]

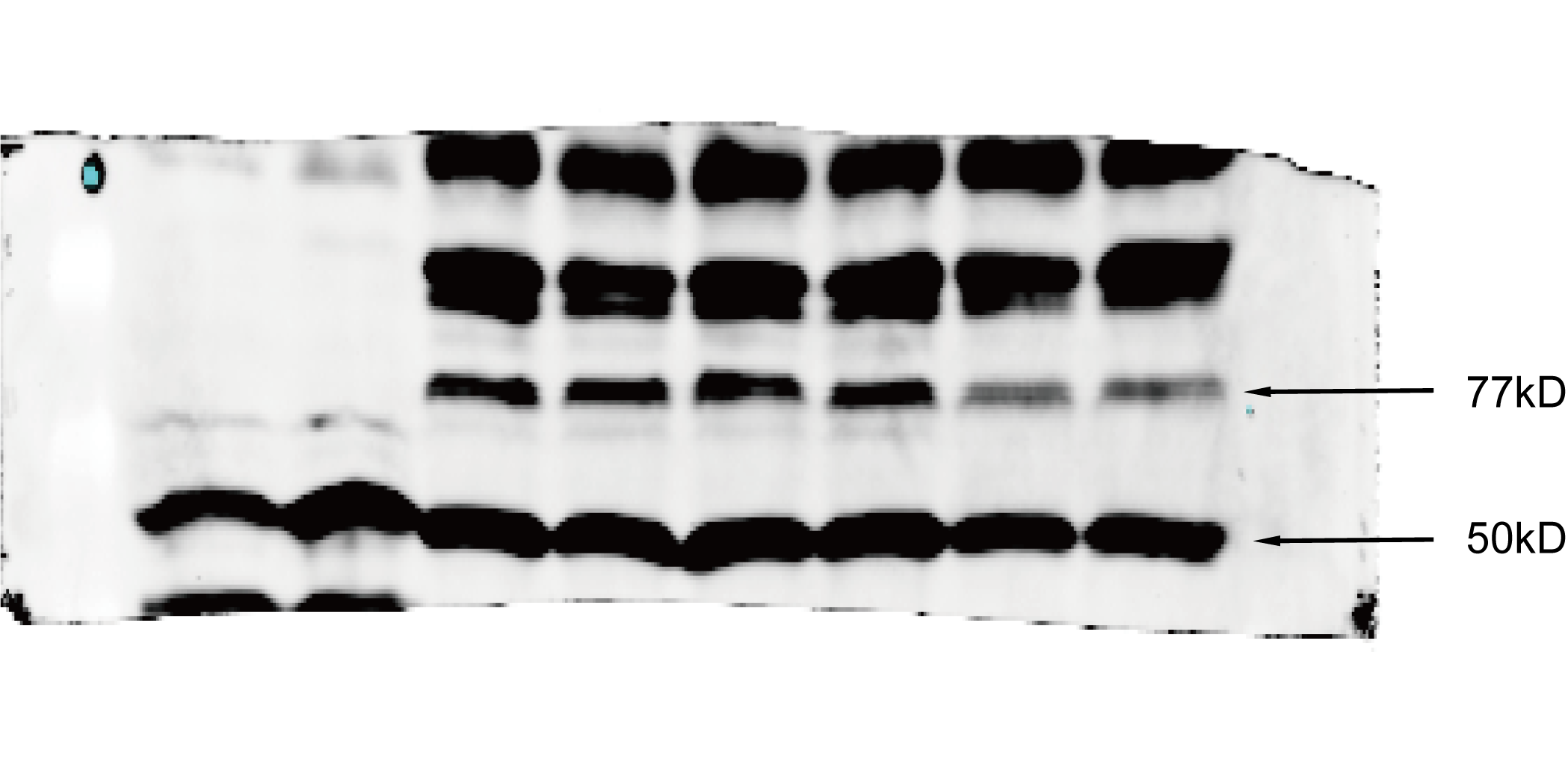

Supplement: Figure 5—figure supplement 2—source data 1. [file elife-76754-fig5-figsupp2-data1.zip › Figure 5 - figure supplement 2 Source data/Figure_5-figure supplement 2 C- Kv7.4 and Tublin protein expression.tif]

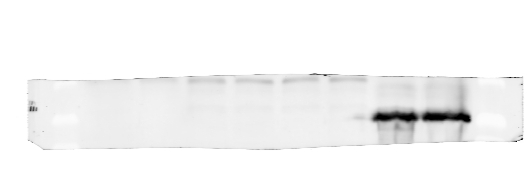

Supplement: Figure 5—figure supplement 2—source data 1. [file elife-76754-fig5-figsupp2-data1.zip › Figure 5 - figure supplement 2 Source data/Figure_5-figure supplement 2C-REST protein expression(original).tif]

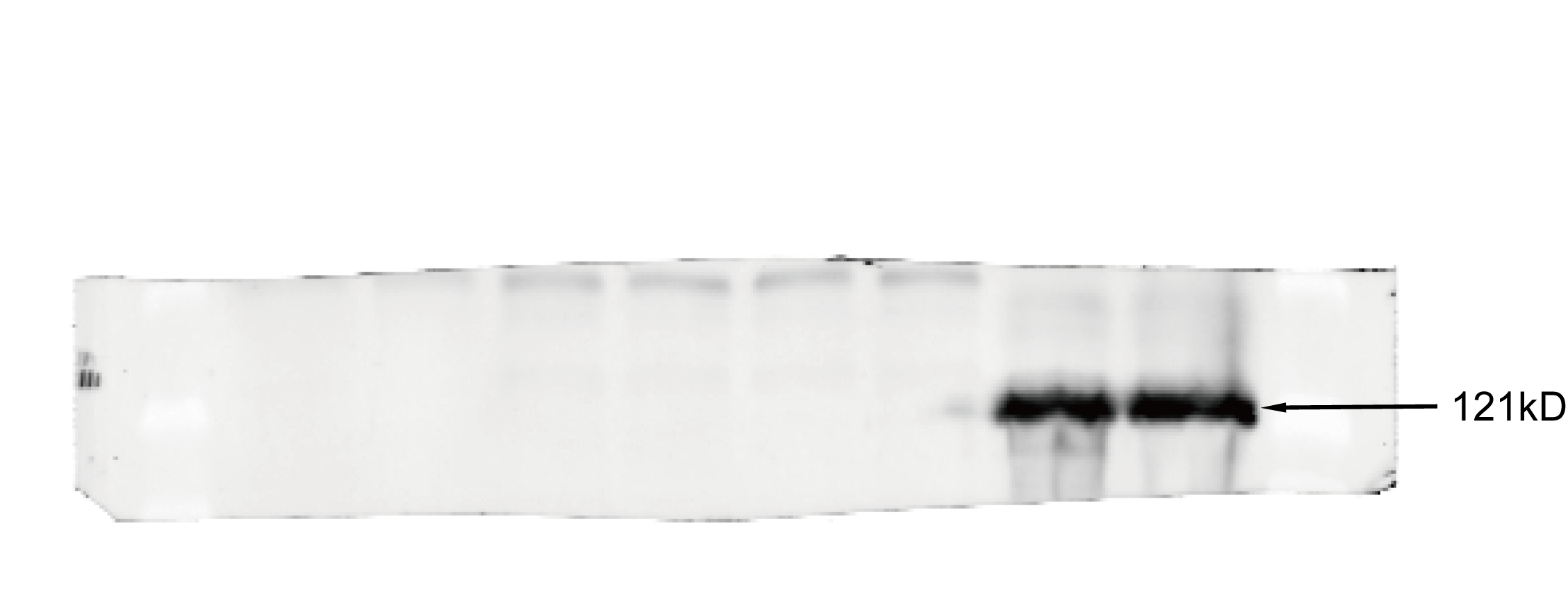

Supplement: Figure 5—figure supplement 2—source data 1. [file elife-76754-fig5-figsupp2-data1.zip › Figure 5 - figure supplement 2 Source data/Figure_5-figure supplement 2C-REST protein expression.tif]

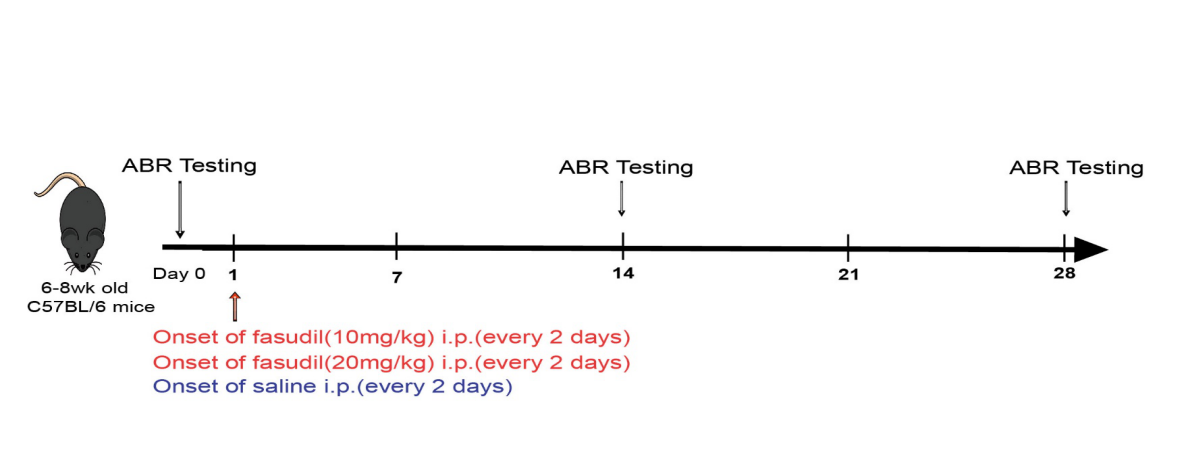

Supplement: Figure 7—source data 1. [file elife-76754-fig7-data1.zip › Figure_7-source_data/Fig.7 A.tif]

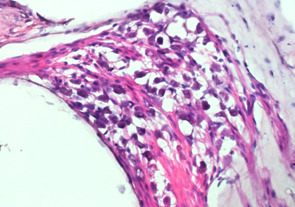

Supplement: Figure 7—figure supplement 2—source data 1. [file elife-76754-fig7-figsupp2-data1.zip › Figure 7 - figure supplement 2 Source data/Figure 7 - figure supplement 2A/Control-apex.tif]

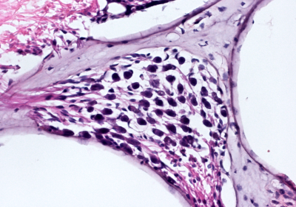

Supplement: Figure 7—figure supplement 2—source data 1. [file elife-76754-fig7-figsupp2-data1.zip › Figure 7 - figure supplement 2 Source data/Figure 7 - figure supplement 2A/Control-base.tif]

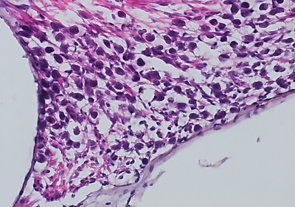

Supplement: Figure 7—figure supplement 2—source data 1. [file elife-76754-fig7-figsupp2-data1.zip › Figure 7 - figure supplement 2 Source data/Figure 7 - figure supplement 2A/Control-middle.tif]

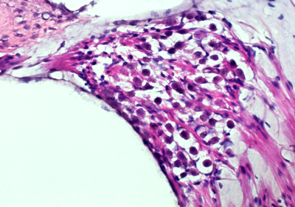

Supplement: Figure 7—figure supplement 2—source data 1. [file elife-76754-fig7-figsupp2-data1.zip › Figure 7 - figure supplement 2 Source data/Figure 7 - figure supplement 2A/Fasudil 10mg kg -apex.tif]

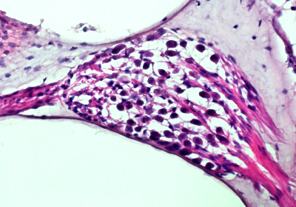

Supplement: Figure 7—figure supplement 2—source data 1. [file elife-76754-fig7-figsupp2-data1.zip › Figure 7 - figure supplement 2 Source data/Figure 7 - figure supplement 2A/Fasudil 10mg kg -base.tif]

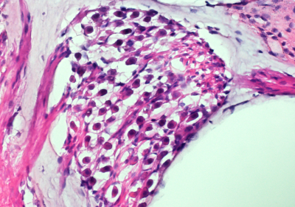

Supplement: Figure 7—figure supplement 2—source data 1. [file elife-76754-fig7-figsupp2-data1.zip › Figure 7 - figure supplement 2 Source data/Figure 7 - figure supplement 2A/Fasudil 10mg kg -middle.tif]

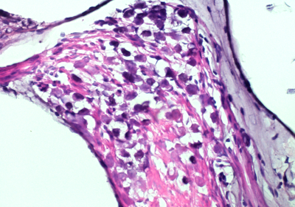

Supplement: Figure 7—figure supplement 2—source data 1. [file elife-76754-fig7-figsupp2-data1.zip › Figure 7 - figure supplement 2 Source data/Figure 7 - figure supplement 2A/Fasudil 20mg kg -apex.tif]

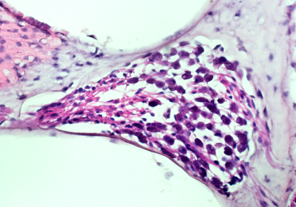

Supplement: Figure 7—figure supplement 2—source data 1. [file elife-76754-fig7-figsupp2-data1.zip › Figure 7 - figure supplement 2 Source data/Figure 7 - figure supplement 2A/Fasudil 20mg kg -base.tif]

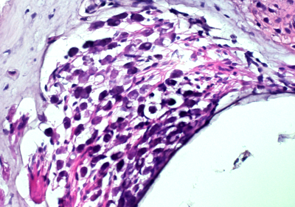

Supplement: Figure 7—figure supplement 2—source data 1. [file elife-76754-fig7-figsupp2-data1.zip › Figure 7 - figure supplement 2 Source data/Figure 7 - figure supplement 2A/Fasudil 20mg kg -middle.tif]

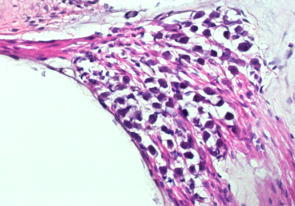

Supplement: Figure 7—figure supplement 2—source data 1. [file elife-76754-fig7-figsupp2-data1.zip › Figure 7 - figure supplement 2 Source data/Figure 7 - figure supplement 2A/Saline-apex.tif]

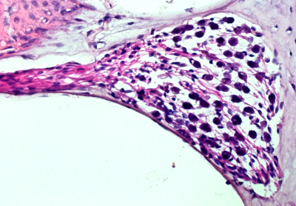

Supplement: Figure 7—figure supplement 2—source data 1. [file elife-76754-fig7-figsupp2-data1.zip › Figure 7 - figure supplement 2 Source data/Figure 7 - figure supplement 2A/Saline-base.tif]

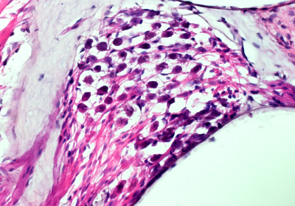

Supplement: Figure 7—figure supplement 2—source data 1. [file elife-76754-fig7-figsupp2-data1.zip › Figure 7 - figure supplement 2 Source data/Figure 7 - figure supplement 2A/Saline-middle.tif]

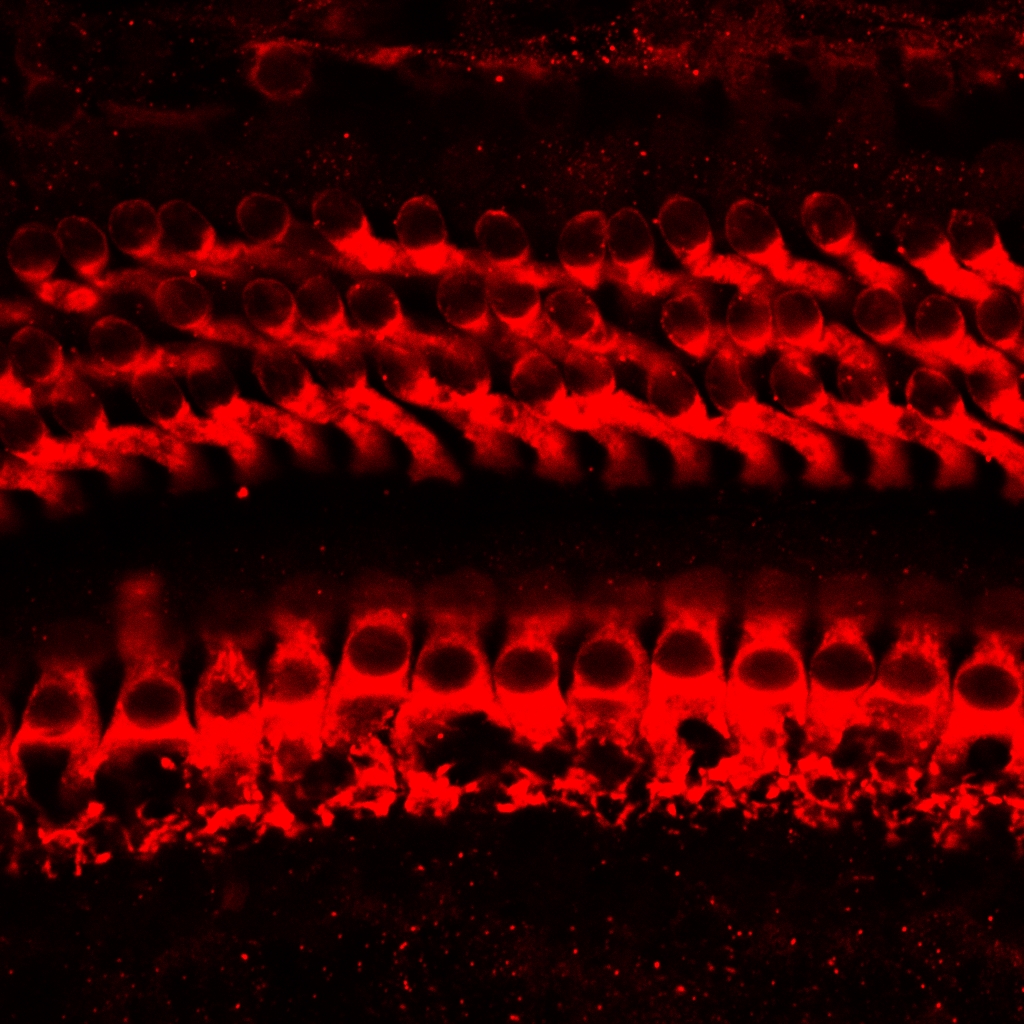

Supplement: Figure 7—figure supplement 2—source data 1. [file elife-76754-fig7-figsupp2-data1.zip › Figure 7 - figure supplement 2 Source data/Figure 7 - figure supplement 2C/Fasudil 10mg kg -apex.tif]

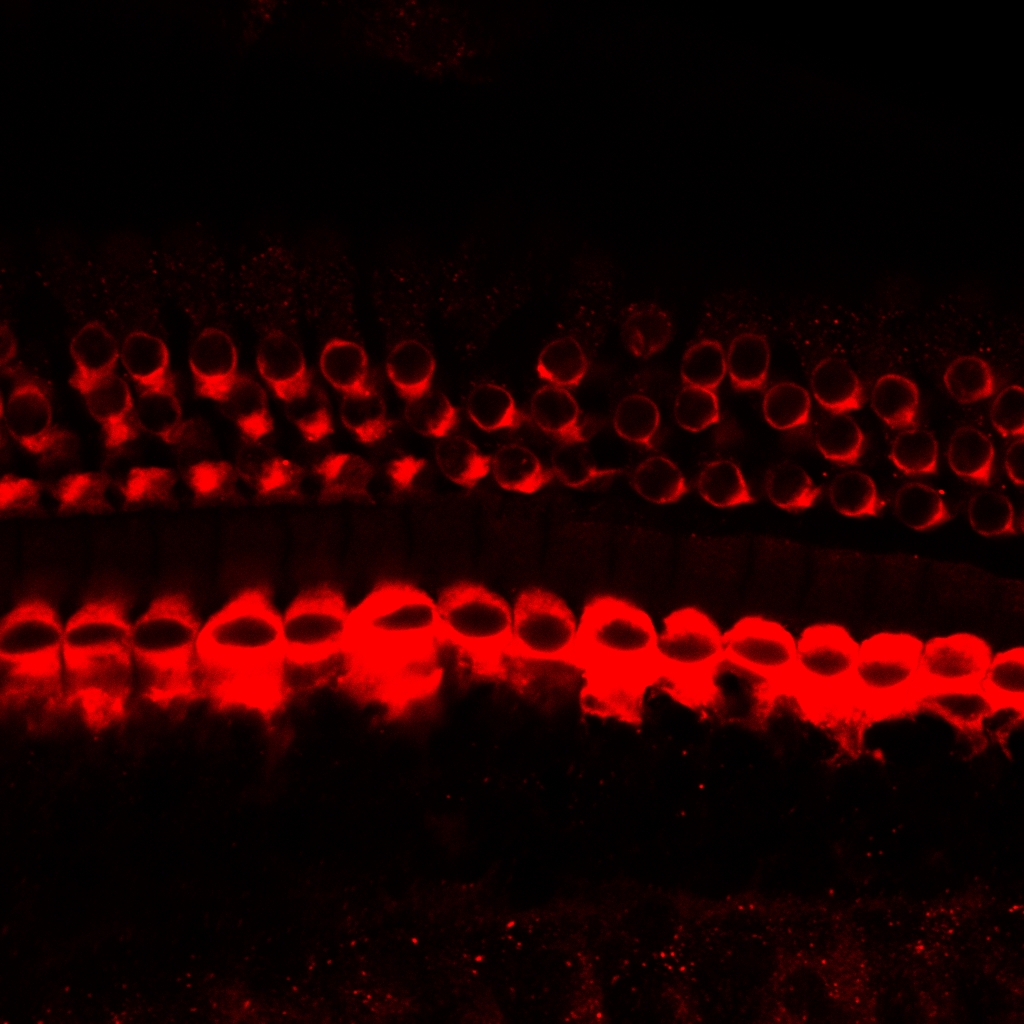

Supplement: Figure 7—figure supplement 2—source data 1. [file elife-76754-fig7-figsupp2-data1.zip › Figure 7 - figure supplement 2 Source data/Figure 7 - figure supplement 2C/Fasudil 10mg kg -base.tif]

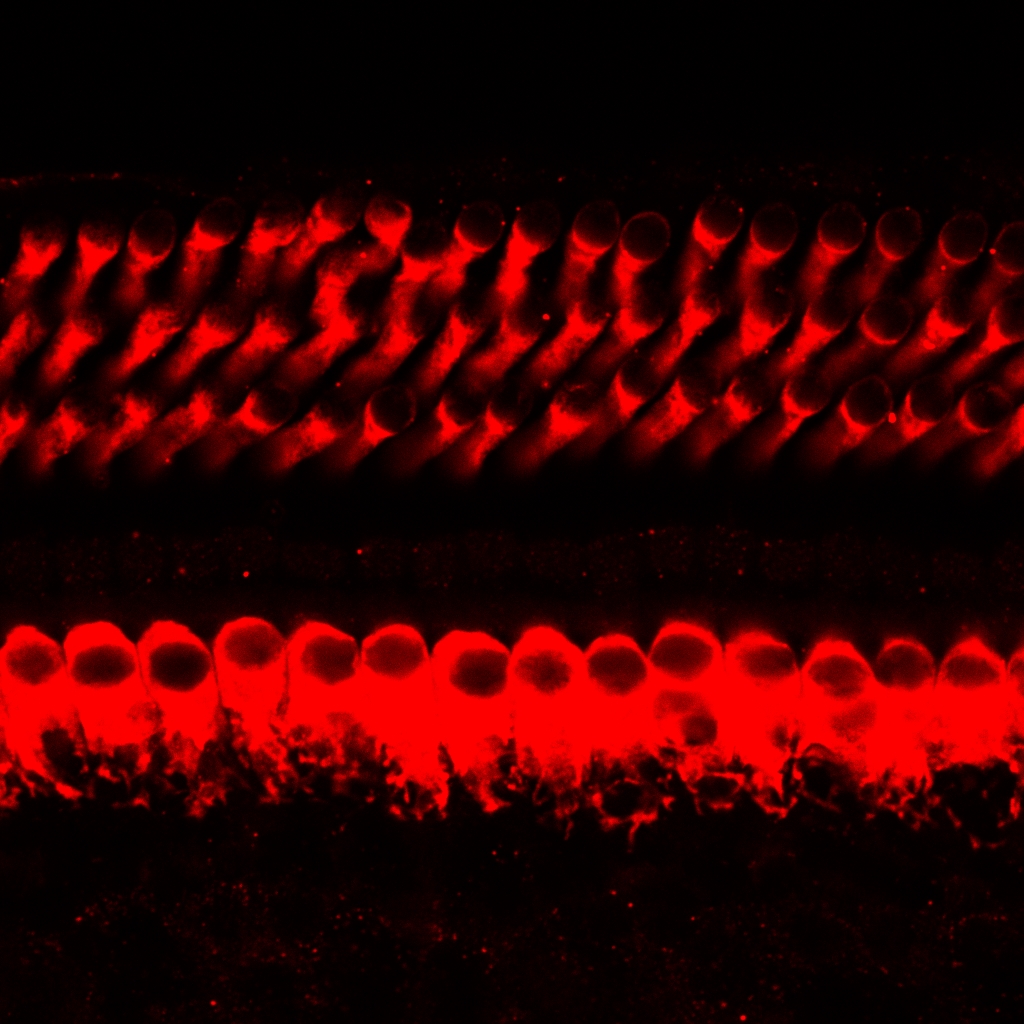

Supplement: Figure 7—figure supplement 2—source data 1. [file elife-76754-fig7-figsupp2-data1.zip › Figure 7 - figure supplement 2 Source data/Figure 7 - figure supplement 2C/Fasudil 10mg kg -middle.tif]

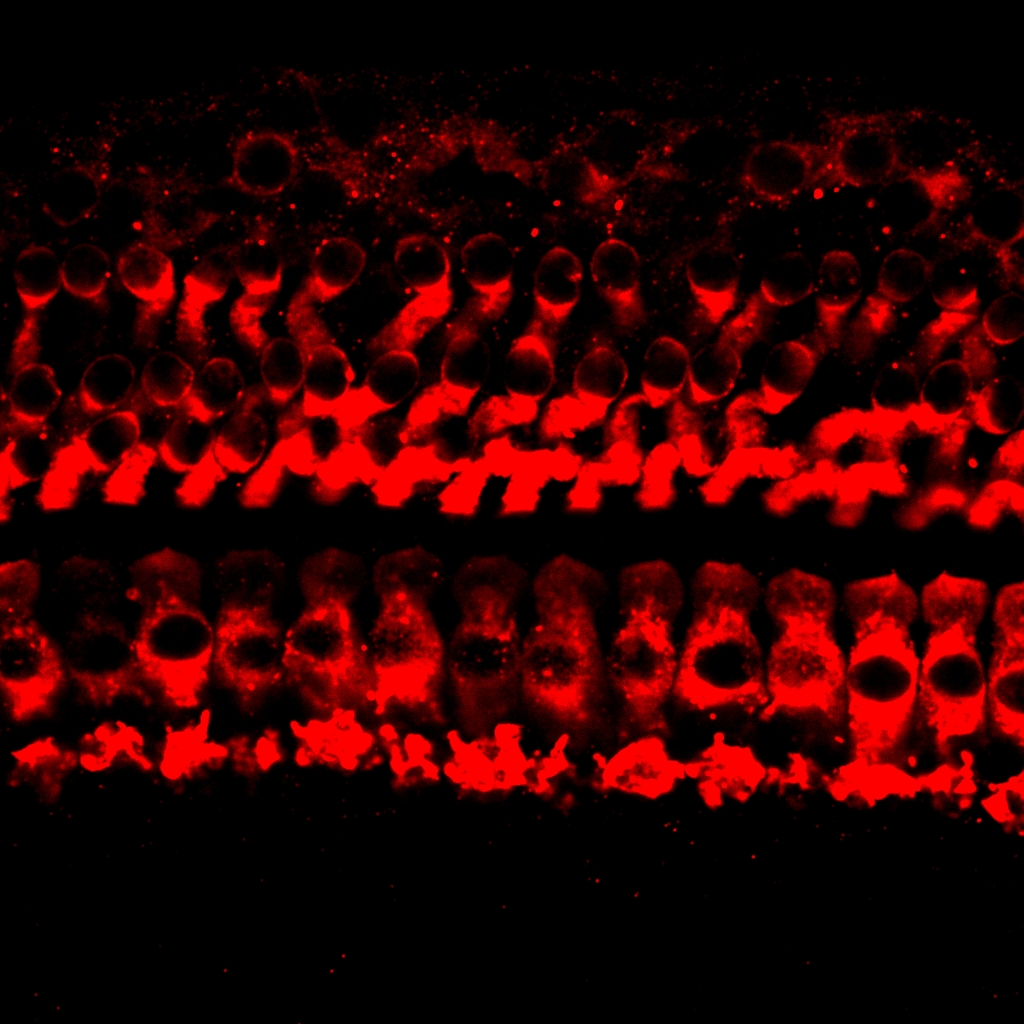

Supplement: Figure 7—figure supplement 2—source data 1. [file elife-76754-fig7-figsupp2-data1.zip › Figure 7 - figure supplement 2 Source data/Figure 7 - figure supplement 2C/Fasudil 20mg kg -apex.tif]

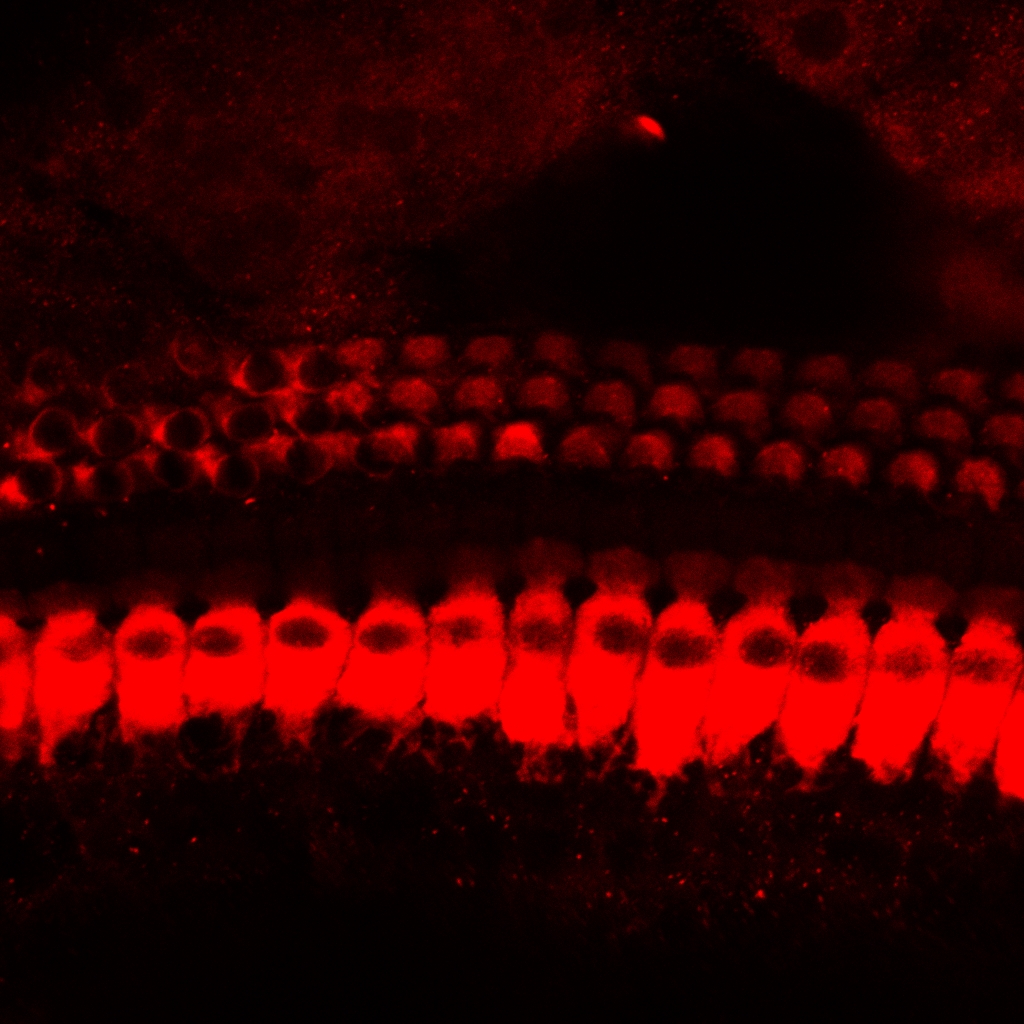

Supplement: Figure 7—figure supplement 2—source data 1. [file elife-76754-fig7-figsupp2-data1.zip › Figure 7 - figure supplement 2 Source data/Figure 7 - figure supplement 2C/Fasudil 20mg kg -base.tif]

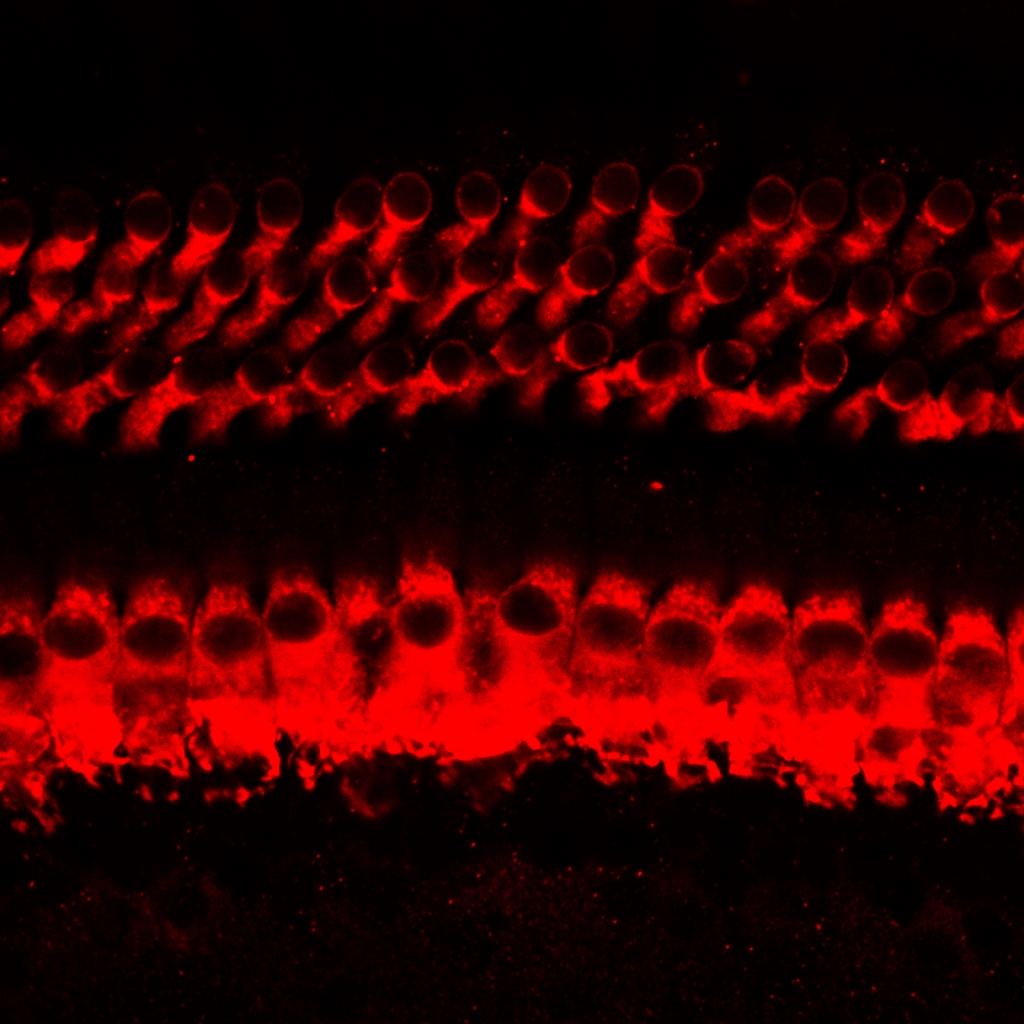

Supplement: Figure 7—figure supplement 2—source data 1. [file elife-76754-fig7-figsupp2-data1.zip › Figure 7 - figure supplement 2 Source data/Figure 7 - figure supplement 2C/Fasudil 20mg kg -middle.tif]

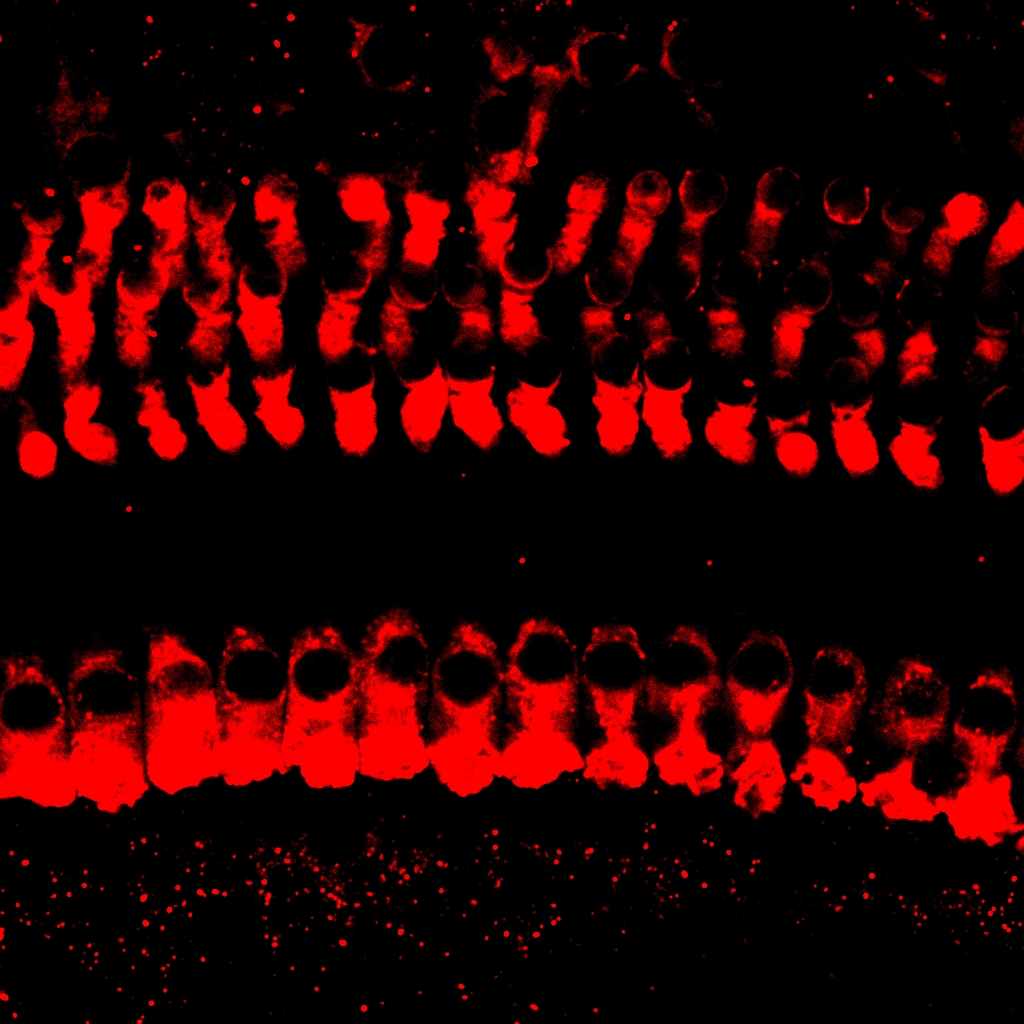

Supplement: Figure 7—figure supplement 2—source data 1. [file elife-76754-fig7-figsupp2-data1.zip › Figure 7 - figure supplement 2 Source data/Figure 7 - figure supplement 2C/Saline -apex.tif]

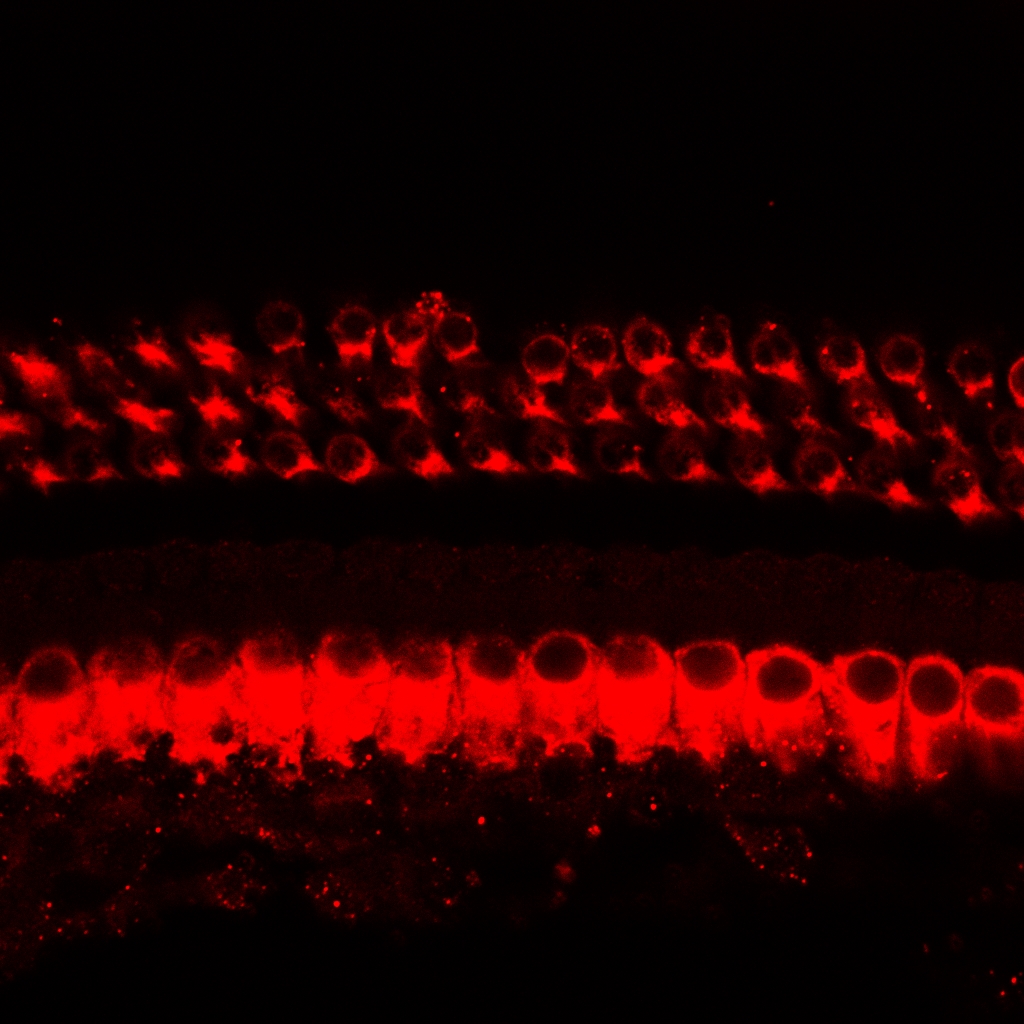

Supplement: Figure 7—figure supplement 2—source data 1. [file elife-76754-fig7-figsupp2-data1.zip › Figure 7 - figure supplement 2 Source data/Figure 7 - figure supplement 2C/Saline -base.tif]

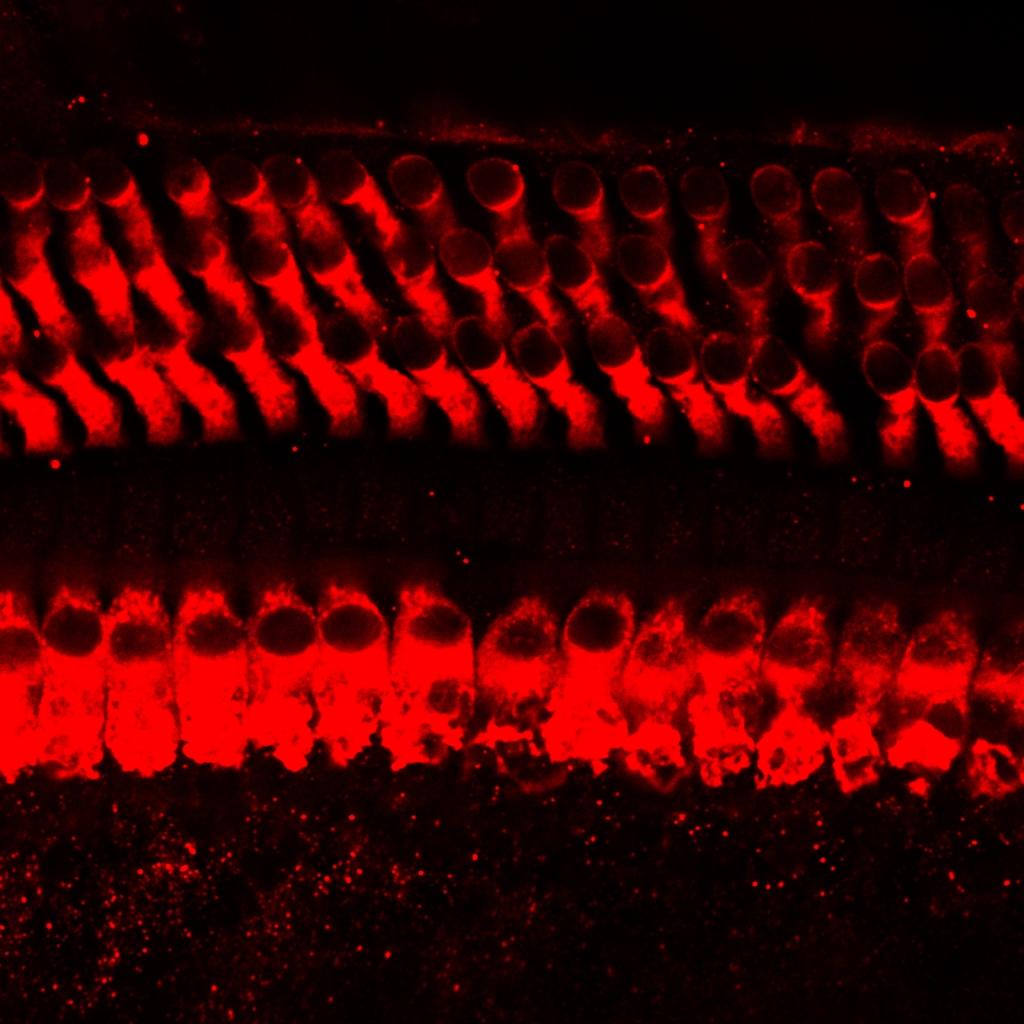

Supplement: Figure 7—figure supplement 2—source data 1. [file elife-76754-fig7-figsupp2-data1.zip › Figure 7 - figure supplement 2 Source data/Figure 7 - figure supplement 2C/Saline -middle.tif]

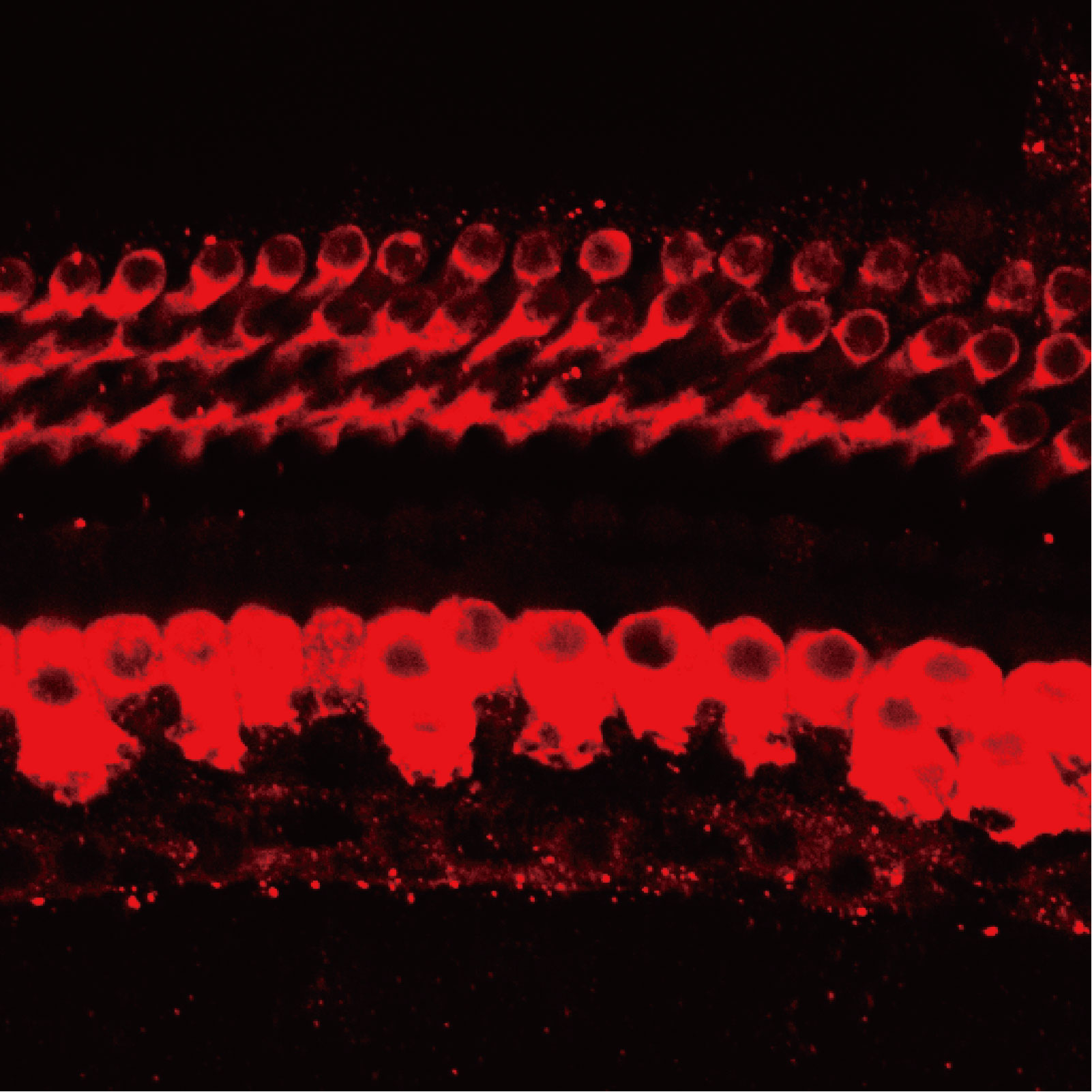

Supplement: Figure 7—figure supplement 2—source data 1. [file elife-76754-fig7-figsupp2-data1.zip › Figure 7 - figure supplement 2 Source data/Figure 7 - figure supplement 2C/control apex.tif]

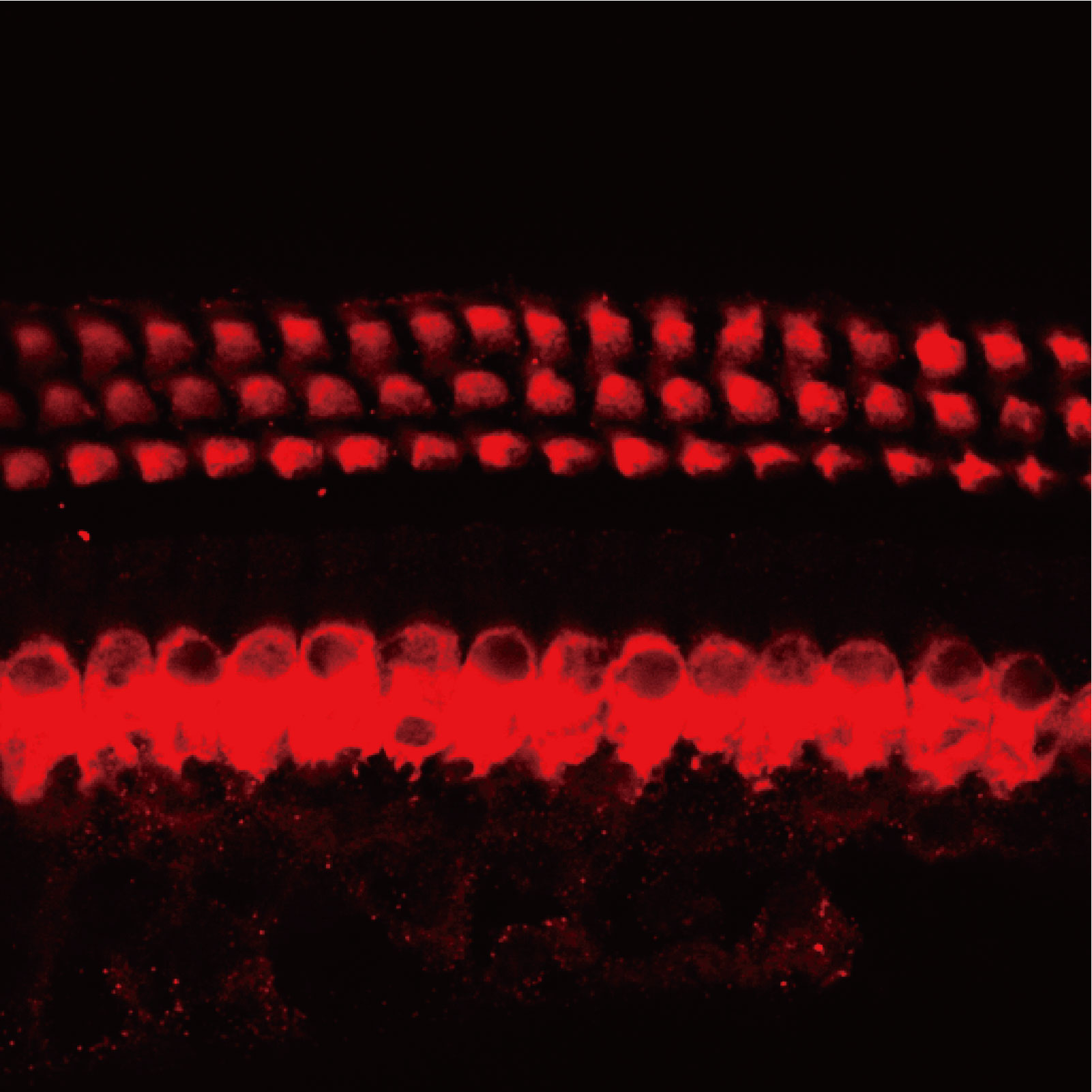

Supplement: Figure 7—figure supplement 2—source data 1. [file elife-76754-fig7-figsupp2-data1.zip › Figure 7 - figure supplement 2 Source data/Figure 7 - figure supplement 2C/control base.tif]

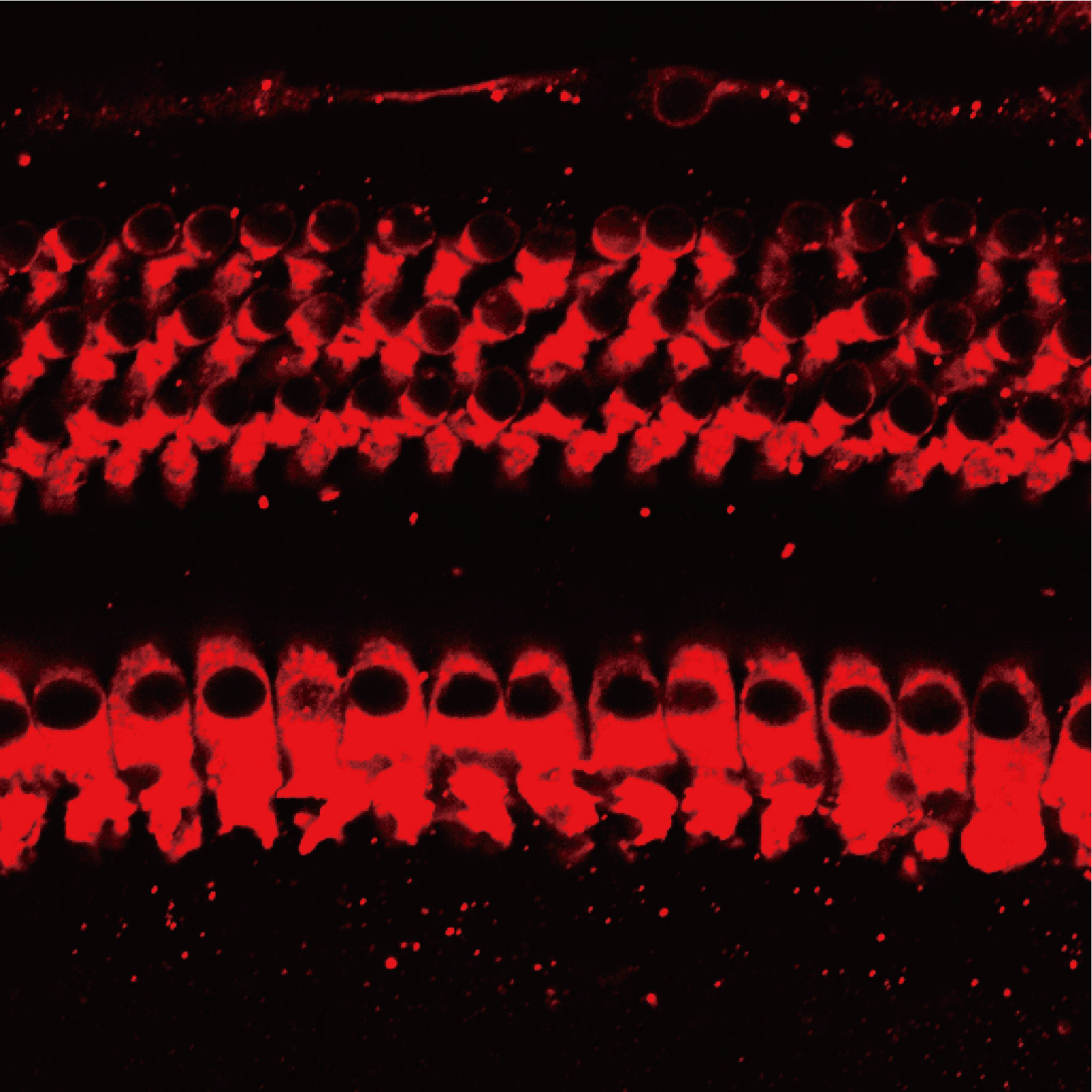

Supplement: Figure 7—figure supplement 2—source data 1. [file elife-76754-fig7-figsupp2-data1.zip › Figure 7 - figure supplement 2 Source data/Figure 7 - figure supplement 2C/control middle.tif]

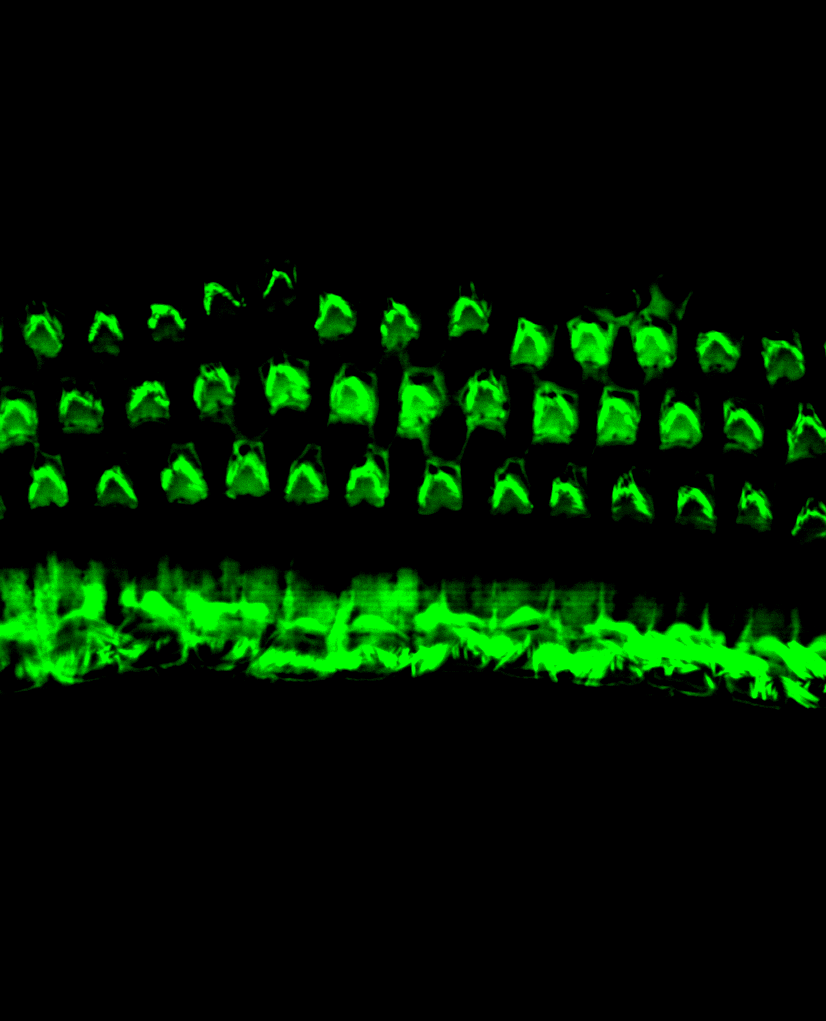

Supplement: Figure 7—figure supplement 2—source data 1. [file elife-76754-fig7-figsupp2-data1.zip › Figure 7 - figure supplement 2 Source data/Figure 7 - figure supplement 2D/control apex.tif]

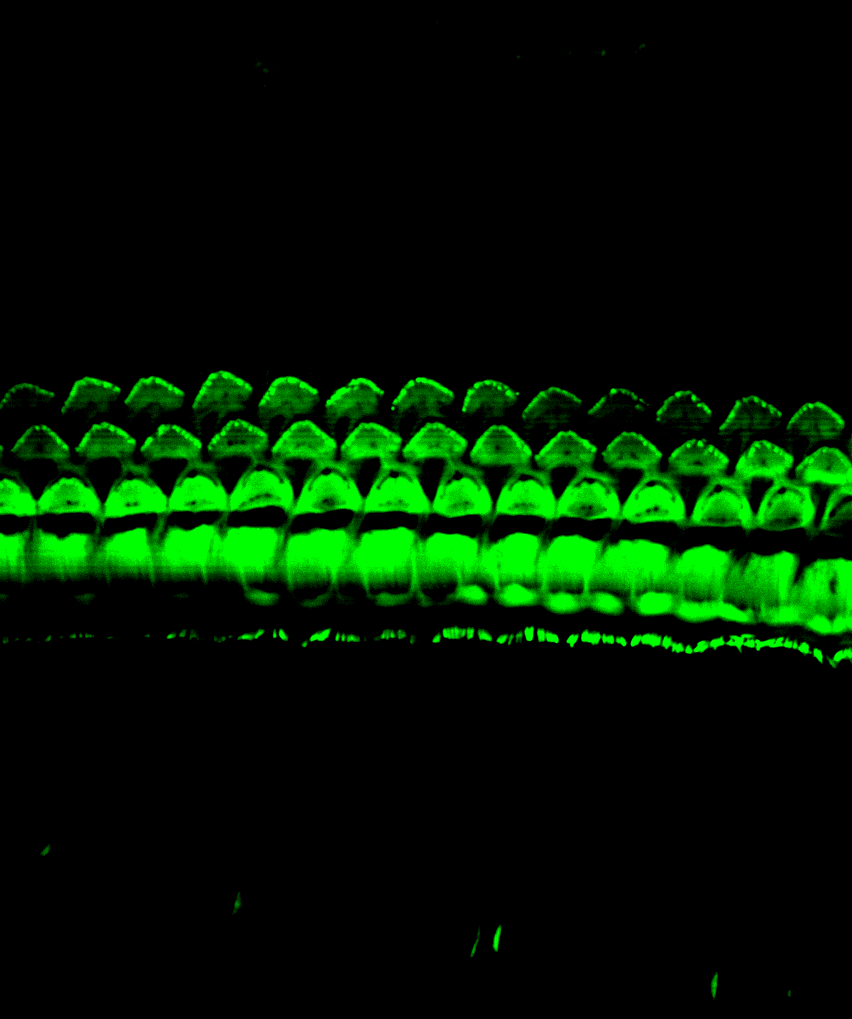

Supplement: Figure 7—figure supplement 2—source data 1. [file elife-76754-fig7-figsupp2-data1.zip › Figure 7 - figure supplement 2 Source data/Figure 7 - figure supplement 2D/control base.tif]

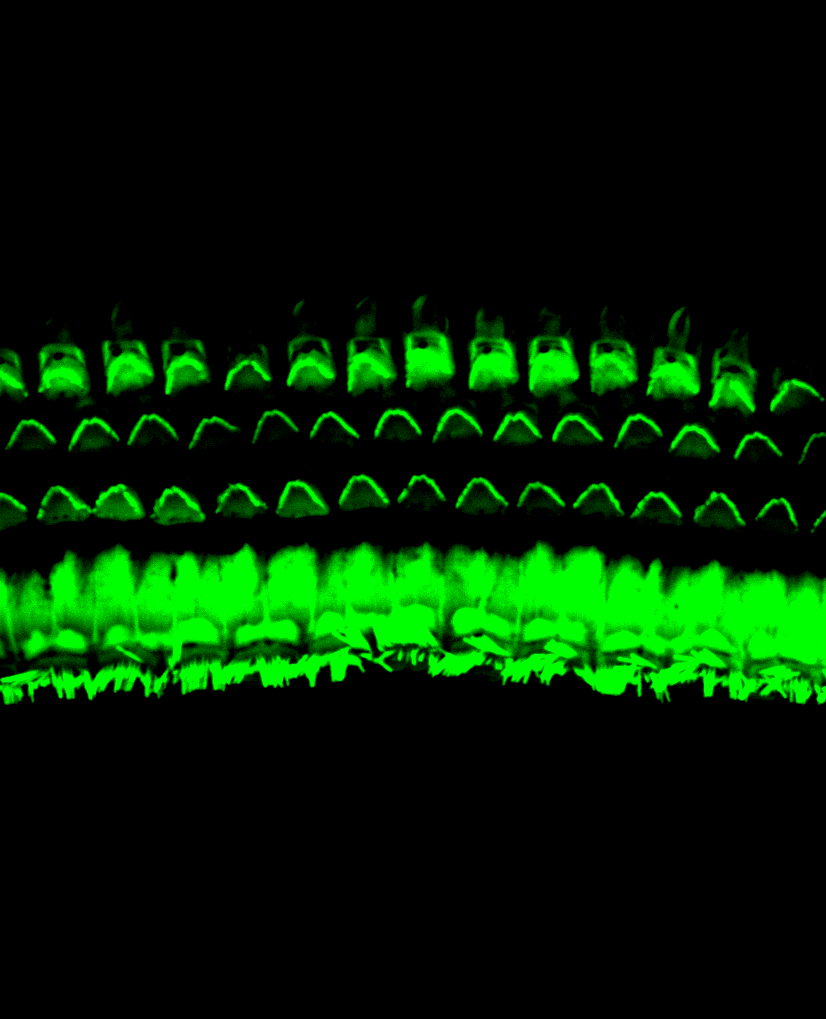

Supplement: Figure 7—figure supplement 2—source data 1. [file elife-76754-fig7-figsupp2-data1.zip › Figure 7 - figure supplement 2 Source data/Figure 7 - figure supplement 2D/control middle.tif]

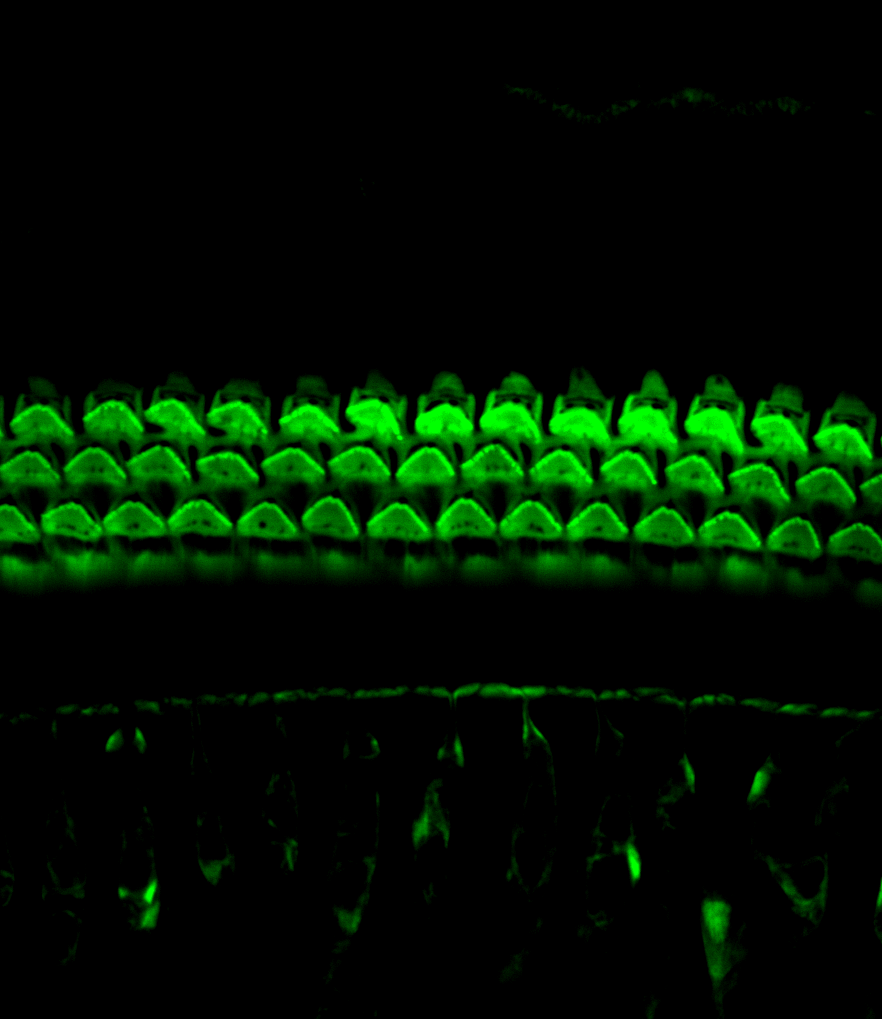

Supplement: Figure 7—figure supplement 2—source data 1. [file elife-76754-fig7-figsupp2-data1.zip › Figure 7 - figure supplement 2 Source data/Figure 7 - figure supplement 2D/fasudil 10mg kg base.tif]

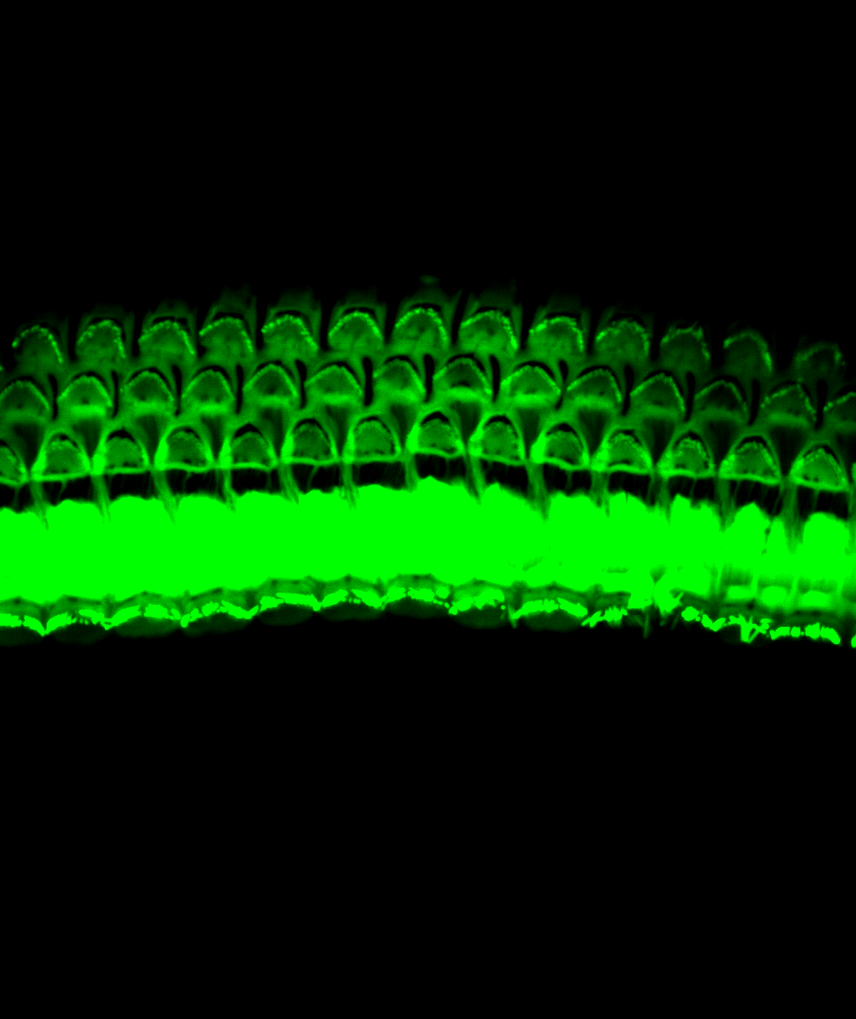

Supplement: Figure 7—figure supplement 2—source data 1. [file elife-76754-fig7-figsupp2-data1.zip › Figure 7 - figure supplement 2 Source data/Figure 7 - figure supplement 2D/fasudil 10mg kg middle.tif]

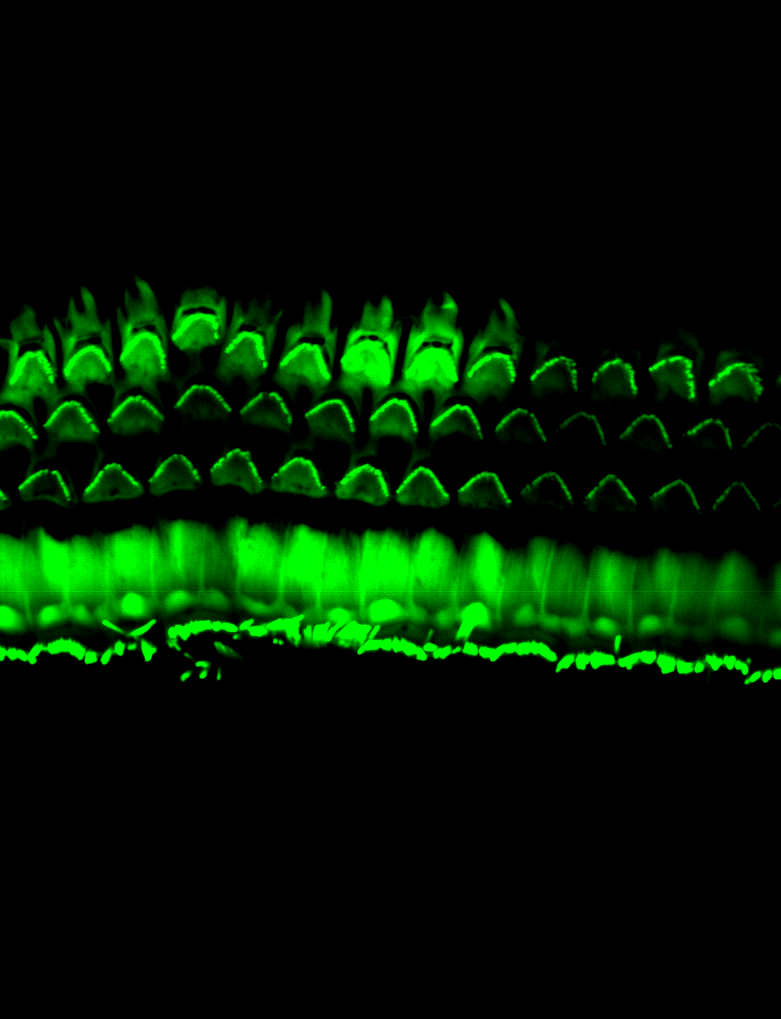

Supplement: Figure 7—figure supplement 2—source data 1. [file elife-76754-fig7-figsupp2-data1.zip › Figure 7 - figure supplement 2 Source data/Figure 7 - figure supplement 2D/fasudil 10mg kg apex.tif]

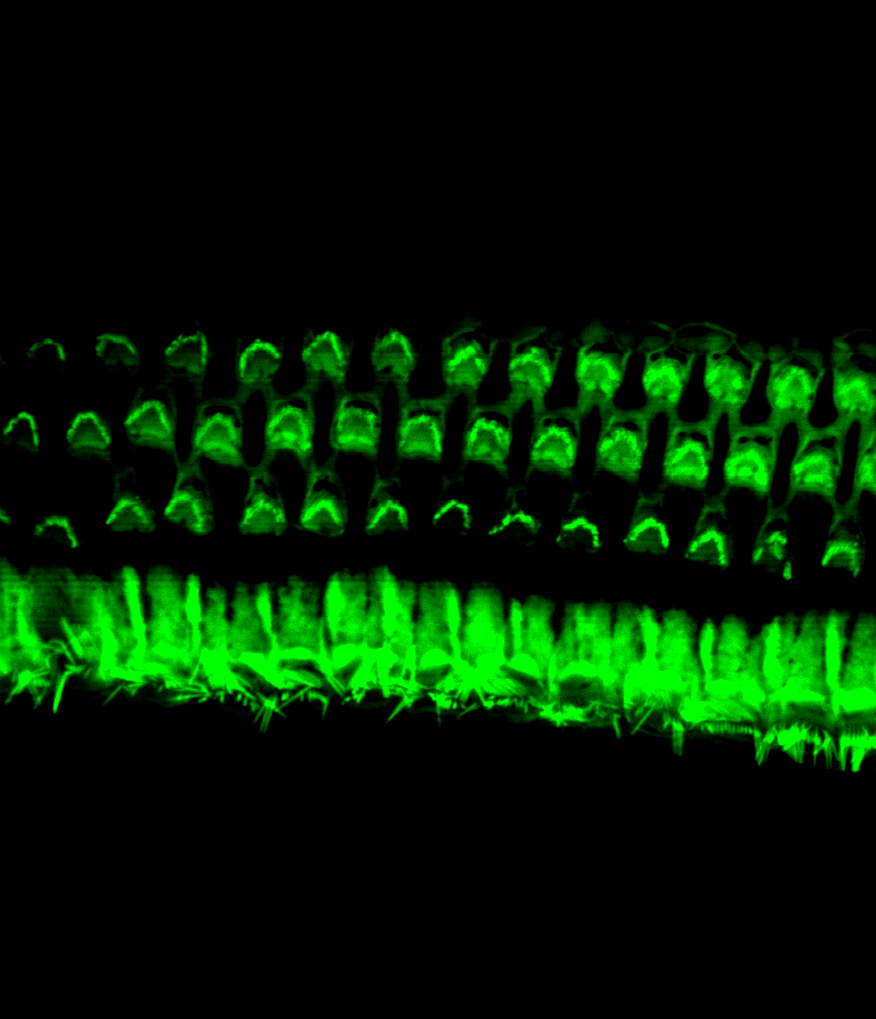

Supplement: Figure 7—figure supplement 2—source data 1. [file elife-76754-fig7-figsupp2-data1.zip › Figure 7 - figure supplement 2 Source data/Figure 7 - figure supplement 2D/fasudil 20mg kg apex.tif]

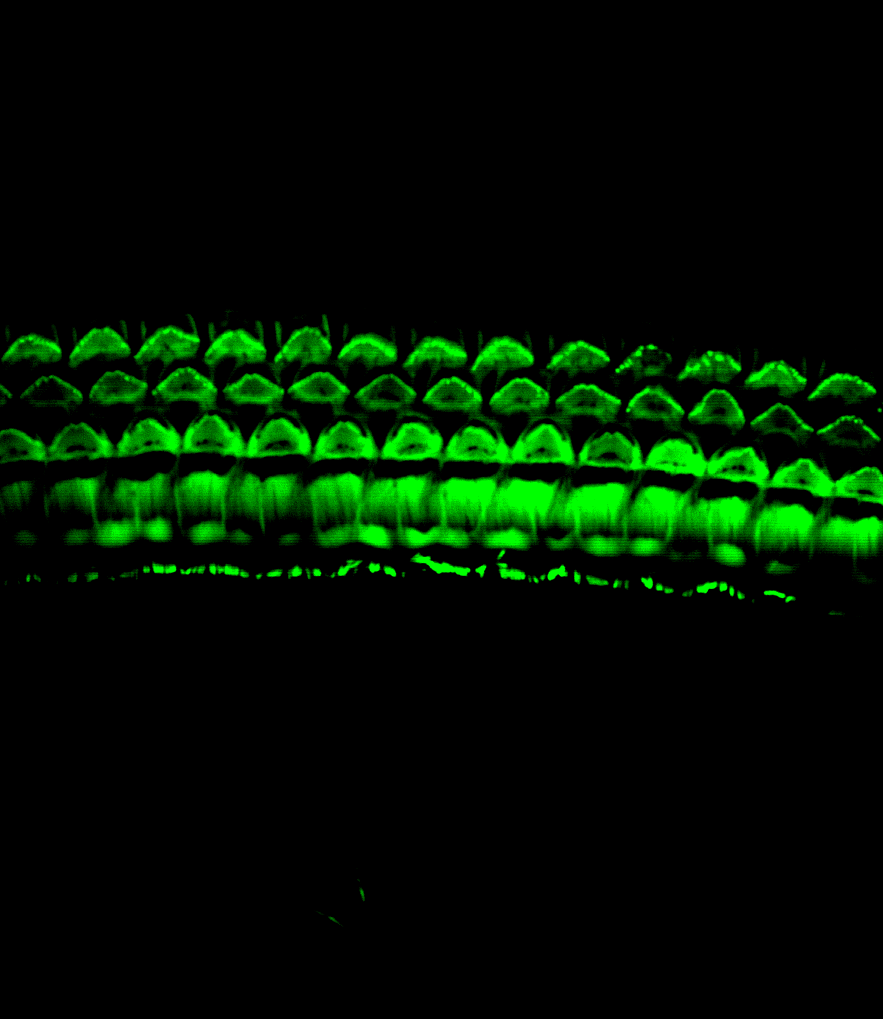

Supplement: Figure 7—figure supplement 2—source data 1. [file elife-76754-fig7-figsupp2-data1.zip › Figure 7 - figure supplement 2 Source data/Figure 7 - figure supplement 2D/fasudil 20mg kg base.tif]

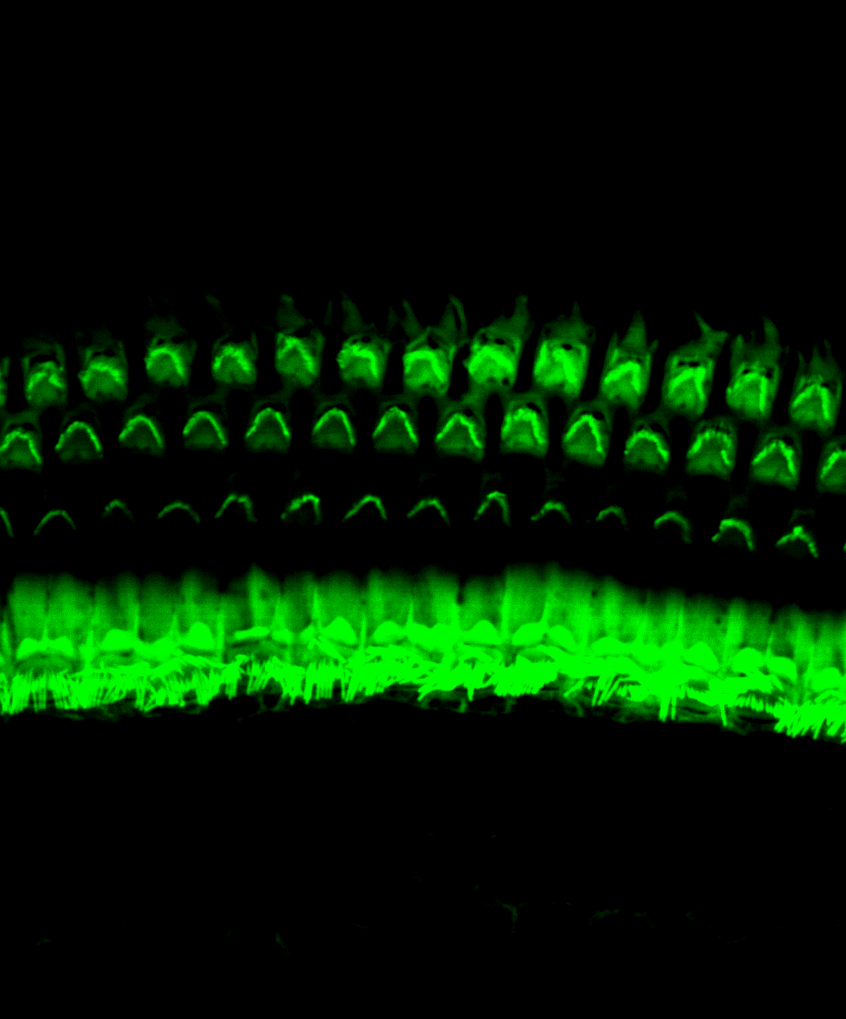

Supplement: Figure 7—figure supplement 2—source data 1. [file elife-76754-fig7-figsupp2-data1.zip › Figure 7 - figure supplement 2 Source data/Figure 7 - figure supplement 2D/fasudil 20mg kg middle.tif]

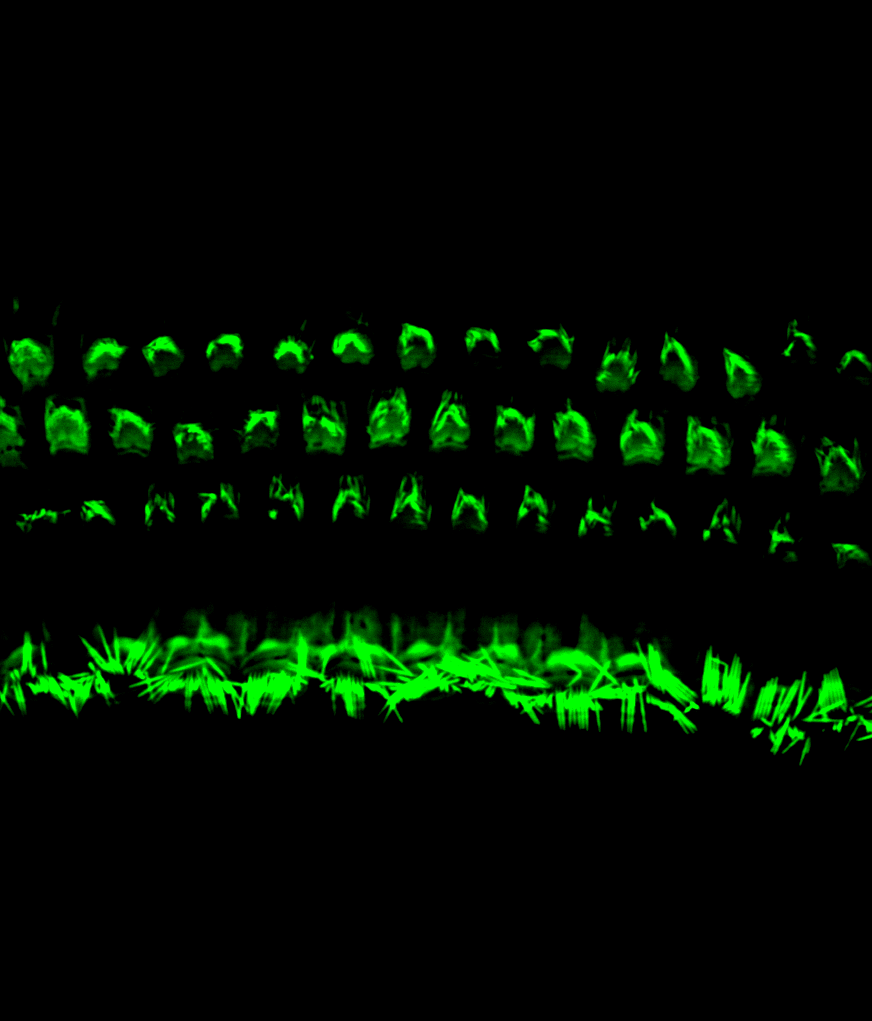

Supplement: Figure 7—figure supplement 2—source data 1. [file elife-76754-fig7-figsupp2-data1.zip › Figure 7 - figure supplement 2 Source data/Figure 7 - figure supplement 2D/saline apex.tif]

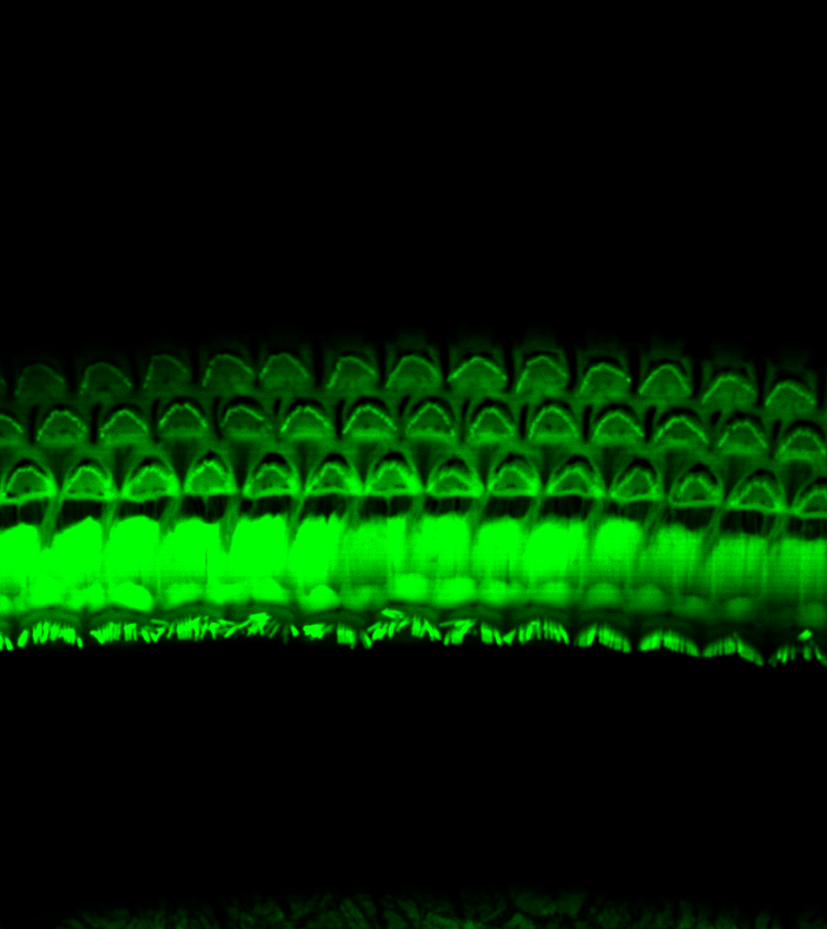

Supplement: Figure 7—figure supplement 2—source data 1. [file elife-76754-fig7-figsupp2-data1.zip › Figure 7 - figure supplement 2 Source data/Figure 7 - figure supplement 2D/saline base.tif]

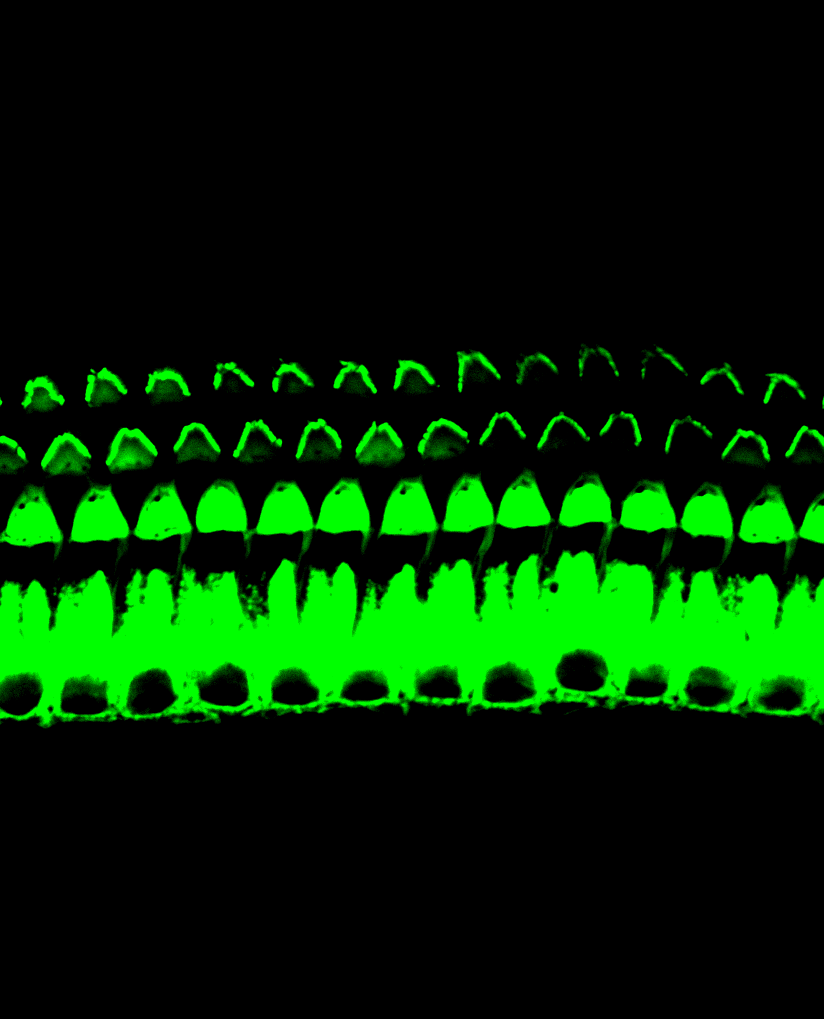

Supplement: Figure 7—figure supplement 2—source data 1. [file elife-76754-fig7-figsupp2-data1.zip › Figure 7 - figure supplement 2 Source data/Figure 7 - figure supplement 2D/saline middle .tif]

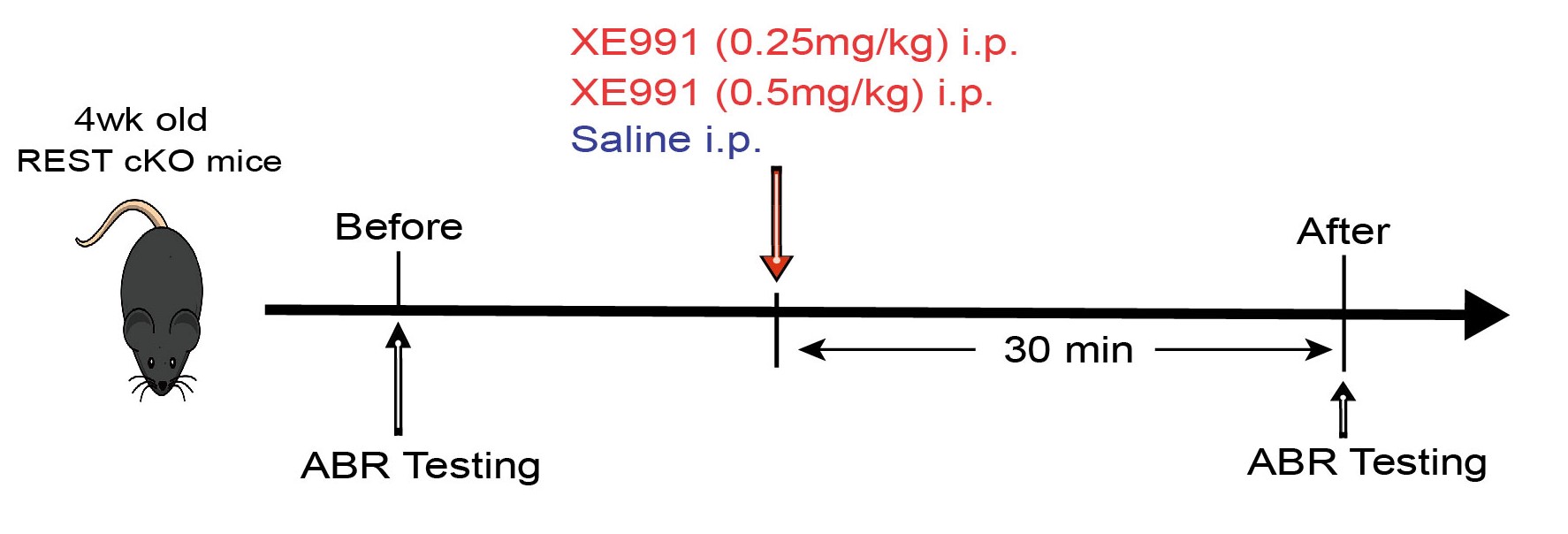

Supplement: Figure 8—source data 1. [file elife-76754-fig8-data1.zip › Figure_8-source_data/Fig.8 A/Fig.8 A.tif]

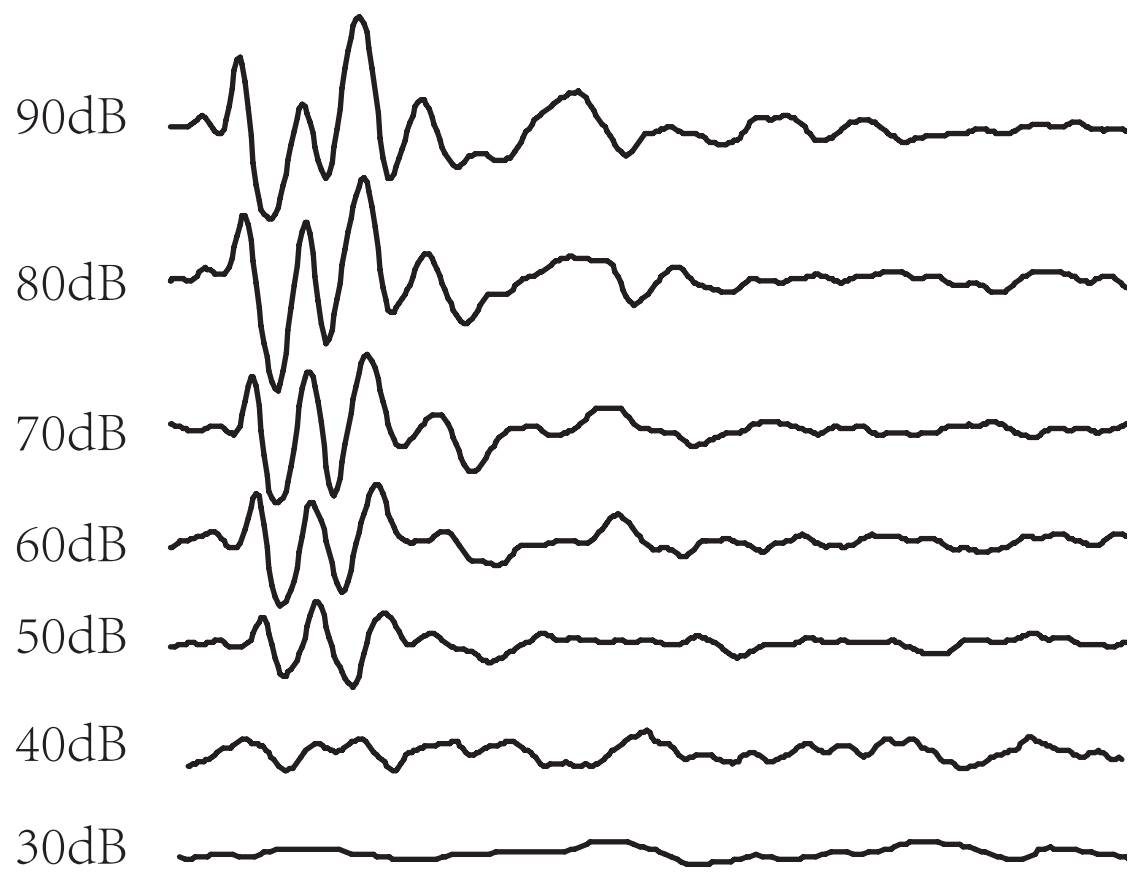

Supplement: Figure 8—source data 1. [file elife-76754-fig8-data1.zip › Figure_8-source_data/Fig.8 B/Fig.8 B-XE991 0.25mg kg after.pdf]

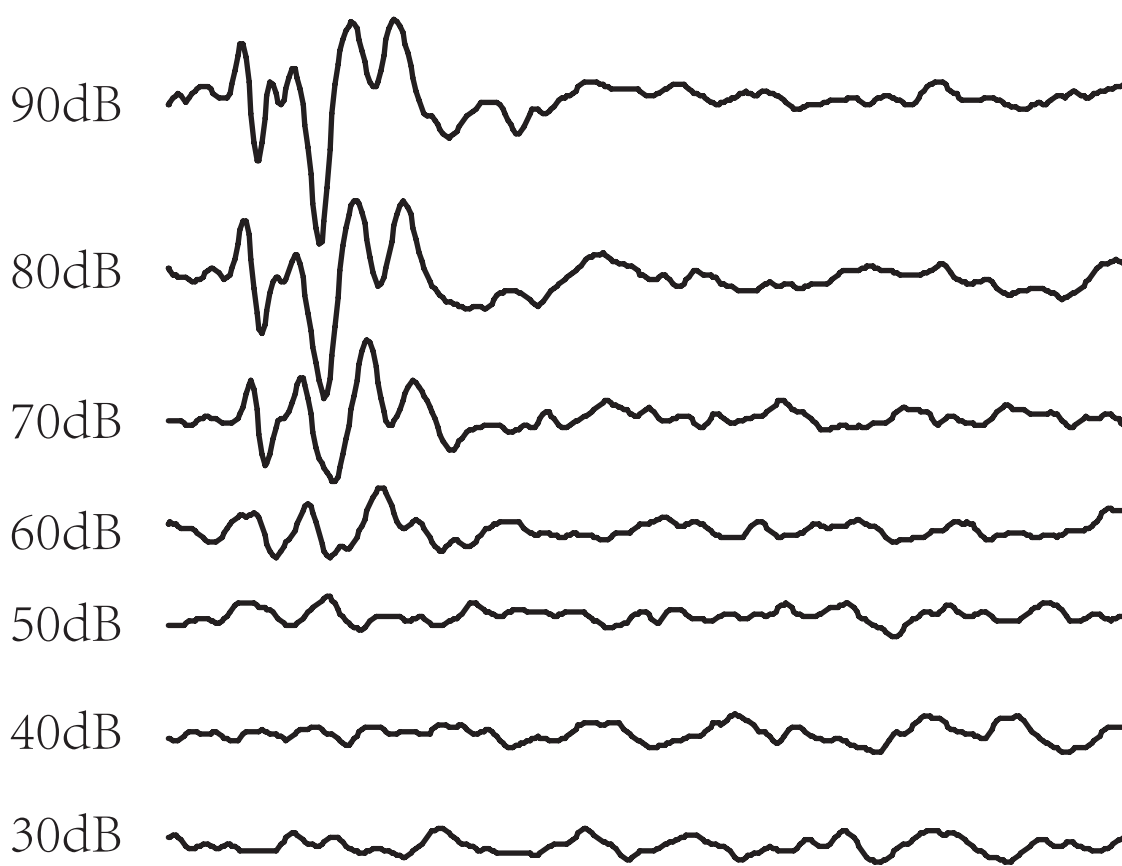

Supplement: Figure 8—source data 1. [file elife-76754-fig8-data1.zip › Figure_8-source_data/Fig.8 B/Fig.8 B-XE991 0.25mg kg before.pdf]

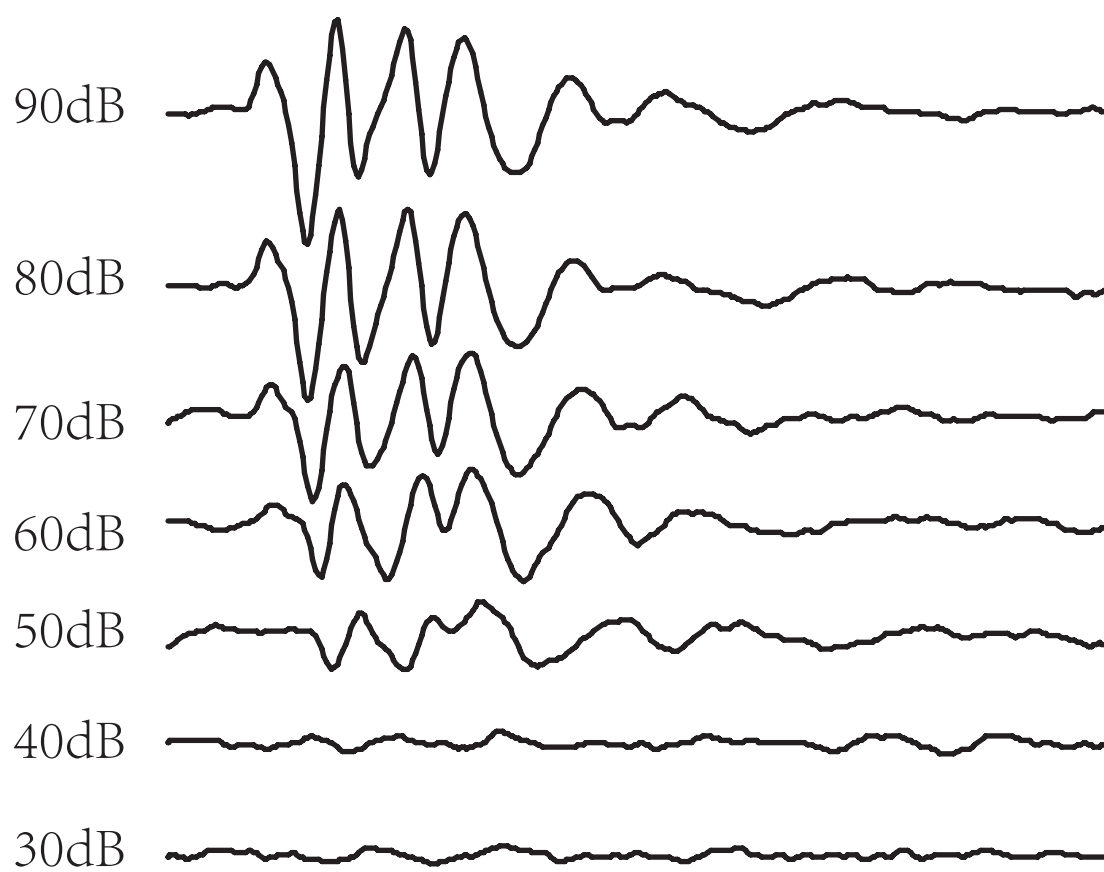

Supplement: Figure 8—source data 1. [file elife-76754-fig8-data1.zip › Figure_8-source_data/Fig.8 B/Fig.8 B-XE991 0.5mg kg after.pdf]

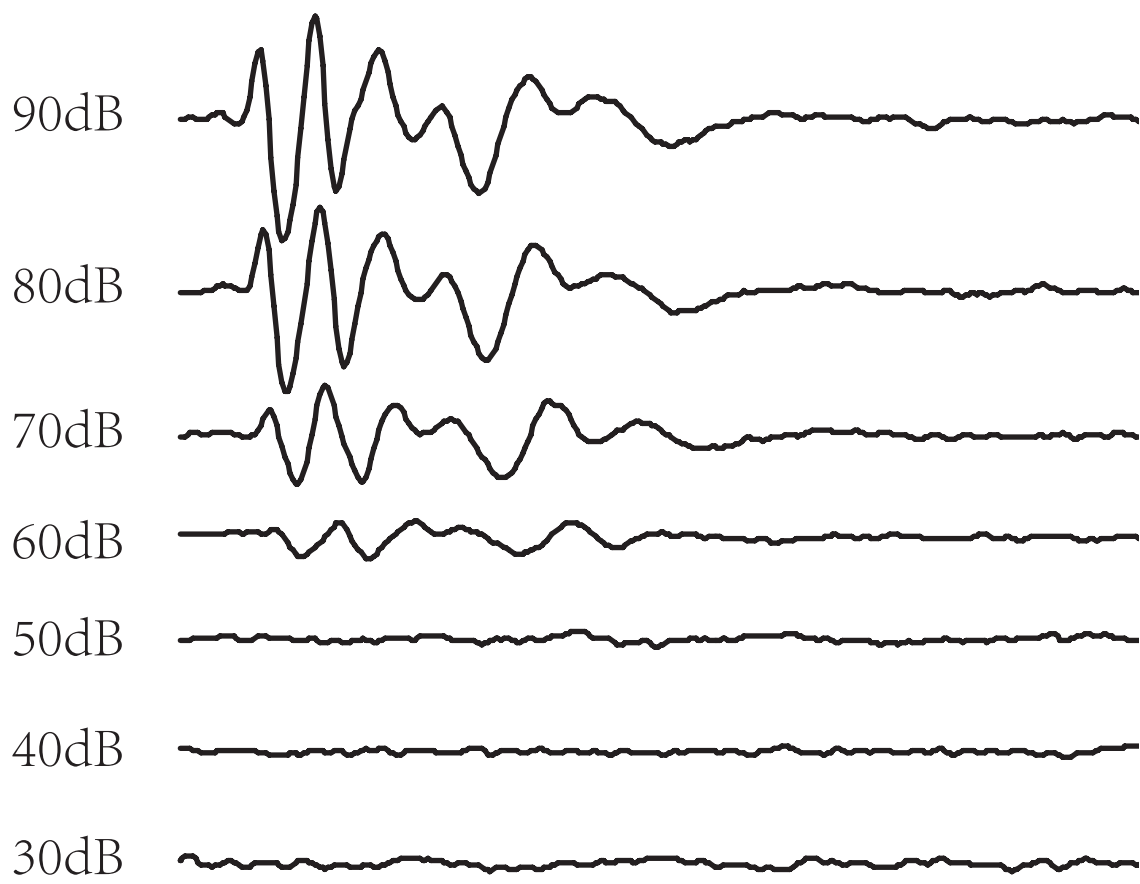

Supplement: Figure 8—source data 1. [file elife-76754-fig8-data1.zip › Figure_8-source_data/Fig.8 B/Fig.8 B-XE991 0.5mg kg before.pdf]

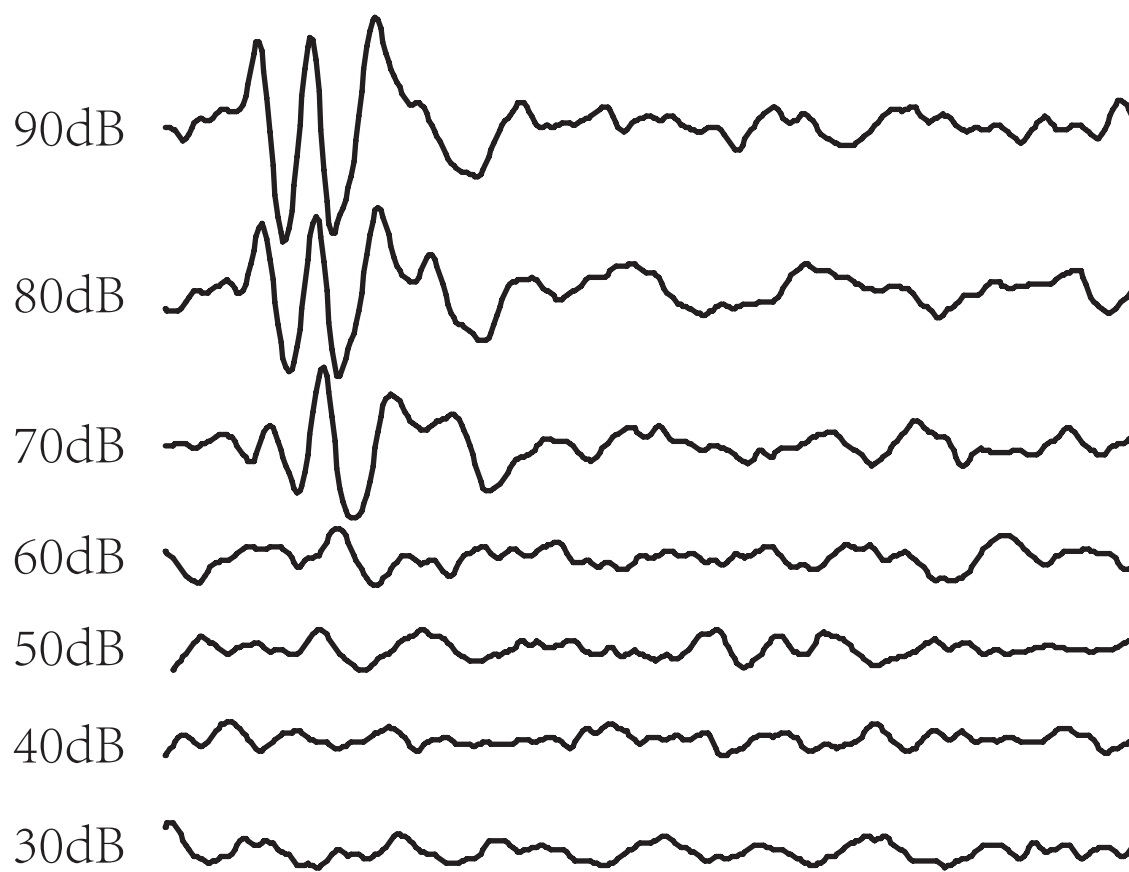

Supplement: Figure 8—source data 1. [file elife-76754-fig8-data1.zip › Figure_8-source_data/Fig.8 B/Fig.8 B-saline after.pdf]

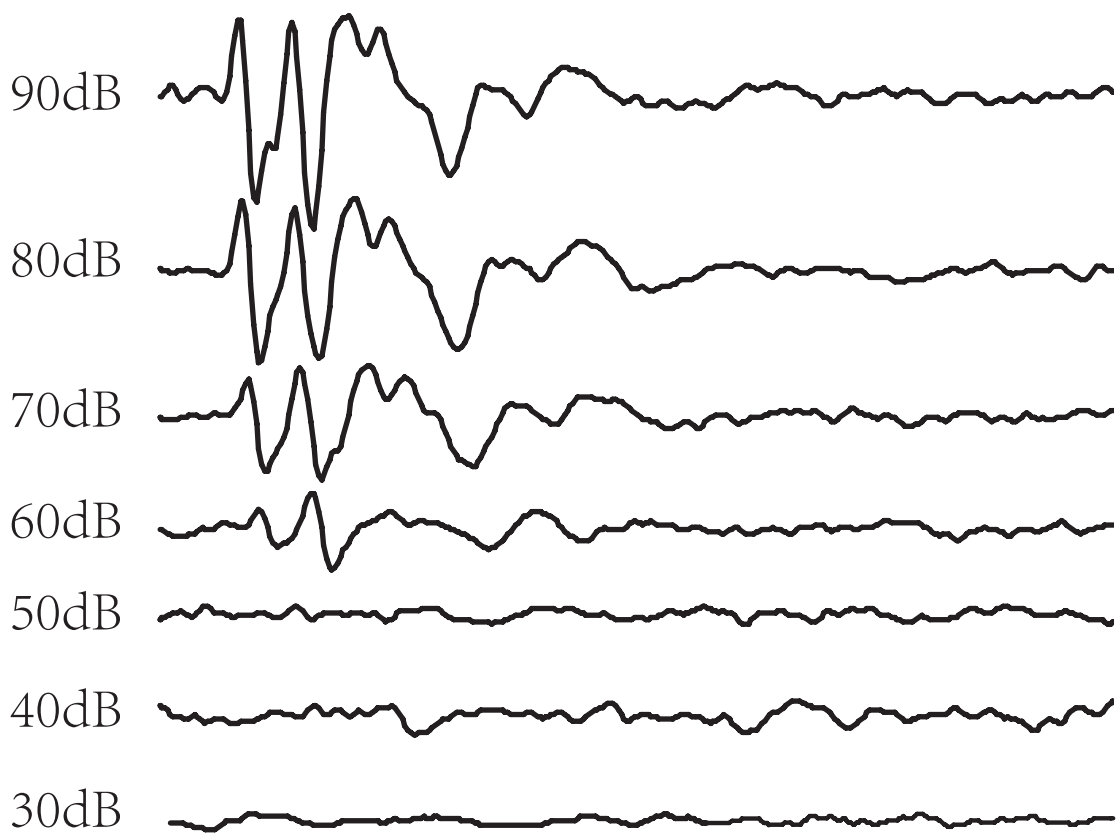

Supplement: Figure 8—source data 1. [file elife-76754-fig8-data1.zip › Figure_8-source_data/Fig.8 B/Fig.8 B-saline before.pdf]
