# Supplementary material for: Repressor element 1-silencing transcription factor deficiency yields profound hearing loss through Kv7.4 channel upsurge in auditory neurons and hair cells
Source: eLife. 2022 Sep 20;11:e76754. doi: 10.7554/eLife.76754 (PMC9525063; doi:10.7554/eLife.76754)
Supplement: Source data 1. [file elife-76754-data1.docx]

**Supplement**

**Repressor element 1-silencing transcription factor deficiency yields profound hearing loss through K_v_7.4 channel upsurge in auditory neurons and hair cells**

Haiwei Zhang^1,2,3^, Hongchen Li^1,2,3^, Mingshun Lu^1,2,3^, Shengnan Wang^1,2,3^, Xueya Ma ^1,2,3^, Fei Wang ^1,2,3^, Jiaxi Liu^1,2,3^, Xinyu Li^1,2,3^, Haichao Yang^1,2,3^, Fan Zhang^2,3^, Haitao Shen^4^, Noel J. Buckley^5,8^, Nikita Gamper^1,2,6^, Ebenezer N. Yamoah^7#^ and Ping Lv^1,2,3,9#^

^1^ Department of Pharmacology, Hebei Medical University, The Key Laboratory of New Drug Pharmacology and Toxicology, Shijiazhuang, Hebei 050017, China

^2^ Center for Innovative Drug Research and Evaluation, Institute of Medical Science and Health, Hebei Medical University, Shijiazhuang, Hebei Province, China

^3^ The Key Laboratory of Neural and Vascular Biology, Ministry of Education, Hebei Medical University, Shijiazhuang, Hebei 050017, China

^4^Lab of Pathology, Hebei Medical University, Shijiazhuang, Hebei 050017, China

^5^ Department of Psychiatry, University of Oxford, Oxford, United Kingdom

^6.^Faculty of Biological Sciences, University of Leeds, Leeds, United Kingdom,

^7^ Department of Physiology and Cell Biology, School of Medicine, University of Nevada, Reno, Reno, NV 89557, United States

^8^ Kavli Institute for Nanoscience Discovery, University of Oxford, Oxford, United Kingdom

^9^The Hebei Collaboration Innovation Center for Mechanism, Diagnosis and Treatment of Neurological and Psychiatric Disease, Hebei Medical University, Shijiazhuang, Hebei 050017, China

Number of Figures: 8

Supplementary Figures: 8

Number of supplementary files : 1a,1b,1c

**Corresponding author:**

Ebenezer N. Yamoah

Department of Physiology and Cell Biology, School of Medicine

University of Nevada, Reno,

Reno, NV 89557, United States

E-mail address: enyamoah@gmail.com

Ping Lv

Department of Pharmacology

Hebei Medical University

Shijiazhuang, Hebei 050017, China.

E-mail address: lping77@hotmail.com

**Supplementary File 1a**

The primer sequences for genotyping

| Primer | Sequence(5' - 3') |
| --- | --- |
| Rest forward | GCCCACACGCCAGGCCTGAAC |
| Rest reverse | TCGGTCCCGAGGCTCGAAGTGG |
| Rest DEL forward | GCACAAGCAGCGCACTATTA |
| Rest DEL reverse | CGGGCG GATTAGGTA AGTTT |
| Atoh1 forward | CCGGCAGAGTTTACAGAAGC |
| Atoh1 reverse | ATGTTTAGCTG GCCCAAATG |
| Atoh1 control forward | CTAGGCCACAGAATTGAAAGATCT |
| Atoh1 control reverse | GTAGGTGGAAATTCTAGCATCATCC |

**Supplementary File 1b**

The primer sequences for real-time PCR

| Primer | Sequence(5' - 3') |
| --- | --- |
| Na_v_1.1 forward | CTCCTCAAATGGGTGGCCTA |
| Na_v_1.1 reverse | GATTTGATGGCCCCGAGTTC |
| Na_v_1.6 forward | CCTGGTGTTCACTGGGATCT |
| Na_v_1.6 reverse | AGGCCCAGCTCCATTAAACT |
| Na_v_1.7 forward | CGAGAGCGGAGAGATGGATT |
| Na_v_1.7 reverse | TTCGCTTCAGTGTGGTTGTG |
| K_v_3.4 forward | TTCTTTTGCCTGGACACTGC |
| K_v_3.4 reverse | GCCACAACGTCGATAATGCT |
| K_v_1.1 forward | GGCGAGAGGGGTTTCCAAAT |
| K_v_1.1 reverse | GGCTTCAGAGCCAGAAGGTT |
| K_v_1.2 forward | GGAGGCTCTGGTACCCATCT |
| K_v_1.2 reverse | CCTTTGGAAGGAAGGAGGCA |
| HCN1 forward | ACATGCTGTGCATTGGTTATGGCG |
| HCN1 reverse | AACAAACATTGCGTAGCAGGTGGC |
| HCN2 forward | ACTTCCGCACCGGCATTGTTATTG |
| HCN2 reverse | TCGATTCCCTTCTCCACTATGAGG |
| K_v_7.2 forward | AGGAAGCCGTTCTGTGTGAT |
| K_v_7.2 reverse | GCAGAGGAAGCCAATGTAC |
| K_v_7.3 forward | GAAGACAGGGGCTATGGGAAT |
| K_v_7.3 reverse | GTTTTGGAGTGGATGGAGGTC |
| K_v_7.4 forward | ATGGGGCGCGTAGTCAAGGT |
| K_v_7.4 reverse | GGGCTGTGGTAGTCCGAGGTG |

**Supplementary File 1c**

The primer sequences for single-cell RT-PCR

| Primer | Sequence(5' - 3') |
| --- | --- |
| Primer sequences in Figure 1B  Rest forward | CGACACATGCGGACTCATTC |
| Rest reverse | AGAGGCCACATAATTGCACTG |
| Primer sequences in Figure 1-figure supplement 1  Rest forward  Rest reverse | GGTCTGATCCCGCTCCG  TGGCCATAACTGTACTCCTCTG |
| GAPDH forward | CCAGCCTCGTCCCGTAGACA |
| GAPDH reverse | CTCGTGGTTCACACCCATCA |
